# Cross-laboratory validation of the OncoScan® FFPE Assay, a multiplex tool for whole genome tumour profiling

**DOI:** 10.1186/s12920-015-0079-z

**Published:** 2015-02-18

**Authors:** Joseph M Foster, Assa Oumie, Fiona S Togneri, Fabiana Ramos Vasques, Debra Hau, Morag Taylor, Emma Tinkler-Hundal, Katie Southward, Paul Medlow, Keith McGreeghan-Crosby, Iris Halfpenny, Dominic J McMullan, Phil Quirke, Katherine E Keating, Mike Griffiths, Karen G Spink, Fiona Brew

**Affiliations:** Affymetrix UK Ltd, High Wycombe, UK; Leeds Institute of Cancer and Pathology, Section of Tumour Biology and Pathology, Leeds University, Leeds, UK; Almac Diagnostics, Craigavon, Northern Ireland UK; West Midlands Regional Genetics Laboratory, Birmingham, UK

**Keywords:** OncoScan, Tumour, Validation, FFPE, Copy number, Somatic mutations, LOH, Reproducibility

## Abstract

**Background:**

Adoption of new technology in both basic research and clinical settings requires rigorous validation of analytical performance. The OncoScan® FFPE Assay is a multiplexing tool that offers genome-wide copy number and loss of heterozygosity detection, as well as identification of frequently tested somatic mutations.

**Methods:**

In this study, 162 formalin fixed paraffin embedded samples, representing six different tumour types, were profiled in triplicate across three independent laboratories. OncoScan® formalin fixed paraffin embedded assay data was then analysed for reproducibility of genome-wide copy number, loss of heterozygosity and somatic mutations. Where available, somatic mutation data was compared to data from orthogonal technologies (pyro/sanger sequencing).

**Results:**

Cross site comparisons of genome-wide copy number and loss of heterozygosity profiles showed greater than 95% average agreement between sites. Somatic mutations pre-validated by orthogonal technologies showed greater than 90% agreement with OncoScan® somatic mutation calls and somatic mutation concordance between sites averaged 97%.

**Conclusions:**

Reproducibility of whole-genome copy number, loss of heterozygosity and somatic mutation data using the OncoScan® assay has been demonstrated with comparatively low DNA inputs from a range of highly degraded formalin fixed paraffin embedded samples. In addition, our data shows examples of clinically-relevant aberrations that demonstrate the potential utility of the OncoScan® assay as a robust clinical tool for guiding tumour therapy.

**Electronic supplementary material:**

The online version of this article (doi:10.1186/s12920-015-0079-z) contains supplementary material, which is available to authorized users.

## Background

Formalin fixed paraffin embedded (FFPE) samples represent approximately 80-90% of all archived solid tumours with more than a billion samples stored in hospitals and tissue banks across the globe [1]. FFPE samples have been historically used for microscopic analysis of tumour material. Fixation of tissues is undertaken under very variable conditions and in most is satisfactory for routine assessments. The genetic profiling of FFPE samples provides valuable clinical information for diagnosis, prognosis and prediction of treatment outcomes. Unfortunately nucleic acids and other macromolecules suffer considerable deterioration in quantity and quality as a result of the routine FFPE process [2], complicating any downstream molecular assay. Characteristics of DNA isolated from FFPE are short fragment length, DNA-protein crosslinks and base damage [3]. The problem of utilising FFPE samples is further compounded by the limited amount of DNA that can be extracted. These inherent features of DNA extracted from FFPE tissue can lead to assay failure, errors in amplification and sequencing, and subsequent misinterpretation of results since most assays are optimised to work with high quality and sufficient quantities of DNA isolated from fresh tissue.

The OncoScan® FFPE Assay Kit (OncoScan® assay) has been optimised for whole genome copy number (CN), loss of heterozygosity (LOH) and somatic mutation (SM) detection from highly degraded FFPE samples. Based on molecular inversion Probe (MIP) [4] technology, this SNP assay provides whole genome coverage with increased resolution in approximately 900 cancer genes while also currently detecting 74 clinically actionable SMs commonly found in 9 cancer genes. The assay requires ≤ 80ng of input DNA and can utilise extremely degraded DNA, with probes having a genomic footprint of just 40bp, making it ideally suited to FPPE samples. The OncoScan® assay has been adopted in a number of research and translational laboratories and its utility across a range of tumour types reported [5-8].

The importance and value of whole genome CN data has been underestimated when regarding the future of clinical practice for the guidance of tumour therapy [9]. Recent literature describes a growing occurrence of copy number aberrations with both prognostic [10], diagnostic and therapy prediction value. It has recently been suggested that CN rather than SM may be of greater importance to guiding therapeutics in certain tumour types due to the availability of drugs that target copy number aberrations [11]. Analysis of TCGA datasets has revealed that several solid tumours are driven by copy number rather than by somatic mutations. These have been designated “C class tumours” and include ovarian cancer, breast cancer, squamous cell lung cancer, head and neck cancers [11]. Whole genome approaches to CN profiling potentially offer considerable advantages in the detection of diverse clinically actionable aberrations over more established single locus, lower resolution targeted tests such as FISH. Currently, there are well defined molecular signatures associated with particular tumour types e.g. *HER2* amplification (with or without *TOP2A* amplification) in breast cancer. However, as more whole genome CN data is collected, the same signatures, albeit at a differing frequency, are being found in a range of tumour types [12,13]. This questions the efficiency of current methods that target only a handful of common aberrations in specific tumour types and points to the need for wider profiling of clinically-relevant aberrations regardless of tumour type. Technology platform aside, it is expected that whole genome CN data, in combination with SM information, will play an important role in both basic research and the future of clinical practice [11].

This study aims to evaluate the reproducibility of all aspects of the OncoScan® assay and where orthogonal pre-validation of SM data is already freely available, estimate the platform’s SM sensitivity. 162 FFPE tumour samples covering six different tissue types were collected and each run on the OncoScan® assay in triplicate across three independent laboratories. Quality Control (QC) metrics, CN, LOH and SM results were compared across the laboratories and SM data compared with results from orthogonal technologies. From these samples we have identified numerous examples of clinically-relevant aberrations that highlight the benefits of combined whole genome CN, LOH and SM approaches in various tissue types which are illustrated and discussed.

## Methods

A collection of 162 FFPE tissue samples of colorectal, breast, ovarian, melanoma, prostate and lung cancers were obtained through West Midlands Regional Genetics Laboratory (WMRGL) from the Human Biomaterials Resource Centre at the University of Birmingham and from the Leeds Institute of Cancer Pathology (LICAP). Samples sourced at WMRGL were collected through the University of Birmingham’s Human Biomaterials Resource Centre (HBRC), an HTA-licensed facility dedicated to the collection, processing and storage of appropriately consented, high quality human biomaterials for research. The HBRC is ethically approved (North West 5 Research Ethics Committee, Haydock Park; Ref 09/H1010/75) and so samples may be released to this study without the requirement for project-specific ethical approval. The HBRC has been supported through Birmingham Science City *-* Experimental Medicine Network of Excellence project. Samples sourced at LICAP were ethically approved by NRES Committee North East - York for use under the study title “Prognostic and predictive factors in Colorectal Cancer”; Ref 08/H0903/62. It is for the purpose of evaluating the real-world reproducibility of OncoScan® that we do not mandate the protocol upstream of the OncoScan® assay. The FFPE blocks were up to 23 years old. With a variety of sample handling/preparation techniques, we believe this dataset well represents the diversity of FFPE tumour material available in the clinic and sample repositories. For Colorectal samples collected at LICAP and Lung samples collected from WMRGL five to ten sections (scrolls), each 5 to 6 μm thick, were cut from the FFPE blocks. The FFPE scrolls were mounted on to Superfrost slides, a single slide was stained with haematoxylin and eosin to identify tumour enriched regions and was used as a template for the macrodissection of this region from the unstained slides. The tumour-enriched region of the remaining scrolls of colorectal and lung cancer was cut and deposited into a 1.5 ml Eppendorf tube for DNA extraction. For all the other tumour types used in this study which were collected at WMRGL, macrodissection of tumour enriched region was not performed; rather the entire scroll was used for DNA extraction. DNA was extracted using QIAamp DNA FFPE Tissue Kit (QIAGEN) following the manufacturer recommended protocol and according to the Standard Operating Procedure (SOP) of each lab.

DNA was quantified using the Quant-iT™ PicoGreen® dsDNA Assay Kit (Life Technologies) following the manufacturer’s recommended protocol. The concentration of the DNA stock was adjusted to 12 ng/μl using reduced EDTA TE buffer (10 mM Tris-HCl, 0.1 mM disodium EDTA, pH 8) or by vacuum evaporation depending on the starting concentration. In preparation for the assay the 12 ng/μl DNA was plated at 6.6 μl/well (giving a total of 79.2 ng DNA/well) in MicroAmp Optical 96-well reaction plates (Life Technologies); this was done in triplicate to provide each of the three test laboratories with enough DNA to run the assay. The DNA plates were sealed with Micro-Amp Clear Adhesive Film (Applied Biosystems) and kept frozen at -20°C. Two plates were shipped to the other laboratories on dry ice and the third plate was kept frozen at the sample origin laboratory until required for the assay. Five Plates containing 56 DNA samples were sent out from LICAP to WMRGL and Almac Diagnostics (Almac) and seven plates containing 106 DNA samples were sent from WMRGL to LICAP and Almac.

The OncoScan® assay utilizes the MIP technology, for the identification of CN alterations, LOH and SMs. MIP probes in the OncoScan® assay capture the alleles of over 220,000 SNPs at carefully selected genomic locations, distributed across the genome with increased probe density within ~900 cancer genes. Furthermore, the MIP probes also enable detection of 74 frequently tested somatic mutations in nine genes implicated in cancer (*BRAF*, *KRAS*, *EGFR*, *IDH1*, *IDH2*, *PTEN*, *PIK3CA*, *NRAS* and *TP53*).

Cross-laboratory comparison of the OncoScan® (Affymetrix Inc) assay’s performance was evaluated by running the assay at the three test laboratories on plates containing the same set of samples and by following the recommended protocol outlined in the OncoScan® assay manual. In short, the copy number and somatic mutation MIP probes were added to the FFPE DNA in each well and allowed to anneal at 58°C overnight (16-18h) after an initial denaturation at 95°C for 5 min. Each sample was then split into two wells and gap fill reaction was performed by adding dATP (A) and dTTP (T) (A/T) in one well and dGTP (G) and dCTP (C) (G/C) to the other well. Uncircularised MIP probes and genomic DNA were digested by using a cocktail of exonucleases, leaving only MIP probes that have been gap filled by the A/T or G/C nucleotides. The circular MIP probes were then linearized using a cleavage enzyme and amplified by PCR. Following a second round of PCR amplification the 120bp amplicons were cleaved into two fragments with the Haeiii enzyme, of which the smaller (44bp) fragment is to be hybridized onto the OncoScan® assay arrays. Samples were then mixed with hybridization buffer and injected into the OncoScan® assay arrays where they were allowed to hybridize for 16-18 h. At the end of the hybridization period, arrays were stained and washed using the GeneChip® Fluidics Station 450 and loaded into the GeneChip® Scanner 3000 7G (Affymetrix) where array fluorescence intensity was scanned to generate array images (DAT file). Array fluorescence intensity (CEL) files were automatically generated from DAT files by the Affymetrix® GeneChip® Command Console® (AGCC) Software version 4.0. By following the manufacturer’s recommended protocol we were able to complete an OncoScan® assay run, of up to 46 samples (plus positive and negative controls) over a 48-hour period.

In total, 162 unique samples were collected from two laboratories; LICAP and WMRGL and pre-validated for a set of somatic mutations (SMs) via a wide range of technologies at the laboratory they were sourced from (See Additional file 1: Table S1).

Array fluorescence intensity data (CEL files), generated by Affymetrix® GeneChip® Command Console® (AGCC) Software version 4.0 were processed using OncoScan® Console software version 1.1.034 to produce OSCHP files and a set of QC metrics. Among the list of QC metrics generated by OncoScan® Console, the two most important for the evaluation of assay performance are MAPD and ndSNPQC. For the purpose of cross-laboratory comparison, samples with “out of bounds” QC metrics were not excluded as the focus is on reproducibility across laboratories, regardless of basal QC level. However, the accuracy of SM calls is strongly correlated to ndSNPQC, so for the purpose of Orthogonal SM validation, any samples that were classed as “out of bounds” according to the product guidelines (ndSNPQC <26) were excluded. The remaining samples (OSCHP files) were then analysed for the concordance of OncoScan® assay SM calls with pre-validated SM calls and cross-laboratory validation of the breadth of QC and platform outputs. Analysis was performed using a set of custom scripts written in the statistical software programming language R, version 3.1.0. Specific analyses were carried out as follows:

### QC comparison

To test the inter-laboratory reproducibility of QC metrics, the MAPD distributions of the 3 laboratories were first compared by a one-way ANOVA. Upon finding any significant difference at the 5% level a follow up pairwise test was performed to identify which laboratories did not have the same MAPD mean. This process was repeated for ndSNPQC.

### CN comparison

For each triplicate, a set of 3 CN profiles were created, each CN profile is made up of ~ 220,000 CN probes distributed across the genome. Each CN profile was simplified from numerical CN values (e.g. 1,2,3,4,7,9) to CN Call e.g. (“homozygous loss”, “loss”, “normal diploid”, “gain”, “high gain”, where “high gain” is defined as CN >3). For each inter-probe distance across the entire length of the genome if all three laboratories agree on the CN Call then that region is said to be in 100% agreement. The percentage of the entire genome for which all three laboratories agree on the CN Call is reported as the genome-wide percentage CN Call Agreement. This was summarised across all triplicates to calculate the median genome-wide percentage CN Call Agreement.

### LOH comparison

For each triplicate, a set of 3 LOH profiles were created, each LOH profile is made up of ~ 220,000 CN probes distributed across the genome. For each inter-probe distance across the entire length of the genome if all three laboratories agree on the LOH Call then that region is said to be in 100% agreement. The percentage of the entire genome for which all three laboratories agree on the LOH Call is reported as the genome-wide percentage LOH Call Agreement. This was summarised across all triplicates to calculate the median genome-wide percentage LOH Call Agreement.

### TuScan comparison

For each triplicate the percentage agreement of ploidy is calculated (i.e. 0%, 66% or 100%). The mean of all triplicate percentage agreement of ploidy was calculated and returned as the average percentage agreement of ploidy. The same process was repeated for aberrant cell fraction estimates to report the average percentage agreement of aberrant cell fraction.

### SM comparison

SM calls are often closely related to ndSNPQC, for the purpose of this comparison triplicates for which all sample runs had ndSNPQC ≥26 (“in-bounds”) were collected. The SM calling software supplied with the OncoScan® assay translates signal intensity from the arrays into a Mutation Score. To this Mutation Score two thresholds are applied on an SM by SM basis that classify Mutation Scores into one SM Call of “Undetected”, “Lower confidence” or “High confidence”. The SM Calls used throughout this study are derived from the default Mutation Score thresholds supplied in the software. On each triplicate the Mutation Call for each SM was compared between laboratories and the % concordance reported, where concordance is defined as the maximum of the percentage of samples which agree on Mutation call. For example where two sample runs agree on a “high confidence” call and the other sample run returns a “lower confidence” call the maximum % of samples that agree with each other is 66%. This flavour of SM concordance tests only whether replicates across laboratories agree, regardless of any pre-validation, sensitivity in regards to pre-validation is described in the subsequent section.

### Orthogonal SM validation

The colorectal cancer samples provided by LICAP were pre-validated by pyrosequencing for BRAF:p.V600E:c.1799T > A and KRAS:p.G12D/V:c.35G > A/T, triplicate runs of 22 samples each (66 sample runs). The somatic mutation pre-validation of samples obtained from WMRGL were performed with Sanger sequencing (*PTEN*, *TP53*, *BRAF*); pyrosequencing (*KRAS*, *NRAS*); and RT-qPCR (*EGFR* and *PIK3CA*). Each SM on the OncoScan® assay returns a Mutation Score derived from the signal present on the OncoScan® array. All pre-validation data was previously initiated outside of the scope of this study and utilised in this study on an ad hoc basis. SM Calls were split into three categories; “Undetected”, “Lower Confidence” and “High Confidence” predefined by a set of SM specific Mutation Score thresholds. “Lower Confidence” SM Calls were treated as “undetected” (negative) for the purpose of calculating sensitivity.

### Single sample clinical analysis

After conversion of CEL files by OncoScan® Console, OSCHP files were loaded into BioDiscovery's Nexus Express for OncoScan® for analysis of clinically relevant CN and LOH events. In parallel SMs were visualised in Somatic Mutation Viewer and cross referenced with the CN data for correlation with CN events. Four types of interesting event were searched for; SM in the presence of Copy Neutral LOH, common tissue specific amplification, general amplifications seen across tissue types and samples for which there were multiple clinically actionable amplifications. For this analysis amplifications were defined as a ratio of “gene of interest”: “baseline for that genome” of >2.2.

## Results

The reproducibility of key CN, LOH, SM and QC metrics were evaluated from OncoScan® assay data produced by the three laboratories on the same 162 samples. The raw microarray data (CEL files) and processed microarray data (OSCHP files) have been made publicly available via the Array Express database (www.ebi.ac.uk/arrayexpress) [14] under accession number E-MTAB-2914. For clarity the results have been subdivided into specific sections devoted to each of these different metrics.

### QC comparison

**Figure 1:**
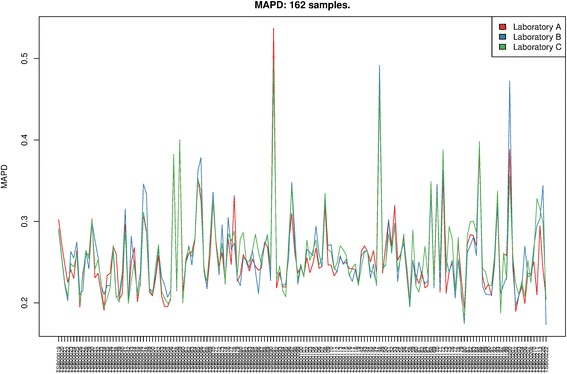
**MAPD of 162 samples ran in triplicate across three laboratories with the Affymetrix OncoScan® assay.** The inter-sample MAPD variance is larger than the mean of the intra-sample MAPD variances. These inter-sample MAPD differences are governed by inherent properties of the samples.

**Figure 2:**
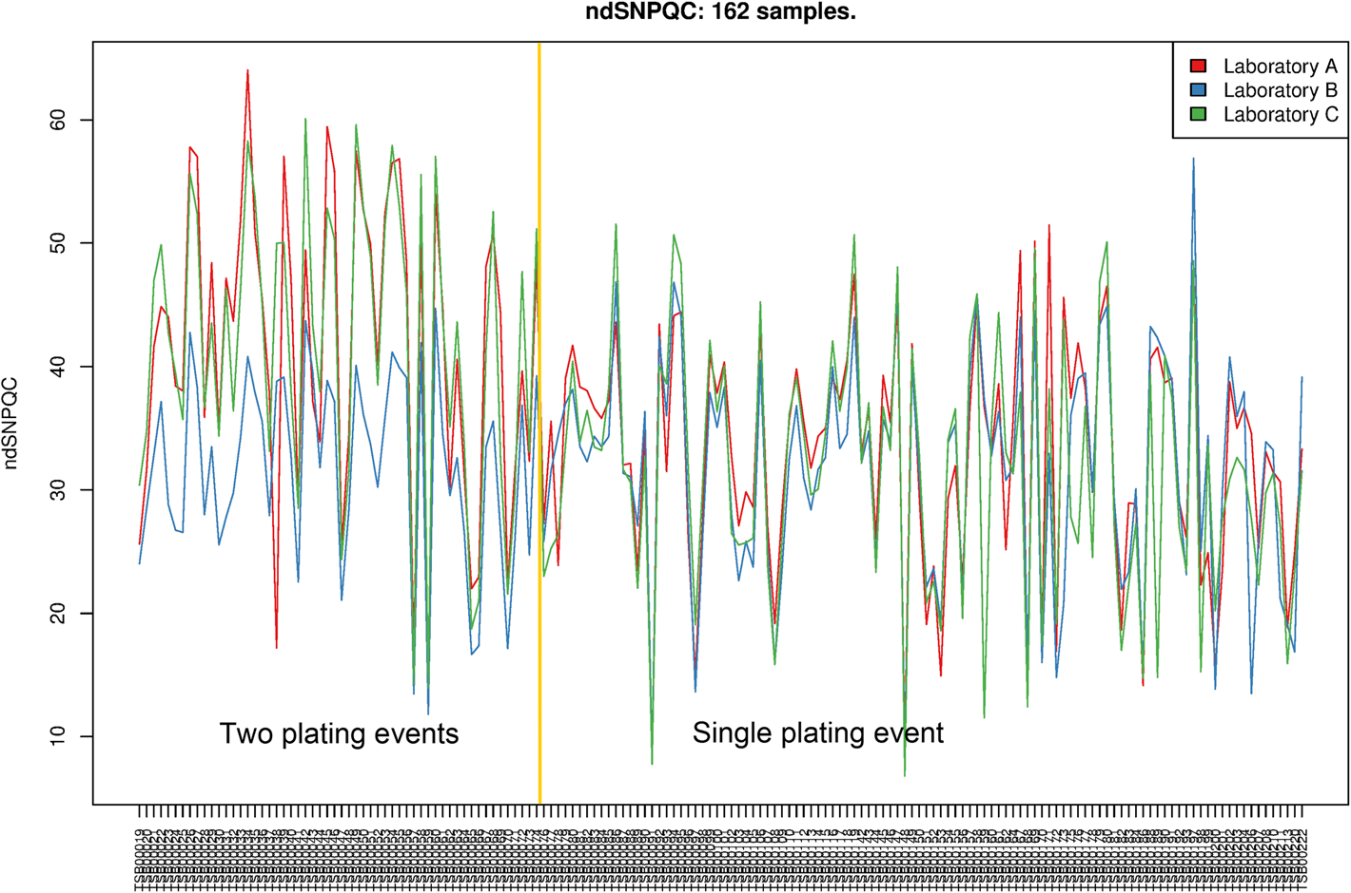
**ndSNPQC of 162 samples ran in triplicate across three laboratories with the Affymetrix OncoScan® assay.** The inter-sample ndSNPQC variance is larger than the mean of the intra-sample ndSNPQC variances. These inter-sample ndSNPQC differences are governed by inherent properties of the samples. TSBIDs 00019-74 have a markedly lower ndSNPQC in sample-runs performed at laboratory B. Investigating the source of this difference we identified that the DNA for these samples was plated in two separate events and only laboratory B used plates from the second plating event.

Routine QC metrics are generated during the conversion of CEL files to OSCHP file by the OncoScan® Console software. Two metrics are used to classify a sample run as “in-bounds” or “out of bounds”, these are; Median Absolute Pairwise Difference (MAPD) and normal diploid SNP Quality Control (ndSNPQC), for which descriptions can be found in the List of Abbreviations. The thresholds for determining “in-bounds” or “out of bounds” were the defaults supplied by the manufacturer (MAPD < =0.3 and ndSNPQC > =26 to be classified as “in-bounds”). Distribution of MAPD between laboratories showed no statistically significant differences at the 5% level (ANOVA). However, it is clear from Figure 1 that properties inherent to the sample have a profound effect on the MAPD values, likewise for ndSNPQC demonstrated in Figure 2. ANOVA followed by a pairwise test of ndSNPQC between laboratories showed a statistically significant difference at the 5% level between laboratory B and the other two laboratories. This difference was isolated to the first 56 samples only (TSB IDs 00019-74). Enquiry into the difference between the first 56 and the later 106 samples with the sample source sites revealed that the first 56 were plated in two independent events at laboratory C, with laboratory C and laboratory A receiving DNA from the first plating event and laboratory B from the second plating event. The remaining 106 samples were all plated in a single plating event at laboratory C. In order to evaluate the effect on CN and LOH results we calculated the genome-wide percentage CN Call agreement for the 56 samples affected by the plating issue on pairs of labs rather than all three sites. We compared the distribution of genome-wide percentage CN Call agreement involving the affected Lab B (LabA-LabB and LabB-LabC comparisons) versus those not involving Lab B (LabA-LabC comparison). The resulting test showed no significant difference between the mean genome-wide percentage CN Call agreement (p =0.773) and so the effect on CN and LOH can be considered negligible. Thus for a DNA sample of a given quality and quantity, the QC metrics remain stable even when the assay is performed in different laboratory setups.

### CN/LOH comparison

**Figure 3:**
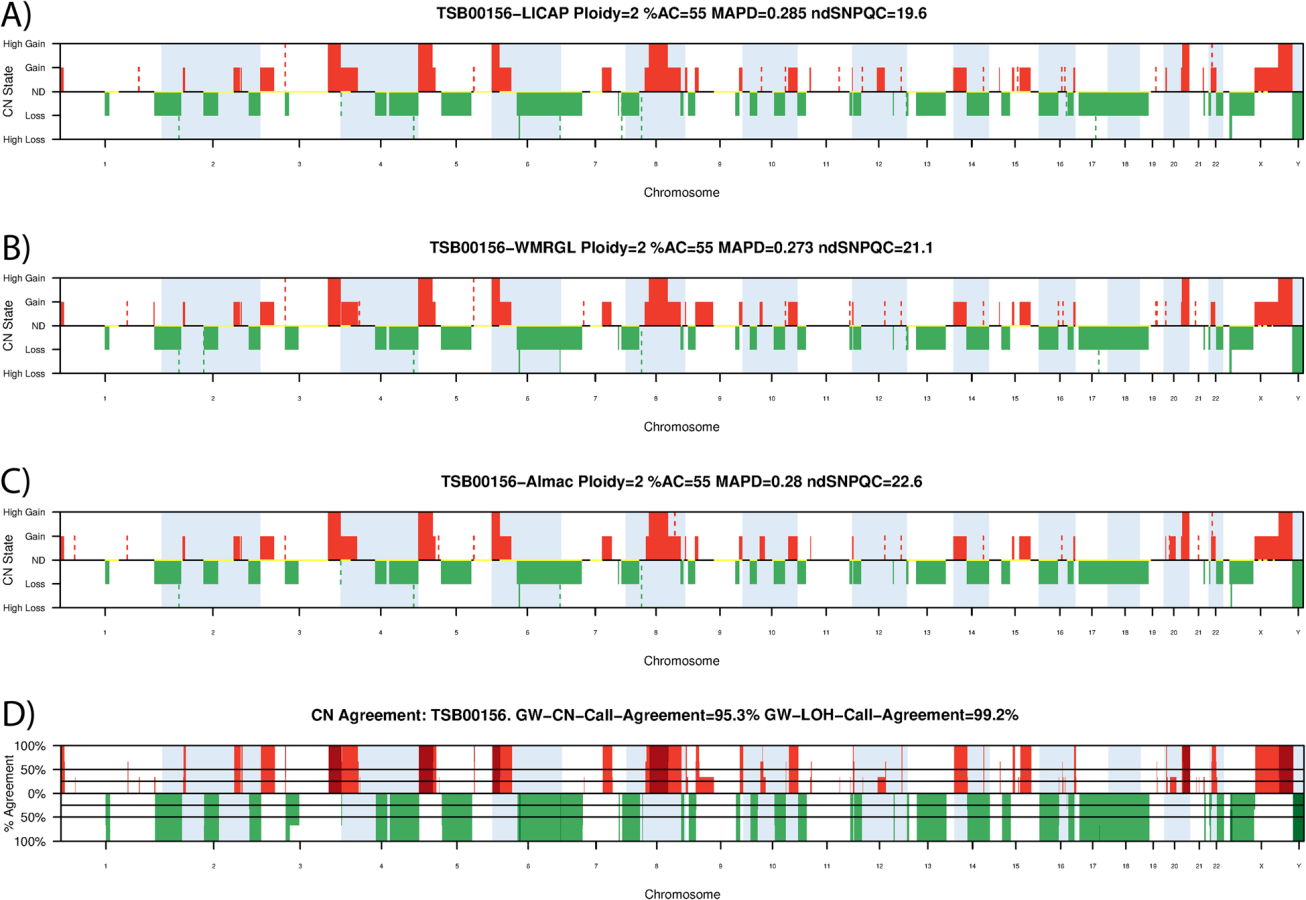
**Copy Number profiles for sample TSB000156, with the CN agreement plot.** Prior to plotting, numeric CN is translated to CN Call; “High Loss”, “Loss”, “ND”, “Gain” and “High Gain” with numeric CN conditions; “CN < 1”, ”1 ≤ CN < 2”, “CN = 2”, “3 ≥ CN > 2” and “CN > 3” respectively. Events smaller than 1 Megabase are plotted as dashed lines. Red represents CN gains (dark red is “High Gain”), green represents loss (dark green is “High loss”). Panels **A)**, **B)** and **C)** show the replicate CN profiles of sample TSB000156 run at three independent laboratories. Regions of LOH are plotted along the CN State = ND line as yellow bars. Panel **D)** shows the percentage of sites that agree on a given CN event along the length of the whole genome. GW-CN-Call-Agreement describes the percentage of the length of the genome for which all three independent laboratories agreed on the CN Call, in this case 94.7%. Similarly for LOH the percentage of the length of the genome for which all three independent laboratories agreed on LOH status is 99.1%. Noteworthy in this example is the out of bounds ndSNPQC value (<26) producing remarkably reproducible CN data.

A set of three CN profiles was plotted for each triplicate (one for each lab), the percentage CN Call Agreement along the length of the genome was also plotted and the genome-wide percentage CN Call agreement calculated (See Figure 3, See Additional file 2). For all triplicates the median genome-wide percentage CN Call Agreement was 97%. This indicates that when a sample is run across 3 laboratories, 97% of the length of the genome can be expected to agree on the CN Call across all three laboratories. As part of the three CN plots, LOH calls were also included (plotted in yellow along the CN State = ND line). The genome-wide percentage LOH call agreement was calculated for each triplicate, and the median taken across all triplicates to give a median genome-wide percentage LOH agreement of 99%.

### TuScan comparison

During analysis of the CEL files the TuScan algorithm generates an estimate of tumour ploidy and aberrant cell fraction. The percentage concordance was calculated for ploidy and aberrant cell fraction for each triplicate. For example, if two of the three laboratories agreed that the ploidy of a sample was 4 then the percentage concordance was 66%. The mean of the set of triplicates where all sample runs had ndSNPQC ≥ 26 was calculated as 97% for ploidy and 90% for aberrant cell fraction.

### SM comparison

Each sample run produces a “High Confidence”, “Lower Confidence” or “Undetected” call for each of the 74 SMs. The mean percentage concordance of all SMs for triplicates where all sample runs have ndSNPQC ≥ 26 was 93%. For a detailed summary by SM and ndSNPQC see Additional file 1: Table S3.

### Orthogonal SM validation

**Figure 4:**
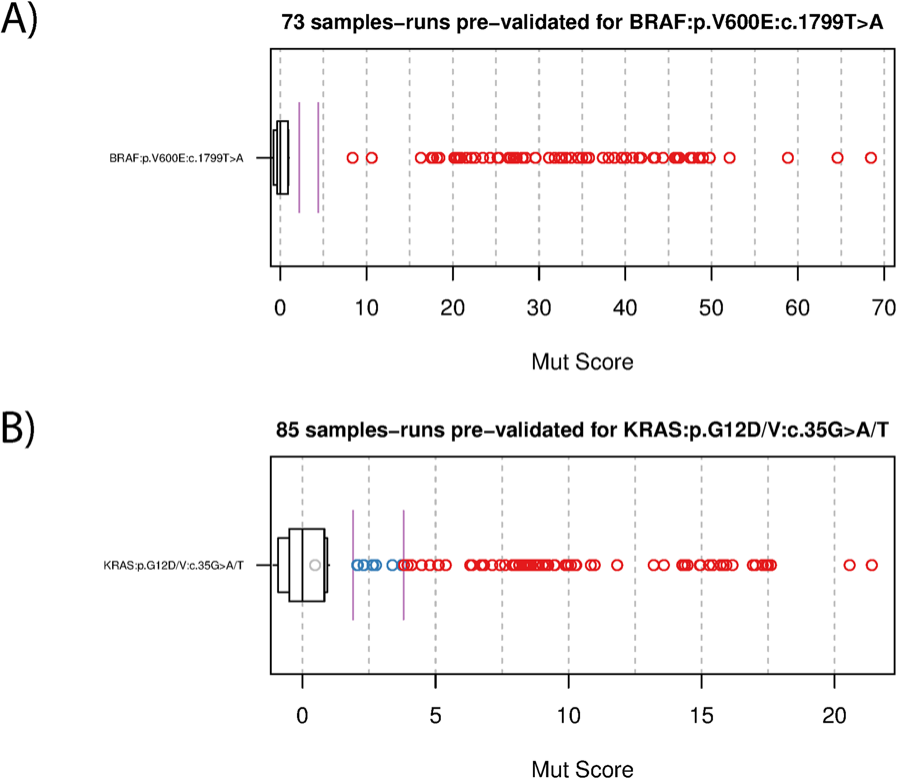
**Marker view of Mutation Score for A) BRAF:p.V600E:c.1799T > A and B) KRAS:p.G12D/V:c.35G > A/T.** Each point represents a sample, with red points representing a high confidence calls, blue points representing a low confidence call and grey point representing an undetected call. The vertical purple lines mark the lower confidence threshold and the high confidence threshold for mutation calls. Each box plot indicates the distribution of Mutation Score for hundreds of normal samples in the universal reference believed to be absent for that SM. **A) S**hows the Mutation Scores for 73 samples pre-validated for BRAF:p.V600E:c.1799T > A. The overall OncoScan® assay high confidence call concordance for BRAF:p.V600E:c.1799T > A to the pre-validated SM calls is 100%. **B)** shows the Mutation Scores for 85 samples pre-validated for KRAS:p.G12D/V:c.35G > A/T. The overall OncoScan® assay high confidence call concordance for KRAS:p.G12D/V:c.35G > A/T to the pre-validated SM calls is 89%.

Where available, orthogonal SM data was collected from sample source sites. This data was originally collected as part of routine clinical diagnostic testing or for the purpose of basic research on samples used here as part of other studies. No orthogonal SM validation data was generated expressly for this study. Out of the 74 SMs detectable by the OncoScan® assay, 18 SMs were pre-validated to be present in at least one sample by an orthogonal technology (RT-qPCR, Sanger sequencing or pyrosequencing). For each SM, all samples pre-validated to be positive for the SM were collected and the Mutation Score plotted. Since each sample was run at all three laboratories, every sample has three sample runs, but only those with ndSNPQC ≥26 (QC metrics in/out of bounds threshold) are present in the Mutation Score plots. Of the 18 pre-validated SMs, 2 were present in large numbers of sample runs; BRAF:p.V600E:c.1799T > A and KRAS:p.G12D/V:c.35G > A/T; with 73 and 85 sample runs respectively. Figure 4A shows the Mutation Score of 73 sample runs pre-validated for BRAF:p.V600E:c.1799T > A. All sample runs exceeded the high confidence threshold, which resulted in 100% sensitivity with respect to the pre-validation data. Figure 4B shows the Mutation Score of 85 sample runs pre-validated for KRAS:p.G12D/V:c.35G > A/T. Of the 85 pre-validated sample runs, the OncoScan® assay detected this mutation with a high confidence call in 76 sample runs, with a lower confidence call in 8 samples runs and as undetected in 1 sample run. Considering only the high confidence calls, the resulting sensitivity with respect to the pre-validation data was 89%. The sensitivity values for the remaining 16 pre-validated SMs did not have a sufficient number of sample runs to provide acceptable resolution on the true sensitivity (See Additional file 1: Table S2). Considering only the higher confidence calls, the mean sensitivity of all pre-validated SMs, weighted by the number of sample runs pre-validated for each SM, interpreted as the overall OncoScan® assay SM sensitivity was 90%.

Copy number, LOH and SM data from each sample was then analysed in tumour specific batches for presence of clinically actionable aberrations and other recurrent, diagnostic or prognostic mutations. Findings of particular interest are discussed below.

## Discussion

The first objective of this study was to demonstrate the reproducibility of the OncoScan® assay when run by operators in different laboratory environments using clinically relevant FFPE material. Assay reproducibility was measured by the inter-laboratory agreement of QC metrics, genomic events such as CN gain/loss, LOH, SM calls, ploidy and aberrant cell fraction estimates.

Our results showed >90% inter-laboratory agreement on all the parameters analysed, demonstrating the robust performance of the OncoScan® assay and its ability to generate highly reproducible results in a variety of laboratory setups with differing SOPs. It is clear from Figures 1 and 2 that intrinsic sample quality plays the largest part in overall inter-sample QC differences, with laboratory environment accounting for little or no difference. This is demonstrated for MAPD by calculating the mean of within-sample variances (variance of each triplicate) against the global variance of all sample-runs, yielding 0.00022 and 0.0025 respectively; an order of magnitude difference. The only major variation between separate laboratory datasets was observed for the ndSNPQC metric. Isolated to only the samples plated in two separate events at laboratory C and run at laboratory B, it is most likely that this is related to an inconsistency during a sample re-plating event where laboratory A and laboratory C received plated DNA from the first plating event and laboratory B received plated DNA from a second, later event. The remaining 106 samples, for which all DNA was plated at the same time at laboratory B, did not show any significant difference between laboratories. The low ndSNPQC at laboratory B for the first 56 re-plated samples points to low DNA input into the assay, which is in agreement with our previous finding that as total DNA input decreases below 40ng of the recommended 80ng, so does ndSNPQC. The differences in ndSNPQC are the most likely explanation for the discrepancies in Mutation Call between laboratories. For each SM, the Mutation Score is calculated from the signal on the array and the underlying variability of that signal in a large reference of samples negative for that SM. For those samples that have Mutation Scores closer to the Mutation Score thresholds that define the boundaries between Mutation Calls (“Undetected”, “Lower Confidence” and “High confidence”), a slight change in ndSNPQC could shift the Mutation Score and result in the change of the SM call from “detected with lower confidence” to “undetected” or from “detected with high confidence” to “detected with lower confidence”. These thresholds can be adjusted with the Somatic Mutation Viewer software provided with the OncoScan® assay, enabling more sophisticated classification of somatic mutation events. The disagreements between laboratories on QC parameters and aberrant cell fraction were minimal. Analysis of genome-wide percentage CN Call agreement between triplicates showed a median of 97% of the length of the genome of a sample run in triplicate agreed on CN Call in all three sample runs. By extension, 3% of the length of the genome showed disagreement in CN Calls between the three sample runs. This disagreement can be attributed to three main sources, two of which are related to the segmentation algorithm; a number of smaller CN gains/losses are sometimes not called if their Log2Ratios and supporting BAF data lay close to the calling boundary, alternatively, small discrepancies in the exact start/end of a larger segment. The third component of the 3% disagreement occurs when there is a disagreement on the ploidy of the sample between laboratories. Whilst rare, when it does occur, disagreement on ploidy results in large reductions to the genome-wide percentage CN Call agreement and therefore the median across all triplicates. This effect is substantially reduced by manual curation of the data which is made possible by the Nexus Express for OncoScan® software. Genome-wide percentage LOH call agreement showed an average 98% of the length of a genome of a sample run in triplicate across three laboratories will agree in LOH call in all three sample runs. The discrepancy is almost entirely explained by small disagreements on the exact start/end of large LOH segment. It is important to note that review of log2ratio pattern by an experienced analyst allows for correction of copy number calls by the algorithm and clinically actionable findings such as biallelic losses or regions of high amplifications can be unambiguously identified by the user. Some level of disagreement is to be expected when dealing with complex algorithms determining discrete results from continuous data and ploidy is no exception. Our reproducibility data showed 3% of triplicates did not unanimously agree on the ploidy. The TuScan algorithm estimates ploidy and aberrant cell fraction simultaneously, determining the most likely solution that fits the observed aberrations in the data. The most likely ploidy and aberrant cell fraction (that best explains the data) is then reported. Rarely (in 3% of the triplicates) there are 2 solutions that fit the data well, but because of some variability one solution is chosen over another in some of the replicates.

The second objective of this study was to demonstrate the validity of the OncoScan® assay in the detection of SMs by using clinical samples pre-validated for a set of SMs by other technologies. To make this assay more reliable to clinical standards, any SMs detected as “lower confidence” were considered undetected. It should be appreciated that in using our stringent approach to somatic mutation calling, any pre-validation errors (false positives) by other technologies would become false negatives in our analysis and lead to underestimation of the sensitivity figures. Of the 9 sample runs pre-validated for KRAS:p.G12D/V:c.35G > A/T with “lower confidence” or “undetected” calls by the OncoScan® assay, 6 have an aberrant cell fraction <20% (TuScan aberrant cell fraction call is “homogeneous”, CN events evident but reduced evidence in BAF data). The 3 remaining sample runs are all from a single sample: TSB00053 which does not have a low aberrant cell fraction. Analysing these 3 sample runs in isolation shows that while the Mutation Score is not high enough to exceed the “lower/high confidence” mutation score threshold and constitute a confident call, KRAS:p.G12D/V:c.35G > A/T is the highest scoring SM in all three sample runs. According to the pyrosequencing pre-validation, this SM was present at 16%. This is in line with the OncoScan® assay product specification of a minimum 20%-25% SM in wild type for the reliable detection of somatic mutations. The CN events for this sample are at much higher frequency than 16%, indicating some level of intra tumour heterogeneity where a minor clone has gained a KRAS:p.G12D/V:c.35G > A/T SM. Again, the Mutation Score thresholds that define the boundaries of mutation call can be manually adjusted in the software to accommodate for out of bounds QC or aberrant cell fraction and in future may lead to more sophisticated SM calling.

Data generated from this validation study were also analysed for identification of SM and CN aberrations of potential clinical relevance for patient stratification or application of targeted therapeutics. Highlighted here are a selection of examples that illustrate the benefits of CN, LOH and SM results from a single platform that can be applied across multiple cancer types.

**Figure 5:**
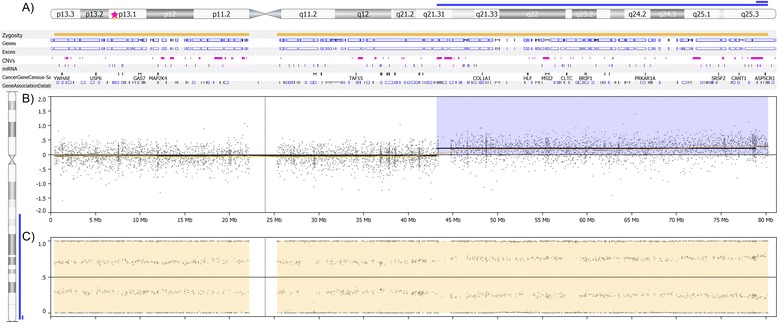
**Whole chromosome 17 CN trace from breast cancer sample TSB00095.** The figure is captured by the Nexus Express for Affymetrix software version 3. **A)** is a karyoview of the chromosome where the blue bar represents a gain and the annotation tracks below highlight genes and other genomic features of interest. **B)** is the log2ratio plot and **C)** the BAF plot. It shows CNNLOH of the entire chromosome with duplication of the distal region of the long arm of chromosome 17. This patient also has a TP53:p.R213*:c.637C > T SM at 17p13.1 annotated as a red star on karyoview.

An ability to identify acquired Copy Number Neutral LOH (CNNLOH) across the whole genome is a key feature of this assay. CNNLOH, sometimes referred to as acquired isodisomy or acquired uniparental disomy (aUPD), has been frequently described in haematological malignancies [15-18] and also in the study of solid tumours [19-21]. This mechanism has been associated with duplication of oncogenic mutations as well as loss of the wild-type allele in the presence of tumour-suppressor mutations. LOH in the region surrounding *TP53* is commonly reported across neoplasms [20,22,23]. In our results, CNNLOH was observed across tumour types with particular recurrence in regions of known key tumour suppressor genes such as *PTEN* and *TP53*. The multiform nature of data derived from the OncoScan® assay adds considerable value to these samples with regions of CNNLOH frequently observed as co-occurring with SMs in both oncogenes and tumour suppressor genes across tumour types. The ability to identify both phenomena through the use of a single assay enables accurate genomic profiling for rapid patient stratification. Concurrent CNNLOH of 17p regions (including *TP53*) together with known, clinically significant *TP53* mutations were observed in colorectal, lung, ovarian and breast cancer samples examined during our study (see Figure 5). Deletion of 17p was also a common finding across the samples examined. This data allows for rapid confirmation of the second hit of LOH in the presence of a SM as removing the wild-type allele and therefore contributing to the development of the cancer genomes in these patients. Biallelic inactivation of *TP53* is proposed as a positive predictor for multiple targeted therapies currently being trialled, as summarised in the TARGET database compiled by van Allen et al [24].

**Figure 6:**
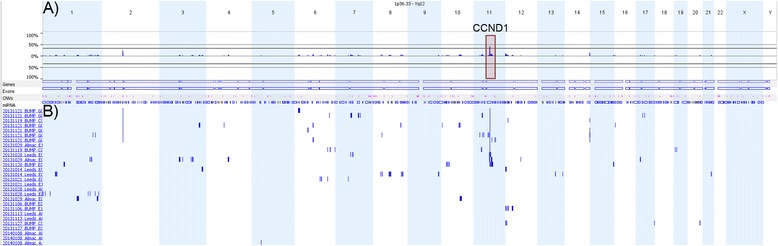
**Multi-sample comparison of 28 different breast cancer samples with focal amplification of CCND1.** The figure is a whole genome view of the CN events captured by the Nexus Express for Affymetrix software version 3. **A)** Is the aggregate plot for these 28 samples which denotes the percentage of samples that contain focal amplification in a region. **B)** Depicts a set of single sample focal amplification event traces. The blue lines denote a region of focal amplification. Focal amplification of CCND1 is visible on the first 12 out of 28 samples on chromosome 11.

Regions of High Focal Amplification (HFA) were frequently observed across tumour types and accurately quantified in a whole genome context by the OncoScan® assay. Many of these aberrations were specific to the primary tissue of origin of the cancer, for example, lung developmental transcription factor *NKX2-1* in lung cancer [25], *AURKA* in colon cancer [26] and *AR* in prostate cancer [27]. Conversely, several others were observed across cancer types, highlighting cross-cancer similarities. *AKT2* amplification, potentially predicting sensitivity to *AKT*/*MTOR* inhibitors [28], was highly amplified in breast, lung and ovarian cancer samples in our dataset. *ERBB2* (*HER2*) amplification, predicting sensitivity to anti-*HER2* therapy [29] was also observed in our cohort in breast, colorectal, lung and ovarian cancer samples. *CCND1* at 11q13, a well-documented region of high amplification [30], represented the gene most commonly involved in HFA in our dataset, across multiple tumour types. *CCND1* focal amplification was observed in samples from patients with breast cancer, melanoma and ovarian cancer. *CCND1* most specifically represented the most common focal amplification in the breast cancer patients analysed, 12/28 (43%) (see Figure 6). Such cross-cancer similarity and recurrence of targetable aberrations highlights the potential for increased cross-cancer therapeutics and argues for re-classification of some tumours away from primary tissue of origin and towards a system based on the biological pathways that are mutated in the cancer genome.

**Figure 7:**
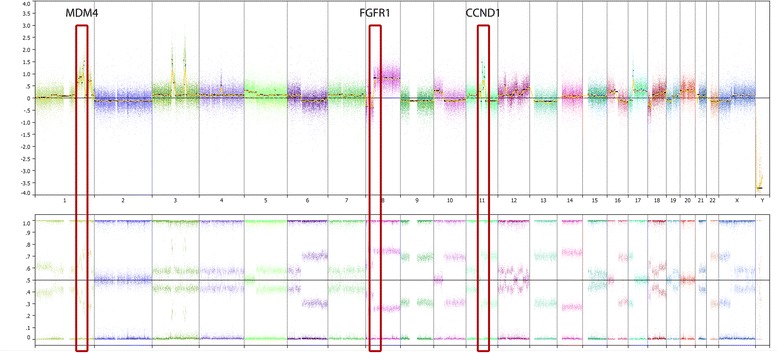
**Whole genome CN trace from breast cancer sample TSB00115.** Captured by the Nexus Express for Affymetrix software version 3. A Complex cancer genome is shown from a patient with breast cancer. Upper panel is the log2ratio plot and lower panel is the BAF plot. Multiple aberrations are observed across the genome, including high focal amplifications of the proposed predictive genes: CCND1, FGFR1 and MDM4.

Another key observation when looking at the landscape of cancer genomes is that the genomic findings are often extremely complex, with frequent co-occurrence of targetable aberrations. An example of this is observed in sample number TSB00115 that shows multiple regions of high amplification, including regions listed in the TARGET database [24]: High focal amplification of *CCND1*, *FGFR1* and *MDM4* are all observed in a complex genomic setting that also includes whole arm amplification of 8q, including *MYC* (see Figure 7). In such instances, single target treatments may not be appropriate, it is important that we can observe the entire aberration landscape to make informed decisions on not only what treatments options are available, but also how they might best be used in combination.

## Conclusions

Effective therapeutic intervention requires an understanding of the global genomic changes that drive tumour growth and the interplay between altered metabolic/signalling pathways. The low quantities of DNA available from FFPE together with the time and costs associated with employing multiple testing strategies highlight a need for multiplexing technologies that allow a broader view of the whole cancer genome from one test, ensuring that patients are diagnosed, stratified appropriately and that correct therapies are employed in clinically relevant timeframes. A single test that delivers accurate whole genome CN data, with high resolution in cancer genes and high dynamic range detection capabilities alongside a panel of frequently tested SMs is a valuable development with potential clinical utility. We have demonstrated that the OncoScan® assay provides reproducible SM, CN, LOH, and QC metrics irrespective of laboratory environment. We have shown how OncoScan® can detect all known actionable copy number alterations in a single assay with high sensitivity, as well as provide an assessment on genomic instability. Furthermore, we have shown that there is > 90% agreement of somatic mutation calls between the OncoScan® assay and sequencing technologies currently widely employed. This validation of CN, LOH and SM reproducibility supports its potential adoption as a clinical diagnostic tool.

Future work will look to further validate SM sensitivity and specificity against NGS approaches for a wider set of the SM panel covered by the OncoScan® assay. A comparison of CN data concordance with orthogonal array, FISH and NGS approaches will be useful for determining CN event boundary accuracy along with lower resolution comparisons to the current routine test for the detection of CN aberrations; FISH. In addition to platform validation, this data set has provided unique insight into genomic events in a wide range of tumour types that will form the basis of future follow up studies.

## Additional files

Additional file 1: Table S1. Shows the 162 unique samples used for analysis in this report, the laboratory from which they were sourced, the primary tumour type, the tissue of origin, any somatic mutation the sample was pre-validated for and the technology used for that pre-validation. **Table S2.** Shows the number of sample runs with ndSNPQC ≥ 26 pre-validated for each somatic mutation. For each somatic mutation the number of true positives (sample runs pre-validated for the SM that record it as a “High Confidence” Call by the OncoScan® assay), false negatives (sample runs pre-validated for the SM that record it as a “Lower Confidence” or “Undetected” Call by the OncoScan® assay) and the Sensitivity (true positives/(true positives + false negatives)). **Table S3.** Shows the concordance of the 64 Somatic Mutations (SMs) available on the OncoScan® assay. SM performance is closely related to the QC metric ndSNPQC. 162 unique samples run in triplicate were analysed and for the purpose of this analysis split into 3 groups; triplicates for which all sample runs had ndSNPQC > =35, triplicates for which all sample runs had ndSNPQC > =26 and triplicates for which all sample runs had ndSNPQC <26. *Supplementary data –*The full microarray dataset are publically available in the ArrayExpress database (www.ebi.ac.uk/arrayexpress) under accession number E-MTAB-2914. Additional file 2:
**For each sample run in triplicate (once at each of the three test laboratories) the CN/LOH profile is plotted alongside a CN agreement plot that displayed the % of the 3 samples that agree on a given CN event.**

## Abbreviations

SM: Somatic Mutation
CN: Copy Number
LOH: Loss of Heterozygosity
ND: Normal Diploid
WMRGL: West Midlands Regional Genetics Laboratory
LICAP: Leeds Institute of Cancer and Pathology
HFA: High focal amplification
SOP: Standard Operating Procedure
MAPD: Median of the Absolute Values of all Pairwise Differences is a global measure of the variation of all microarray probes across the genome. It represents the median of the distribution of changes in log2 ratio between adjacent probes. Since it measures differences between adjacent probes, it is a measure of short-range noise in the microarray data. Lower MAPD values are better, with those exceeding 0.30 being considered “out of bounds”. Further information can be found in the OncoScan® Console Manual at http://www.affymetrix.com/support/downloads/manuals/oncoscan_console_1_1_user_manual.pdf
SNPQC: Single Nucleotide Polymorphism Quality Control is a measure of how well the genotype alleles are resolved in the micro array data. ndSNPQC is the same metric as SNPQC but only applied to markers pre-classified as normal diploid (those that have been determined to have Copy Number equal to two in the sample). Larger ndSNPQC values are better, where samples with ndSNPQC less than 26 being considered “out of bounds”. Further information can be found in the OncoScan® Console Manual http://www.affymetrix.com/support/downloads/manuals/oncoscan_console_1_1_user_manual.pdf
Mutation Score: Mutation Score is a measure of the signal intensity of the marker relative to the expected signal distribution of this marker in the absence of the mutation. A higher Mutation Score for the same marker indicates greater confidence that the mutation is present, and is correlated with higher % mutant allele
Mutation Call: For each SM a pair of thresholds exists that classifies a Mutation Score into one of three Mutation Call categories: “undetected”, “low confidence” and “high confidence”. SMs with a Mutation Score less than the low confidence threshold are classified as undetected. SMs with a Mutation Score higher than the high confidence threshold are classified as high confidence. SMs with a Mutation Score between the low and high confidence threshold are classified as low confidence
Sensitivity: True positives/(true positives + false negatives)
Concordance: Max(number of observations in agreement) /number of observations
Inter-probe distance: The number of base pairs between two adjacent probes
TuScan: Analysis algorithm to determine copy number for samples processed using OncoScan® FFPE Assay Kit
Aberrant Cell Fraction: An output of the TuScan algorithm, this is the estimated percentage tumour cells in a sample. When returned as “homogeneous” this is interpreted as either 100% tumour or 100% normal depending upon the supporting CN data
