# Supplementary material for: Cross-laboratory validation of the OncoScan® FFPE Assay, a multiplex tool for whole genome tumour profiling
Source: BMC Med Genomics. 2015 Feb 18;8:5. doi: 10.1186/s12920-015-0079-z (PMC4342810; doi:10.1186/s12920-015-0079-z)
Supplement: Additional file 1: Table S1. — Shows the 162 unique samples used for analysis in this report, the laboratory from which they were sourced, the primary tumour type, the tissue of origin, any somatic mutation the sample was pre-validated for and the technology used for that pre-validation. Table S2. Shows the number of sample runs with ndSNPQC ≥ 26 pre-validated for each somatic mutation. For each somatic mutation the number of true positives (sample runs pre-validated for the SM that record it as a “High Confidence” Call by the OncoScan® assay), false negatives (sample runs pre-validated for the SM that record it as a “Lower Confidence” or “Undetected” Call by the OncoScan® assay) and the Sensitivity (true positives/(true positives + false negatives)). Table S3. Shows the concordance of the 64 Somatic Mutations (SMs) available on the OncoScan® assay. SM performance is closely related to the QC metric ndSNPQC. 162 unique samples run in triplicate were analysed and for the purpose of this analysis split into 3 groups; triplicates for which all sample runs had ndSNPQC > =35, triplicates for which all sample runs had ndSNPQC > =26 and triplicates for which all sample runs had ndSNPQC <26. Supplementary data –The full microarray dataset are publically available in the ArrayExpress database (www.ebi.ac.uk/arrayexpress) under accession number E-MTAB-2914. [file 12920_2015_79_MOESM1_ESM.pdf]

Supplementary Table 1

Sample	Source	Primary Tumour	Tissue of Origin	SM	Technology
TSB00019	LabC	Colorectal	Colon	BRAF c.1799T>A	Pyrosequencing
TSB00020	LabC	Colorectal	Colon	BRAF c.1799T>A	Pyrosequencing
TSB00021	LabC	Colorectal	Colon	BRAF c.1799T>A	Pyrosequencing
TSB00022	LabC	Colorectal	Colon	BRAF c.1799T>A	Pyrosequencing
TSB00023	LabC	Colorectal	Colon	BRAF c.1799T>A	Pyrosequencing
TSB00024	LabC	Colorectal	Colon	BRAF c.1799T>A	Pyrosequencing
TSB00025	LabC	Colorectal	Colon	BRAF c.1799T>A	Pyrosequencing
TSB00026	LabC	Colorectal	Colon	BRAF c.1799T>A	Pyrosequencing
TSB00027	LabC	Colorectal	Colon	BRAF c.1799T>A	Pyrosequencing
TSB00028	LabC	Colorectal	Colon	BRAF c.1799T>A	Pyrosequencing
TSB00029	LabC	Colorectal	Colon	BRAF c.1799T>A	Pyrosequencing
TSB00030	LabC	Colorectal	Colon	BRAF c.1799T>A	Pyrosequencing
TSB00031	LabC	Colorectal	Colon	BRAF c.1799T>A	Pyrosequencing
TSB00032	LabC	Colorectal	Colon	BRAF c.1799T>A	Pyrosequencing
TSB00033	LabC	Colorectal	Colon	BRAF c.1799T>A	Pyrosequencing
TSB00034	LabC	Colorectal	Colon	BRAF c.1799T>A	Pyrosequencing
TSB00035	LabC	Colorectal	Colon	BRAF c.1799T>A	Pyrosequencing
TSB00036	LabC	Colorectal	Colon	BRAF c.1799T>A	Pyrosequencing
TSB00037	LabC	Colorectal	Colon	BRAF c.1799T>A	Pyrosequencing
TSB00038	LabC	Colorectal	Colon	BRAF c.1799T>A	Pyrosequencing
TSB00039	LabC	Colorectal	Colon	BRAF c.1799T>A	Pyrosequencing
TSB00040	LabC	Colorectal	Colon	BRAF c.1799T>A	Pyrosequencing
TSB00041	LabC	Colorectal	Colon	KRAS c.35G>T	Pyrosequencing
TSB00042	LabC	Colorectal	Colon	KRAS c.35G>T	Pyrosequencing
TSB00043	LabC	Colorectal	Colon	KRAS c.35G>T	Pyrosequencing
TSB00044	LabC	Colorectal	Colon	KRAS c.35G>T	Pyrosequencing
TSB00045	LabC	Colorectal	Colon	KRAS c.35G>T	Pyrosequencing
TSB00046	LabC	Colorectal	Colon	KRAS c.35G>T	Pyrosequencing
TSB00047	LabC	Colorectal	Colon	KRAS c.35G>T	Pyrosequencing
TSB00048	LabC	Colorectal	Colon	KRAS c.35G>T	Pyrosequencing
TSB00049	LabC	Colorectal	Colon	KRAS c.35G>T	Pyrosequencing
TSB00050	LabC	Colorectal	Colon	KRAS c.35G>T	Pyrosequencing
TSB00051	LabC	Colorectal	Colon	KRAS c.35G>T	Pyrosequencing
TSB00052	LabC	Colorectal	Colon	KRAS c.35G>T	Pyrosequencing
TSB00053	LabC	Colorectal	Colon	KRAS c.35G>T	Pyrosequencing
TSB00054	LabC	Colorectal	Colon	KRAS c.35G>T	Pyrosequencing
TSB00055	LabC	Colorectal	Colon	KRAS c.35G>T	Pyrosequencing
TSB00056	LabC	Colorectal	Colon	KRAS c.35G>T	Pyrosequencing
TSB00057	LabC	Colorectal	Colon	KRAS c.35G>T	Pyrosequencing
TSB00058	LabC	Colorectal	Colon	KRAS c.35G>T	Pyrosequencing
TSB00059	LabC	Colorectal	Colon	KRAS c.35G>T	Pyrosequencing
TSB00060	LabC	Colorectal	Colon	KRAS c.35G>T	Pyrosequencing

TSB00061	LabC	Colorectal	Colon	KRAS c.35G>T	Pyrosequencing
TSB00062	LabC	Colorectal	Colon	KRAS c.35G>T	Pyrosequencing
TSB00063	LabC	Colorectal	Colon	NA	Pyrosequencing
TSB00064	LabC	Colorectal	Colon	NA	Pyrosequencing
TSB00065	LabC	Colorectal	Colon	NA	Pyrosequencing
TSB00066	LabC	Colorectal	Colon	NA	Pyrosequencing
TSB00067	LabC	Colorectal	Colon	NA	Pyrosequencing
TSB00068	LabC	Colorectal	Colon	NA	Pyrosequencing
TSB00069	LabC	Colorectal	Colon	NA	Pyrosequencing
TSB00070	LabC	Colorectal	Colon	NA	Pyrosequencing
TSB00071	LabC	Colorectal	Colon	NA	Pyrosequencing
TSB00072	LabC	Colorectal	Colon	NA	Pyrosequencing
TSB00073	LabC	Colorectal	Colon	NA	Pyrosequencing
TSB00074	LabC	Colorectal	Colon	NA	Pyrosequencing
TSB00076	LabB	Colorectal	Liver	KRAS c.35G>T,TP53 c.818G>A	Pyrosequencing/Sanger
TSB00077	LabB	Colorectal	Liver	KRAS c.35G>T	Pyrosequencing
TSB00078	LabB	Colorectal	Colon	TP53 c.743G>A	Sanger
TSB00079	LabB	Melanoma	Skin	BRAF c.1798_1799delGTinsAA	Sanger
TSB00080	LabB	Melanoma	Skin	BRAF c.1799T>A	Sanger
TSB00081	LabB	Melanoma	Skin	NRAS c.182A>G	Pyrosequencing
TSB00082	LabB	Colorectal	Colon	BRAF c.1799T>A	Sanger
TSB00083	LabB	Colorectal	Liver	NRAS c.35G>T	Pyrosequencing
TSB00084	LabB	Colorectal	Liver	TP53 c.743G>A	Sanger
TSB00085	LabB	Colorectal	Liver	KRAS c.34G>A	Pyrosequencing
TSB00086	LabB	Colorectal	Liver	KRAS c.35G>A	Pyrosequencing
TSB00087	LabB	Colorectal	Colon	KRAS c.35G>T	Pyrosequencing
TSB00088	LabB	Colorectal	Colon	BRAF c.1799T>A	Sanger
TSB00089	LabB	Melanoma	Skin	BRAF c.1799T>A	Sanger
TSB00090	LabB	Colorectal	Colon	KRAS c.35G>T	Pyrosequencing
TSB00091	LabB	Colorectal	Liver	BRAF c.1799T>A	Sanger
TSB00092	LabB	Colorectal	Colon	KRAS c.38G>A	Pyrosequencing
TSB00093	LabB	Breast	Breast	PIK3CA c.3140A>G	RT-QPCR
TSB00094	LabB	Breast	Breast	PIK3CA c.3140A>G	RT-QPCR
TSB00095	LabB	Breast	Breast	TP53 c.637C>T	Sanger
TSB00096	LabB	Breast	Breast	NA	NA
TSB00097	LabB	Melanoma	Skin	BRAF c.1799T>A	Sanger
TSB00098	LabB	Melanoma	Skin	NRAS c.182A>G	Pyrosequencing
TSB00099	LabB	Melanoma	Skin	NRAS c.182A>G	Pyrosequencing
TSB00100	LabB	Colorectal	Colon	KRAS c.35G>A,PIK3CA c.1624G>A	Pyrosequencing/RT-QPCR
TSB00101	LabB	Colorectal	Colon	KRAS c.35G>A	Pyrosequencing
TSB00102	LabB	Colorectal	Liver	KRAS c.35G>T	Pyrosequencing
TSB00103	LabB	Melanoma	Skin	BRAF c.1799T>A	Sanger
TSB00104	LabB	Melanoma	Skin	NRAS c.181C>A	Pyrosequencing
TSB00105	LabB	Melanoma	Skin	NRAS c.182A>G	Pyrosequencing

TSB00106	LabB	Colorectal	Colon	TP53 c.916C>T	Sanger
TSB00107	LabB	Colorectal	Colon	KRAS c.35G>C,TP53 c.733G>A	Pyrosequencing/Sanger
TSB00108	LabB	Colorectal	Colon	PIK3CA c.1633G>A	RT-QPCR
TSB00109	LabB	Colorectal	Colon	KRAS c.35G>A,PIK3CA c.1624G>A	Pyrosequencing/RT-QPCR
TSB00110	LabB	Colorectal	Colon	TP53 c.637C>T	Sanger
TSB00111	LabB	Colorectal	Colon	KRAS c.35G>A	Pyrosequencing
TSB00112	LabB	Colorectal	Colon	KRAS c.35G>A	Pyrosequencing
TSB00113	LabB	Colorectal	Liver	TP53 c.524G>A	Sanger
TSB00114	LabB	Breast	Breast	NA	NA
TSB00115	LabB	Breast	Breast	NA	NA
TSB00116	LabB	Breast	Breast	PIK3CA c.3140A>T	RT-QPCR
TSB00117	LabB	Breast	Breast	TP53 c.743G>A	Sanger
TSB00118	LabB	Breast	Breast	PIK3CA c.1624G>A	RT-QPCR
TSB00119	LabB	Breast	Breast	NA	NA
TSB00142	LabB	Colorectal	Colon	NA	NA
TSB00143	LabB	Colorectal	Liver	NA	NA
TSB00144	LabB	Colorectal	Liver	NA	NA
TSB00145	LabB	Colorectal	Colon	NA	NA
TSB00146	LabB	Prostate	Prostate	NA	NA
TSB00147	LabB	Breast	Breast	NA	NA
TSB00148	LabB	Colorectal	Liver	NA	NA
TSB00149	LabB	Colorectal	Liver	NA	NA
TSB00150	LabB	Colorectal	Liver	NA	NA
TSB00151	LabB	Colorectal	Liver	NA	NA
TSB00152	LabB	Colorectal	Colon	NA	NA
TSB00153	LabB	Colorectal	Colon	NA	NA
TSB00154	LabB	Other	Lymph Node	NA	NA
TSB00155	LabB	Melanoma	Liver Tumour	NA	NA
TSB00156	LabB	Ovarian	Ovary	NA	NA
TSB00157	LabB	Breast	Breast	NA	NA
TSB00158	LabB	Breast	Breast	NA	NA
TSB00159	LabB	Colorectal	Colon	NA	NA
TSB00160	LabB	Colorectal	Liver	NA	NA
TSB00161	LabB	Breast	Breast	NA	NA
TSB00162	LabB	Colorectal	Colon	NA	NA
TSB00164	LabB	Colorectal	Colon	KRAS c.35G>A	Pyrosequencing
TSB00167	LabB	Breast	Breast	PIK3CA c.1633G>A	RT-QPCR
TSB00168	LabB	Breast	Breast	NA	NA
TSB00169	LabB	Breast	Breast	PIK3CA c.3140A>G	RT-QPCR
TSB00170	LabB	Breast	Breast	PIK3CA c.1633G>A	RT-QPCR
TSB00171	LabB	Breast	Breast	PIK3CA c.1633G>A	RT-QPCR
TSB00172	LabB	Colorectal	Colon	TP53 c.488A>G	Sanger
TSB00173	LabB	Breast	Breast	TP53 c.637C>T	Sanger
TSB00175	LabB	Colorectal	Liver	NA	NA

TSB00176	LabB	Colorectal	Liver	TP53 c.398T>A	Sanger
TSB00177	LabB	Colorectal	Lymph node	NA	NA
TSB00178	LabB	Colorectal	Colon	NA	NA
TSB00179	LabB	Colorectal	Colon	NA	NA
TSB00180	LabB	Colorectal	Colon	NA	NA
TSB00181	LabB	Colorectal	Liver	NA	NA
TSB00182	LabB	Melanoma	Lymph node	NA	NA
TSB00183	LabB	Normal tissue	Tonsil	NA	NA
TSB00184	LabB	Colorectal	Liver	NA	NA
TSB00186	LabB	Prostate	Prostate	NA	NA
TSB00188	LabB	Prostate	Prostate	NA	NA
TSB00189	LabB	Prostate	Prostate	NA	NA
TSB00190	LabB	Prostate	Prostate	NA	NA
TSB00191	LabB	Prostate	Prostate	NA	NA
TSB00192	LabB	Ovarian	Ovary	NA	NA
TSB00193	LabB	Ovarian	Ovary	NA	NA
TSB00197	LabB	Ovarian	Ovary	NA	NA
TSB00198	LabB	Lung	Lung	NA	NA
TSB00199	LabB	Ovarian	Ovary	NA	NA
TSB00200	LabB	Ovarian	Ovary	NA	NA
TSB00201	LabB	Ovarian	Ovary	NA	NA
TSB00202	LabB	Lung	Lung	NA	NA
TSB00203	LabB	Lung	Lung	NA	NA
TSB00204	LabB	Lung	Lung	NA	NA
TSB00206	LabB	Lung	Lung	NA	NA
TSB00207	LabB	Lung	Lung	NA	NA
TSB00208	LabB	Lung	Lung	NA	NA
TSB00210	LabB	Lung	Lung	NA	NA
TSB00211	LabB	Lung	Lung	NA	NA
TSB00213	LabB	Lung	Lung	NA	NA
TSB00220	LabB	Lung	Lung	NA	NA
TSB00222	LabB	Lung	Lung	NA	NA

Supplementary table 1 shows the 162 unique samples used for analysis in this report, the site from which they were sourced, the primary tumour type, the tissue of origin, any somatic mutation the sample was pre-validated for and the technology used for that pre-validation.

Supplementary Table 2

SM	Sample-Runs	True-Positives	False-Negatives	Sensitivity
BRAF:p.V600K:c.1798_1799GT>AA	3	3	0	100
BRAF:p.V600E:c.1799T>A	73	73	0	100
KRAS:p.G12A:c.35G>C	2	2	0	100
KRAS:p.G12D/V:c.35G>A/T	91	78	13	85.7
KRAS:p.G12C/S:c.34G>T/A	3	2	1	66.7
KRAS:p.G13D:c.38G>A	3	3	0	100
NRAS:p.G12V:c.35G>T	3	3	0	100
PIK3CA:p.E545K:c.1633G>A	6	4	2	66.7
PIK3CA:p.H1047R:c.3140A>G	9	7	2	77.8
TP53:p.G245S/C:c.733G>A/T	2	2	0	100
NRAS:p.Q61K:c.181C>A	1	1	0	100
PIK3CA:p.E542K:c.1624G>A	7	6	1	85.7
PIK3CA:p.H1047L:c.3140A>T	3	3	0	100
NRAS:p.Q61R:c.182A>G	9	9	0	100
TP53:p.R248Q/L:c.743G>A/T	8	8	0	100
TP53:p.R213*:c.637C>T	8	8	0	100
TP53:p.R273H/L:c.818G>A/T	1	1	0	100
TP53:p.R306*:c.916C>T	3	0	3	0

Supplementary table 2 shows the number of sample-runs with ndSNPQC >=26 pre-validated for each somatic mutation. For each somatic mutation the number of true positives (sample-runs pre-validated for the SM that record it as a “High Confidence” Call on the OncoScan platform), false negatives (sample-runs pre-validated for the SM that record it as a “Lower Confidence” or “Undetected” Call on the OncoScan platform) and the Sensitivity (true positives/(true positives + false negatives)).

Supplementary Table 3

SM	<26	>=26	>=35
BRAF:p.G469A:c.1406G>C	0.995885	0.99697	0.993197
BRAF:p.G469E:c.1406G>A	0.899177	0.909091	0.938776
BRAF:p.V600E:c.1799T>A	0.907407	0.915152	0.938776
BRAF:p.V600K:c.1798_1799GT>AA	0.987654	0.993939	1
EGFR:p.D770_N771insSVD:c.2311_2312ins9	0.975309	0.972727	0.993197
EGFR:p.E746_A750del:c.2235_2249del15	0.995885	0.993939	1
EGFR:p.E746_A750del:c.2236_2250del15	0.989712	0.990909	0.979592
EGFR:p.E746_T751>A:c.2237_2251del15	0.99177	0.99697	1
EGFR:p.G719A:c.2156G>C	0.960905	0.969697	1
EGFR:p.G719C:c.2155G>T	0.979424	0.984848	1
EGFR:p.G719S:c.2155G>A	0.860082	0.9	0.952381
EGFR:p.H773_V774insNPH:c.2319_2320ins9	0.820988	0.839394	0.918367
EGFR:p.L747_E749P/del:c.2239_2248>C/G	0.769547	0.745455	0.85034
EGFR:p.L747_P753>S:c.2240_2257del18	0.995885	0.993939	1
EGFR:p.L747_T751del:c.2240_2254del15	1	1	1
EGFR:p.L858R:c.2573T>G	0.806584	0.8	0.816327
EGFR:p.L861Q:c.2582T>A	1	1	1
EGFR:p.T790M:c.2369C>T	0.858025	0.893939	0.965986
EGFR:p.V769_D770insASV:c.2307_2308ins9	0.853909	0.884848	0.952381
IDH1:p.R132H:c.395G>A	0.993827	1	1
IDH2:p.R140Q:c.419G>A	0.917695	0.948485	0.979592
IDH2:p.R172K:c.515G>A	0.975309	0.993939	1
KRAS:p.A146P:c.436G>C	0.985597	0.987879	0.993197
KRAS:p.G12A:c.35G>C	0.820988	0.839394	0.931973
KRAS:p.G12C/S:c.34G>T/A	0.843621	0.909091	0.979592
KRAS:p.G12D/V:c.35G>A/T	0.808642	0.787879	0.85034
KRAS:p.G13D:c.38G>A	0.868313	0.939394	0.979592
KRAS:p.Q61H:c.183A>C	0.763374	0.830303	0.911565
KRAS:p.Q61H:c.183A>T	0.985597	0.987879	1
KRAS:p.Q61K/K:c.180_181TC>TA/AA	0.997942	0.99697	0.993197
NRAS:p.G12D:c.35G>A	0.847737	0.872727	0.952381
NRAS:p.G12S/C:c.34G>A/T	0.825103	0.818182	0.843537
NRAS:p.G12V:c.35G>T	0.829218	0.869697	0.965986
NRAS:p.Q61L:c.182A>T	0.960905	0.978788	1
PIK3CA:p.E545K:c.1633G>A	0.86214	0.89697	0.945578
PIK3CA:p.H1047R:c.3140A>G	0.938272	0.972727	0.993197
PTEN:p.K267fs*9:c.800delA	0.839506	0.878788	0.92517
PTEN:p.R130*:c.388C>T	0.942387	0.966667	0.972789
PTEN:p.R130Q/fs*4:c.389G>A/delG	0.997942	1	1
PTEN:p.R233*:c.697C>T	0.843621	0.90303	0.952381
TP53:p.G245S/C:c.733G>A/T	0.790123	0.824242	0.911565
TP53:p.R175H:c.524G>A	0.901235	0.887879	0.92517

Supplementary Table 3 shows the concordance of the 64 Somatic Mutations (SMs) available on the OncoScan assay. SM performance is closely related to the QC metric ndSNPQC. 162 unique samples run in triplicate were analysed and fro the purpose of this analysis split into 3 groups; triplicates for which all sample runs had ndSNPQC >=35, triplicates for which all sample runs had ndSNPQC >=26 and triplicates for which all sample runs had ndSNPQC <26.
