# Supplementary material for: Cross-laboratory validation of the OncoScan® FFPE Assay, a multiplex tool for whole genome tumour profiling
Source: BMC Med Genomics. 2015 Feb 18;8:5. doi: 10.1186/s12920-015-0079-z (PMC4342810; doi:10.1186/s12920-015-0079-z)

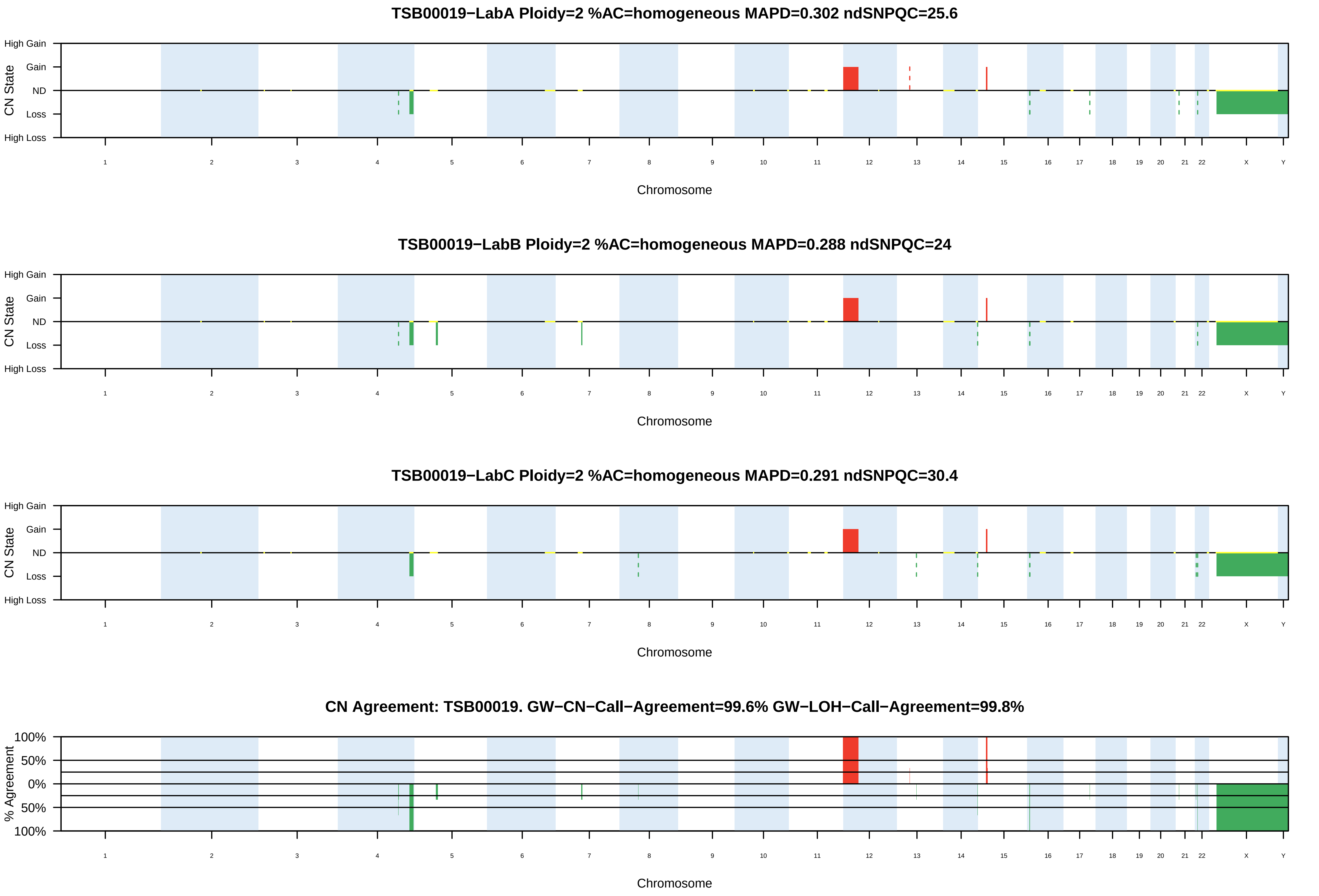
TSB00019−LabA Ploidy=2 %AC=homogeneous MAPD=0.302 ndSNPQC=25.6
TSB00019−LabB Ploidy=2 %AC=homogeneous MAPD=0.288 ndSNPQC=24
TSB00019−LabC Ploidy=2 %AC=homogeneous MAPD=0.291 ndSNPQC=30.4
CN Agreement: TSB00019. GW−CN−Call−Agreement=99.6% GW−LOH−Call−Agreement=99.8%
CN State
High Gain
Gain
ND
Loss
High Loss
% Agreement
100%
50%
0%
50%
100%
Chromosome
1 2 3 4 5 6 7 8 9 10 11 12 13 14 15 16 17 18 19 20 21 22 X Y

TSB00020−LabA Ploidy=2 %AC=45 MAPD=0.272 ndSNPQC=31.7

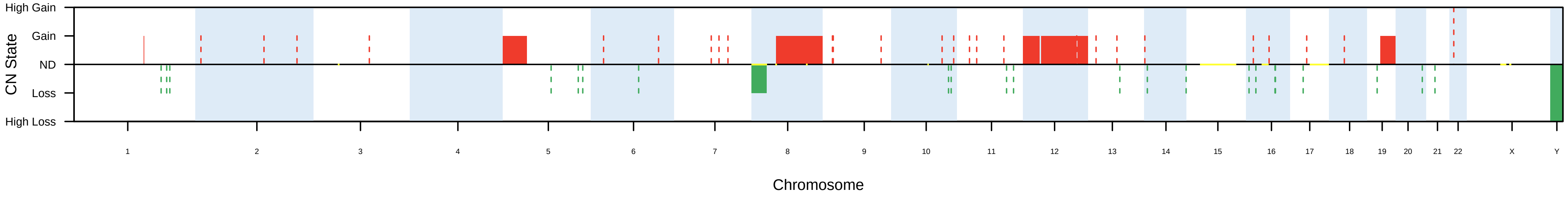

TSB00020−LabB Ploidy=2 %AC=45 MAPD=0.255 ndSNPQC=28.5

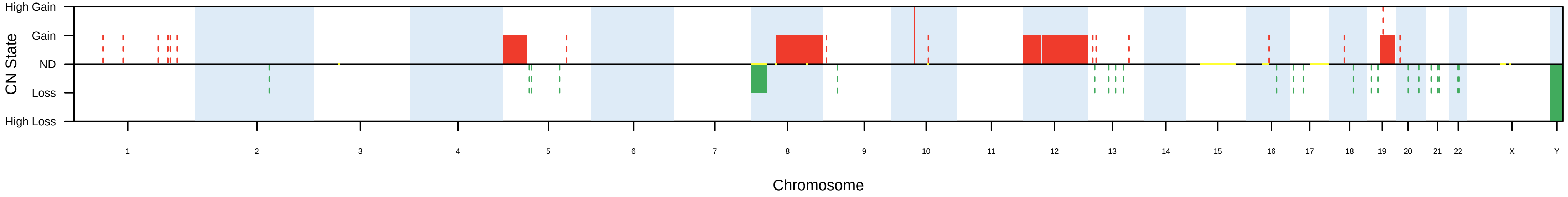

TSB00020−LabC Ploidy=2 %AC=45 MAPD=0.258 ndSNPQC=34.8

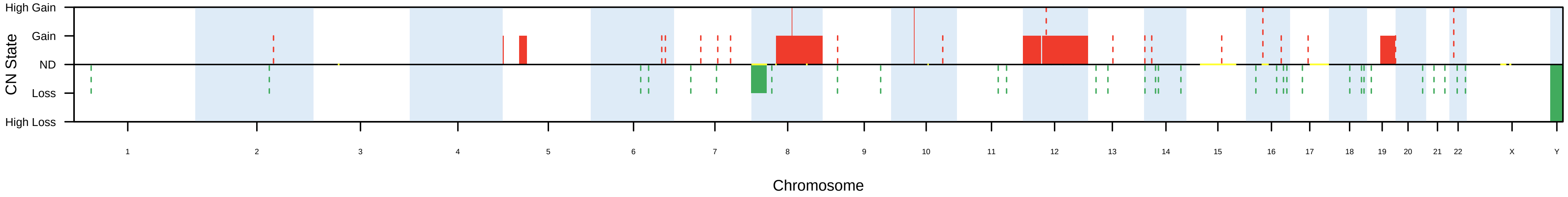

CN Agreement: TSB00020. GW−CN−Call−Agreement=97.3% GW−LOH−Call−Agreement=99.9%

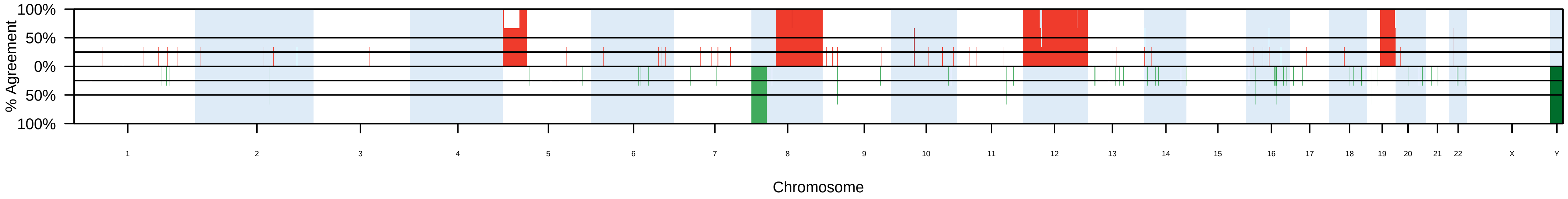

TSB00021−LabA Ploidy=2 %AC=40 MAPD=0.248 ndSNPQC=41.7

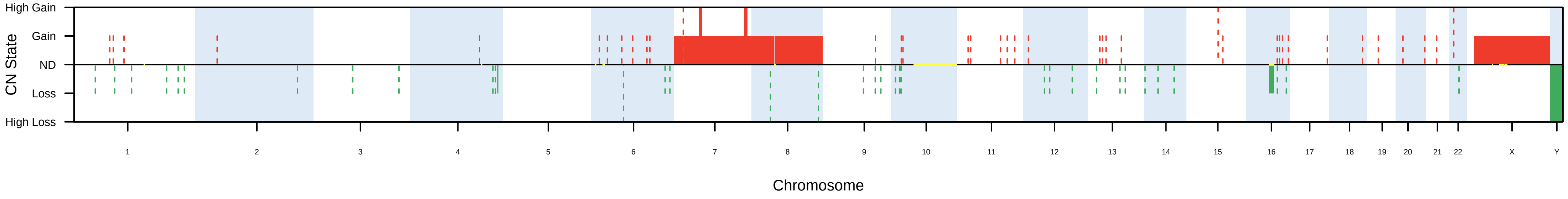

TSB00021−LabB Ploidy=2 %AC=40 MAPD=0.222 ndSNPQC=32.9

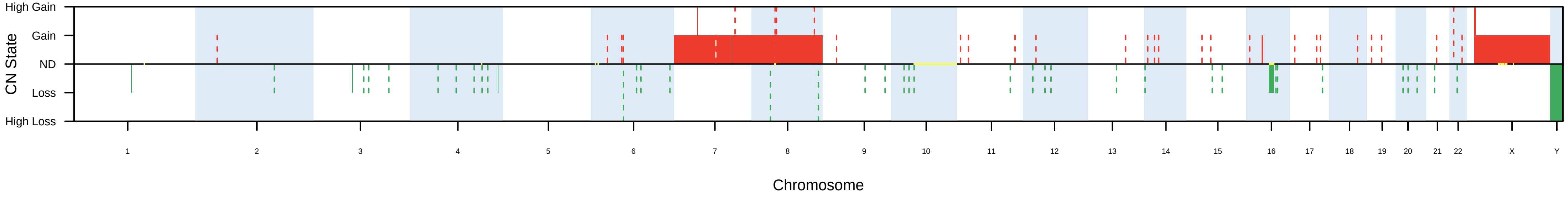

TSB00021−LabC Ploidy=2 %AC=40 MAPD=0.225 ndSNPQC=47

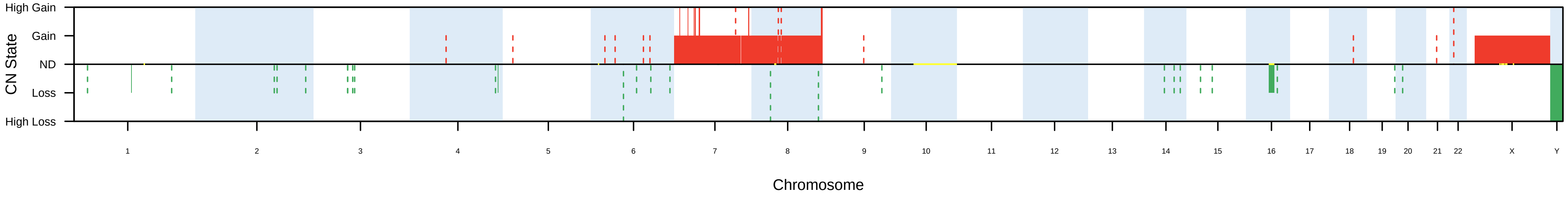

CN Agreement: TSB00021. GW−CN−Call−Agreement=97% GW−LOH−Call−Agreement=99.3%

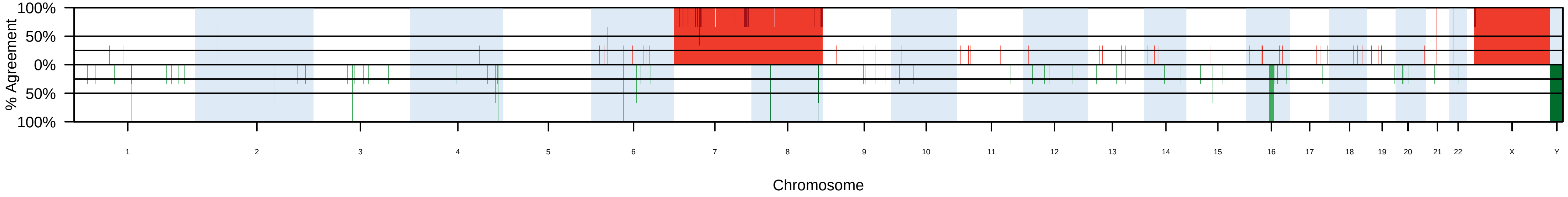

TSB00022−LabA Ploidy=2 %AC=70 MAPD=0.225 ndSNPQC=44.8

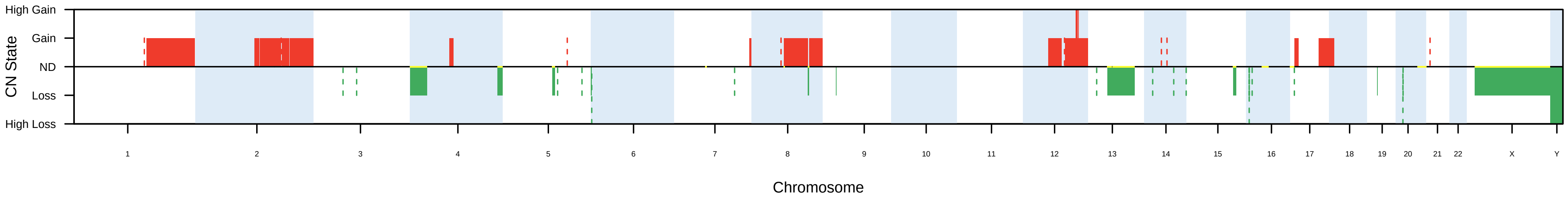

TSB00022−LabB Ploidy=2 %AC=70 MAPD=0.203 ndSNPQC=37.2

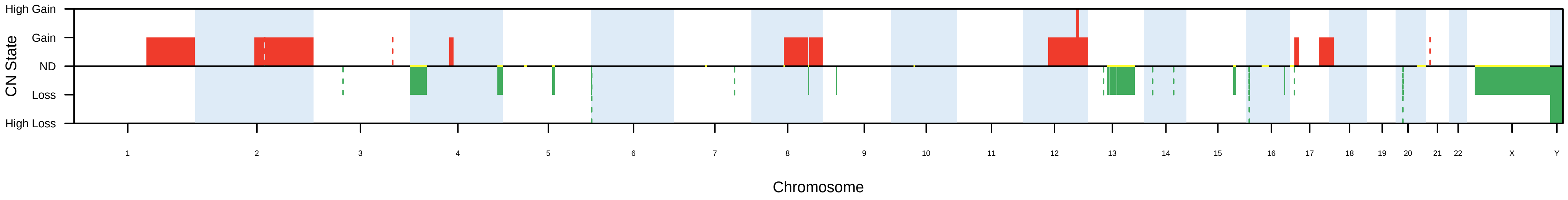

TSB00022−LabC Ploidy=2 %AC=70 MAPD=0.208 ndSNPQC=49.9

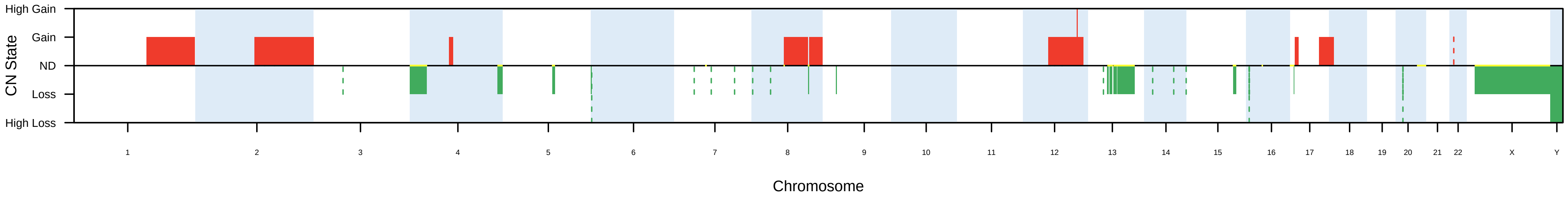

CN Agreement: TSB00022. GW−CN−Call−Agreement=98.3% GW−LOH−Call−Agreement=99.2%

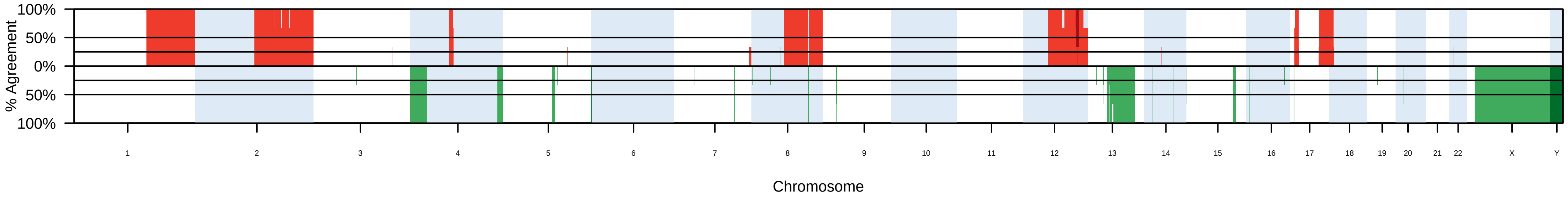

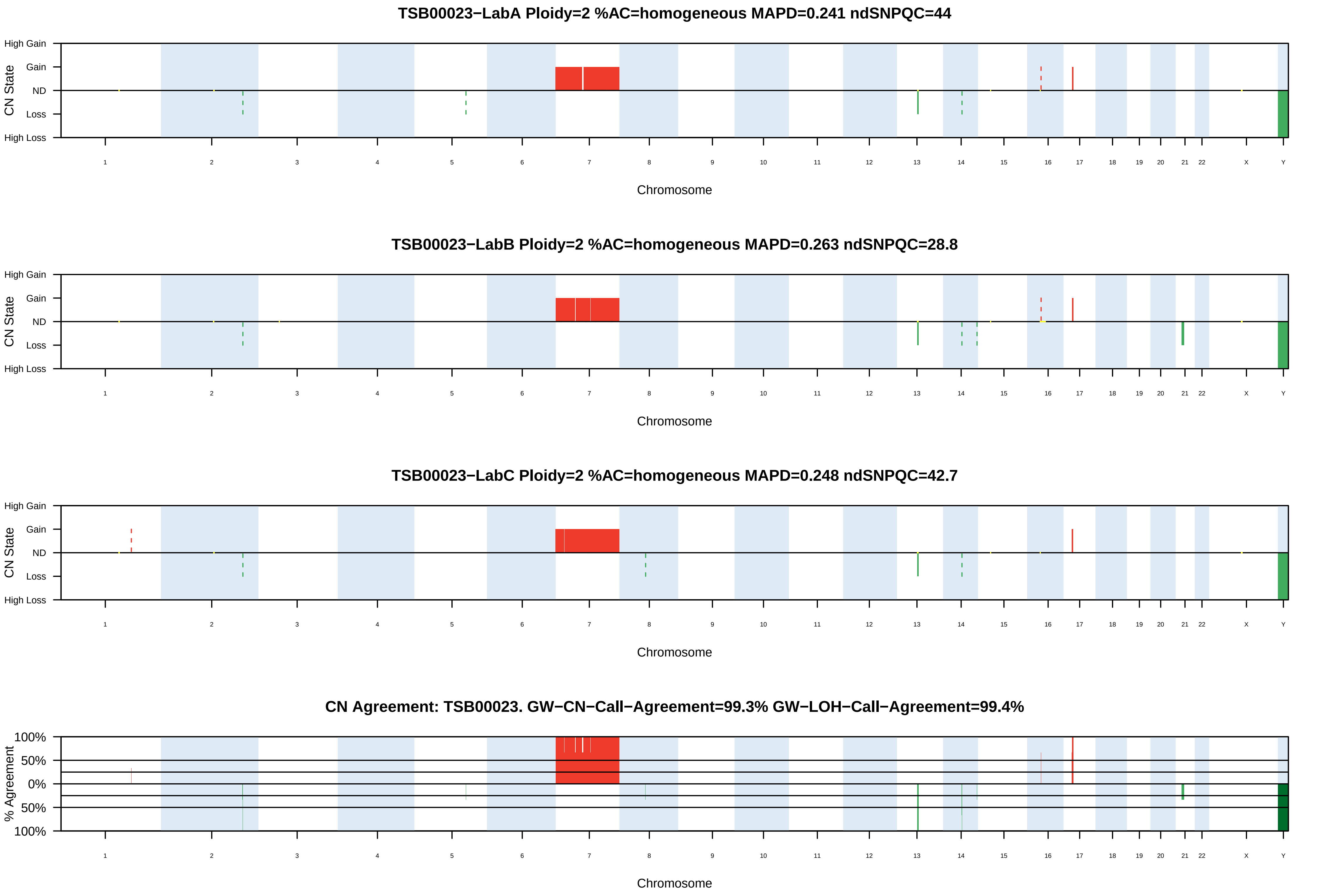

TSB00023−LabA Ploidy=2 %AC=homogeneous MAPD=0.241 ndSNPQC=44
High Gain
Gain
ND
Loss
High Loss
CN State
1 2 3 4 5 6 7 8 9 10 11 12 13 14 15 16 17 18 19 20 21 22 X Y
Chromosome
TSB00023−LabB Ploidy=2 %AC=homogeneous MAPD=0.263 ndSNPQC=28.8
High Gain
Gain
ND
Loss
High Loss
CN State
1 2 3 4 5 6 7 8 9 10 11 12 13 14 15 16 17 18 19 20 21 22 X Y
Chromosome
TSB00023−LabC Ploidy=2 %AC=homogeneous MAPD=0.248 ndSNPQC=42.7
High Gain
Gain
ND
Loss
High Loss
CN State
1 2 3 4 5 6 7 8 9 10 11 12 13 14 15 16 17 18 19 20 21 22 X Y
Chromosome
CN Agreement: TSB00023. GW−CN−Call−Agreement=99.3% GW−LOH−Call−Agreement=99.4%
100%
50%
0%
50%
100%
% Agreement
1 2 3 4 5 6 7 8 9 10 11 12 13 14 15 16 17 18 19 20 21 22 X Y
Chromosome

TSB00024−LabA Ploidy=NA %AC=NA MAPD=0.23 ndSNPQC=38.4

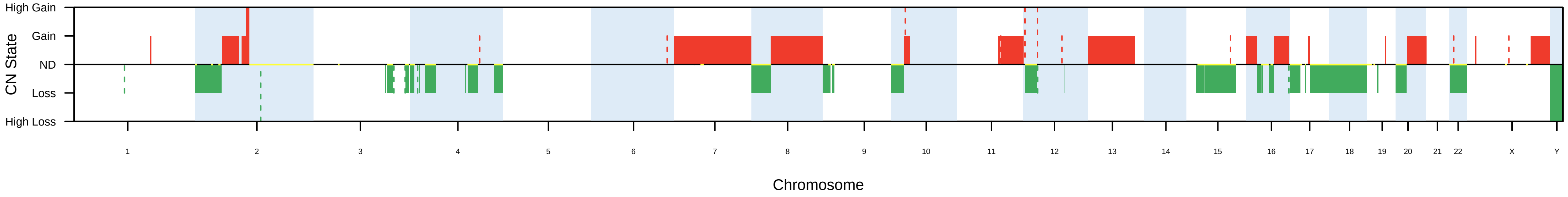

TSB00024−LabB Ploidy=2 %AC=50 MAPD=0.255 ndSNPQC=26.7

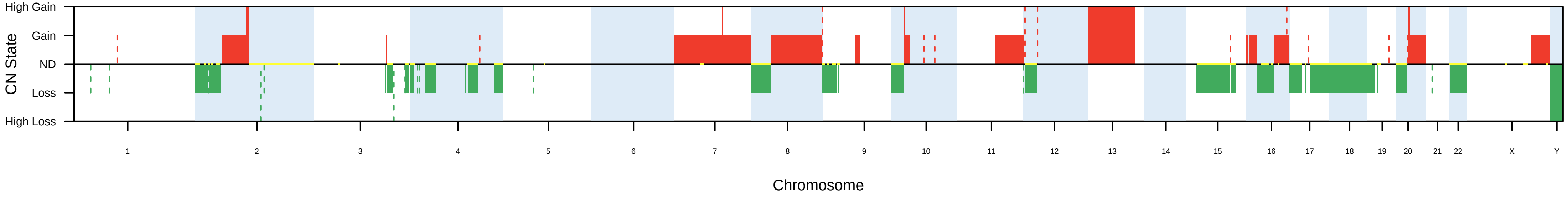

TSB00024−LabC Ploidy=NA %AC=NA MAPD=0.244 ndSNPQC=39.5

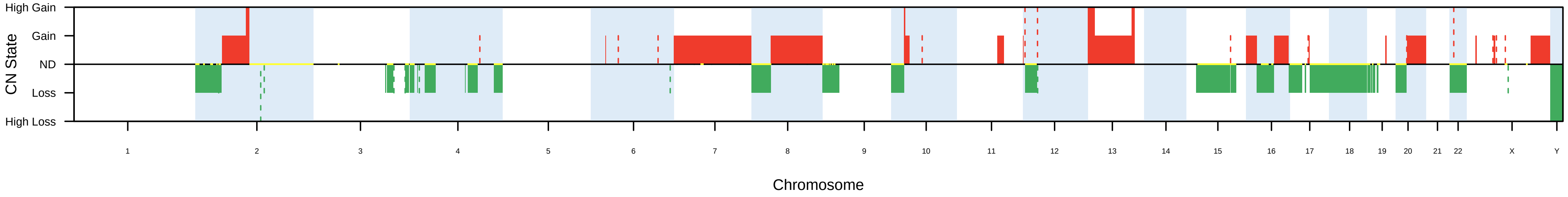

CN Agreement: TSB00024. GW−CN−Call−Agreement=90.9% GW−LOH−Call−Agreement=97.2%

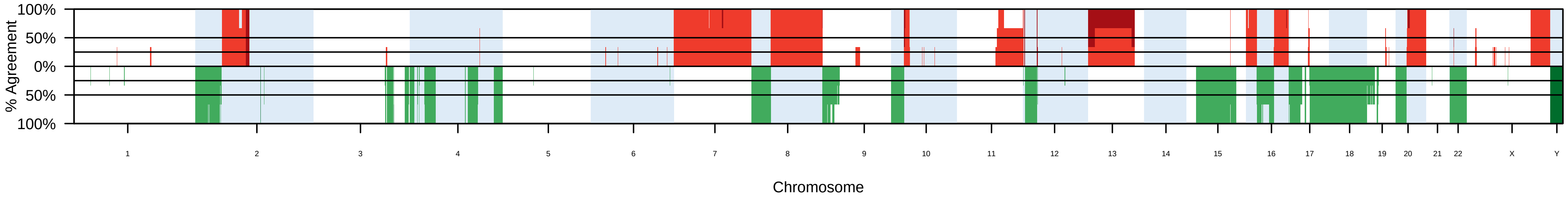

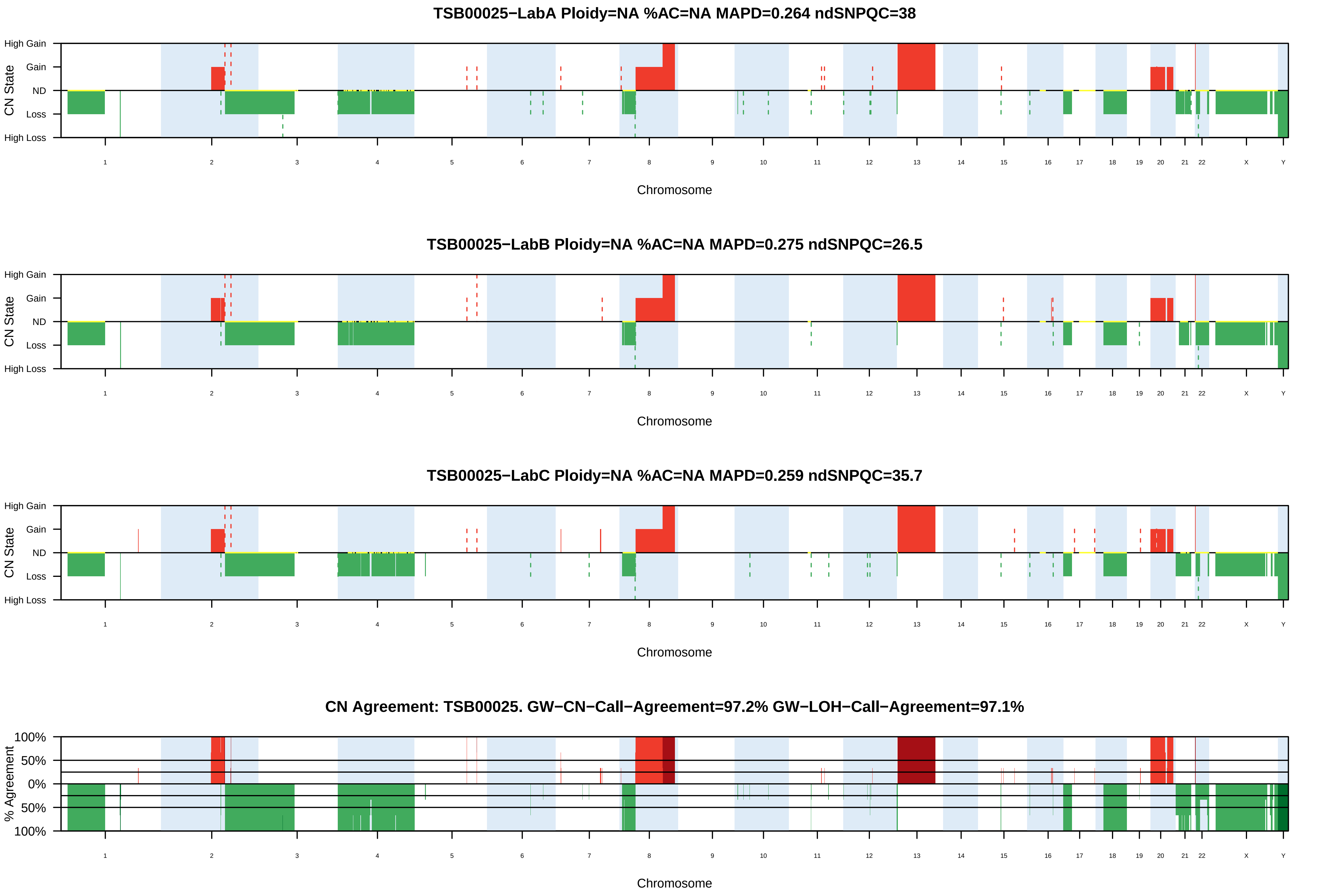
TSB00025−LabA Ploidy=NA %AC=NA MAPD=0.264 ndSNPQC=38
TSB00025−LabB Ploidy=NA %AC=NA MAPD=0.275 ndSNPQC=26.5
TSB00025−LabC Ploidy=NA %AC=NA MAPD=0.259 ndSNPQC=35.7
CN Agreement: TSB00025. GW−CN−Call−Agreement=97.2% GW−LOH−Call−Agreement=97.1%
CN State
High Gain
Gain
ND
Loss
High Loss
% Agreement
100%
50%
0%
Chromosome
1 2 3 4 5 6 7 8 9 10 11 12 13 14 15 16 17 18 19 20 21 22 X Y

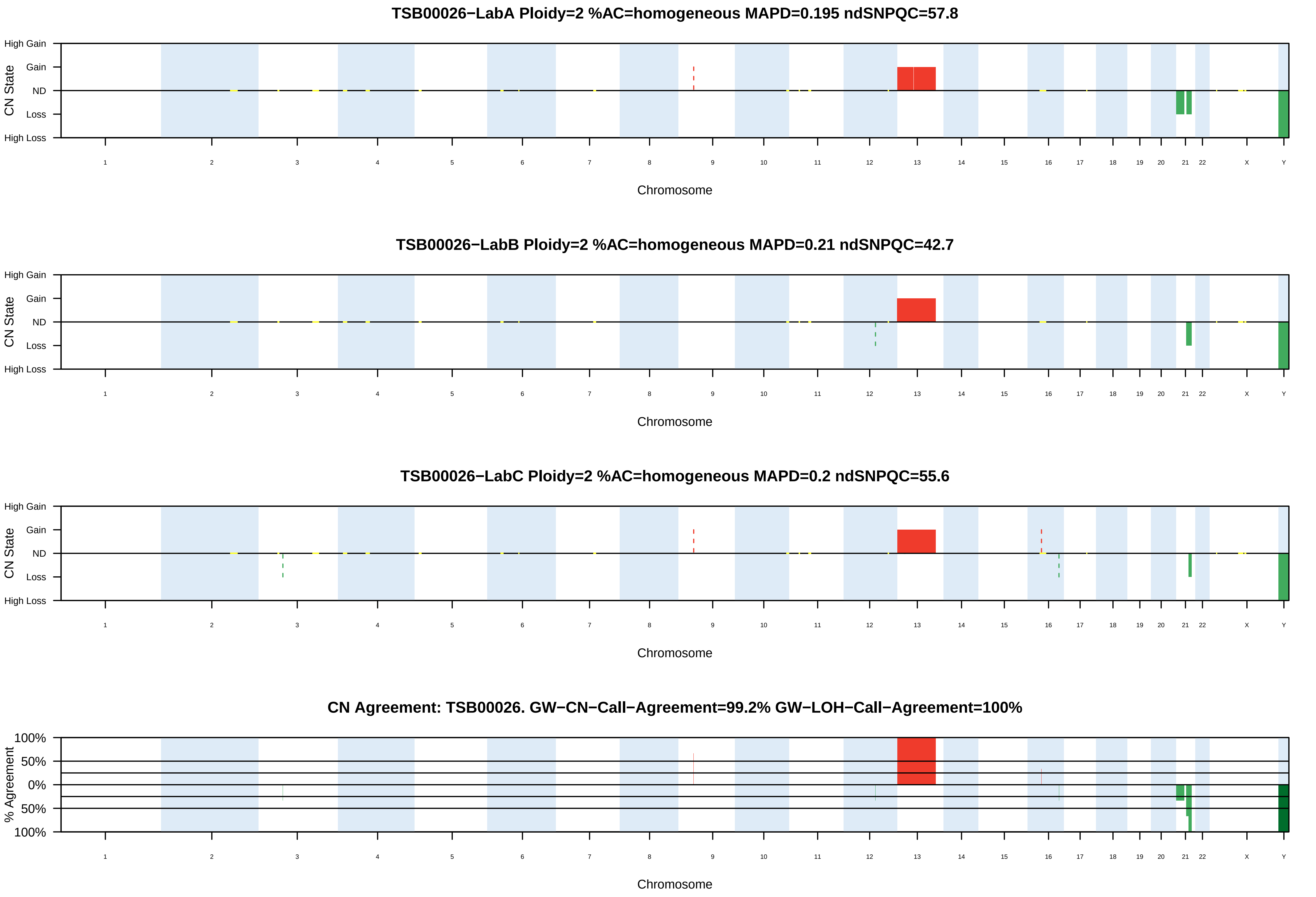
TSB00026−LabA Ploidy=2 %AC=homogeneous MAPD=0.195 ndSNPQC=57.8
CN State
High Gain
Gain
ND
Loss
High Loss
1 2 3 4 5 6 7 8 9 10 11 12 13 14 15 16 17 18 19 20 21 22 X Y
Chromosome
TSB00026−LabB Ploidy=2 %AC=homogeneous MAPD=0.21 ndSNPQC=42.7
CN State
High Gain
Gain
ND
Loss
High Loss
1 2 3 4 5 6 7 8 9 10 11 12 13 14 15 16 17 18 19 20 21 22 X Y
Chromosome
TSB00026−LabC Ploidy=2 %AC=homogeneous MAPD=0.2 ndSNPQC=55.6
CN State
High Gain
Gain
ND
Loss
High Loss
1 2 3 4 5 6 7 8 9 10 11 12 13 14 15 16 17 18 19 20 21 22 X Y
Chromosome
CN Agreement: TSB00026. GW−CN−Call−Agreement=99.2% GW−LOH−Call−Agreement=100%
% Agreement
100%
50%
0%
50%
100%
1 2 3 4 5 6 7 8 9 10 11 12 13 14 15 16 17 18 19 20 21 22 X Y
Chromosome

TSB00027−LabA Ploidy=2 %AC=50 MAPD=0.237 ndSNPQC=57

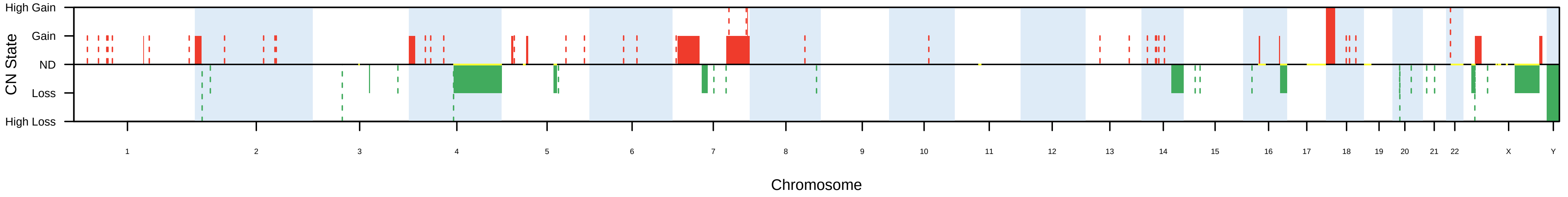

TSB00027−LabB Ploidy=2 %AC=45 MAPD=0.215 ndSNPQC=38.3

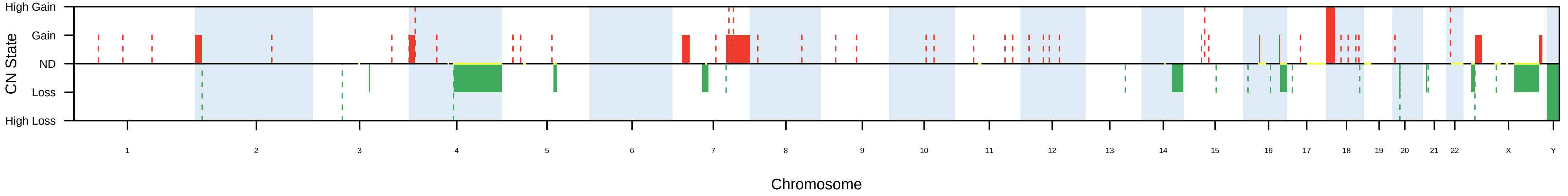

TSB00027−LabC Ploidy=2 %AC=45 MAPD=0.234 ndSNPQC=52.4

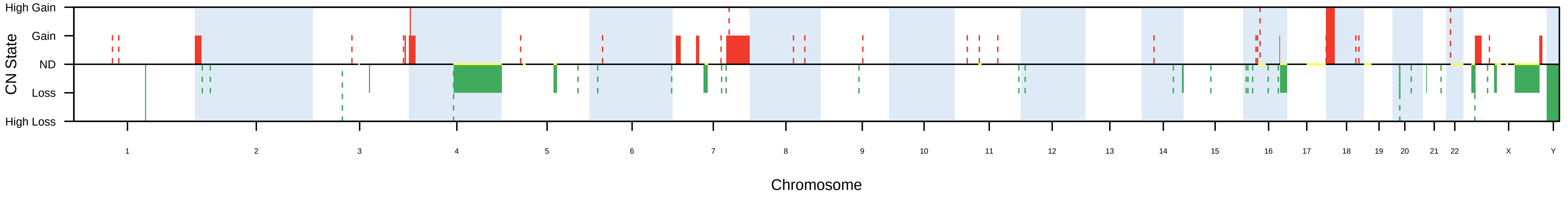

CN Agreement: TSB00027. GW−CN−Call−Agreement=95.3% GW−LOH−Call−Agreement=99.5%

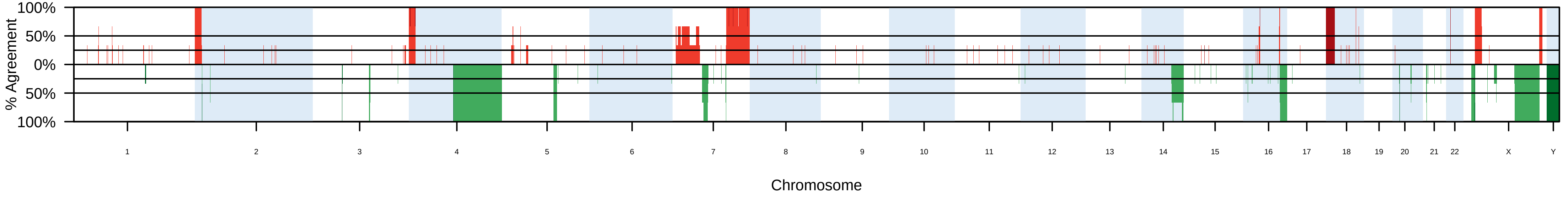

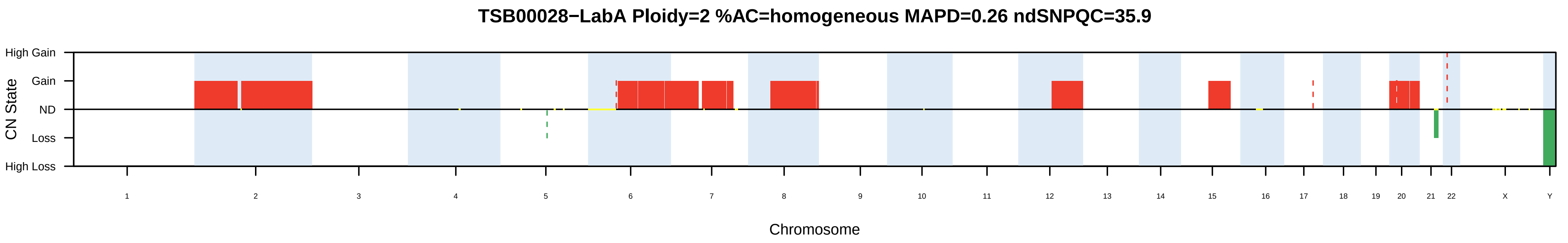
TSB00028−LabA Ploidy=2 %AC=homogeneous MAPD=0.26 ndSNPQC=35.9
High Gain
Gain
ND
Loss
High Loss
CN State
1 2 3 4 5 6 7 8 9 10 11 12 13 14 15 16 17 18 19 20 21 22 X Y
Chromosome

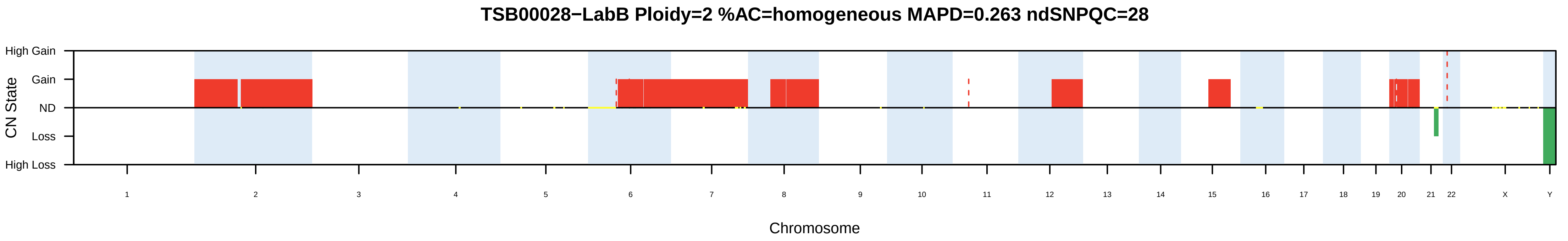
TSB00028−LabB Ploidy=2 %AC=homogeneous MAPD=0.263 ndSNPQC=28
High Gain
Gain
ND
Loss
High Loss
CN State
1 2 3 4 5 6 7 8 9 10 11 12 13 14 15 16 17 18 19 20 21 22 X Y
Chromosome

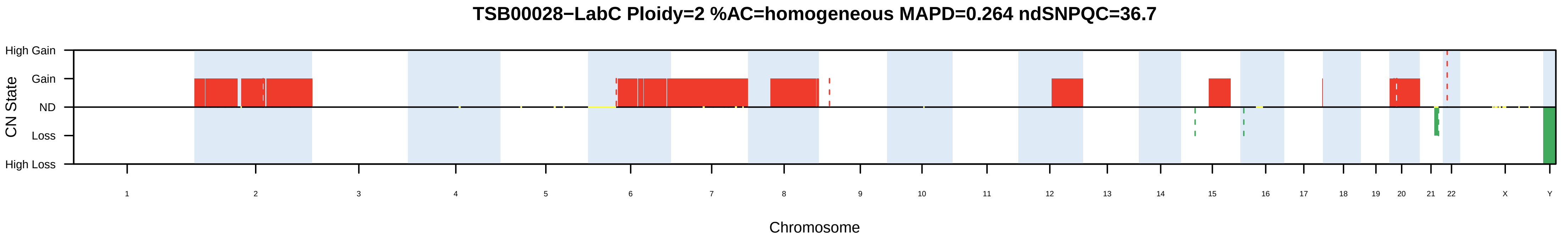
TSB00028−LabC Ploidy=2 %AC=homogeneous MAPD=0.264 ndSNPQC=36.7
High Gain
Gain
ND
Loss
High Loss
CN State
1 2 3 4 5 6 7 8 9 10 11 12 13 14 15 16 17 18 19 20 21 22 X Y
Chromosome

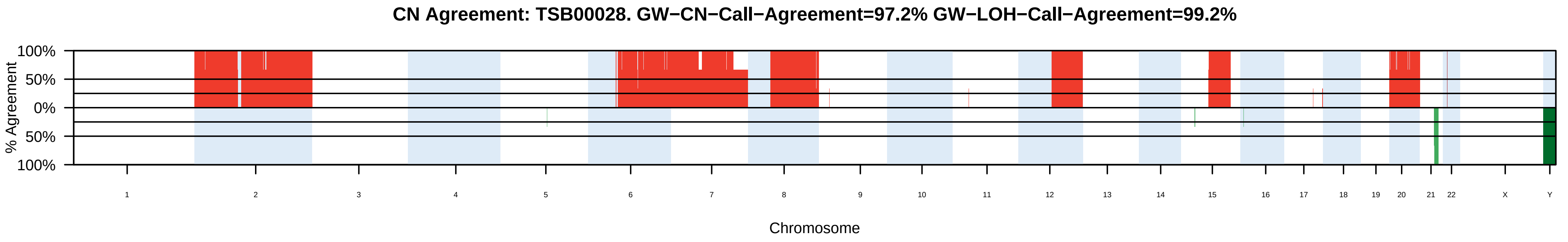
CN Agreement: TSB00028. GW−CN−Call−Agreement=97.2% GW−LOH−Call−Agreement=99.2%
100%
50%
0%
50%
100%
% Agreement
1 2 3 4 5 6 7 8 9 10 11 12 13 14 15 16 17 18 19 20 21 22 X Y
Chromosome

TSB00029−LabA Ploidy=2 %AC=75 MAPD=0.259 ndSNPQC=48.4

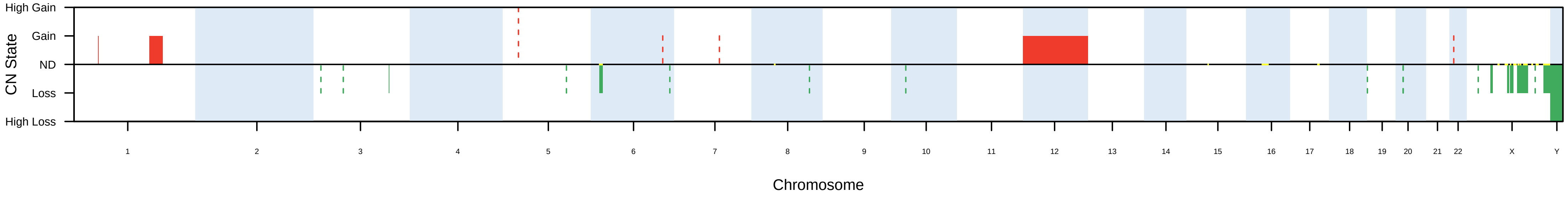

TSB00029−LabB Ploidy=2 %AC=80 MAPD=0.242 ndSNPQC=33.5

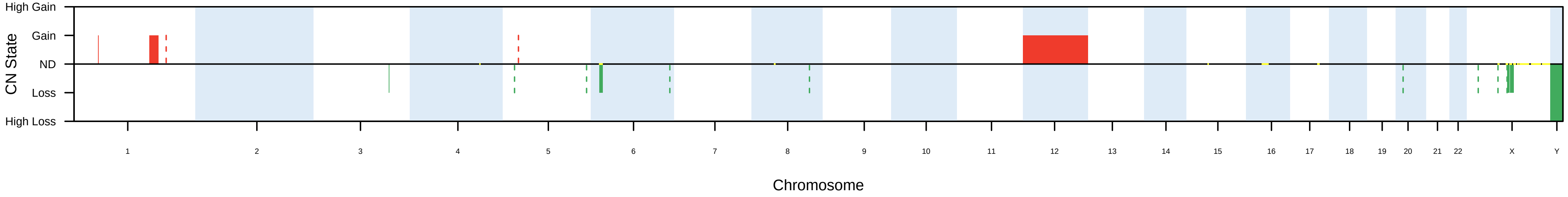

TSB00029−LabC Ploidy=2 %AC=75 MAPD=0.254 ndSNPQC=43.5

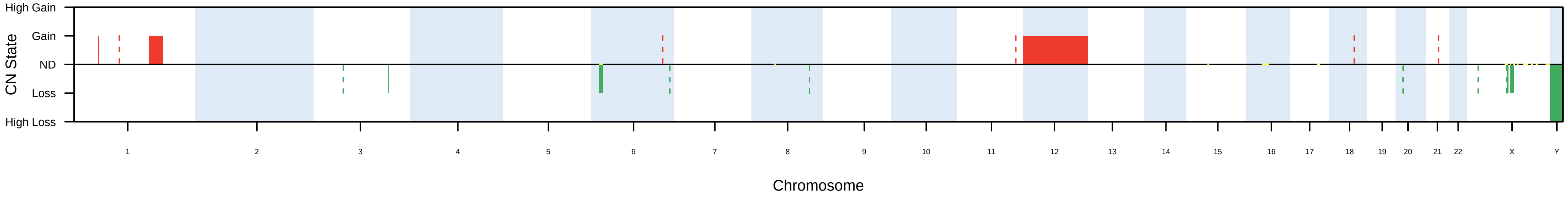

CN Agreement: TSB00029. GW−CN−Call−Agreement=97.9% GW−LOH−Call−Agreement=98.2%

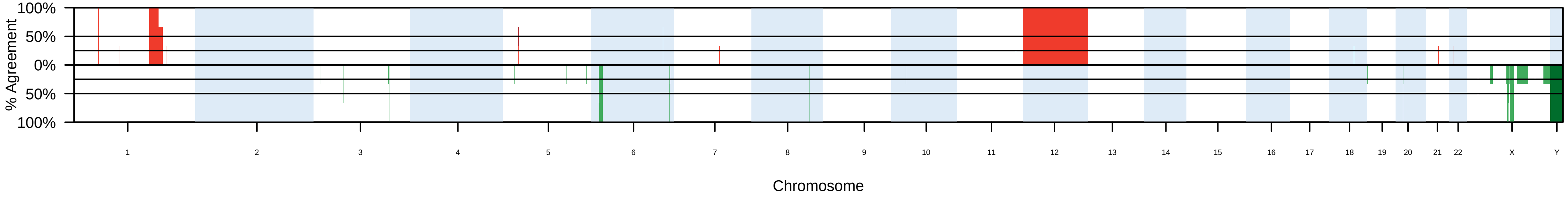

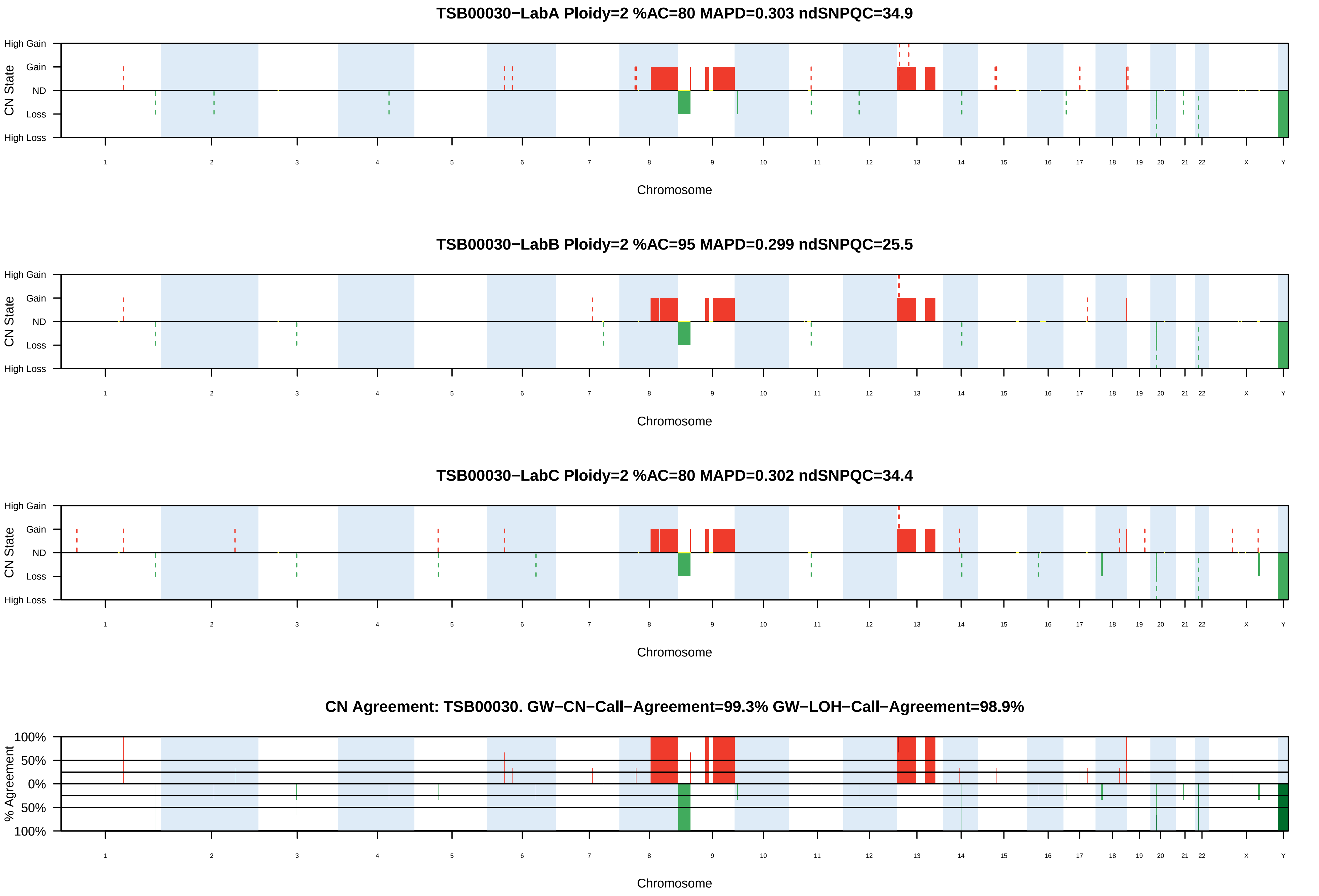

TSB00030−LabA Ploidy=2 %AC=80 MAPD=0.303 ndSNPQC=34.9
TSB00030−LabB Ploidy=2 %AC=95 MAPD=0.299 ndSNPQC=25.5
TSB00030−LabC Ploidy=2 %AC=80 MAPD=0.302 ndSNPQC=34.4
CN Agreement: TSB00030. GW−CN−Call−Agreement=99.3% GW−LOH−Call−Agreement=98.9%
CN State
High Gain
Gain
ND
Loss
High Loss
% Agreement
100%
50%
0%
50%
100%
Chromosome
1
2
3
4
5
6
7
8
9
10
11
12
13
14
15
16
17
18
19
20
21
22
X
Y

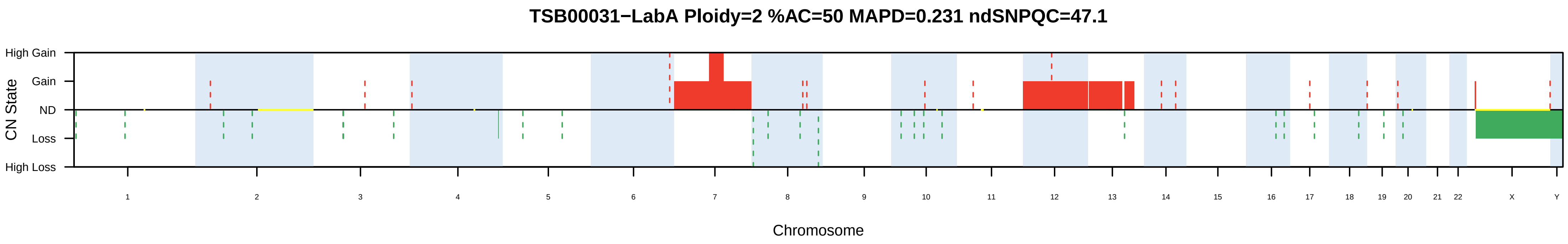
TSB00031−LabA Ploidy=2 %AC=50 MAPD=0.231 ndSNPQC=47.1
High Gain
Gain
ND
Loss
High Loss
CN State
1 2 3 4 5 6 7 8 9 10 11 12 13 14 15 16 17 18 19 20 21 22 X Y
Chromosome

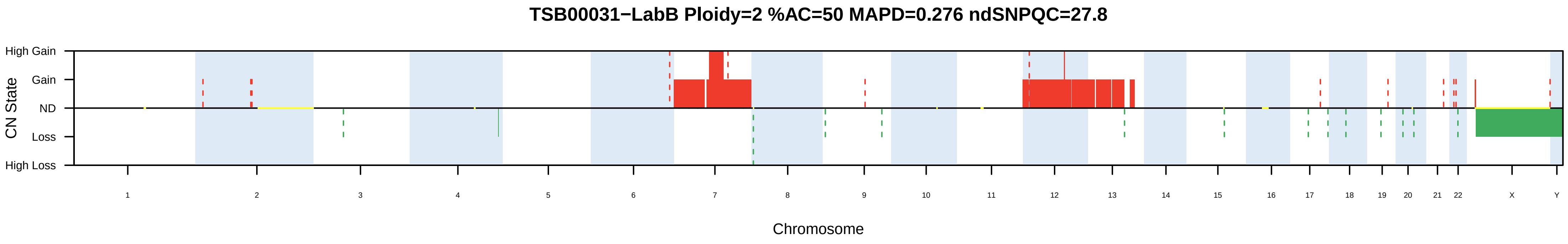
TSB00031−LabB Ploidy=2 %AC=50 MAPD=0.276 ndSNPQC=27.8
High Gain
Gain
ND
Loss
High Loss
CN State
1 2 3 4 5 6 7 8 9 10 11 12 13 14 15 16 17 18 19 20 21 22 X Y
Chromosome

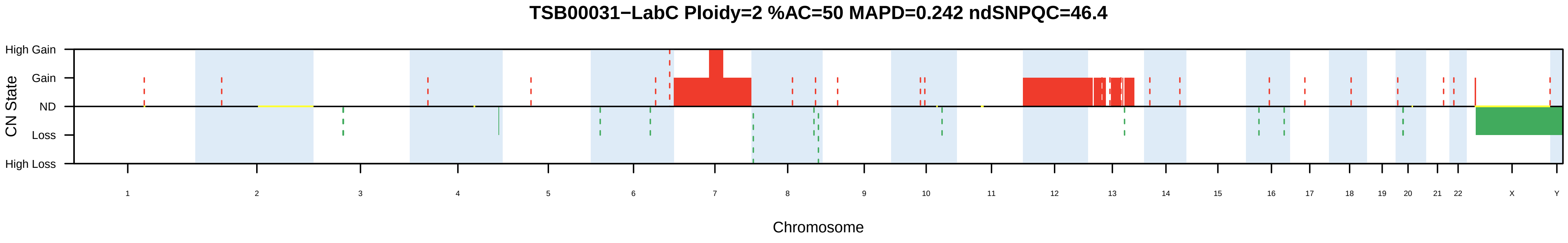
TSB00031−LabC Ploidy=2 %AC=50 MAPD=0.242 ndSNPQC=46.4
High Gain
Gain
ND
Loss
High Loss
CN State
1 2 3 4 5 6 7 8 9 10 11 12 13 14 15 16 17 18 19 20 21 22 X Y
Chromosome

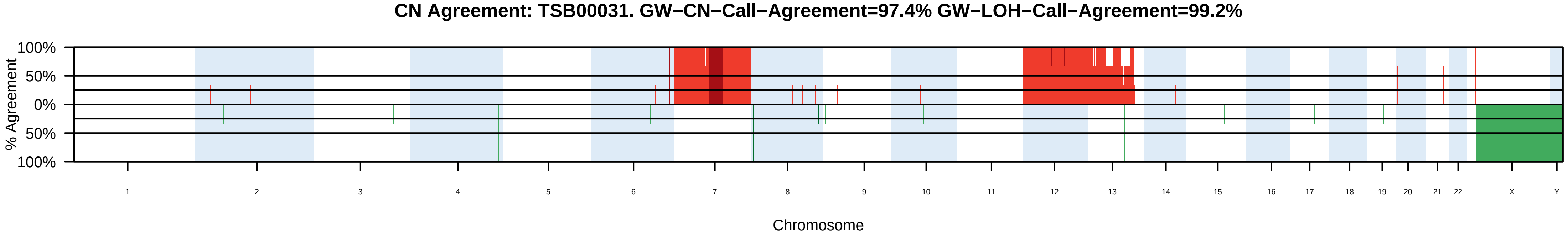
CN Agreement: TSB00031. GW−CN−Call−Agreement=97.4% GW−LOH−Call−Agreement=99.2%
100%
50%
0%
50%
100%
% Agreement
1 2 3 4 5 6 7 8 9 10 11 12 13 14 15 16 17 18 19 20 21 22 X Y
Chromosome

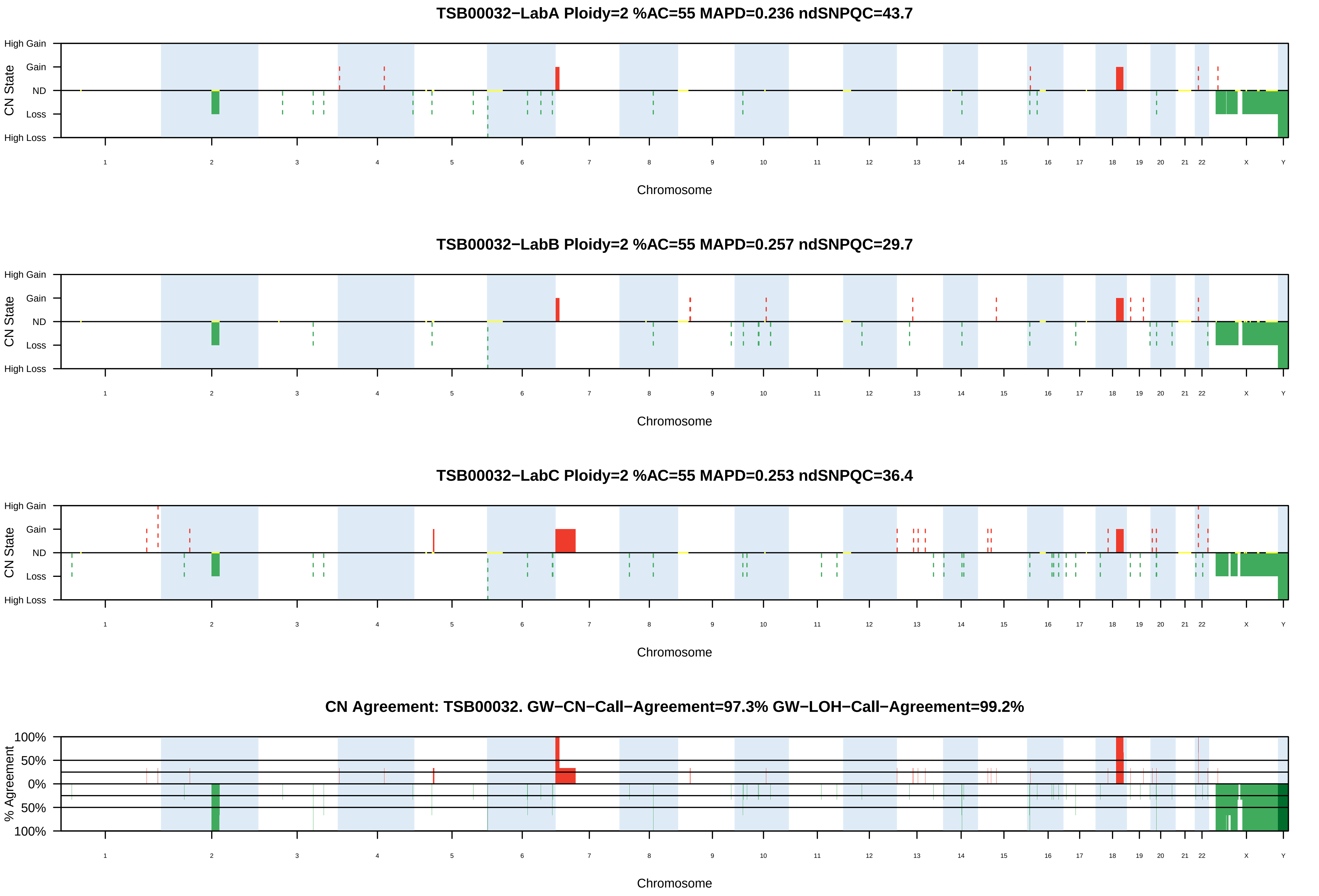

TSB00032−LabA Ploidy=2 %AC=55 MAPD=0.236 ndSNPQC=43.7
High Gain
Gain
ND
Loss
High Loss
CN State
1 2 3 4 5 6 7 8 9 10 11 12 13 14 15 16 17 18 19 20 21 22 X Y
Chromosome
TSB00032−LabB Ploidy=2 %AC=55 MAPD=0.257 ndSNPQC=29.7
High Gain
Gain
ND
Loss
High Loss
CN State
1 2 3 4 5 6 7 8 9 10 11 12 13 14 15 16 17 18 19 20 21 22 X Y
Chromosome
TSB00032−LabC Ploidy=2 %AC=55 MAPD=0.253 ndSNPQC=36.4
High Gain
Gain
ND
Loss
High Loss
CN State
1 2 3 4 5 6 7 8 9 10 11 12 13 14 15 16 17 18 19 20 21 22 X Y
Chromosome
CN Agreement: TSB00032. GW−CN−Call−Agreement=97.3% GW−LOH−Call−Agreement=99.2%
100%
50%
0%
50%
100%
% Agreement
1 2 3 4 5 6 7 8 9 10 11 12 13 14 15 16 17 18 19 20 21 22 X Y
Chromosome

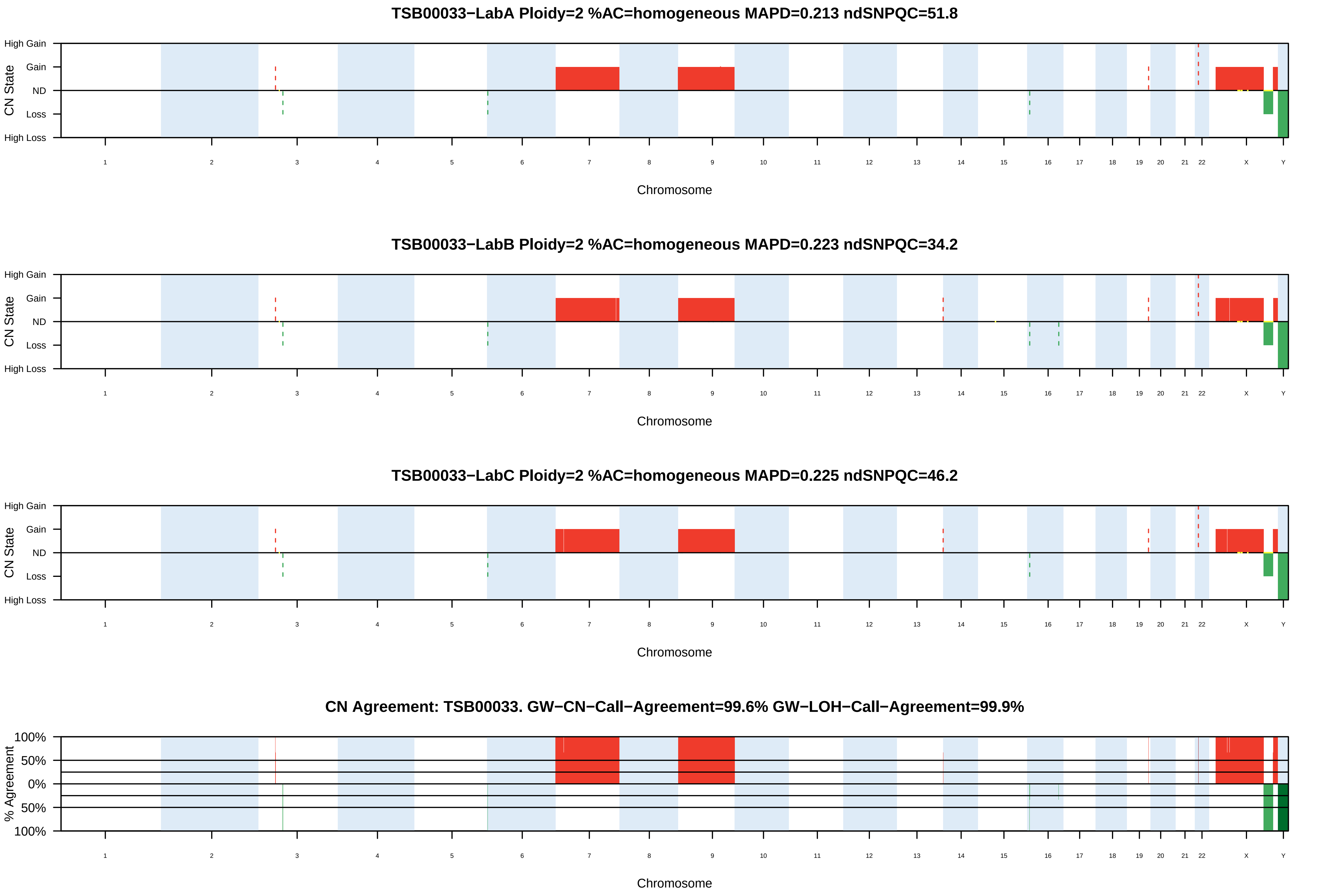
TSB00033−LabA Ploidy=2 %AC=homogeneous MAPD=0.213 ndSNPQC=51.8
TSB00033−LabB Ploidy=2 %AC=homogeneous MAPD=0.223 ndSNPQC=34.2
TSB00033−LabC Ploidy=2 %AC=homogeneous MAPD=0.225 ndSNPQC=46.2
CN State
High Gain
Gain
ND
Loss
High Loss
1 2 3 4 5 6 7 8 9 10 11 12 13 14 15 16 17 18 19 20 21 22 X Y
Chromosome
CN Agreement: TSB00033. GW−CN−Call−Agreement=99.6% GW−LOH−Call−Agreement=99.9%
% Agreement
100%
50%
0%
50%
100%

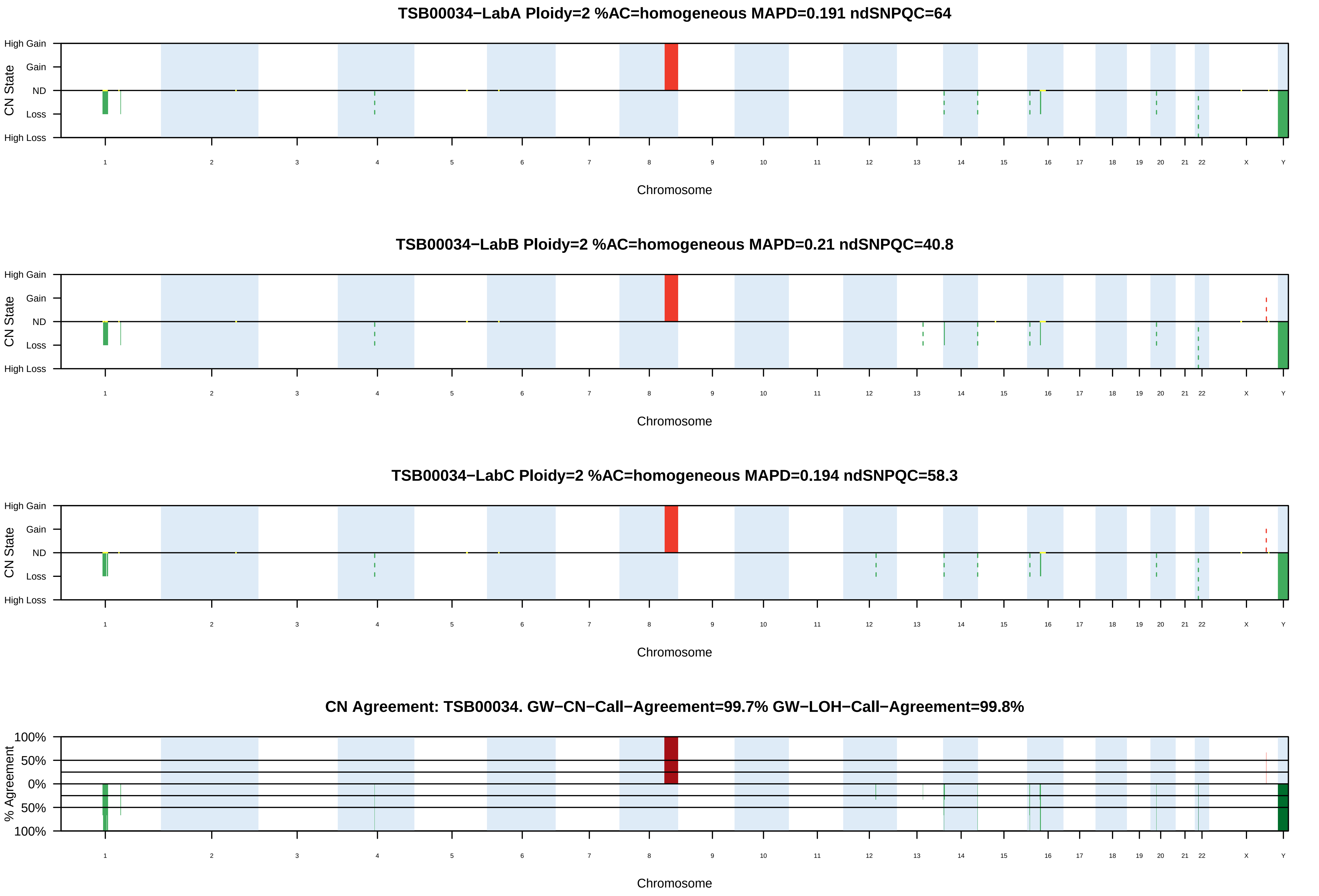

TSB00034−LabA Ploidy=2 %AC=homogeneous MAPD=0.191 ndSNPQC=64
High Gain
Gain
ND
Loss
High Loss
CN State
1 2 3 4 5 6 7 8 9 10 11 12 13 14 15 16 17 18 19 20 21 22 X Y
Chromosome
TSB00034−LabB Ploidy=2 %AC=homogeneous MAPD=0.21 ndSNPQC=40.8
High Gain
Gain
ND
Loss
High Loss
CN State
1 2 3 4 5 6 7 8 9 10 11 12 13 14 15 16 17 18 19 20 21 22 X Y
Chromosome
TSB00034−LabC Ploidy=2 %AC=homogeneous MAPD=0.194 ndSNPQC=58.3
High Gain
Gain
ND
Loss
High Loss
CN State
1 2 3 4 5 6 7 8 9 10 11 12 13 14 15 16 17 18 19 20 21 22 X Y
Chromosome
CN Agreement: TSB00034. GW−CN−Call−Agreement=99.7% GW−LOH−Call−Agreement=99.8%
100%
50%
0%
50%
100%
% Agreement
1 2 3 4 5 6 7 8 9 10 11 12 13 14 15 16 17 18 19 20 21 22 X Y
Chromosome

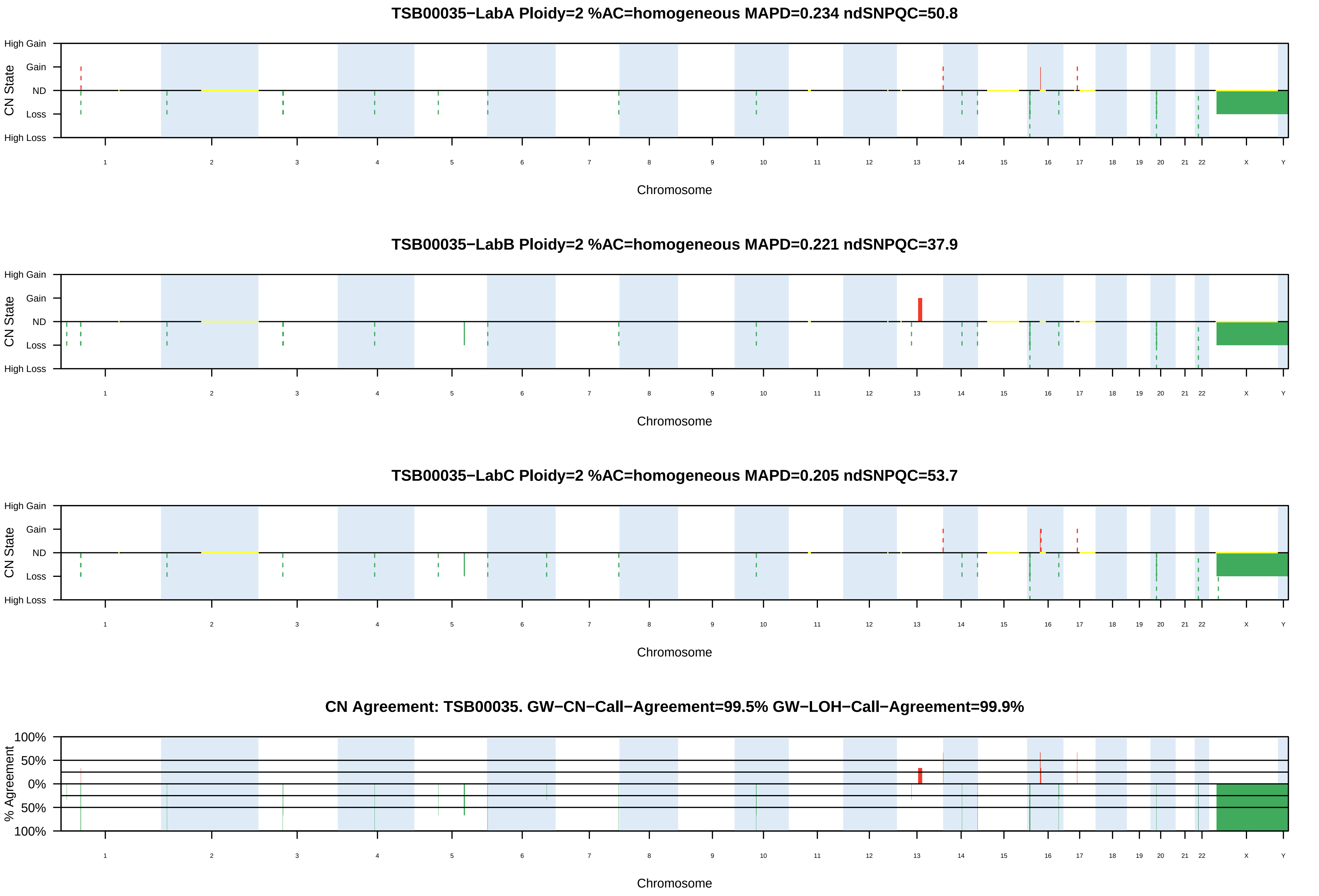
TSB00035−LabA Ploidy=2 %AC=homogeneous MAPD=0.234 ndSNPQC=50.8
High Gain
Gain
ND
Loss
High Loss
CN State
1 2 3 4 5 6 7 8 9 10 11 12 13 14 15 16 17 18 19 20 21 22 X Y
Chromosome
TSB00035−LabB Ploidy=2 %AC=homogeneous MAPD=0.221 ndSNPQC=37.9
High Gain
Gain
ND
Loss
High Loss
CN State
1 2 3 4 5 6 7 8 9 10 11 12 13 14 15 16 17 18 19 20 21 22 X Y
Chromosome
TSB00035−LabC Ploidy=2 %AC=homogeneous MAPD=0.205 ndSNPQC=53.7
High Gain
Gain
ND
Loss
High Loss
CN State
1 2 3 4 5 6 7 8 9 10 11 12 13 14 15 16 17 18 19 20 21 22 X Y
Chromosome
CN Agreement: TSB00035. GW−CN−Call−Agreement=99.5% GW−LOH−Call−Agreement=99.9%
100%
50%
0%
50%
100%
% Agreement
1 2 3 4 5 6 7 8 9 10 11 12 13 14 15 16 17 18 19 20 21 22 X Y
Chromosome

TSB00036−LabA Ploidy=2 %AC=65 MAPD=0.236 ndSNPQC=45.5

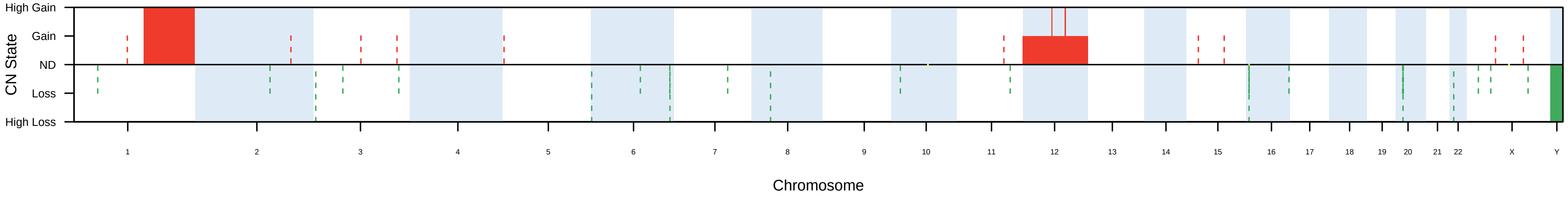

TSB00036−LabB Ploidy=2 %AC=60 MAPD=0.221 ndSNPQC=35.5

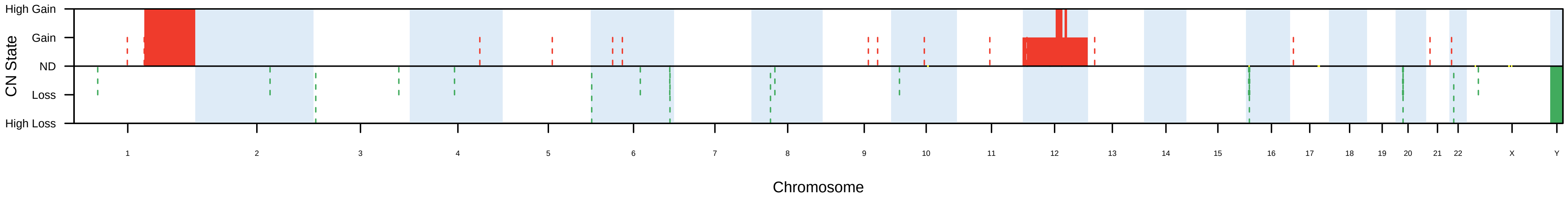

TSB00036−LabC Ploidy=2 %AC=60 MAPD=0.218 ndSNPQC=45

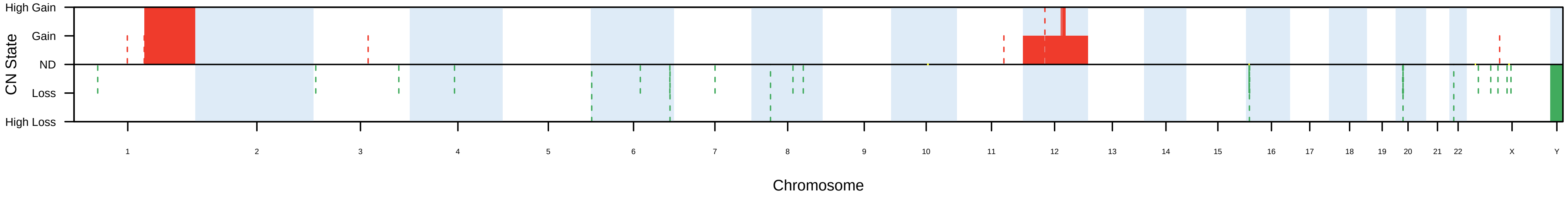

CN Agreement: TSB00036. GW−CN−Call−Agreement=98.6% GW−LOH−Call−Agreement=99.7%

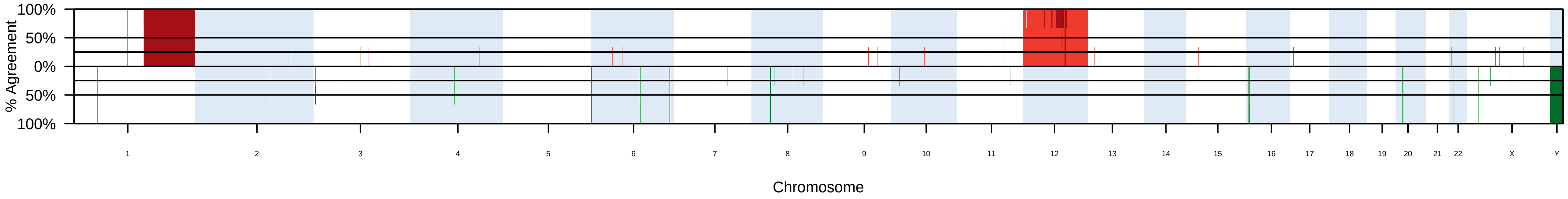

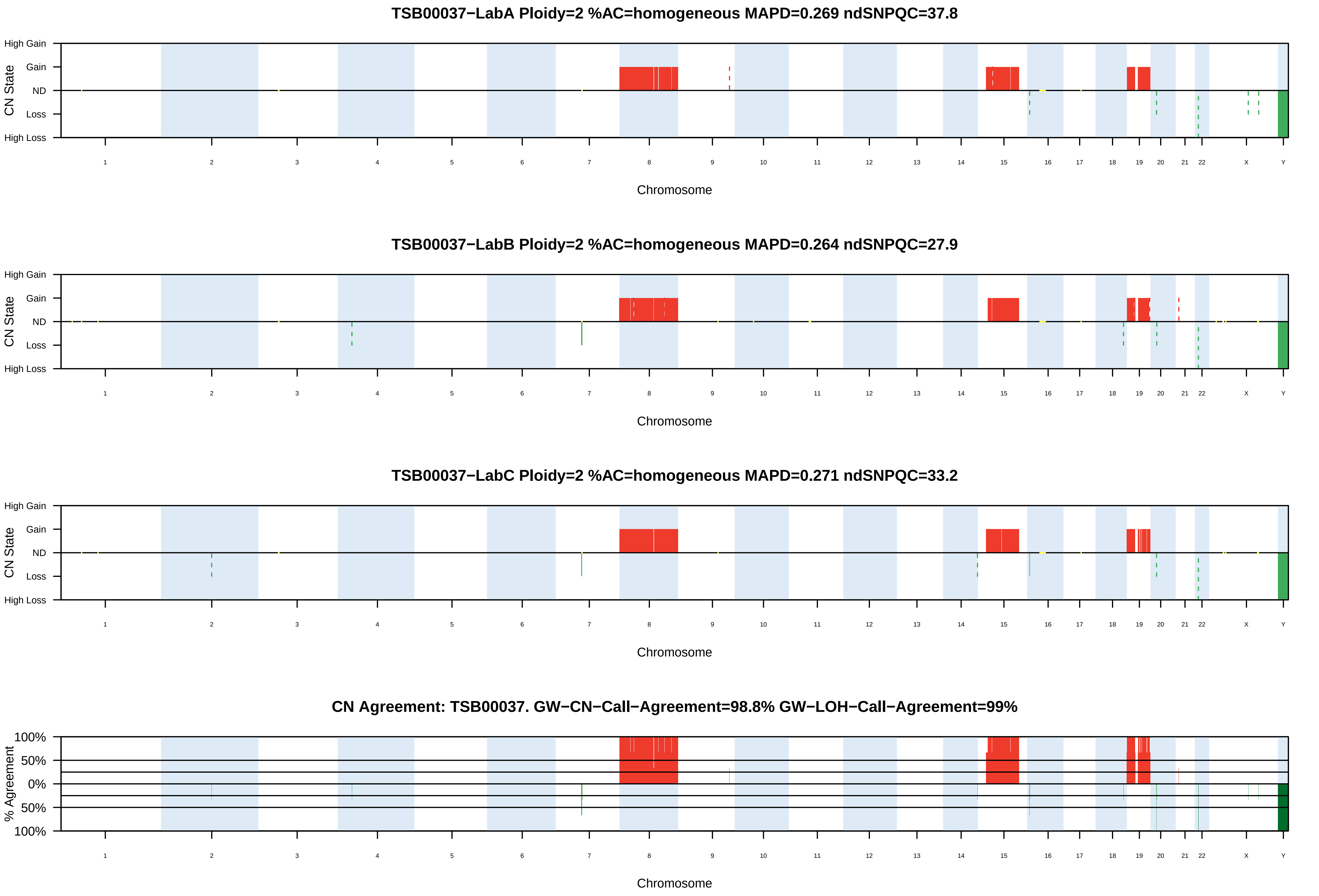
TSB00037−LabA Ploidy=2 %AC=homogeneous MAPD=0.269 ndSNPQC=37.8
TSB00037−LabB Ploidy=2 %AC=homogeneous MAPD=0.264 ndSNPQC=27.9
TSB00037−LabC Ploidy=2 %AC=homogeneous MAPD=0.271 ndSNPQC=33.2
CN Agreement: TSB00037. GW−CN−Call−Agreement=98.8% GW−LOH−Call−Agreement=99%
CN State
High Gain
Gain
ND
Loss
High Loss
% Agreement
100%
50%
0%
50%
100%
Chromosome
1 2 3 4 5 6 7 8 9 10 11 12 13 14 15 16 17 18 19 20 21 22 X Y

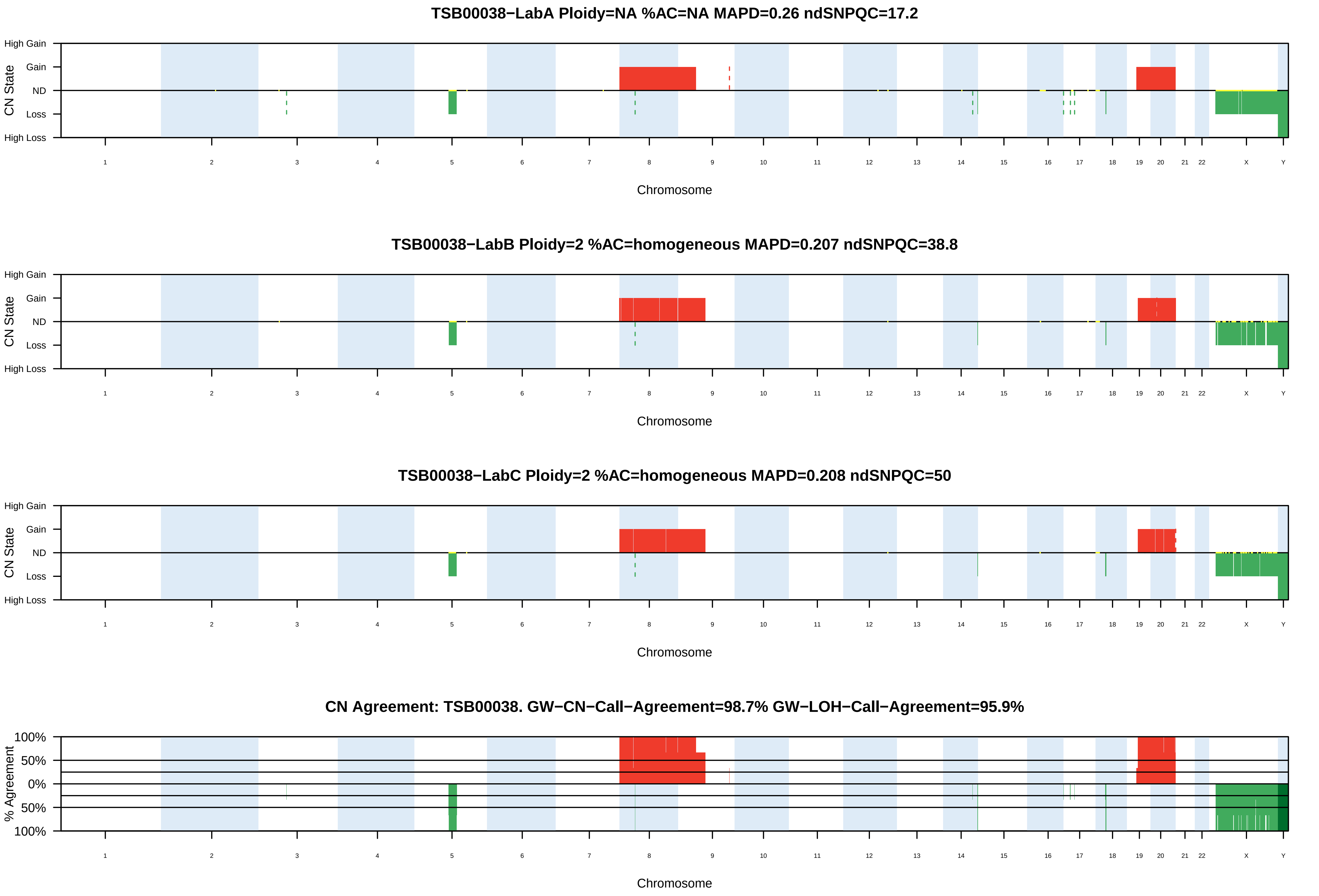
TSB00038−LabA Ploidy=NA %AC=NA MAPD=0.26 ndSNPQC=17.2
TSB00038−LabB Ploidy=2 %AC=homogeneous MAPD=0.207 ndSNPQC=38.8
TSB00038−LabC Ploidy=2 %AC=homogeneous MAPD=0.208 ndSNPQC=50
CN Agreement: TSB00038. GW−CN−Call−Agreement=98.7% GW−LOH−Call−Agreement=95.9%
CN State
High Gain
Gain
ND
Loss
High Loss
% Agreement
100%
50%
0%
50%
100%
Chromosome
1 2 3 4 5 6 7 8 9 10 11 12 13 14 15 16 17 18 19 20 21 22 X Y

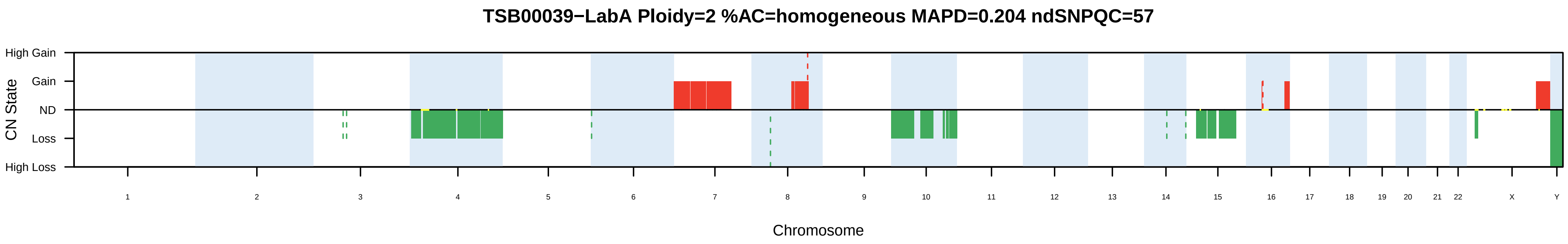
TSB00039−LabA Ploidy=2 %AC=homogeneous MAPD=0.204 ndSNPQC=57
CN State
High Gain
Gain
ND
Loss
High Loss
1 2 3 4 5 6 7 8 9 10 11 12 13 14 15 16 17 18 19 20 21 22 X Y
Chromosome

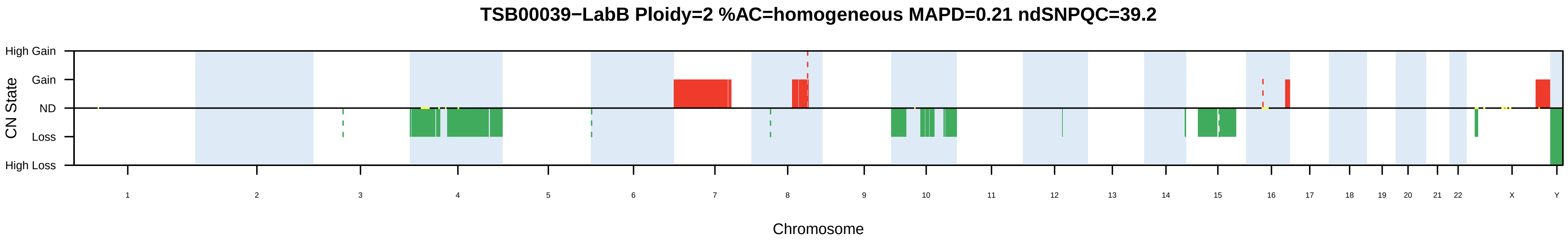
TSB00039−LabB Ploidy=2 %AC=homogeneous MAPD=0.21 ndSNPQC=39.2
CN State
High Gain
Gain
ND
Loss
High Loss
1 2 3 4 5 6 7 8 9 10 11 12 13 14 15 16 17 18 19 20 21 22 X Y
Chromosome

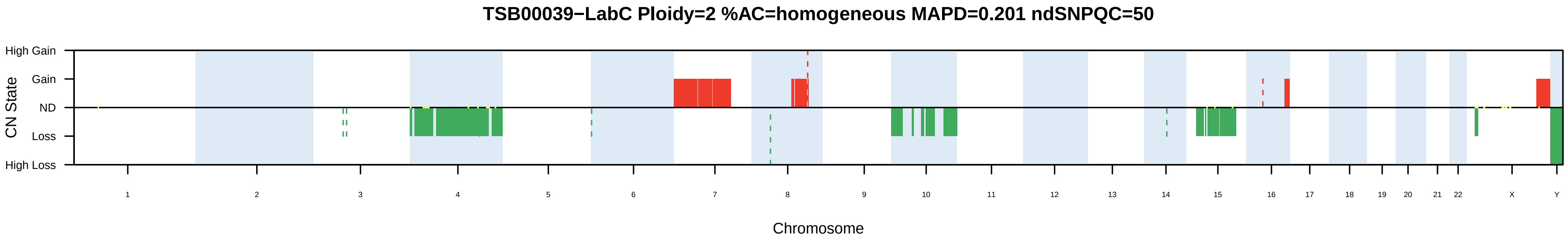
TSB00039−LabC Ploidy=2 %AC=homogeneous MAPD=0.201 ndSNPQC=50
CN State
High Gain
Gain
ND
Loss
High Loss
1 2 3 4 5 6 7 8 9 10 11 12 13 14 15 16 17 18 19 20 21 22 X Y
Chromosome

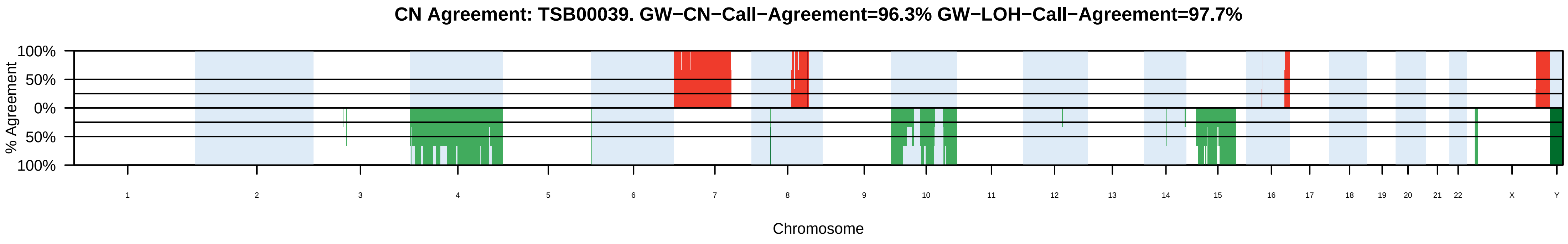
CN Agreement: TSB00039. GW−CN−Call−Agreement=96.3% GW−LOH−Call−Agreement=97.7%
% Agreement
100%
50%
0%
50%
100%
1 2 3 4 5 6 7 8 9 10 11 12 13 14 15 16 17 18 19 20 21 22 X Y
Chromosome

TSB00040−LabA Ploidy=2 %AC=60 MAPD=0.211 ndSNPQC=47

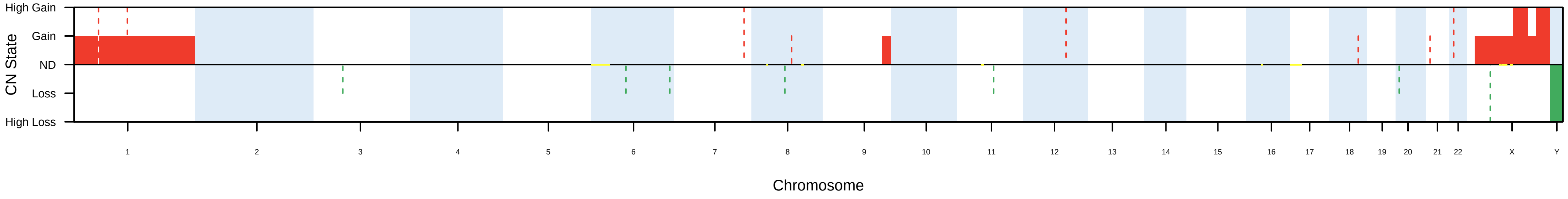

TSB00040−LabB Ploidy=2 %AC=60 MAPD=0.236 ndSNPQC=32.8

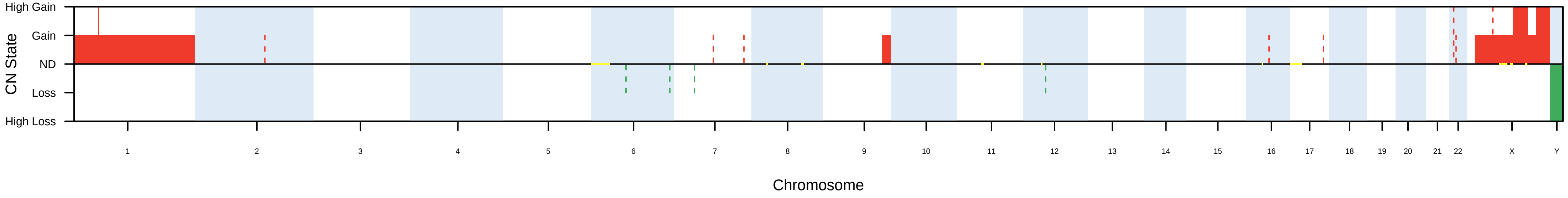

TSB00040−LabC Ploidy=2 %AC=60 MAPD=0.23 ndSNPQC=38

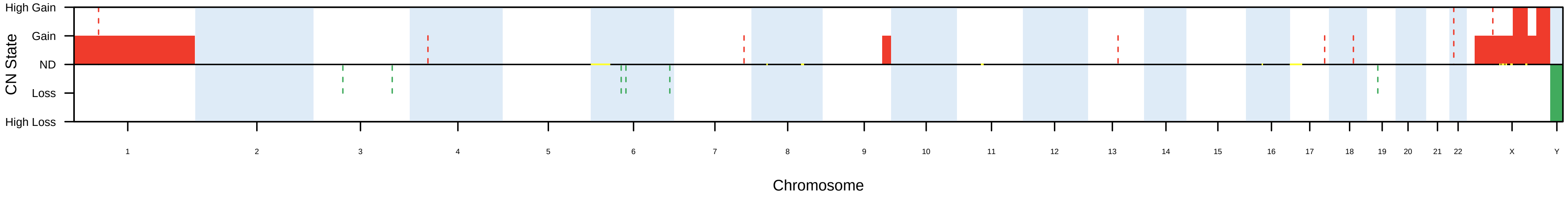

CN Agreement: TSB00040. GW−CN−Call−Agreement=99.7% GW−LOH−Call−Agreement=99.7%

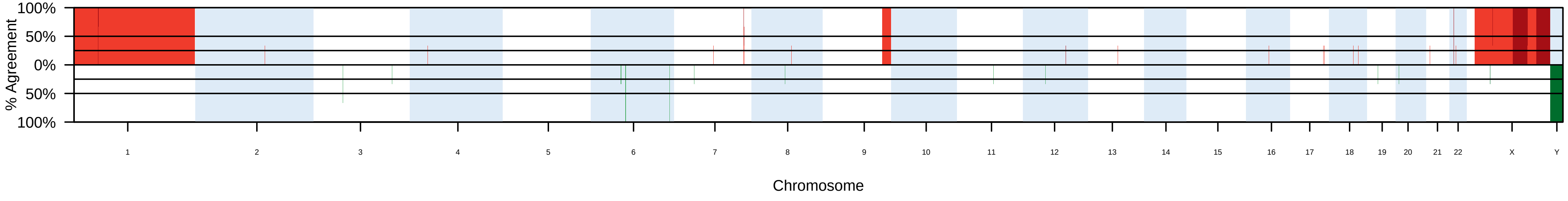

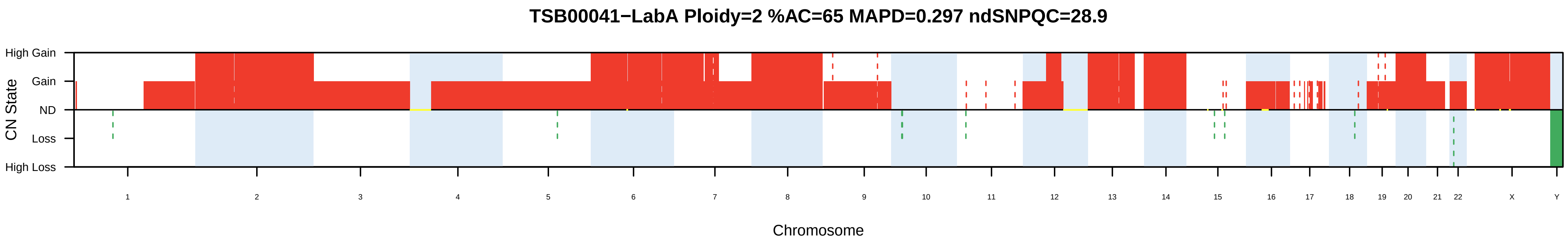
TSB00041−LabA Ploidy=2 %AC=65 MAPD=0.297 ndSNPQC=28.9
CN State
High Gain
Gain
ND
Loss
High Loss
1 2 3 4 5 6 7 8 9 10 11 12 13 14 15 16 17 18 19 20 21 22 X Y
Chromosome

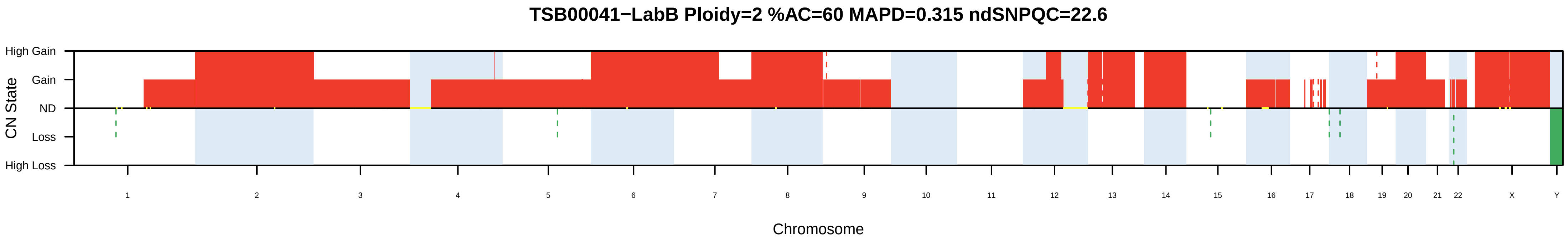
TSB00041−LabB Ploidy=2 %AC=60 MAPD=0.315 ndSNPQC=22.6
CN State
High Gain
Gain
ND
Loss
High Loss
1 2 3 4 5 6 7 8 9 10 11 12 13 14 15 16 17 18 19 20 21 22 X Y
Chromosome

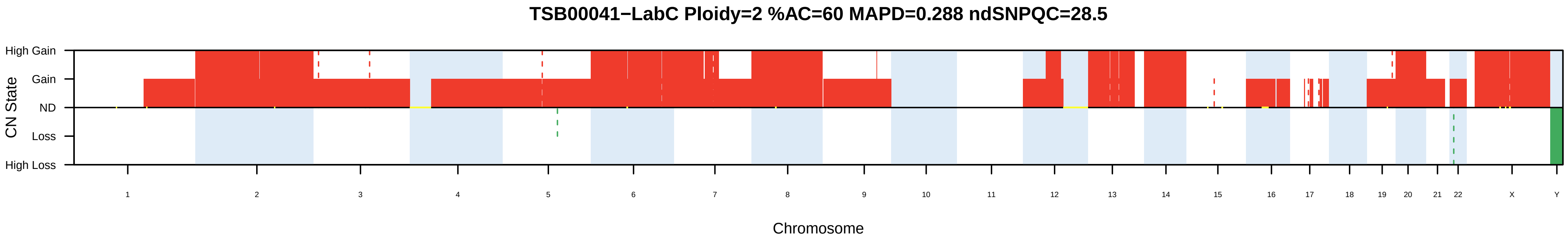
TSB00041−LabC Ploidy=2 %AC=60 MAPD=0.288 ndSNPQC=28.5
CN State
High Gain
Gain
ND
Loss
High Loss
1 2 3 4 5 6 7 8 9 10 11 12 13 14 15 16 17 18 19 20 21 22 X Y
Chromosome

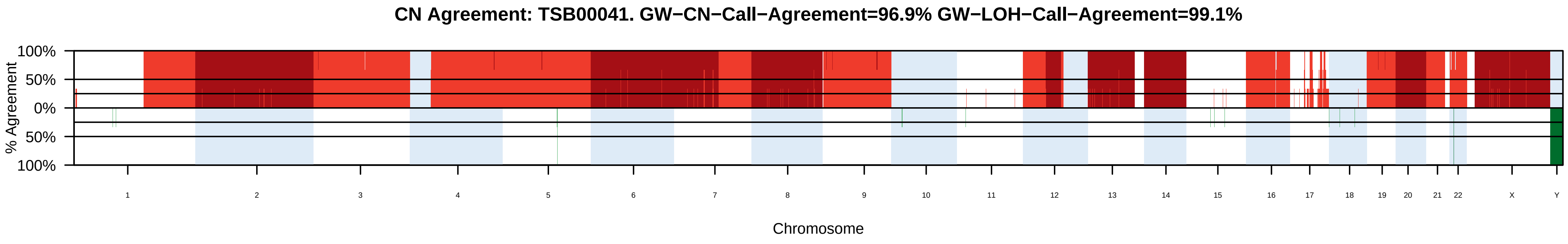
CN Agreement: TSB00041. GW−CN−Call−Agreement=96.9% GW−LOH−Call−Agreement=99.1%
% Agreement
100%
50%
0%
50%
100%
1 2 3 4 5 6 7 8 9 10 11 12 13 14 15 16 17 18 19 20 21 22 X Y
Chromosome

TSB00042−LabA Ploidy=4 %AC=40 MAPD=0.203 ndSNPQC=49.4

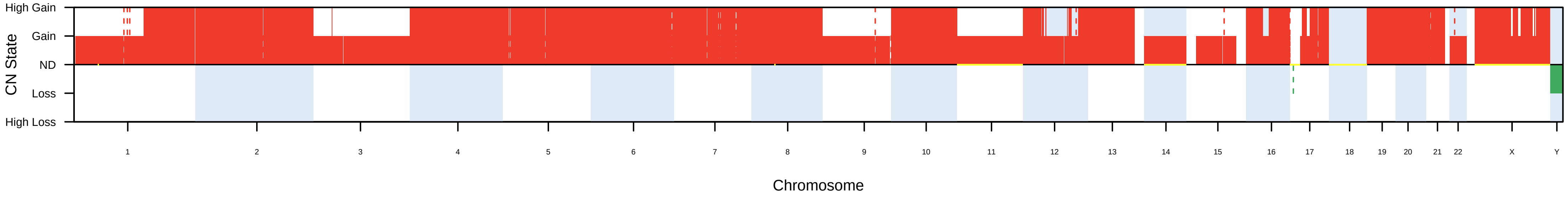

TSB00042−LabB Ploidy=4 %AC=40 MAPD=0.201 ndSNPQC=43.7

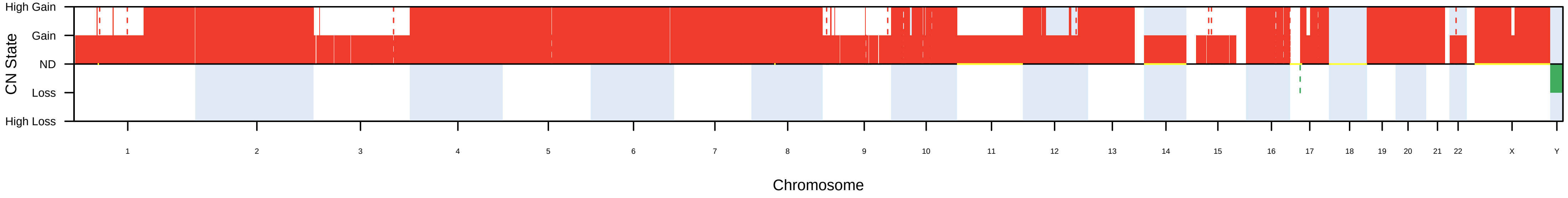

TSB00042−LabC Ploidy=4 %AC=40 MAPD=0.199 ndSNPQC=60.1

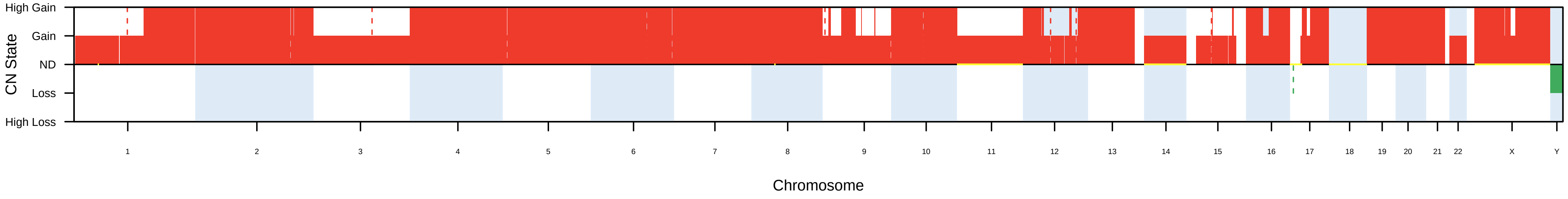

CN Agreement: TSB00042. GW−CN−Call−Agreement=95.4% GW−LOH−Call−Agreement=99.9%

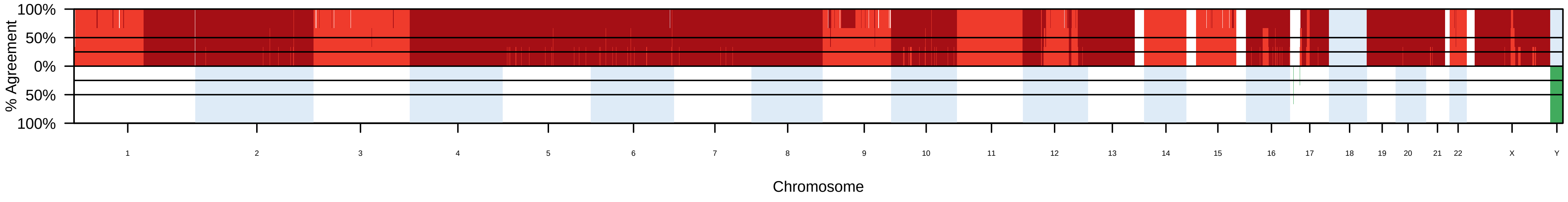

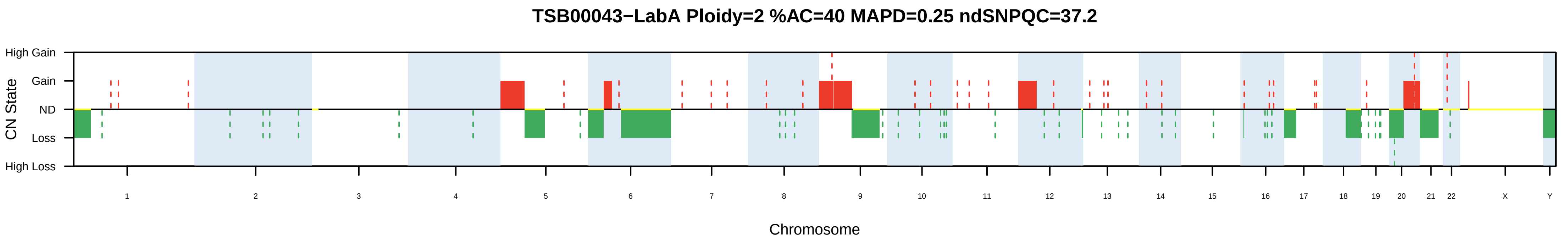
TSB00043−LabA Ploidy=2 %AC=40 MAPD=0.25 ndSNPQC=37.2
High Gain
Gain
ND
Loss
High Loss
CN State
1 2 3 4 5 6 7 8 9 10 11 12 13 14 15 16 17 18 19 20 21 22 X Y
Chromosome

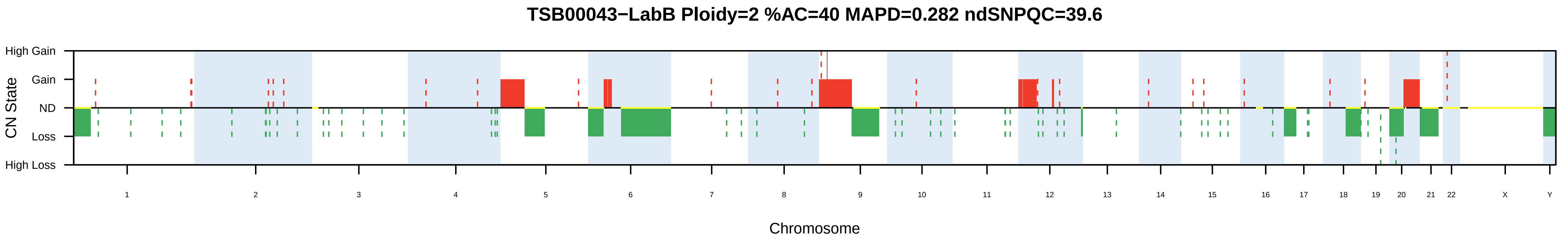
TSB00043−LabB Ploidy=2 %AC=40 MAPD=0.282 ndSNPQC=39.6
High Gain
Gain
ND
Loss
High Loss
CN State
1 2 3 4 5 6 7 8 9 10 11 12 13 14 15 16 17 18 19 20 21 22 X Y
Chromosome

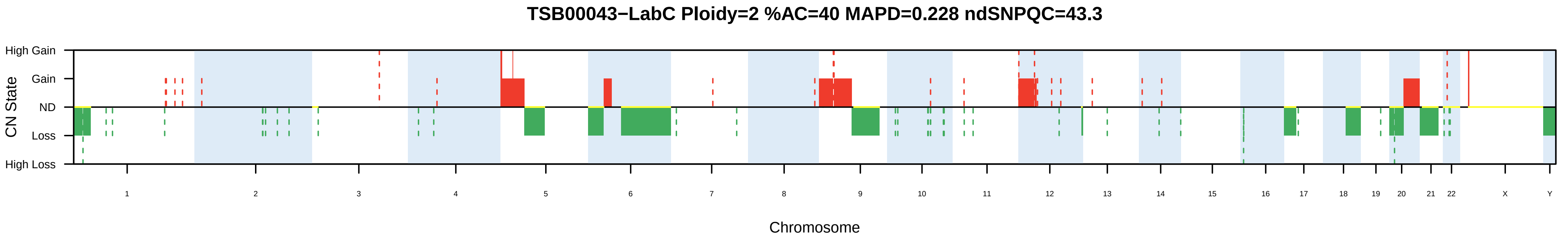
TSB00043−LabC Ploidy=2 %AC=40 MAPD=0.228 ndSNPQC=43.3
High Gain
Gain
ND
Loss
High Loss
CN State
1 2 3 4 5 6 7 8 9 10 11 12 13 14 15 16 17 18 19 20 21 22 X Y
Chromosome

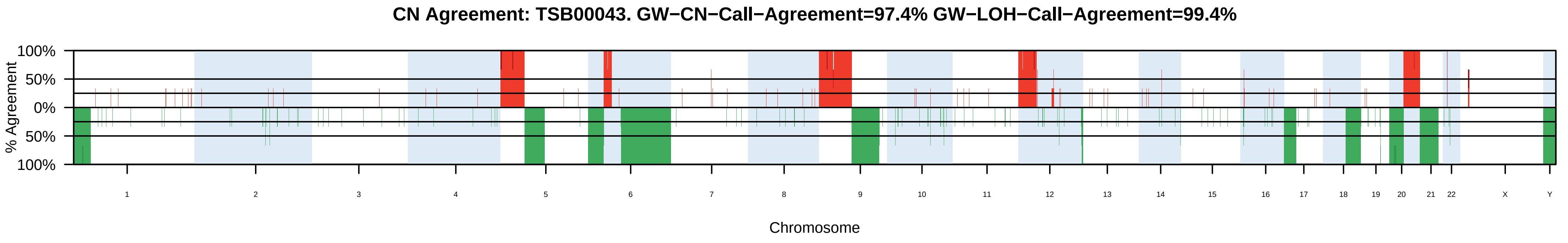
CN Agreement: TSB00043. GW−CN−Call−Agreement=97.4% GW−LOH−Call−Agreement=99.4%
100%
50%
0%
50%
100%
% Agreement
1 2 3 4 5 6 7 8 9 10 11 12 13 14 15 16 17 18 19 20 21 22 X Y
Chromosome

TSB00044−LabA Ploidy=NA %AC=NA MAPD=0.268 ndSNPQC=33.9

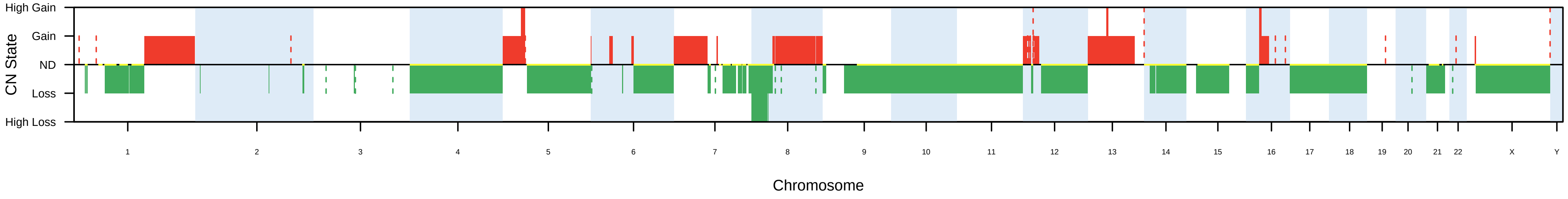

TSB00044−LabB Ploidy=NA %AC=NA MAPD=0.244 ndSNPQC=31.8

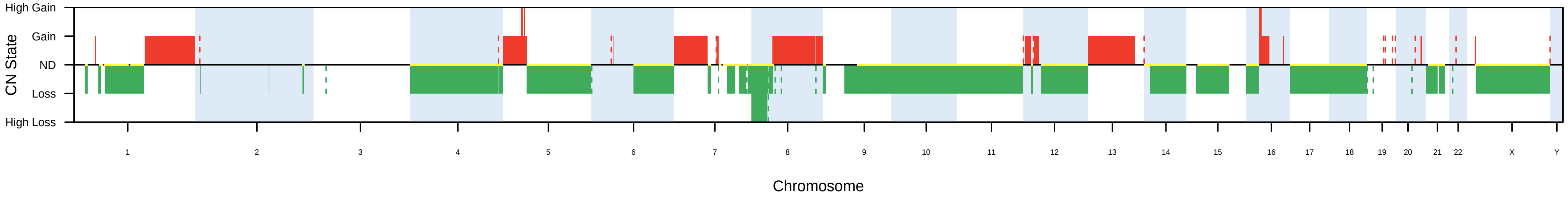

TSB00044−LabC Ploidy=NA %AC=NA MAPD=0.251 ndSNPQC=38

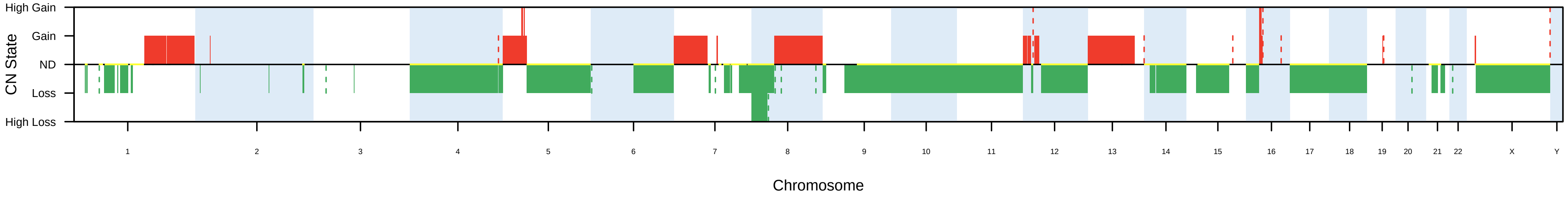

CN Agreement: TSB00044. GW−CN−Call−Agreement=95.3% GW−LOH−Call−Agreement=98.7%

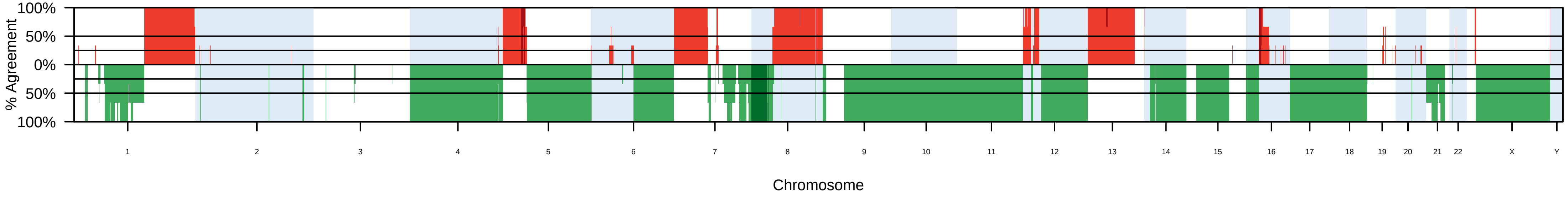

TSB00045−LabA Ploidy=2 %AC=65 MAPD=0.201 ndSNPQC=59.4

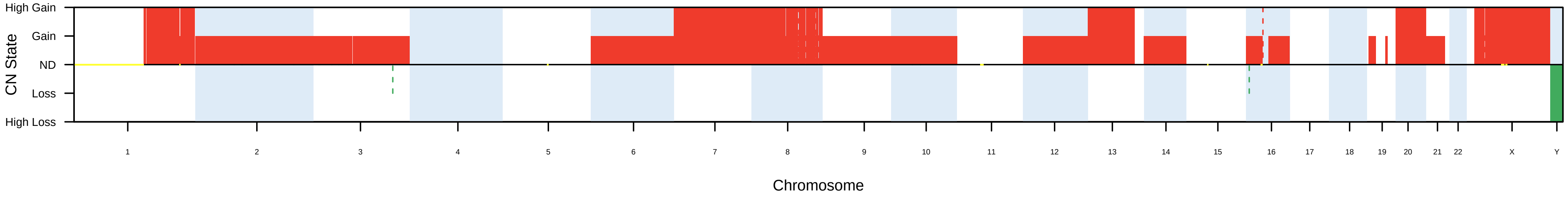

TSB00045−LabB Ploidy=2 %AC=65 MAPD=0.213 ndSNPQC=38.9

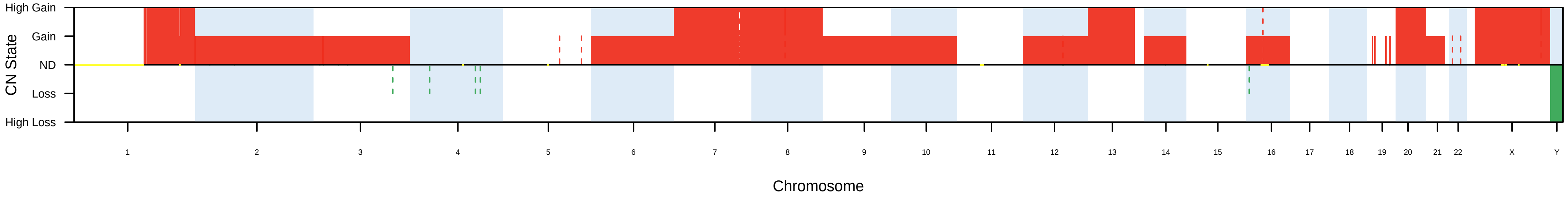

TSB00045−LabC Ploidy=2 %AC=70 MAPD=0.208 ndSNPQC=52.8

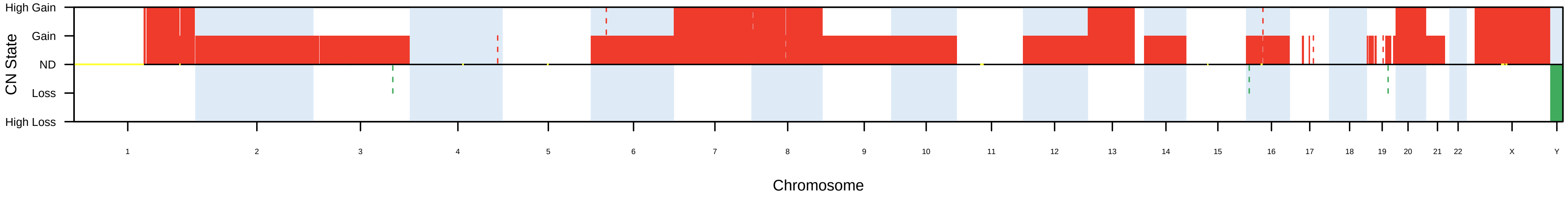

CN Agreement: TSB00045. GW−CN−Call−Agreement=96.7% GW−LOH−Call−Agreement=99.4%

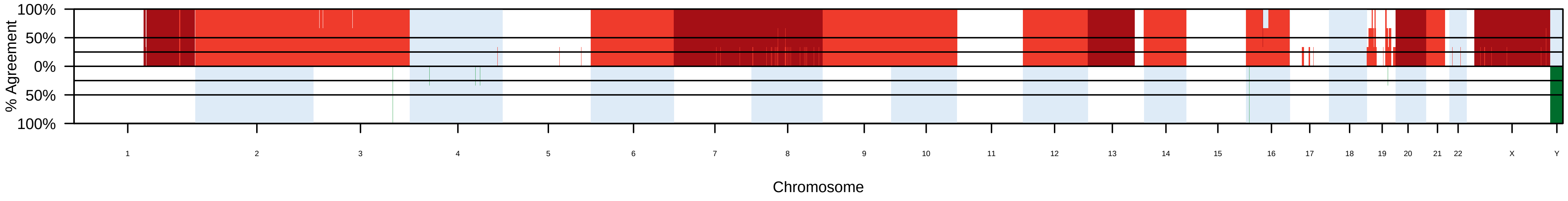

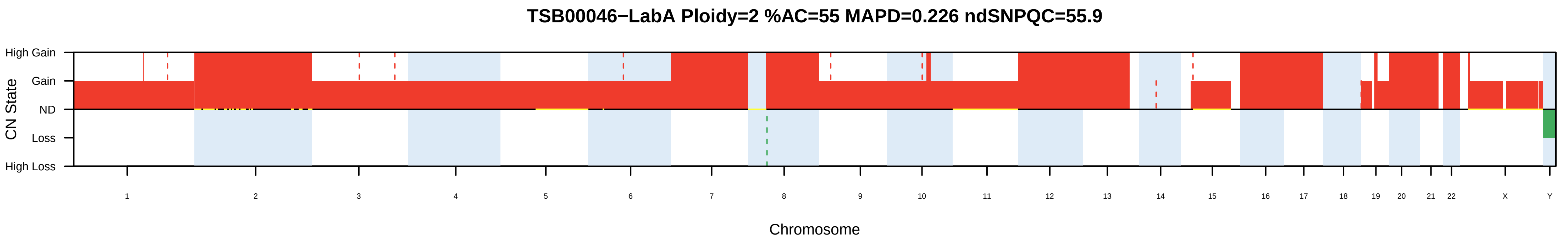

TSB00046−LabA Ploidy=2 %AC=55 MAPD=0.226 ndSNPQC=55.9
CN State
High Gain
Gain
ND
Loss
High Loss
1 2 3 4 5 6 7 8 9 10 11 12 13 14 15 16 17 18 19 20 21 22 X Y
Chromosome

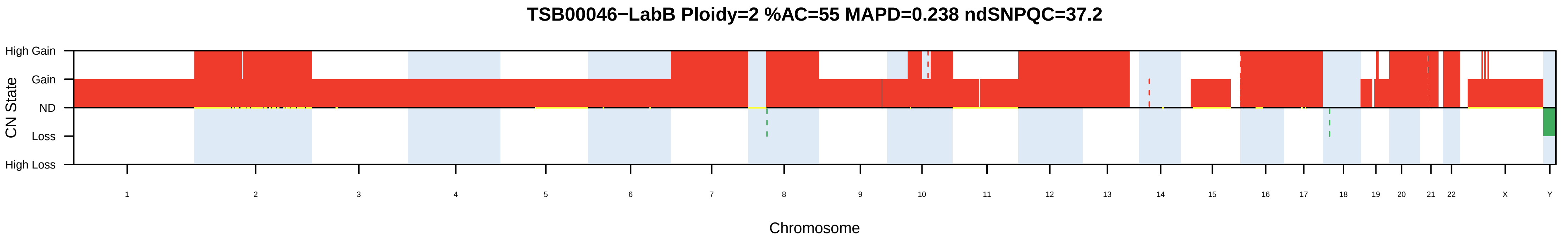

TSB00046−LabB Ploidy=2 %AC=55 MAPD=0.238 ndSNPQC=37.2
CN State
High Gain
Gain
ND
Loss
High Loss
1 2 3 4 5 6 7 8 9 10 11 12 13 14 15 16 17 18 19 20 21 22 X Y
Chromosome

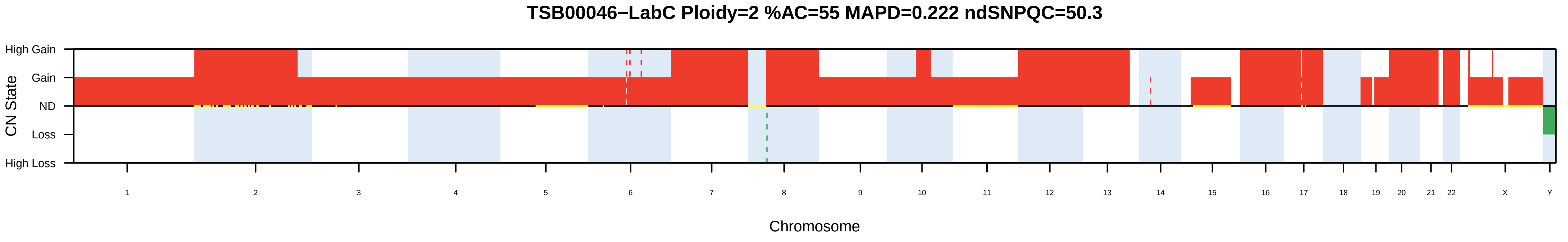

TSB00046−LabC Ploidy=2 %AC=55 MAPD=0.222 ndSNPQC=50.3
CN State
High Gain
Gain
ND
Loss
High Loss
1 2 3 4 5 6 7 8 9 10 11 12 13 14 15 16 17 18 19 20 21 22 X Y
Chromosome

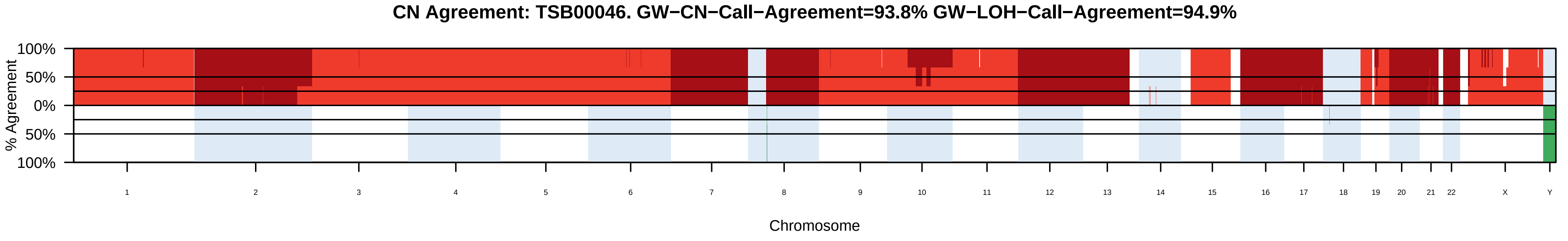

CN Agreement: TSB00046. GW−CN−Call−Agreement=93.8% GW−LOH−Call−Agreement=94.9%
% Agreement
100%
50%
0%
50%
100%
1 2 3 4 5 6 7 8 9 10 11 12 13 14 15 16 17 18 19 20 21 22 X Y
Chromosome

TSB00047−LabA Ploidy=2 %AC=65 MAPD=0.308 ndSNPQC=25.4

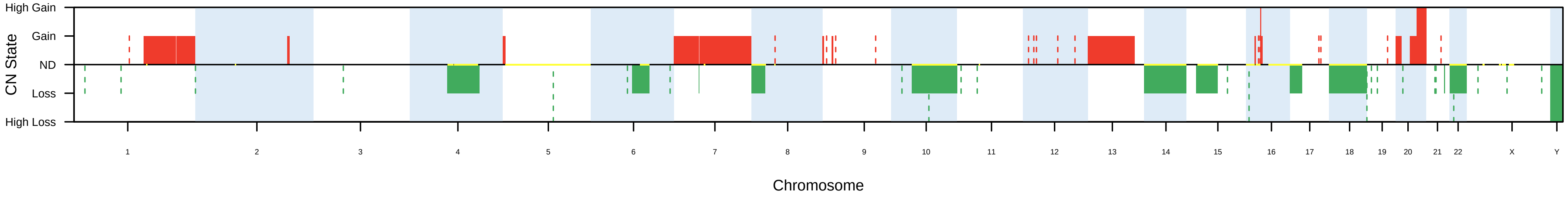

TSB00047−LabB Ploidy=2 %AC=65 MAPD=0.346 ndSNPQC=21.1

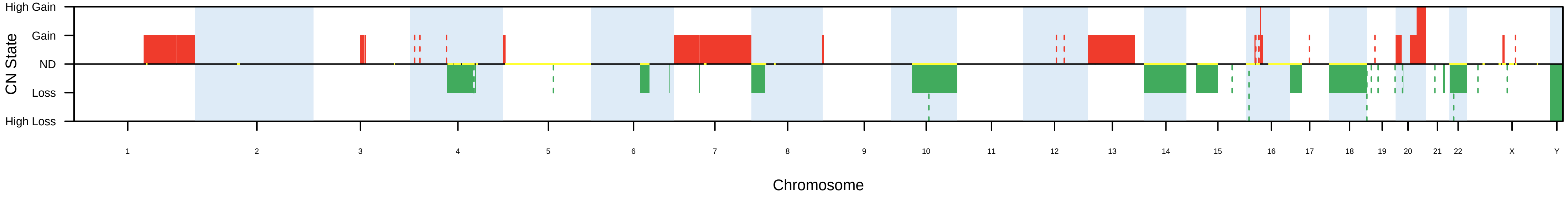

TSB00047−LabC Ploidy=2 %AC=65 MAPD=0.311 ndSNPQC=24.3

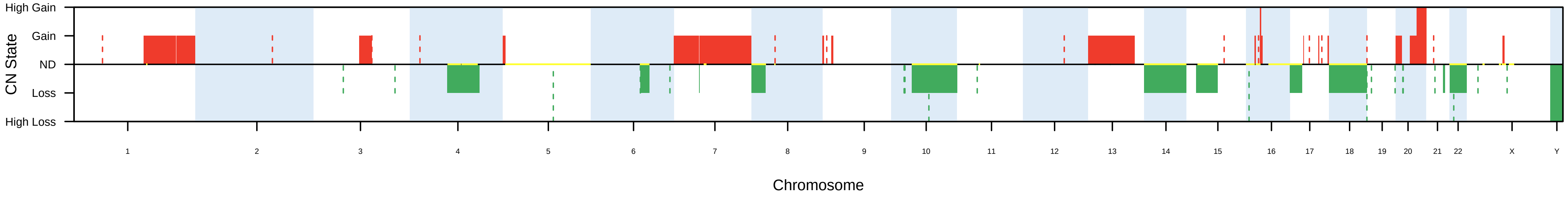

CN Agreement: TSB00047. GW−CN−Call−Agreement=96.5% GW−LOH−Call−Agreement=98.9%

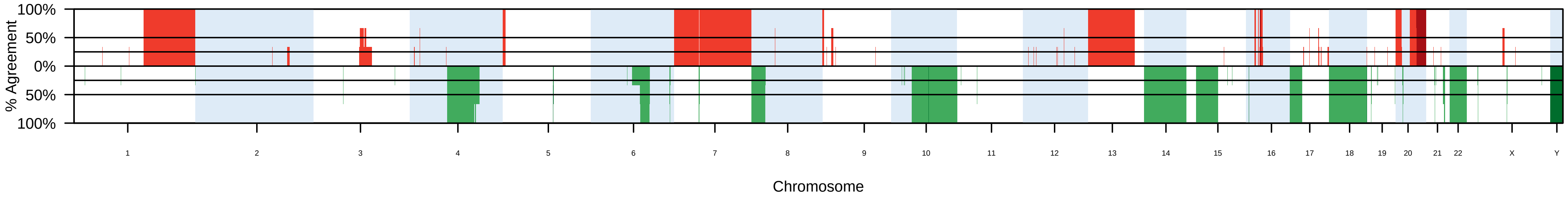

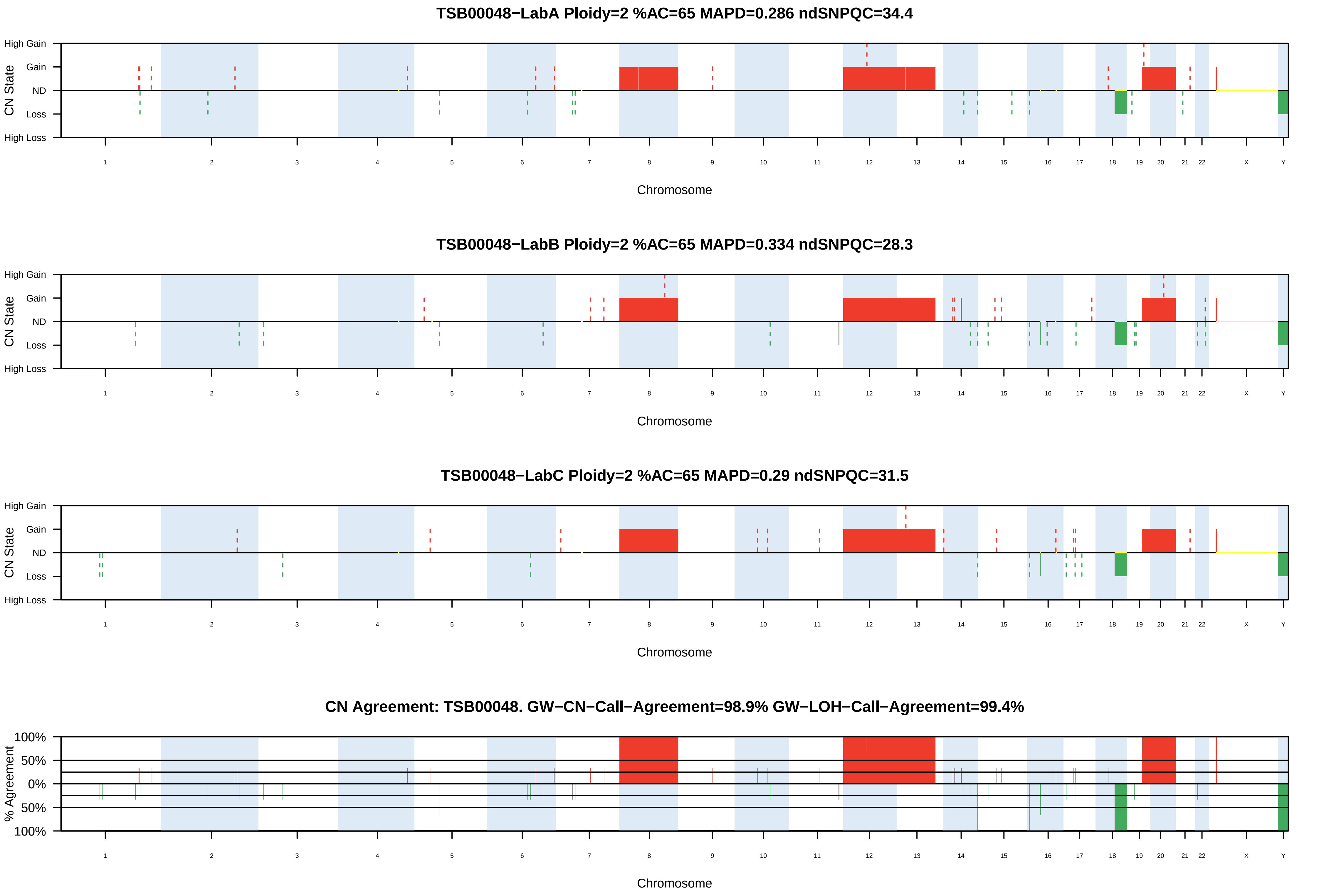
TSB00048−LabA Ploidy=2 %AC=65 MAPD=0.286 ndSNPQC=34.4
High Gain
Gain
ND
Loss
High Loss
CN State
1 2 3 4 5 6 7 8 9 10 11 12 13 14 15 16 17 18 19 20 21 22 X Y
Chromosome
TSB00048−LabB Ploidy=2 %AC=65 MAPD=0.334 ndSNPQC=28.3
High Gain
Gain
ND
Loss
High Loss
CN State
1 2 3 4 5 6 7 8 9 10 11 12 13 14 15 16 17 18 19 20 21 22 X Y
Chromosome
TSB00048−LabC Ploidy=2 %AC=65 MAPD=0.29 ndSNPQC=31.5
High Gain
Gain
ND
Loss
High Loss
CN State
1 2 3 4 5 6 7 8 9 10 11 12 13 14 15 16 17 18 19 20 21 22 X Y
Chromosome
CN Agreement: TSB00048. GW−CN−Call−Agreement=98.9% GW−LOH−Call−Agreement=99.4%
100%
50%
0%
50%
100%
% Agreement
1 2 3 4 5 6 7 8 9 10 11 12 13 14 15 16 17 18 19 20 21 22 X Y
Chromosome

TSB00049−LabA Ploidy=2 %AC=60 MAPD=0.214 ndSNPQC=57.5

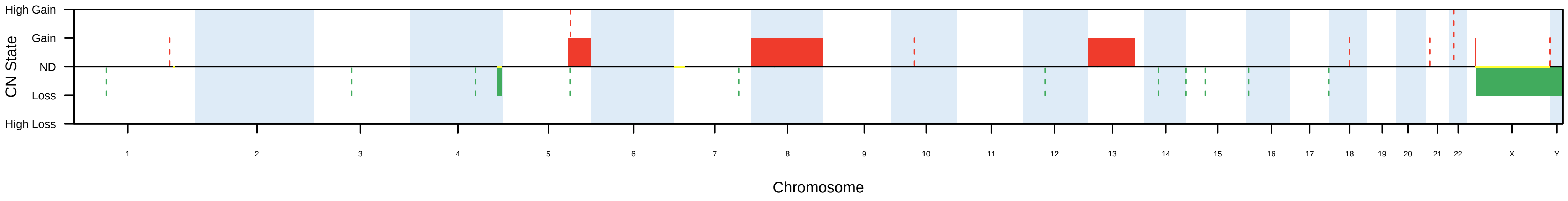

TSB00049−LabB Ploidy=2 %AC=60 MAPD=0.218 ndSNPQC=40.1

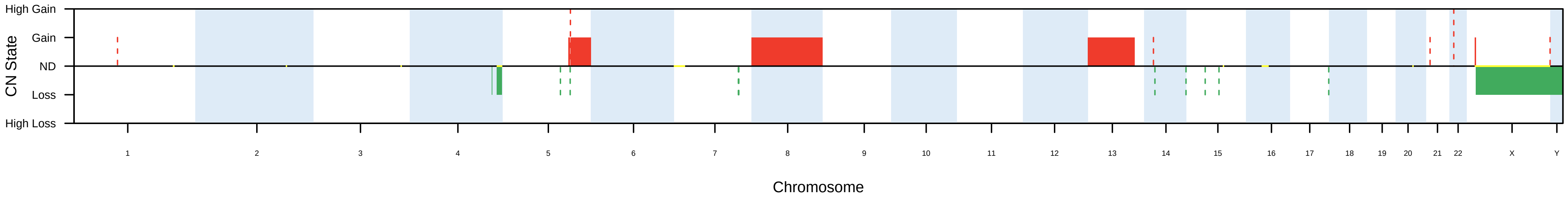

TSB00049−LabC Ploidy=2 %AC=60 MAPD=0.217 ndSNPQC=59.6

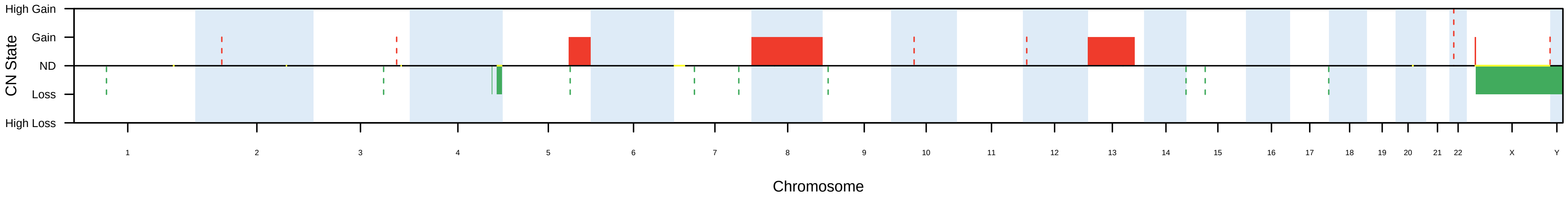

CN Agreement: TSB00049. GW−CN−Call−Agreement=99.7% GW−LOH−Call−Agreement=99.1%

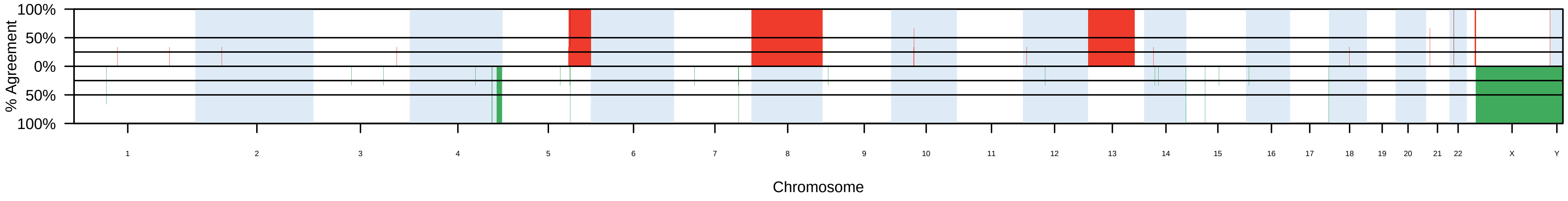

TSB00050−LabA Ploidy=2 %AC=70 MAPD=0.209 ndSNPQC=52.7

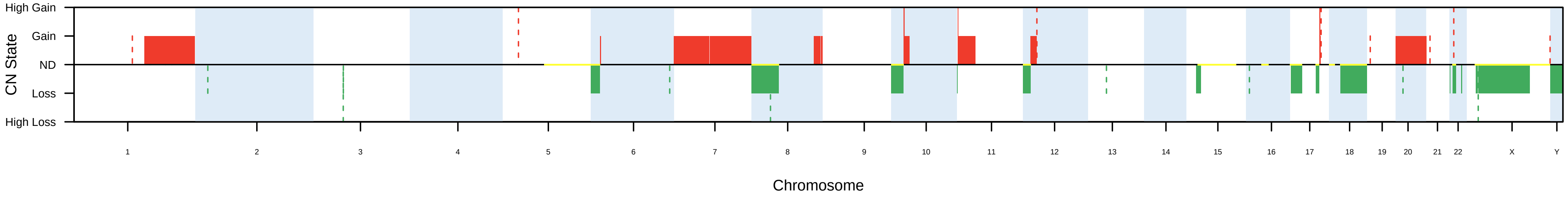

TSB00050−LabB Ploidy=2 %AC=70 MAPD=0.214 ndSNPQC=36.1

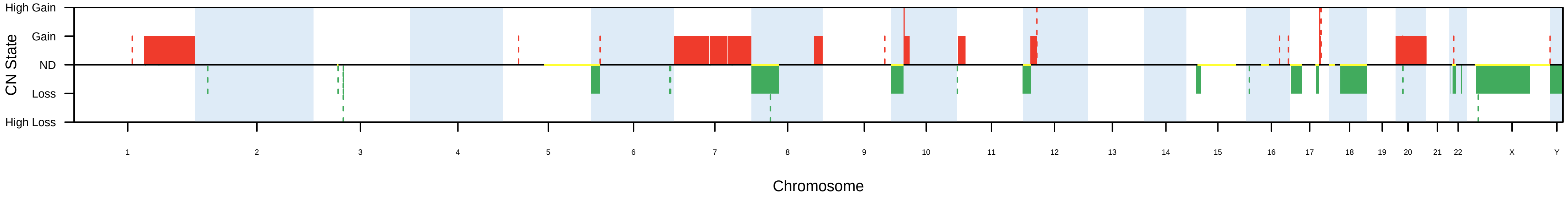

TSB00050−LabC Ploidy=2 %AC=70 MAPD=0.21 ndSNPQC=52.9

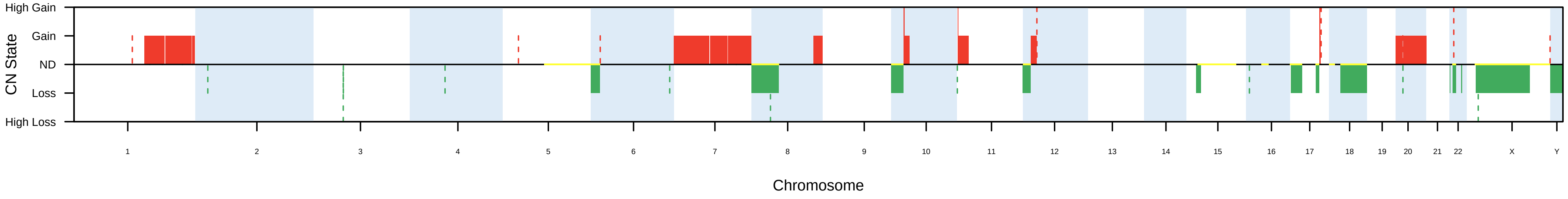

CN Agreement: TSB00050. GW−CN−Call−Agreement=98.7% GW−LOH−Call−Agreement=99.9%

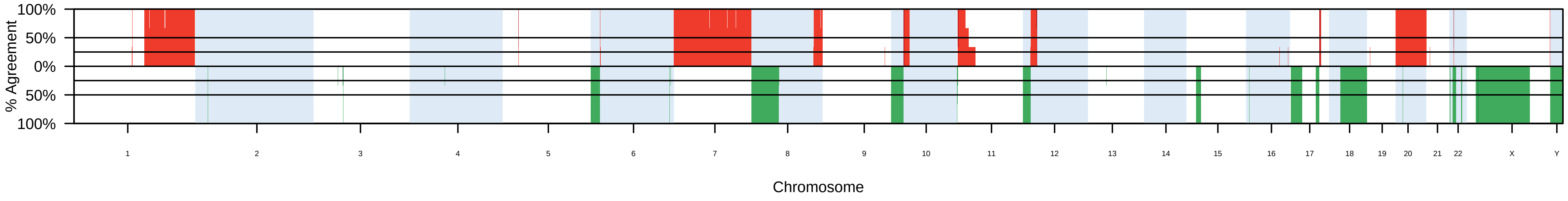

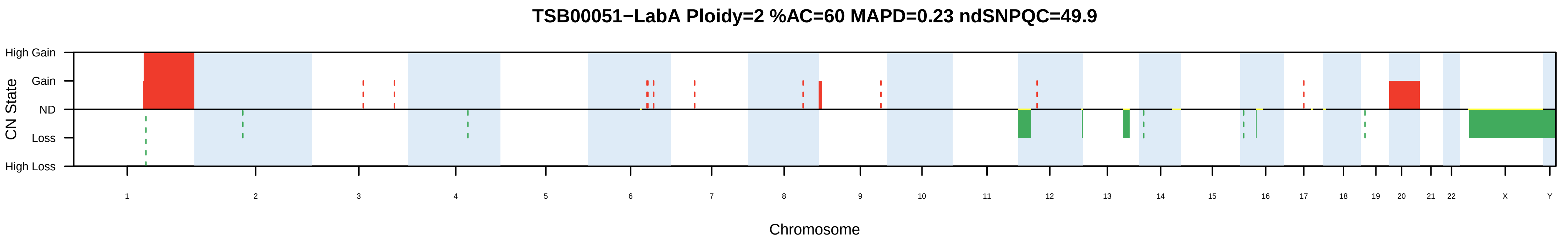
TSB00051−LabA Ploidy=2 %AC=60 MAPD=0.23 ndSNPQC=49.9
High Gain
Gain
ND
Loss
High Loss
CN State
1 2 3 4 5 6 7 8 9 10 11 12 13 14 15 16 17 18 19 20 21 22 X Y
Chromosome

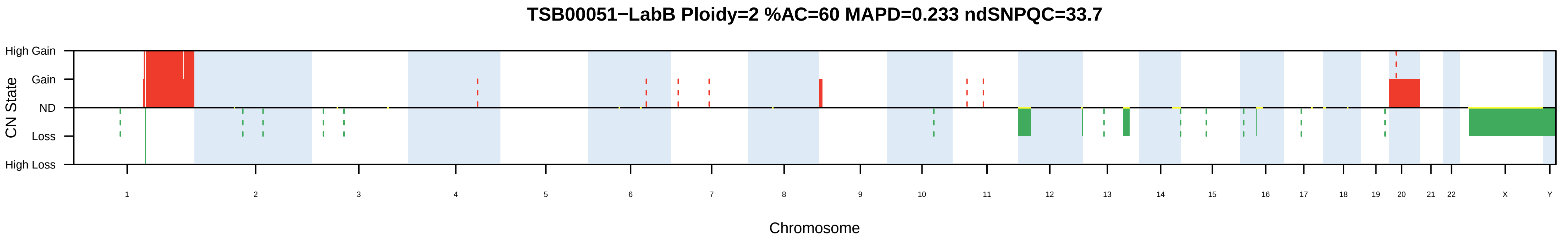
TSB00051−LabB Ploidy=2 %AC=60 MAPD=0.233 ndSNPQC=33.7
High Gain
Gain
ND
Loss
High Loss
CN State
1 2 3 4 5 6 7 8 9 10 11 12 13 14 15 16 17 18 19 20 21 22 X Y
Chromosome

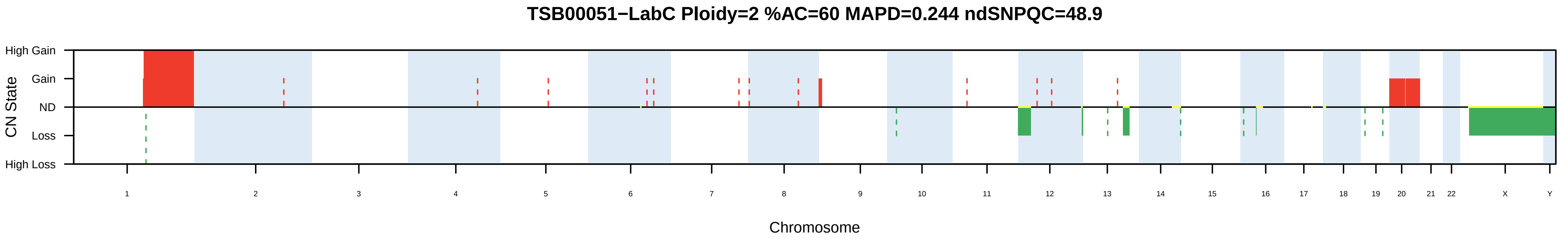
TSB00051−LabC Ploidy=2 %AC=60 MAPD=0.244 ndSNPQC=48.9
High Gain
Gain
ND
Loss
High Loss
CN State
1 2 3 4 5 6 7 8 9 10 11 12 13 14 15 16 17 18 19 20 21 22 X Y
Chromosome

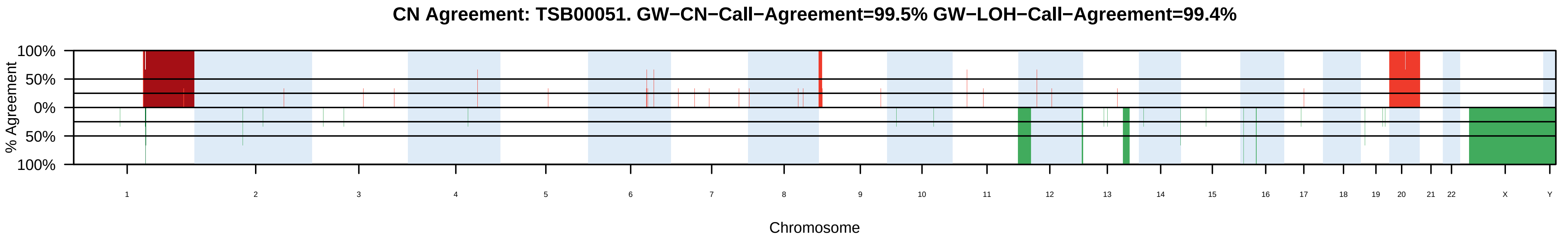
CN Agreement: TSB00051. GW−CN−Call−Agreement=99.5% GW−LOH−Call−Agreement=99.4%
100%
50%
0%
50%
100%
% Agreement
1 2 3 4 5 6 7 8 9 10 11 12 13 14 15 16 17 18 19 20 21 22 X Y
Chromosome

TSB00052−LabA Ploidy=2 %AC=50 MAPD=0.258 ndSNPQC=40.1

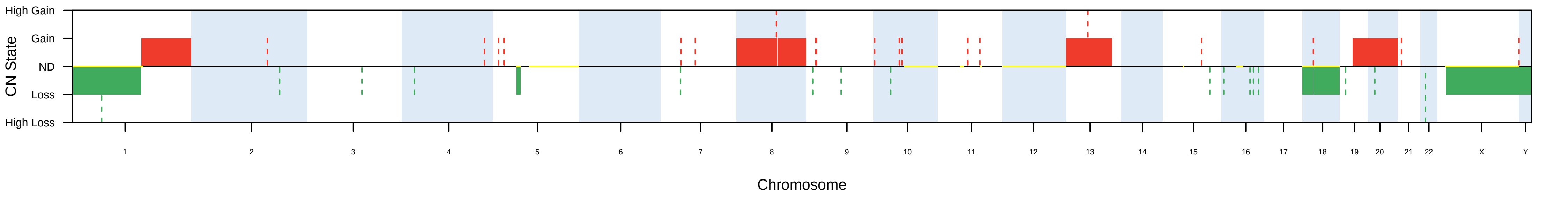

TSB00052−LabB Ploidy=2 %AC=50 MAPD=0.266 ndSNPQC=30.2

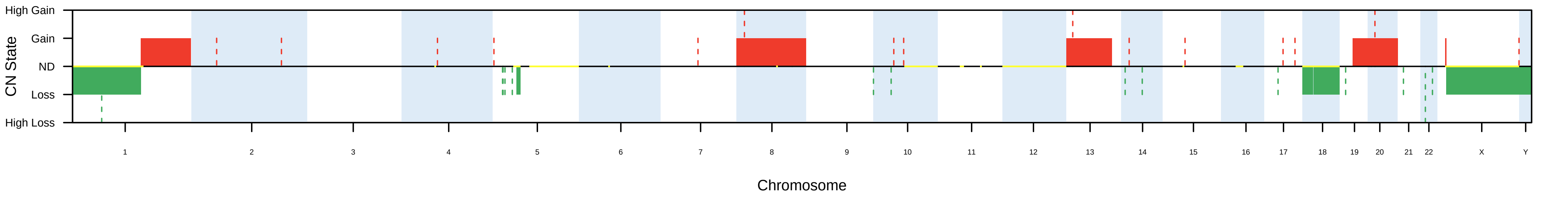

TSB00052−LabC Ploidy=2 %AC=50 MAPD=0.271 ndSNPQC=38.5

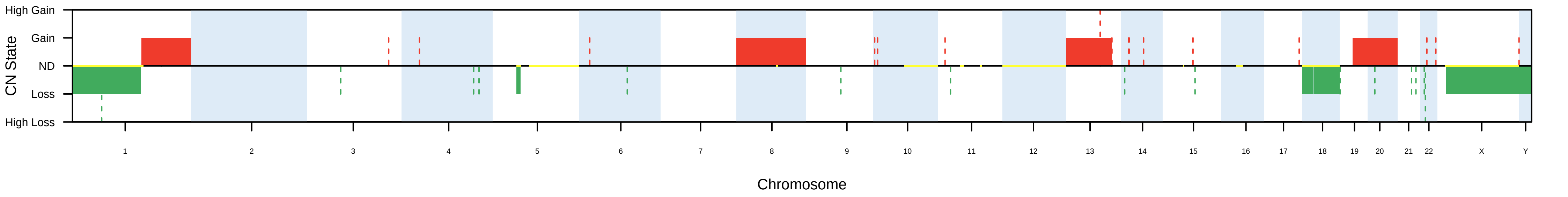

CN Agreement: TSB00052. GW−CN−Call−Agreement=98.8% GW−LOH−Call−Agreement=99.4%

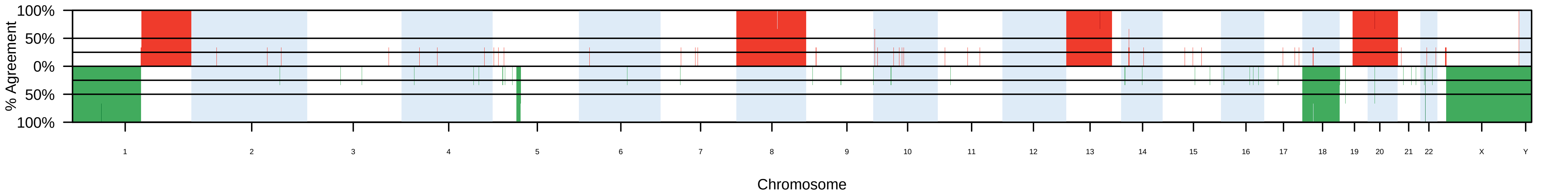

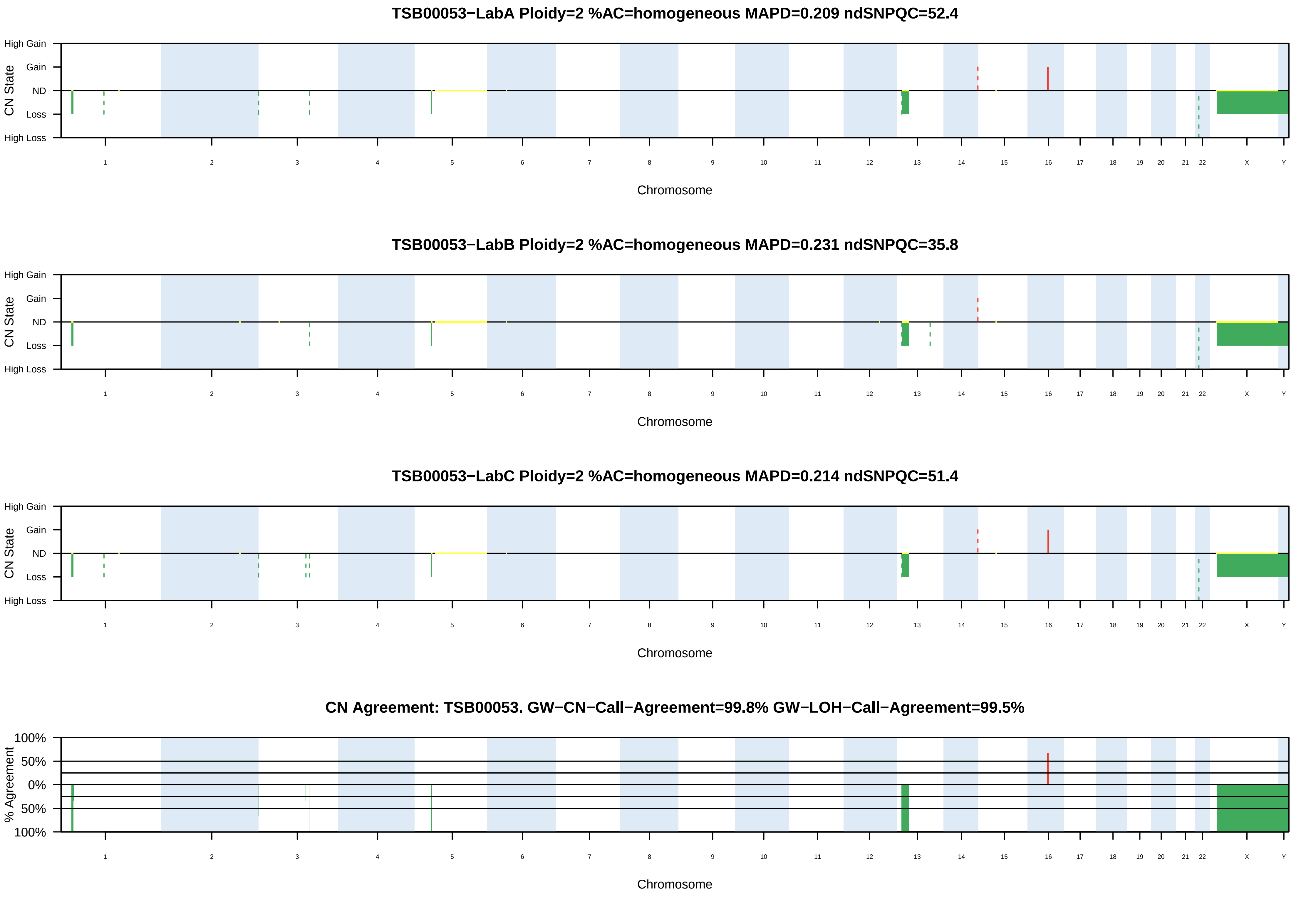
TSB00053−LabA Ploidy=2 %AC=homogeneous MAPD=0.209 ndSNPQC=52.4
TSB00053−LabB Ploidy=2 %AC=homogeneous MAPD=0.231 ndSNPQC=35.8
TSB00053−LabC Ploidy=2 %AC=homogeneous MAPD=0.214 ndSNPQC=51.4
CN State
High Gain
Gain
ND
Loss
High Loss
Chromosome
1 2 3 4 5 6 7 8 9 10 11 12 13 14 15 16 17 18 19 20 21 22 X Y
CN Agreement: TSB00053. GW−CN−Call−Agreement=99.8% GW−LOH−Call−Agreement=99.5%
% Agreement
100%
50%
0%
50%
100%

TSB00054−LabA Ploidy=2 %AC=50 MAPD=0.195 ndSNPQC=56.5

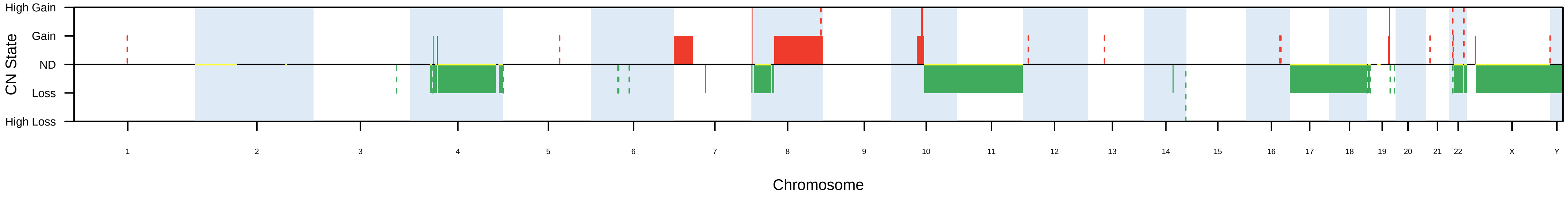

TSB00054−LabB Ploidy=2 %AC=50 MAPD=0.222 ndSNPQC=41.2

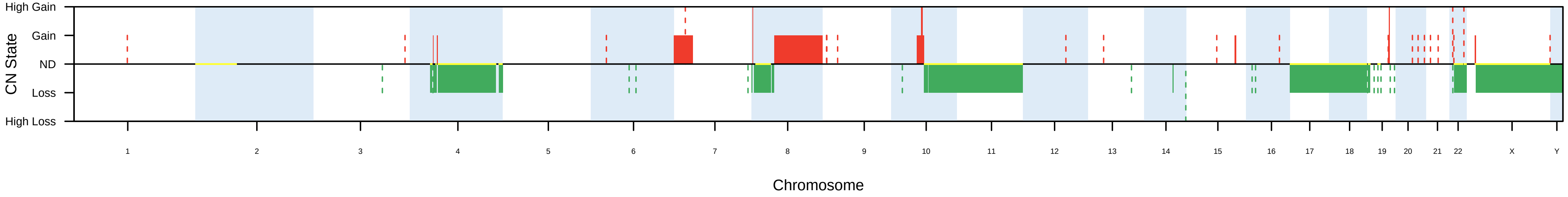

TSB00054−LabC Ploidy=2 %AC=50 MAPD=0.206 ndSNPQC=57.9

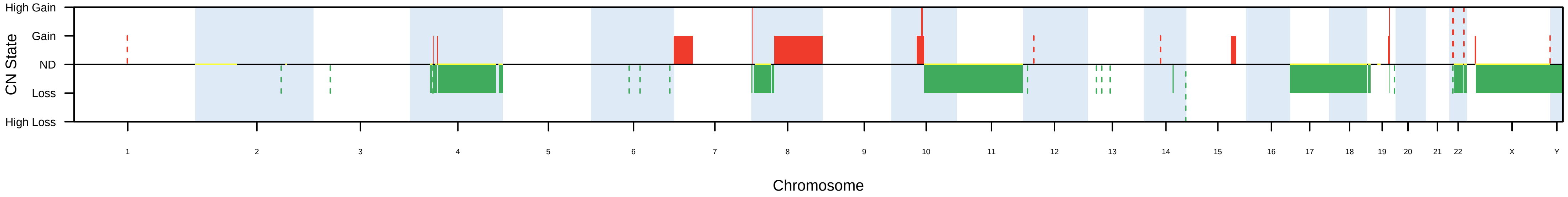

CN Agreement: TSB00054. GW−CN−Call−Agreement=98.7% GW−LOH−Call−Agreement=99.8%

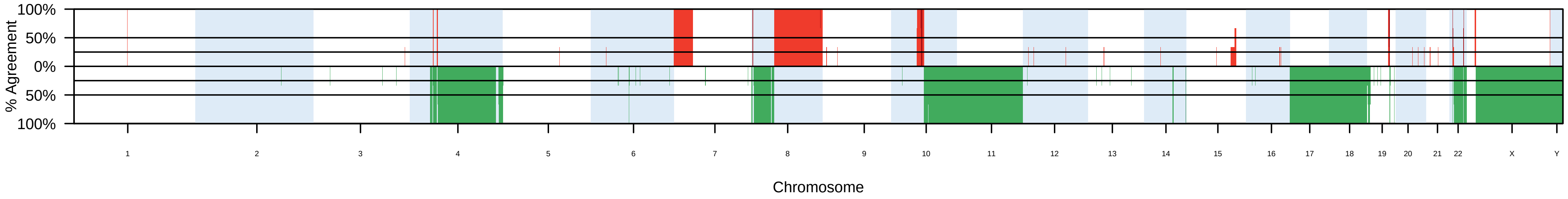

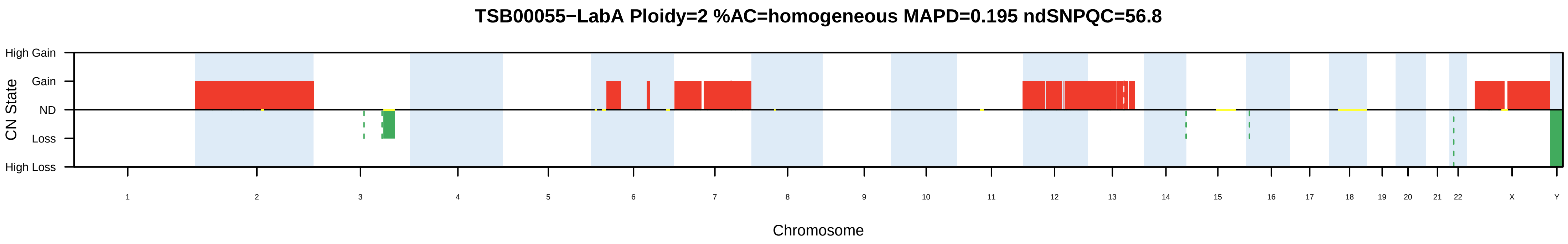
TSB00055−LabA Ploidy=2 %AC=homogeneous MAPD=0.195 ndSNPQC=56.8
High Gain
Gain
ND
Loss
High Loss
CN State
1 2 3 4 5 6 7 8 9 10 11 12 13 14 15 16 17 18 19 20 21 22 X Y
Chromosome

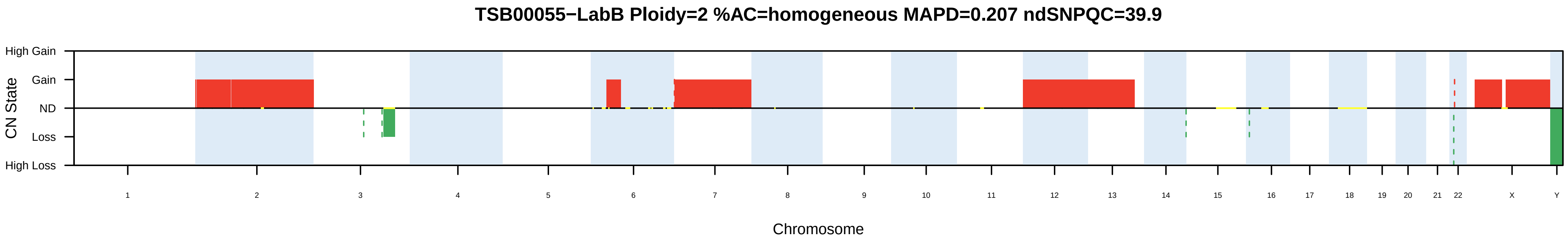
TSB00055−LabB Ploidy=2 %AC=homogeneous MAPD=0.207 ndSNPQC=39.9
High Gain
Gain
ND
Loss
High Loss
CN State
1 2 3 4 5 6 7 8 9 10 11 12 13 14 15 16 17 18 19 20 21 22 X Y
Chromosome

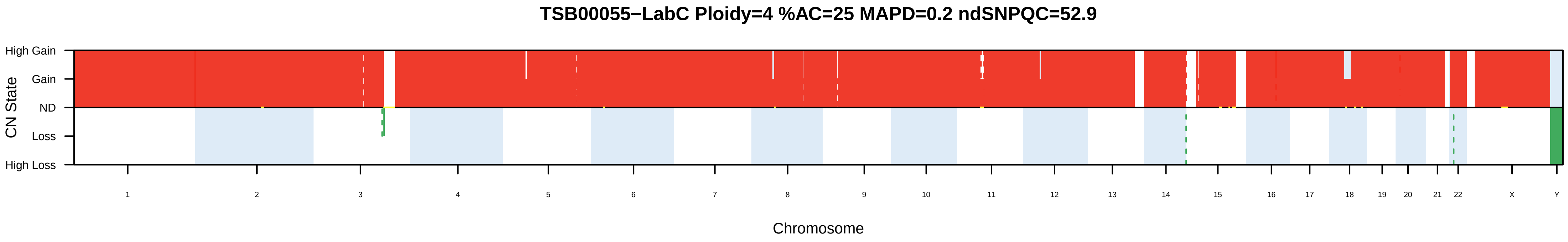
TSB00055−LabC Ploidy=4 %AC=25 MAPD=0.2 ndSNPQC=52.9
High Gain
Gain
ND
Loss
High Loss
CN State
1 2 3 4 5 6 7 8 9 10 11 12 13 14 15 16 17 18 19 20 21 22 X Y
Chromosome

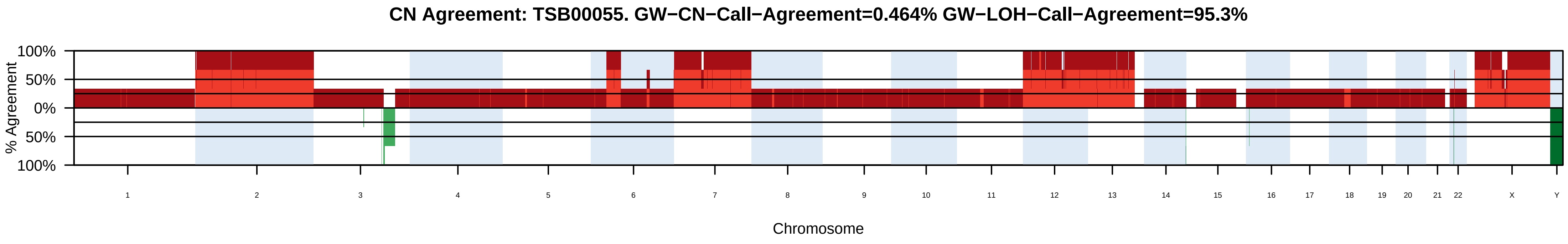
CN Agreement: TSB00055. GW−CN−Call−Agreement=0.464% GW−LOH−Call−Agreement=95.3%
100%
50%
0%
50%
100%
% Agreement
1 2 3 4 5 6 7 8 9 10 11 12 13 14 15 16 17 18 19 20 21 22 X Y
Chromosome

TSB00056−LabA Ploidy=4 %AC=50 MAPD=0.206 ndSNPQC=48.4

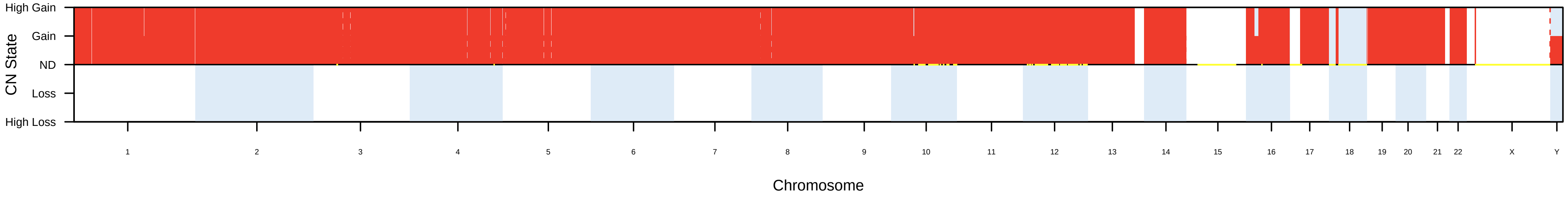

TSB00056−LabB Ploidy=4 %AC=55 MAPD=0.215 ndSNPQC=39.1

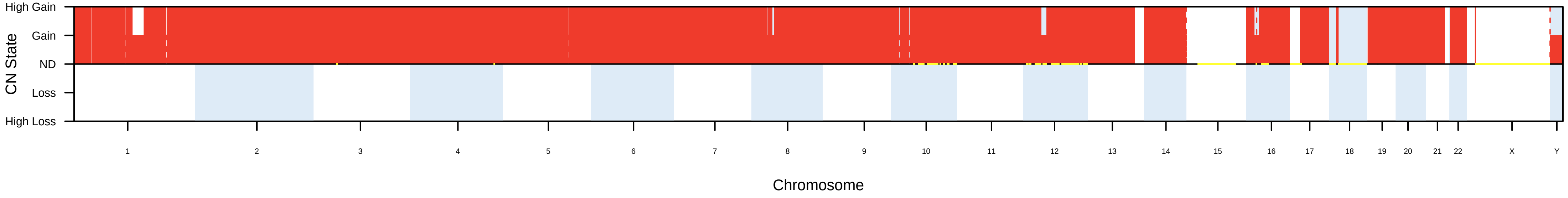

TSB00056−LabC Ploidy=4 %AC=55 MAPD=0.203 ndSNPQC=45.9

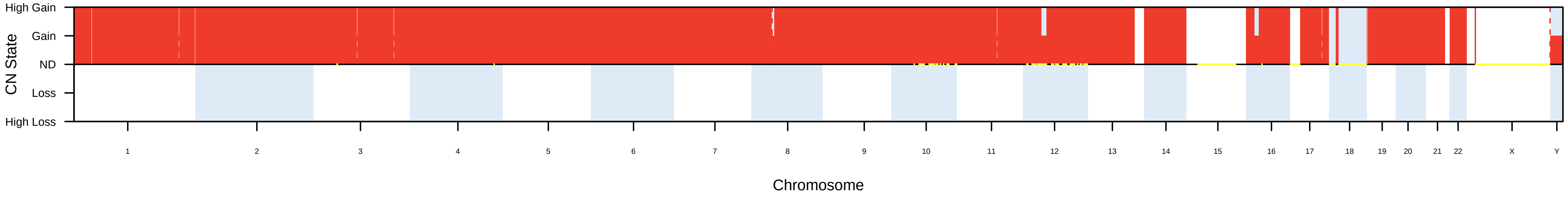

CN Agreement: TSB00056. GW−CN−Call−Agreement=98.1% GW−LOH−Call−Agreement=97.6%

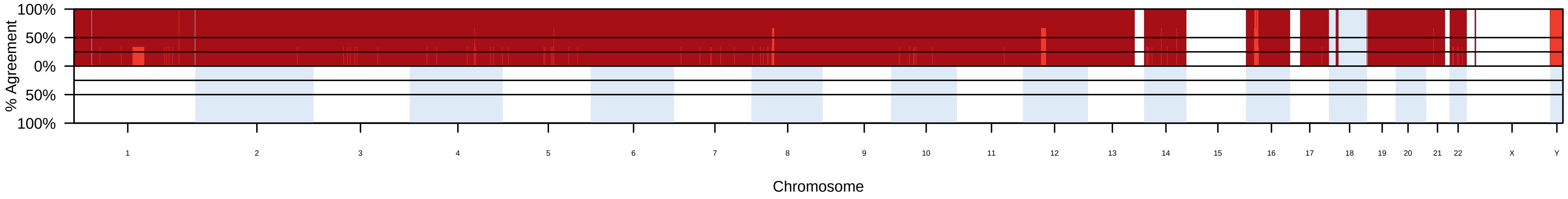

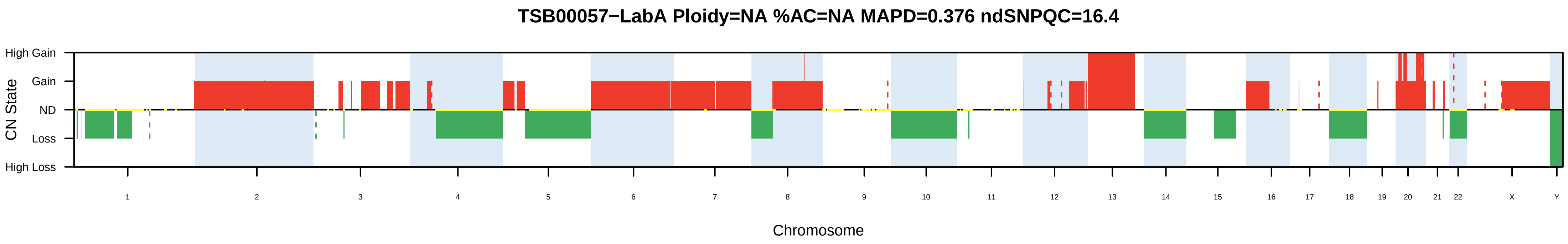
TSB00057−LabA Ploidy=NA %AC=NA MAPD=0.376 ndSNPQC=16.4
CN State
High Gain
Gain
ND
Loss
High Loss
1 2 3 4 5 6 7 8 9 10 11 12 13 14 15 16 17 18 19 20 21 22 X Y
Chromosome

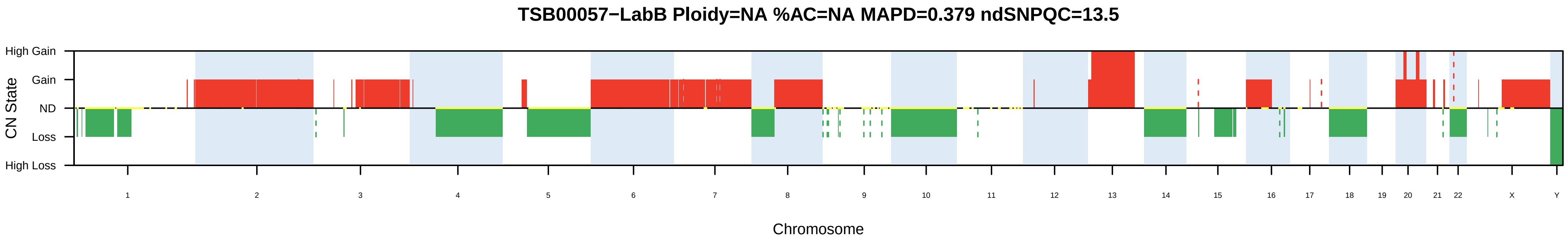
TSB00057−LabB Ploidy=NA %AC=NA MAPD=0.379 ndSNPQC=13.5
CN State
High Gain
Gain
ND
Loss
High Loss
1 2 3 4 5 6 7 8 9 10 11 12 13 14 15 16 17 18 19 20 21 22 X Y
Chromosome

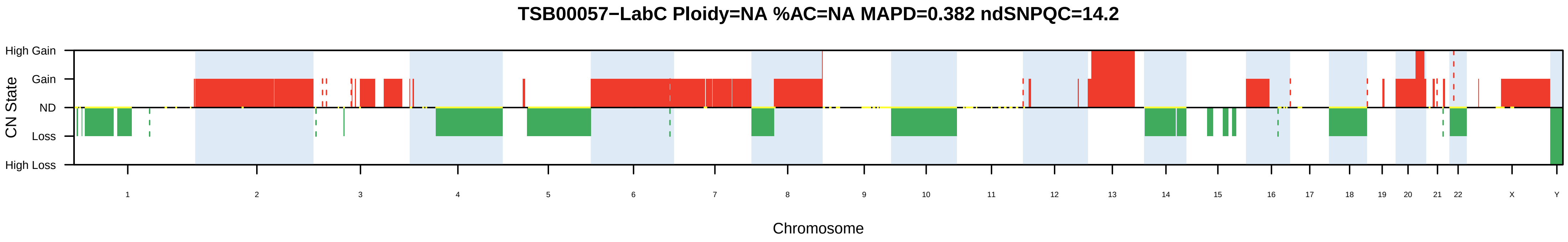
TSB00057−LabC Ploidy=NA %AC=NA MAPD=0.382 ndSNPQC=14.2
CN State
High Gain
Gain
ND
Loss
High Loss
1 2 3 4 5 6 7 8 9 10 11 12 13 14 15 16 17 18 19 20 21 22 X Y
Chromosome

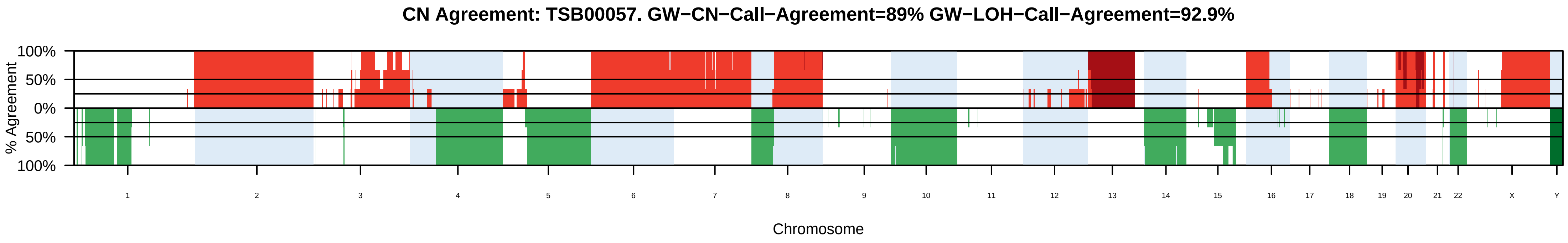
CN Agreement: TSB00057. GW−CN−Call−Agreement=89% GW−LOH−Call−Agreement=92.9%
% Agreement
100%
50%
0%
50%
100%
1 2 3 4 5 6 7 8 9 10 11 12 13 14 15 16 17 18 19 20 21 22 X Y
Chromosome

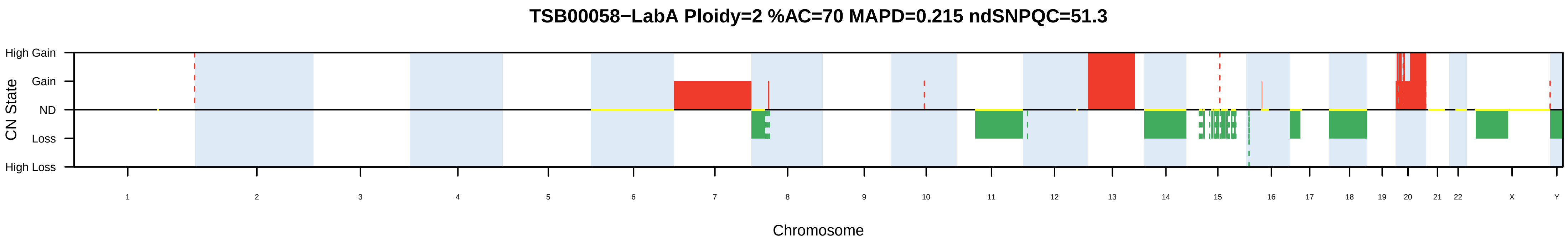
TSB00058−LabA Ploidy=2 %AC=70 MAPD=0.215 ndSNPQC=51.3
High Gain
Gain
ND
Loss
High Loss
CN State
1 2 3 4 5 6 7 8 9 10 11 12 13 14 15 16 17 18 19 20 21 22 X Y
Chromosome

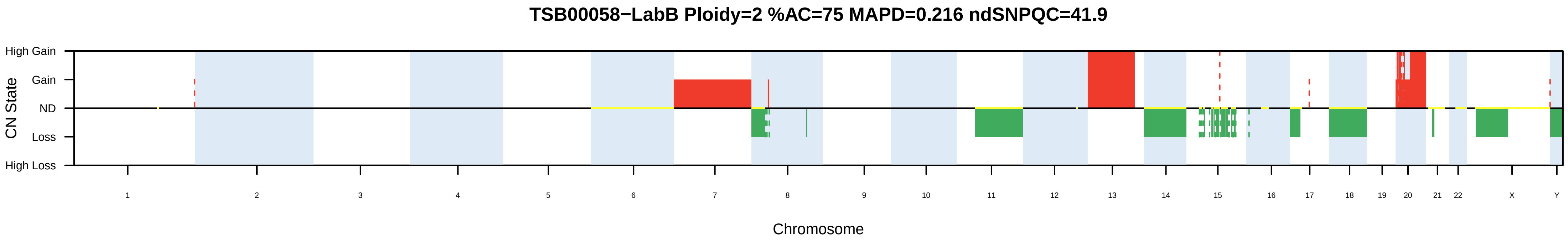
TSB00058−LabB Ploidy=2 %AC=75 MAPD=0.216 ndSNPQC=41.9
High Gain
Gain
ND
Loss
High Loss
CN State
1 2 3 4 5 6 7 8 9 10 11 12 13 14 15 16 17 18 19 20 21 22 X Y
Chromosome

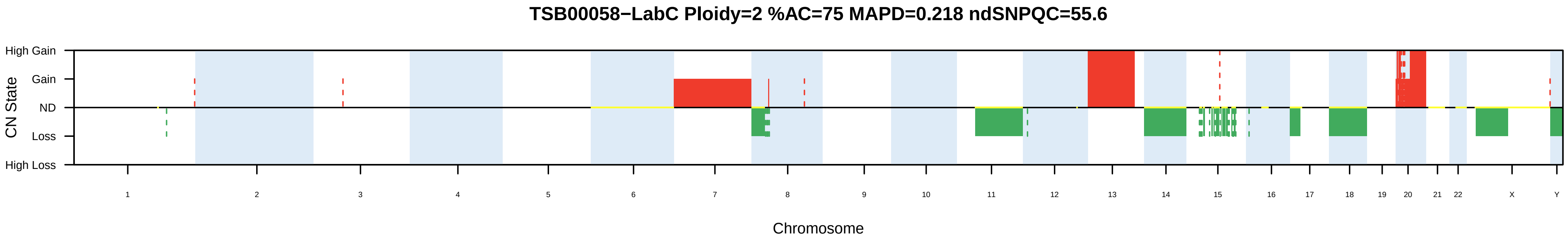
TSB00058−LabC Ploidy=2 %AC=75 MAPD=0.218 ndSNPQC=55.6
High Gain
Gain
ND
Loss
High Loss
CN State
1 2 3 4 5 6 7 8 9 10 11 12 13 14 15 16 17 18 19 20 21 22 X Y
Chromosome

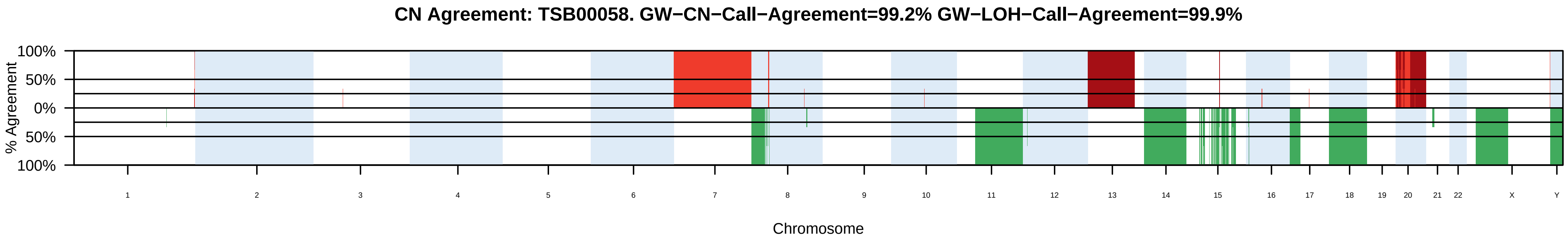
CN Agreement: TSB00058. GW−CN−Call−Agreement=99.2% GW−LOH−Call−Agreement=99.9%
100%
50%
0%
50%
100%
% Agreement
1 2 3 4 5 6 7 8 9 10 11 12 13 14 15 16 17 18 19 20 21 22 X Y
Chromosome

TSB00059−LabA Ploidy=NA %AC=NA MAPD=0.4 ndSNPQC=12.6

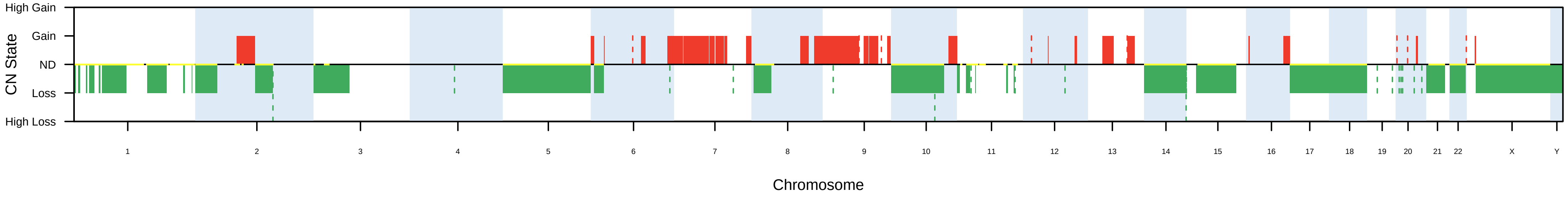

TSB00059−LabB Ploidy=NA %AC=NA MAPD=0.399 ndSNPQC=11.8

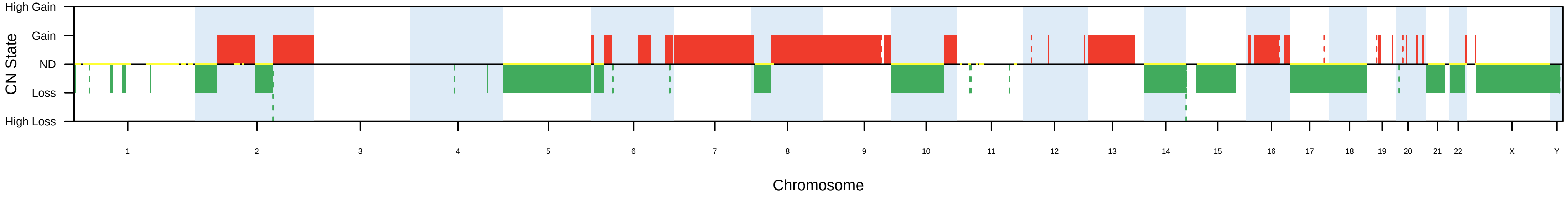

TSB00059−LabC Ploidy=NA %AC=NA MAPD=0.398 ndSNPQC=13.9

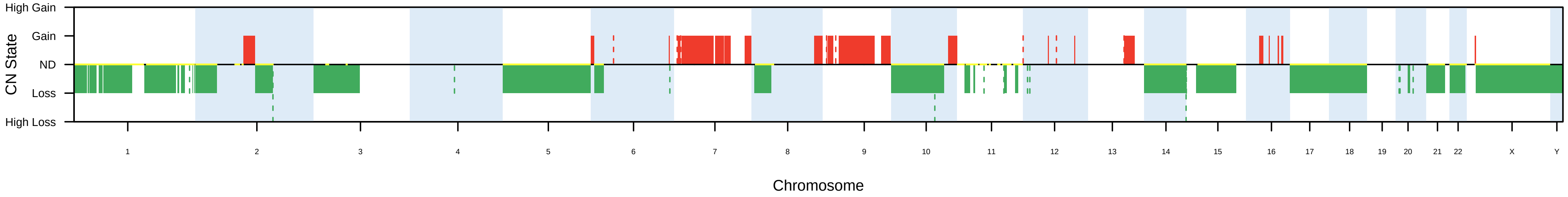

CN Agreement: TSB00059. GW−CN−Call−Agreement=67.7% GW−LOH−Call−Agreement=95.1%

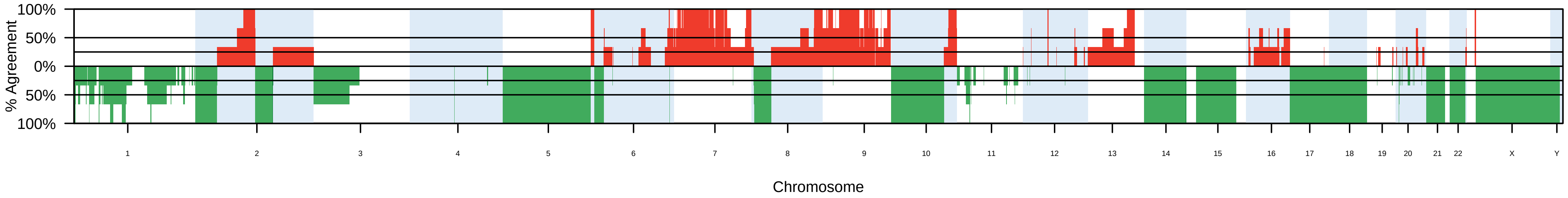

TSB00060−LabA Ploidy=2 %AC=45 MAPD=0.214 ndSNPQC=54

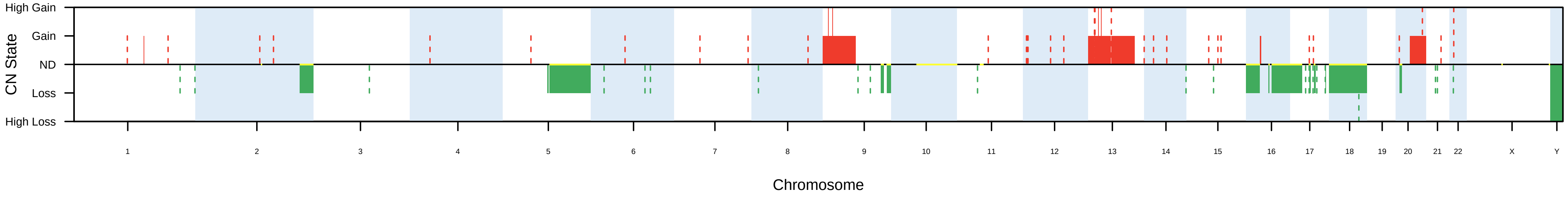

TSB00060−LabB Ploidy=2 %AC=45 MAPD=0.2 ndSNPQC=44.7

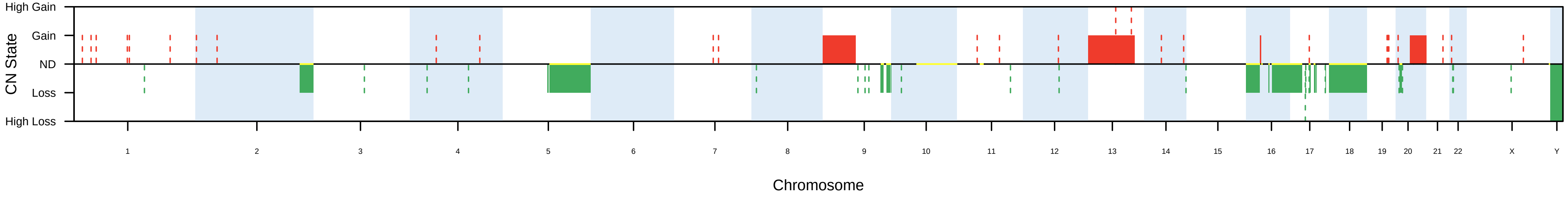

TSB00060−LabC Ploidy=2 %AC=45 MAPD=0.2 ndSNPQC=57

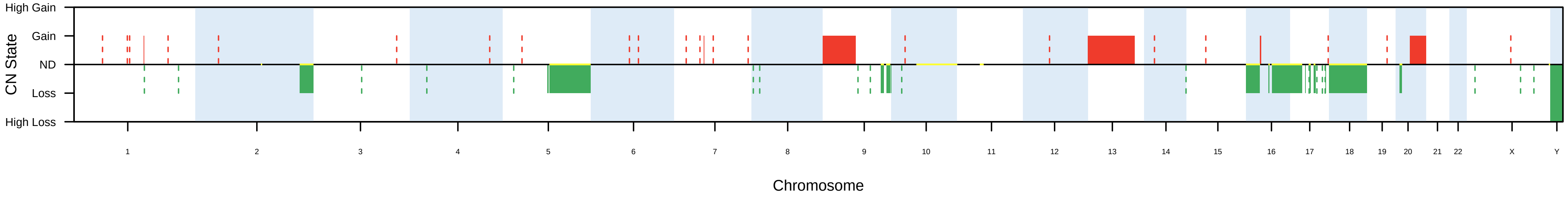

CN Agreement: TSB00060. GW−CN−Call−Agreement=98.4% GW−LOH−Call−Agreement=99.6%

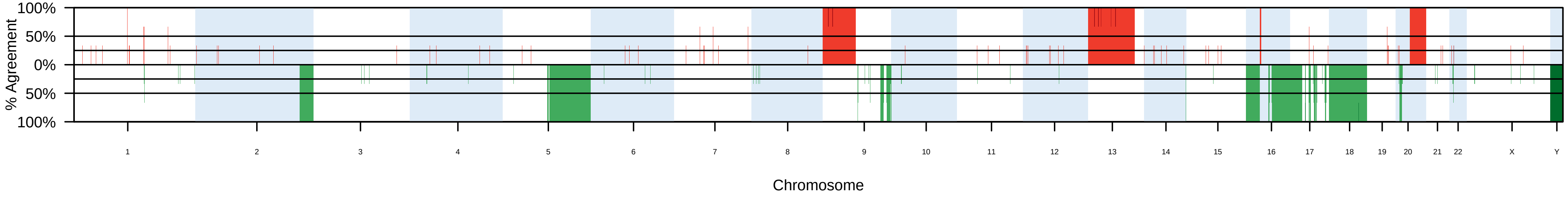

TSB00061−LabA Ploidy=2 %AC=65 MAPD=0.249 ndSNPQC=44.8

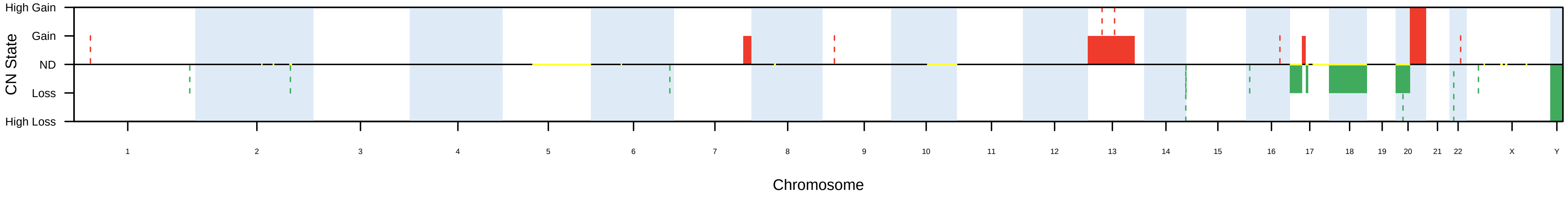

TSB00061−LabB Ploidy=2 %AC=65 MAPD=0.248 ndSNPQC=34.5

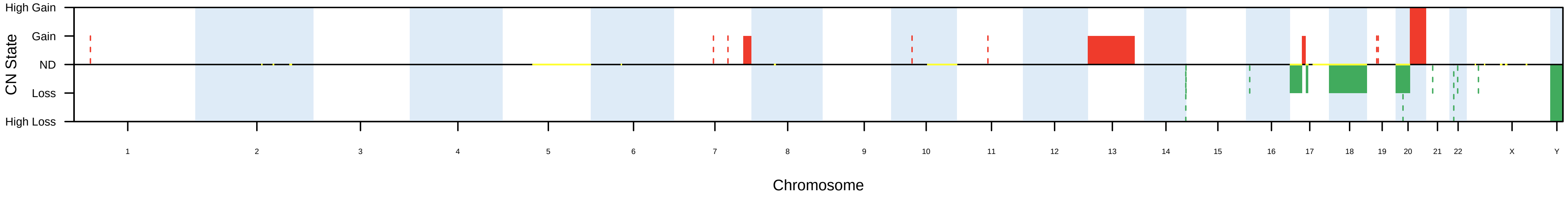

TSB00061−LabC Ploidy=2 %AC=65 MAPD=0.254 ndSNPQC=44

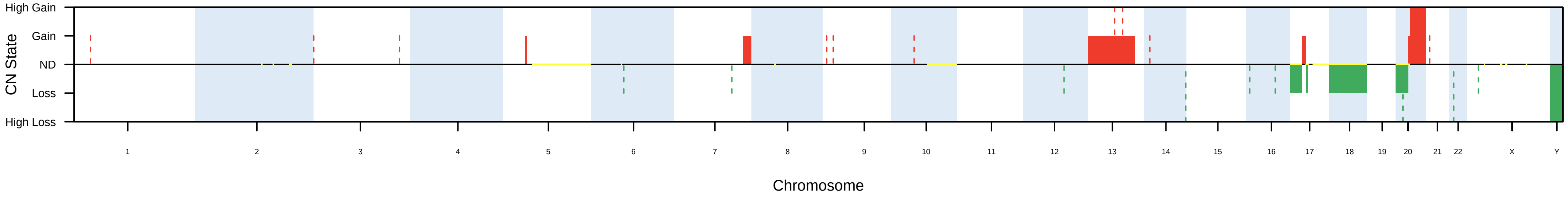

CN Agreement: TSB00061. GW−CN−Call−Agreement=99.5% GW−LOH−Call−Agreement=99.9%

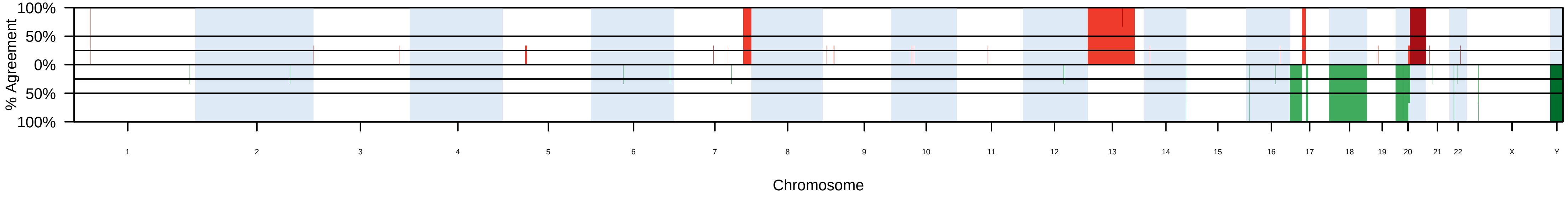

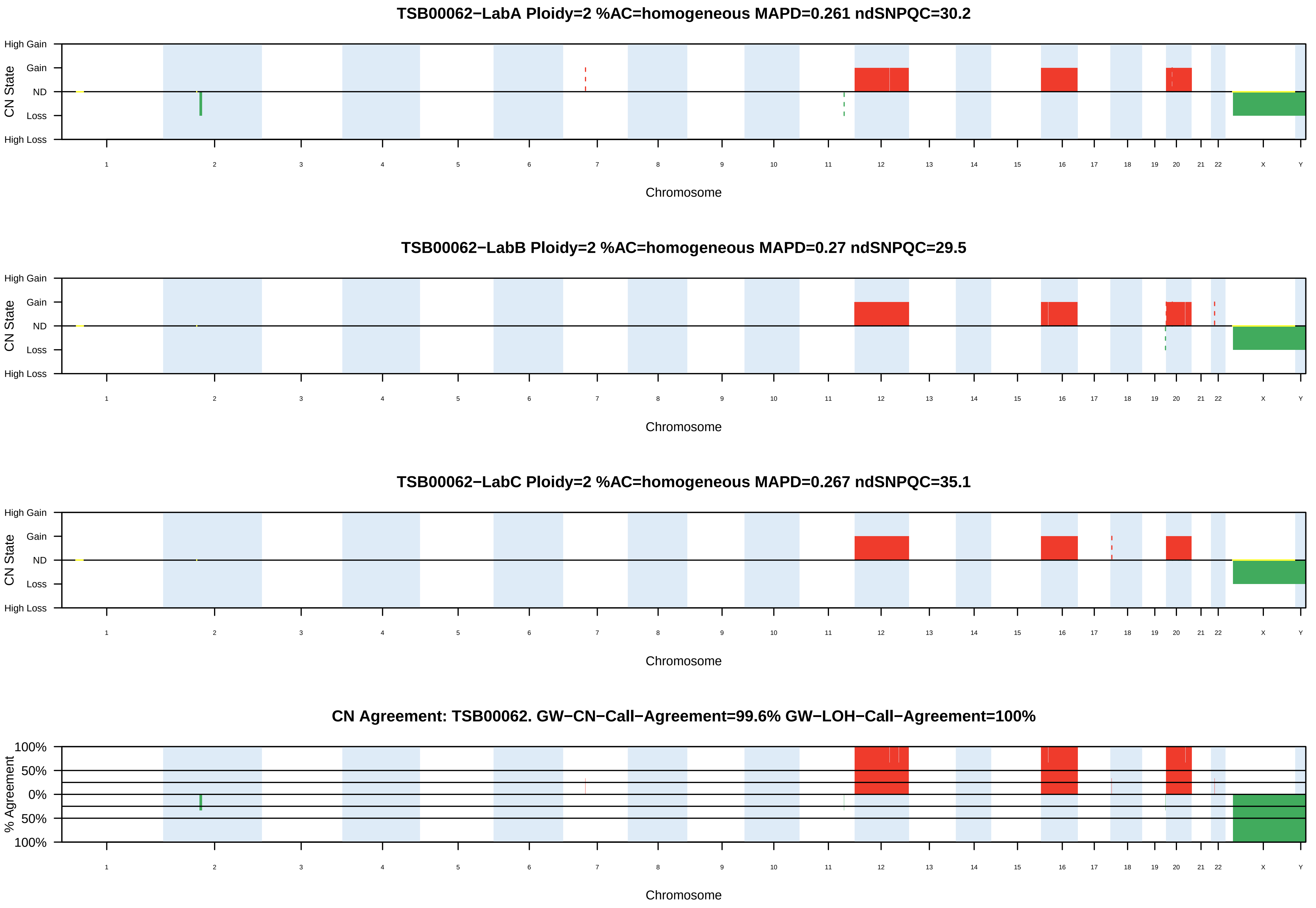
TSB00062−LabA Ploidy=2 %AC=homogeneous MAPD=0.261 ndSNPQC=30.2
TSB00062−LabB Ploidy=2 %AC=homogeneous MAPD=0.27 ndSNPQC=29.5
TSB00062−LabC Ploidy=2 %AC=homogeneous MAPD=0.267 ndSNPQC=35.1
CN Agreement: TSB00062. GW−CN−Call−Agreement=99.6% GW−LOH−Call−Agreement=100%
CN State
High Gain
Gain
ND
Loss
High Loss
% Agreement
100%
50%
0%
50%
100%
Chromosome
1 2 3 4 5 6 7 8 9 10 11 12 13 14 15 16 17 18 19 20 21 22 X Y

TSB00063−LabA Ploidy=2 %AC=homogeneous MAPD=0.262 ndSNPQC=40.6

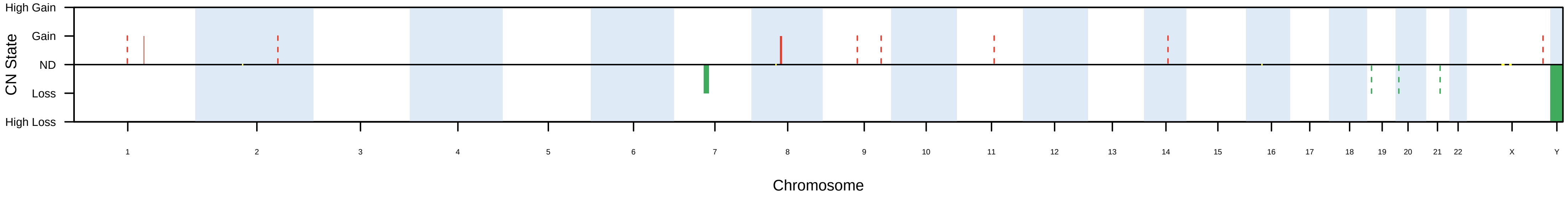

TSB00063−LabB Ploidy=2 %AC=homogeneous MAPD=0.247 ndSNPQC=32.6

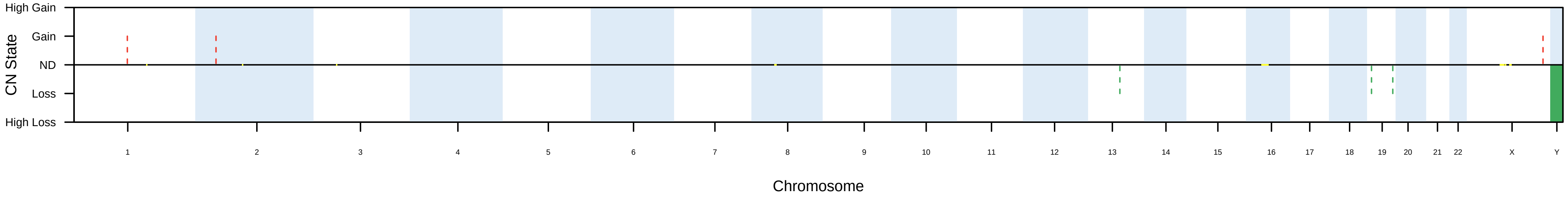

TSB00063−LabC Ploidy=2 %AC=homogeneous MAPD=0.257 ndSNPQC=43.6

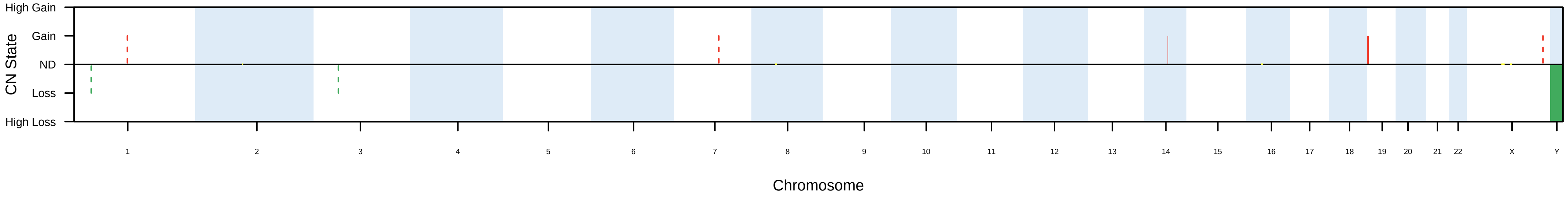

CN Agreement: TSB00063. GW−CN−Call−Agreement=99.2% GW−LOH−Call−Agreement=99.1%

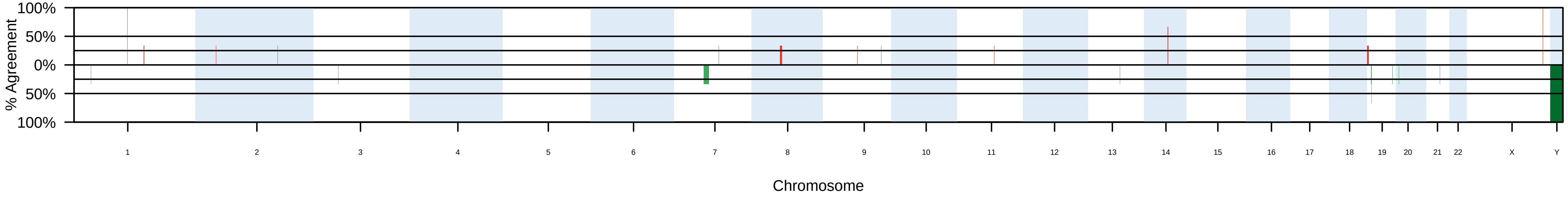

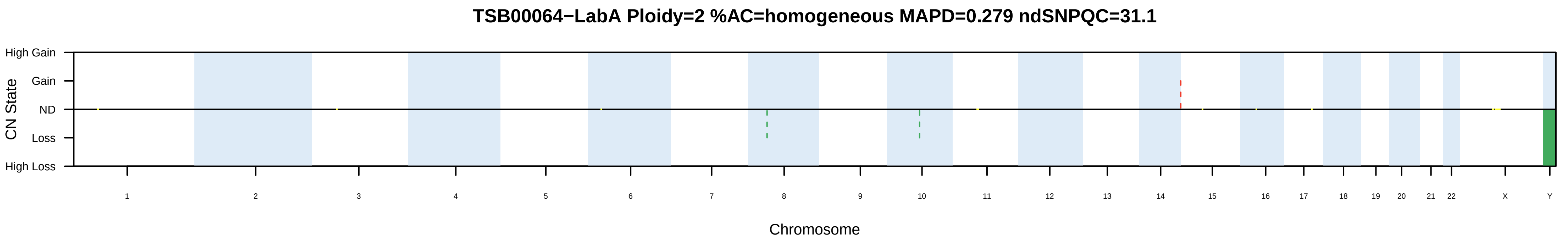

TSB00064−LabA Ploidy=2 %AC=homogeneous MAPD=0.279 ndSNPQC=31.1
High Gain
Gain
ND
Loss
High Loss
CN State
1 2 3 4 5 6 7 8 9 10 11 12 13 14 15 16 17 18 19 20 21 22 X Y
Chromosome

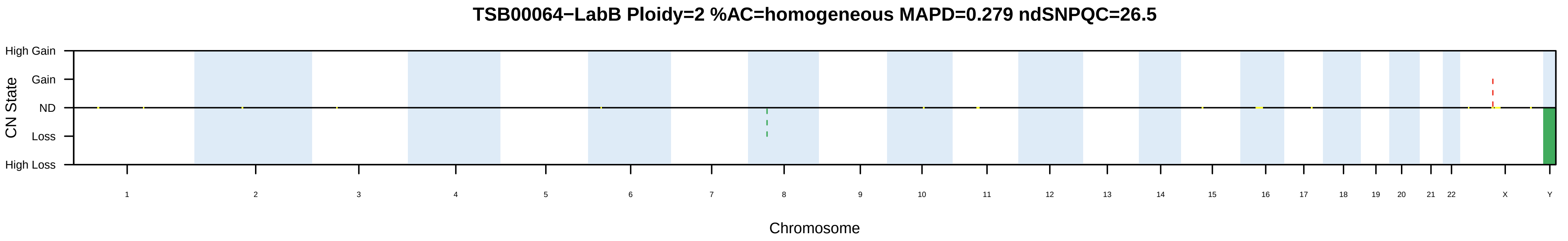

TSB00064−LabB Ploidy=2 %AC=homogeneous MAPD=0.279 ndSNPQC=26.5
High Gain
Gain
ND
Loss
High Loss
CN State
1 2 3 4 5 6 7 8 9 10 11 12 13 14 15 16 17 18 19 20 21 22 X Y
Chromosome

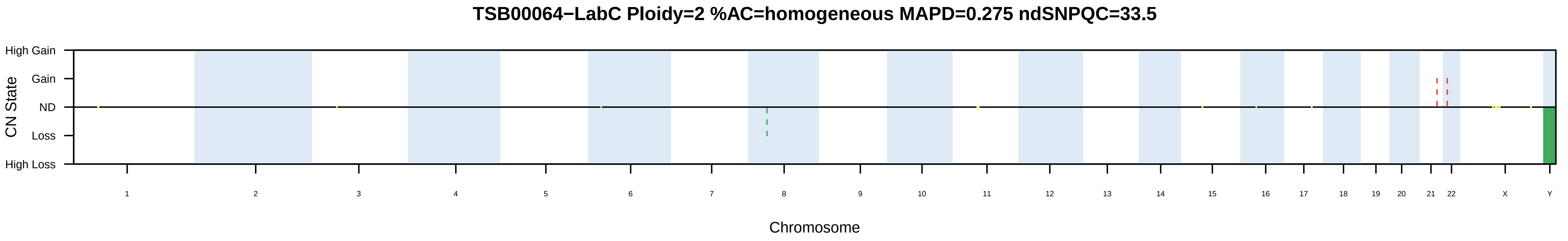

TSB00064−LabC Ploidy=2 %AC=homogeneous MAPD=0.275 ndSNPQC=33.5
High Gain
Gain
ND
Loss
High Loss
CN State
1 2 3 4 5 6 7 8 9 10 11 12 13 14 15 16 17 18 19 20 21 22 X Y
Chromosome

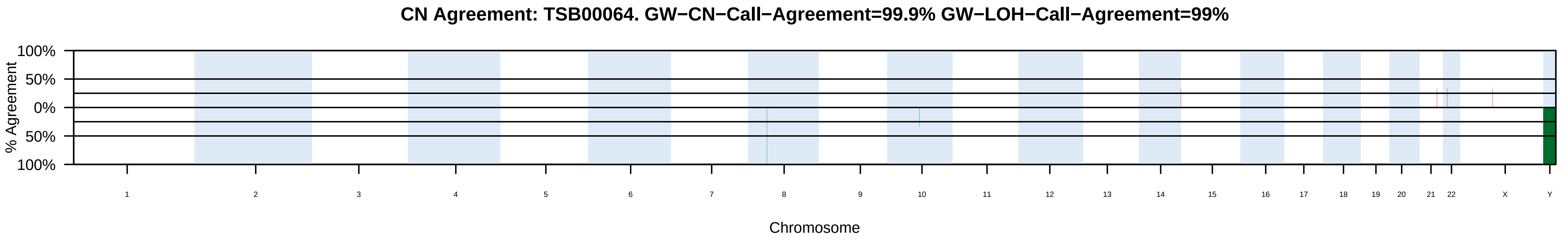

CN Agreement: TSB00064. GW−CN−Call−Agreement=99.9% GW−LOH−Call−Agreement=99%
100%
50%
0%
50%
100%
% Agreement
1 2 3 4 5 6 7 8 9 10 11 12 13 14 15 16 17 18 19 20 21 22 X Y
Chromosome

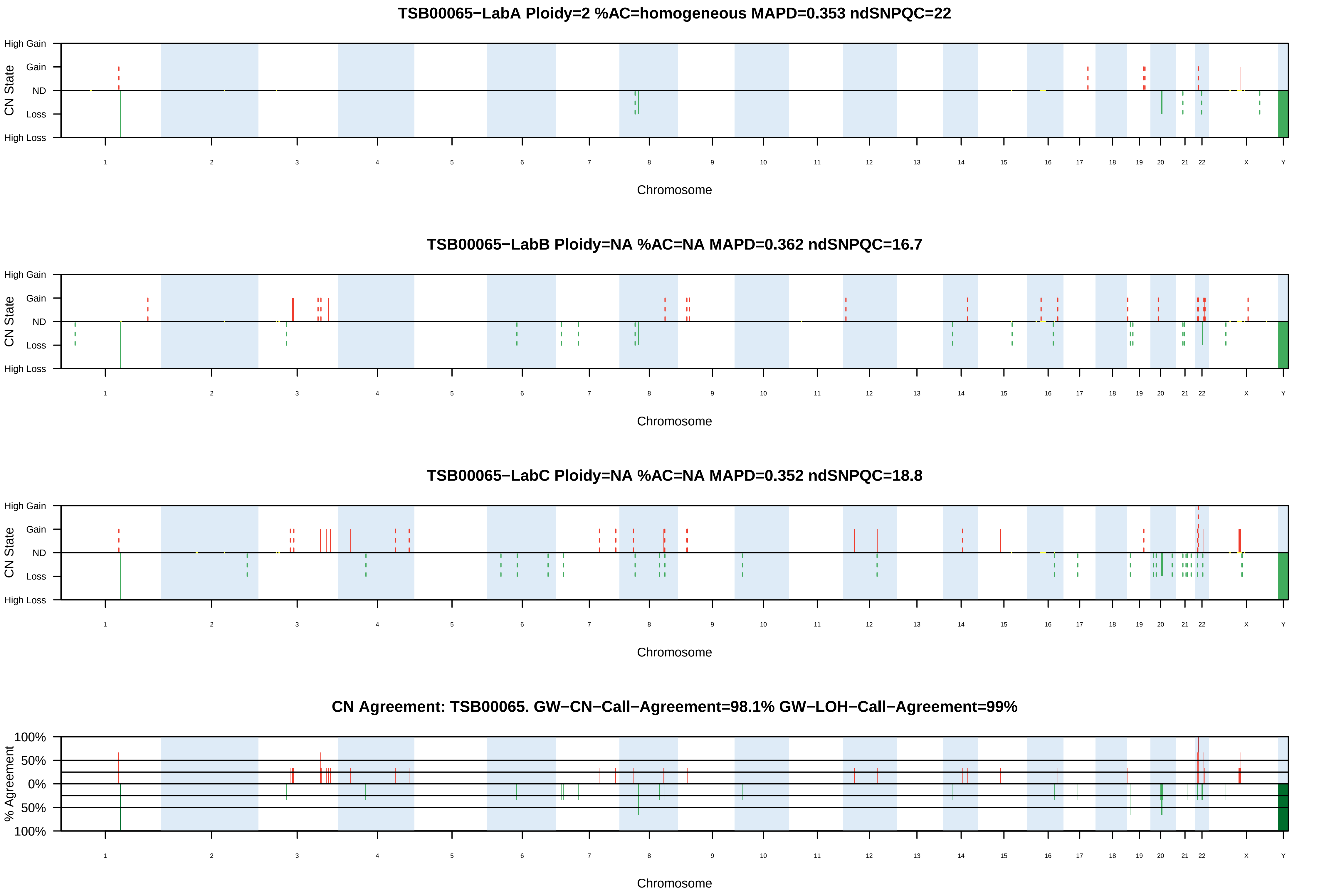
TSB00065−LabA Ploidy=2 %AC=homogeneous MAPD=0.353 ndSNPQC=22
TSB00065−LabB Ploidy=NA %AC=NA MAPD=0.362 ndSNPQC=16.7
TSB00065−LabC Ploidy=NA %AC=NA MAPD=0.352 ndSNPQC=18.8
CN State
High Gain
Gain
ND
Loss
High Loss
Chromosome
1 2 3 4 5 6 7 8 9 10 11 12 13 14 15 16 17 18 19 20 21 22 X Y
CN Agreement: TSB00065. GW−CN−Call−Agreement=98.1% GW−LOH−Call−Agreement=99%
% Agreement
100%
50%
0%
50%
100%

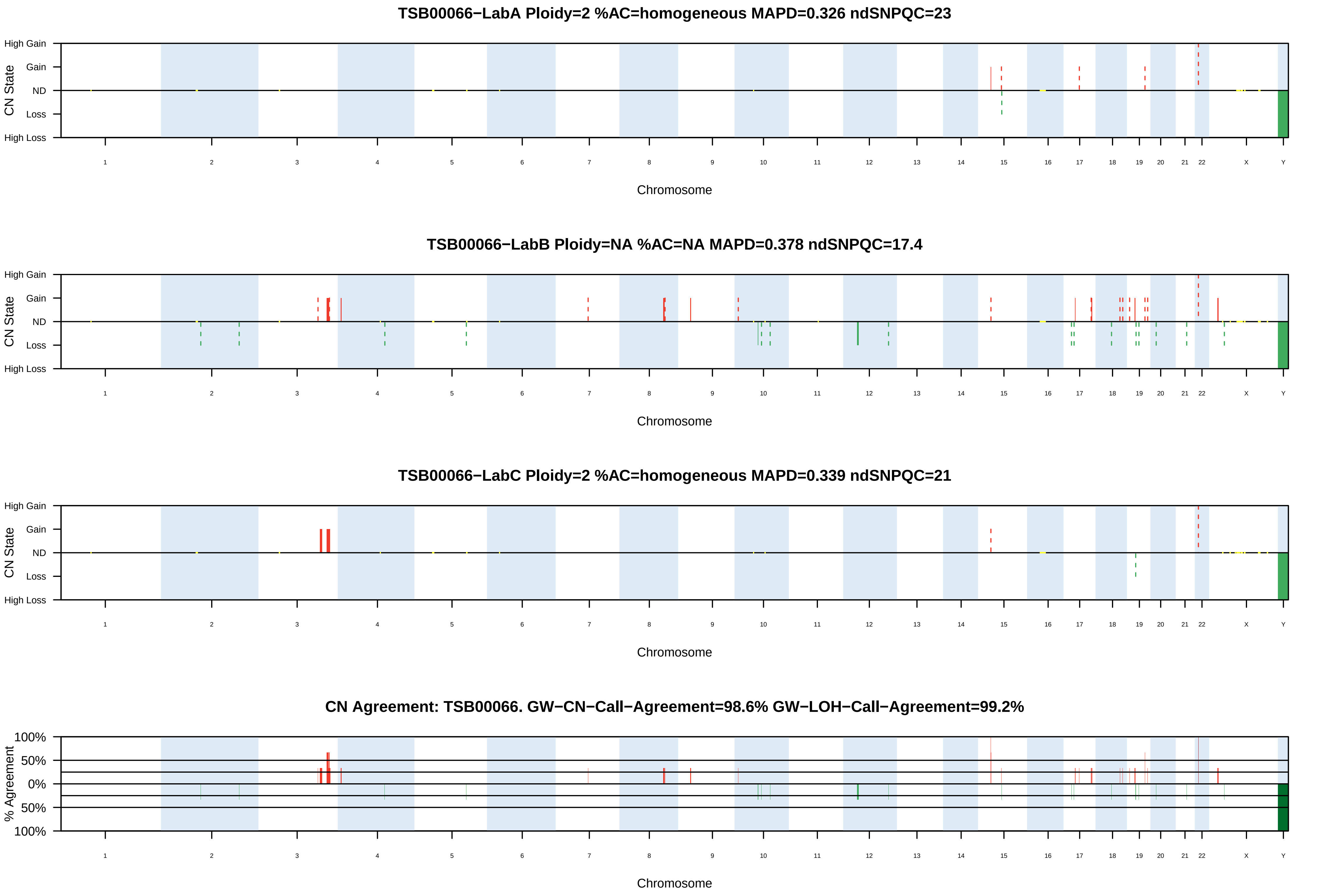
TSB00066−LabA Ploidy=2 %AC=homogeneous MAPD=0.326 ndSNPQC=23
High Gain
Gain
ND
Loss
High Loss
CN State
1 2 3 4 5 6 7 8 9 10 11 12 13 14 15 16 17 18 19 20 21 22 X Y
Chromosome
TSB00066−LabB Ploidy=NA %AC=NA MAPD=0.378 ndSNPQC=17.4
High Gain
Gain
ND
Loss
High Loss
CN State
1 2 3 4 5 6 7 8 9 10 11 12 13 14 15 16 17 18 19 20 21 22 X Y
Chromosome
TSB00066−LabC Ploidy=2 %AC=homogeneous MAPD=0.339 ndSNPQC=21
High Gain
Gain
ND
Loss
High Loss
CN State
1 2 3 4 5 6 7 8 9 10 11 12 13 14 15 16 17 18 19 20 21 22 X Y
Chromosome
CN Agreement: TSB00066. GW−CN−Call−Agreement=98.6% GW−LOH−Call−Agreement=99.2%
100%
50%
0%
50%
100%
% Agreement
1 2 3 4 5 6 7 8 9 10 11 12 13 14 15 16 17 18 19 20 21 22 X Y
Chromosome

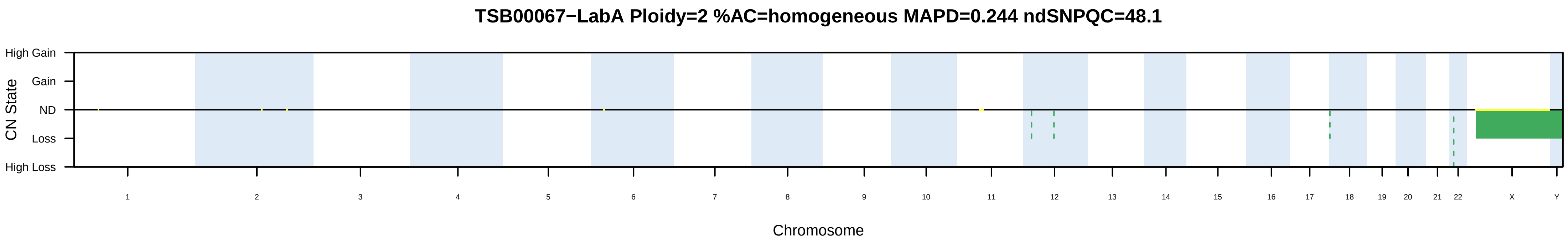
TSB00067−LabA Ploidy=2 %AC=homogeneous MAPD=0.244 ndSNPQC=48.1
CN State
High Gain
Gain
ND
Loss
High Loss
1 2 3 4 5 6 7 8 9 10 11 12 13 14 15 16 17 18 19 20 21 22 X Y
Chromosome

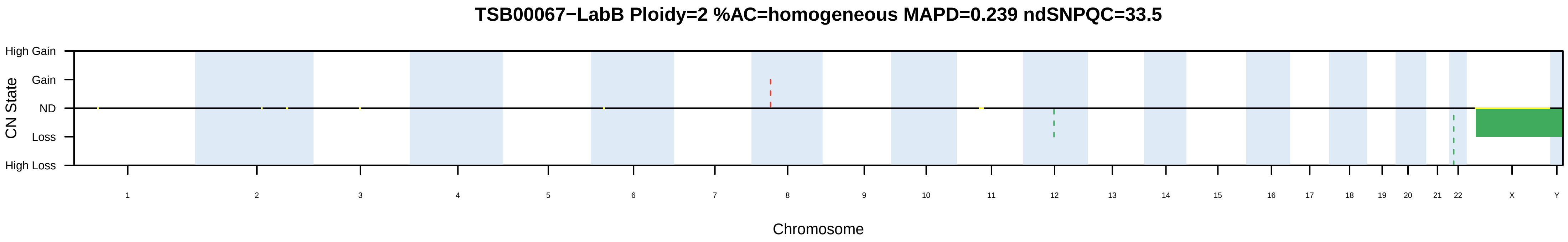
TSB00067−LabB Ploidy=2 %AC=homogeneous MAPD=0.239 ndSNPQC=33.5
CN State
High Gain
Gain
ND
Loss
High Loss
1 2 3 4 5 6 7 8 9 10 11 12 13 14 15 16 17 18 19 20 21 22 X Y
Chromosome

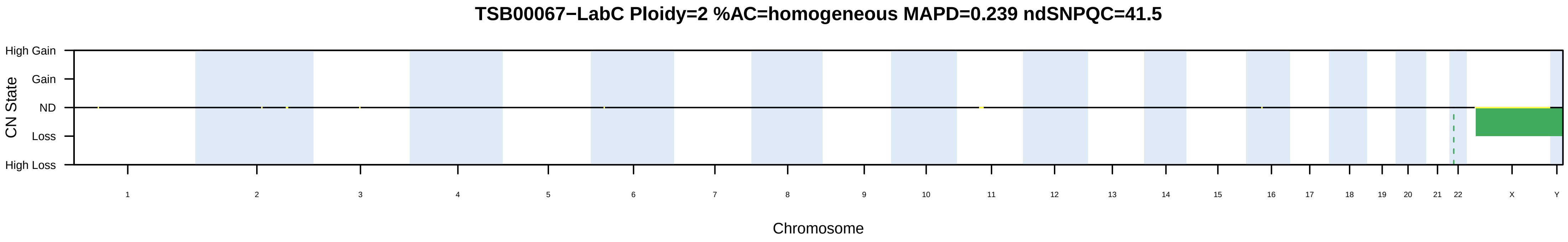
TSB00067−LabC Ploidy=2 %AC=homogeneous MAPD=0.239 ndSNPQC=41.5
CN State
High Gain
Gain
ND
Loss
High Loss
1 2 3 4 5 6 7 8 9 10 11 12 13 14 15 16 17 18 19 20 21 22 X Y
Chromosome

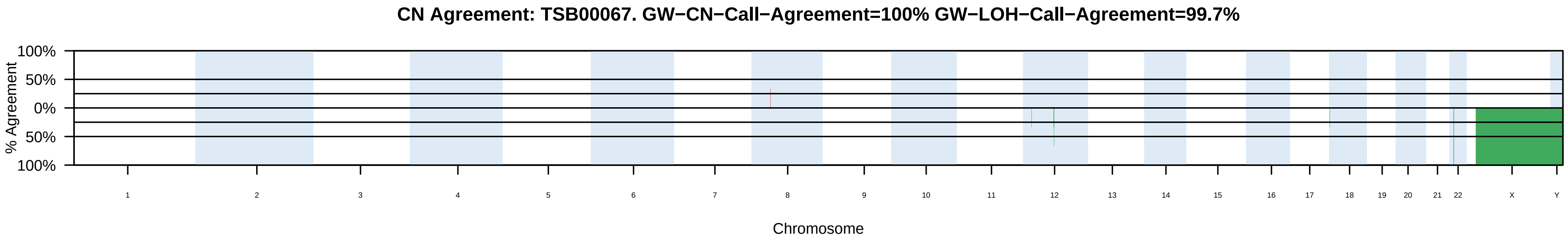
CN Agreement: TSB00067. GW−CN−Call−Agreement=100% GW−LOH−Call−Agreement=99.7%
% Agreement
100%
50%
0%
50%
100%
1 2 3 4 5 6 7 8 9 10 11 12 13 14 15 16 17 18 19 20 21 22 X Y
Chromosome

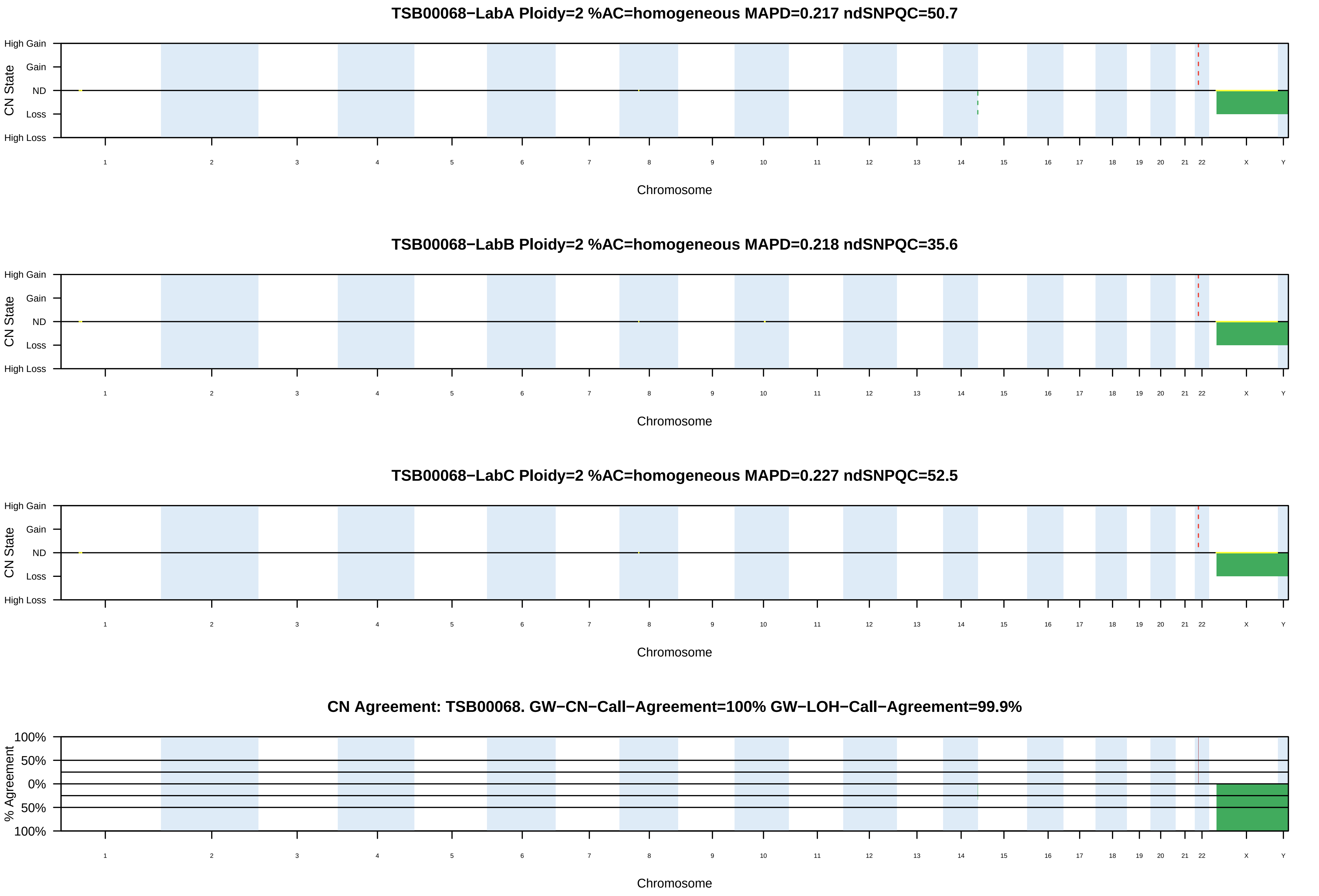
TSB00068−LabA Ploidy=2 %AC=homogeneous MAPD=0.217 ndSNPQC=50.7
TSB00068−LabB Ploidy=2 %AC=homogeneous MAPD=0.218 ndSNPQC=35.6
TSB00068−LabC Ploidy=2 %AC=homogeneous MAPD=0.227 ndSNPQC=52.5
CN Agreement: TSB00068. GW−CN−Call−Agreement=100% GW−LOH−Call−Agreement=99.9%
CN State
High Gain
Gain
ND
Loss
High Loss
% Agreement
100%
50%
0%
50%
100%
1 2 3 4 5 6 7 8 9 10 11 12 13 14 15 16 17 18 19 20 21 22 X Y
Chromosome

TSB00069−LabA Ploidy=2 %AC=homogeneous MAPD=0.258 ndSNPQC=44.4

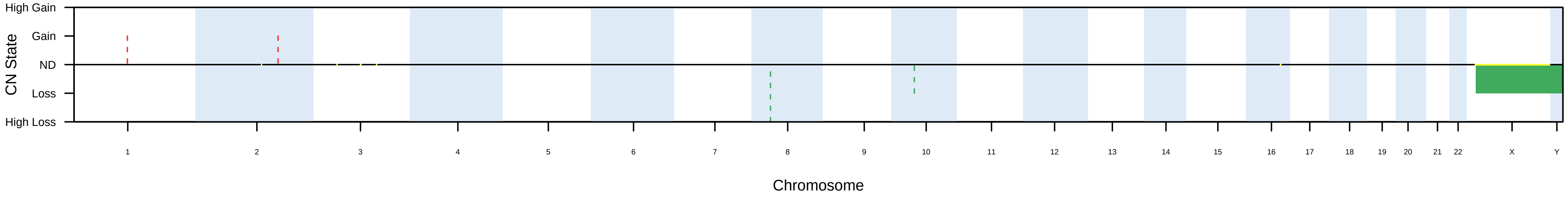

TSB00069−LabB Ploidy=2 %AC=homogeneous MAPD=0.277 ndSNPQC=26.6

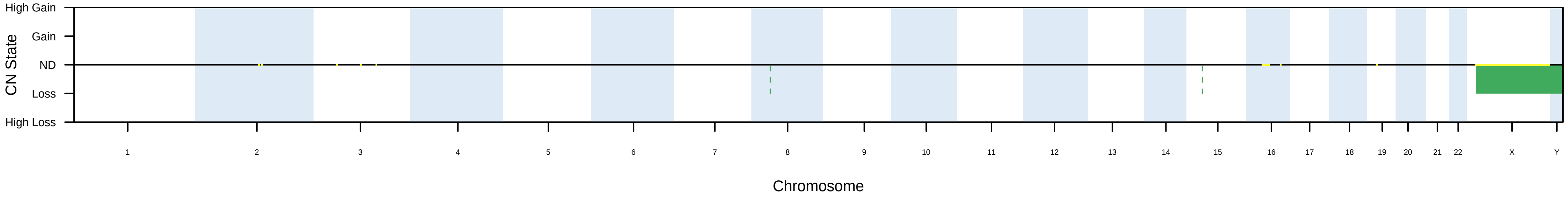

TSB00069−LabC Ploidy=2 %AC=homogeneous MAPD=0.273 ndSNPQC=32.6

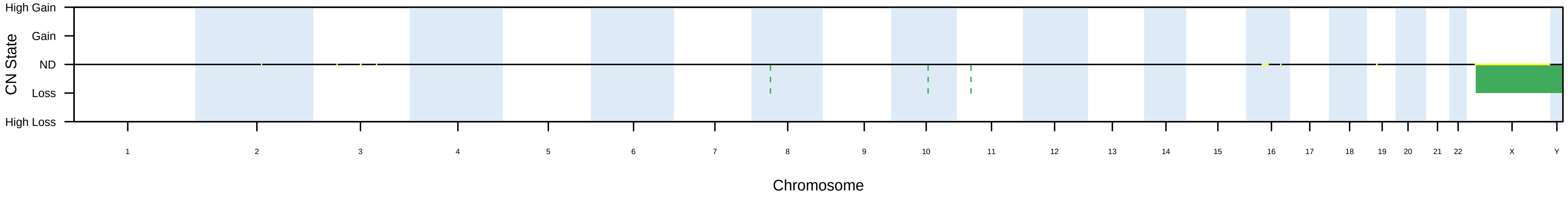

CN Agreement: TSB00069. GW−CN−Call−Agreement=99.9% GW−LOH−Call−Agreement=99.2%

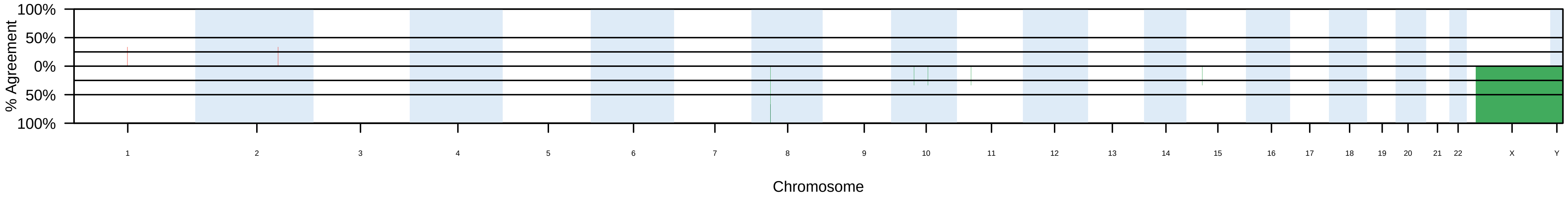

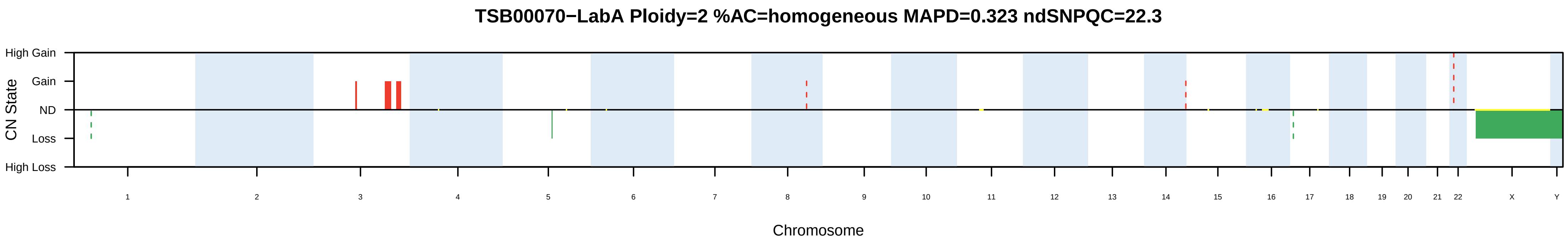
TSB00070−LabA Ploidy=2 %AC=homogeneous MAPD=0.323 ndSNPQC=22.3
High Gain
Gain
ND
Loss
High Loss
CN State
1 2 3 4 5 6 7 8 9 10 11 12 13 14 15 16 17 18 19 20 21 22 X Y
Chromosome

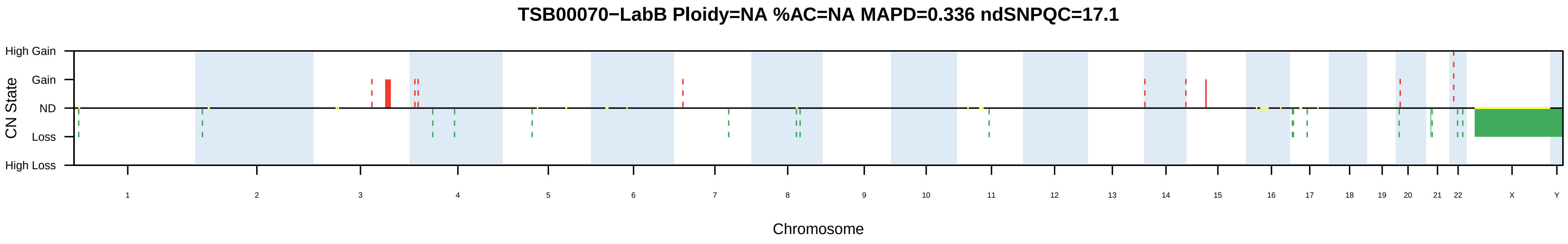
TSB00070−LabB Ploidy=NA %AC=NA MAPD=0.336 ndSNPQC=17.1
High Gain
Gain
ND
Loss
High Loss
CN State
1 2 3 4 5 6 7 8 9 10 11 12 13 14 15 16 17 18 19 20 21 22 X Y
Chromosome

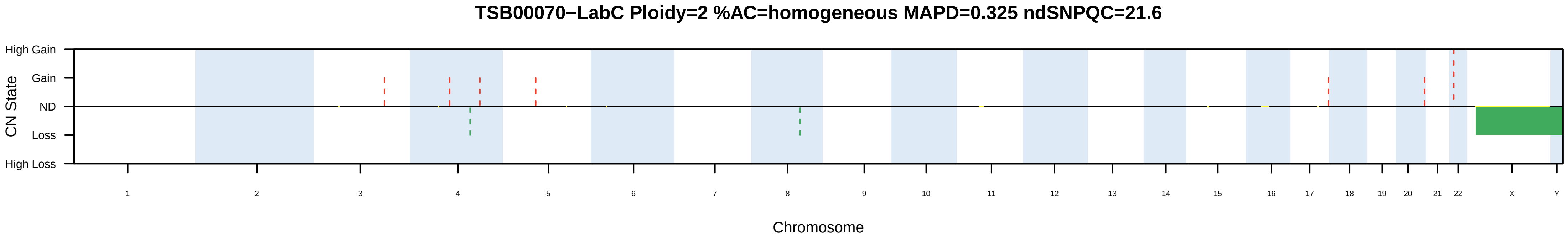
TSB00070−LabC Ploidy=2 %AC=homogeneous MAPD=0.325 ndSNPQC=21.6
High Gain
Gain
ND
Loss
High Loss
CN State
1 2 3 4 5 6 7 8 9 10 11 12 13 14 15 16 17 18 19 20 21 22 X Y
Chromosome

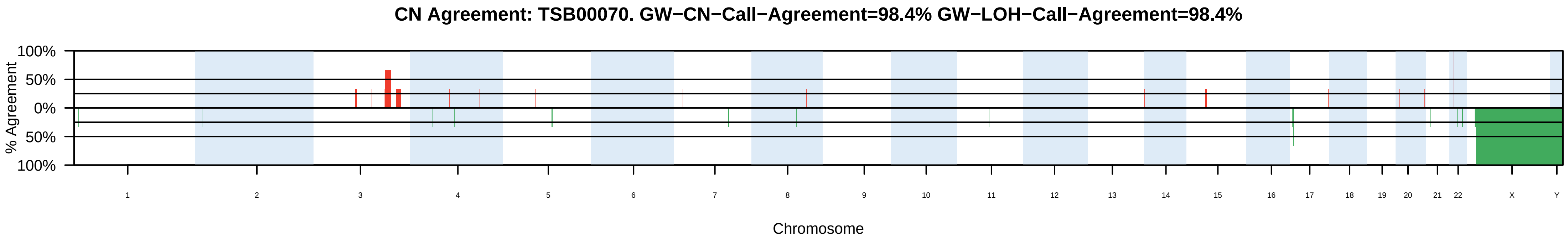
CN Agreement: TSB00070. GW−CN−Call−Agreement=98.4% GW−LOH−Call−Agreement=98.4%
100%
50%
0%
50%
100%
% Agreement
1 2 3 4 5 6 7 8 9 10 11 12 13 14 15 16 17 18 19 20 21 22 X Y
Chromosome

TSB00071−LabA Ploidy=2 %AC=homogeneous MAPD=0.274 ndSNPQC=31.5

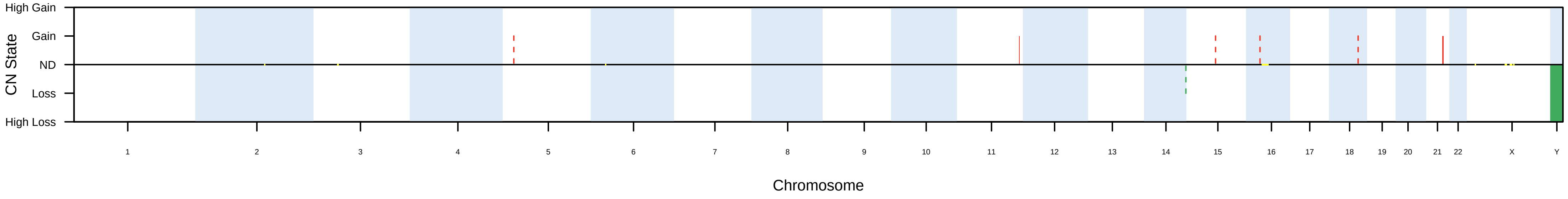

TSB00071−LabB Ploidy=2 %AC=homogeneous MAPD=0.271 ndSNPQC=25.9

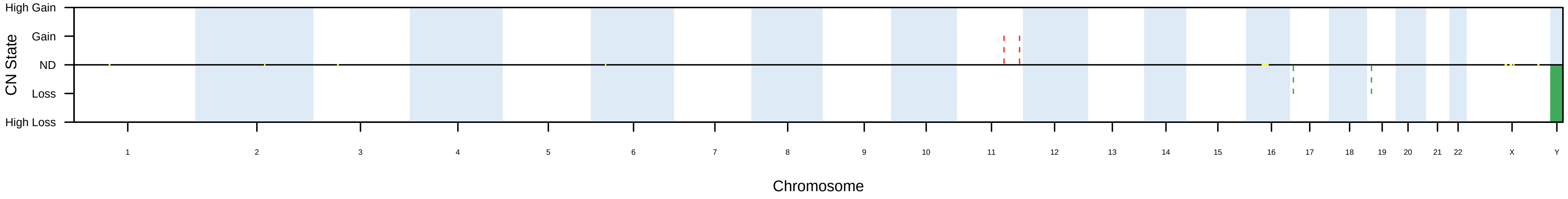

TSB00071−LabC Ploidy=2 %AC=homogeneous MAPD=0.271 ndSNPQC=29.7

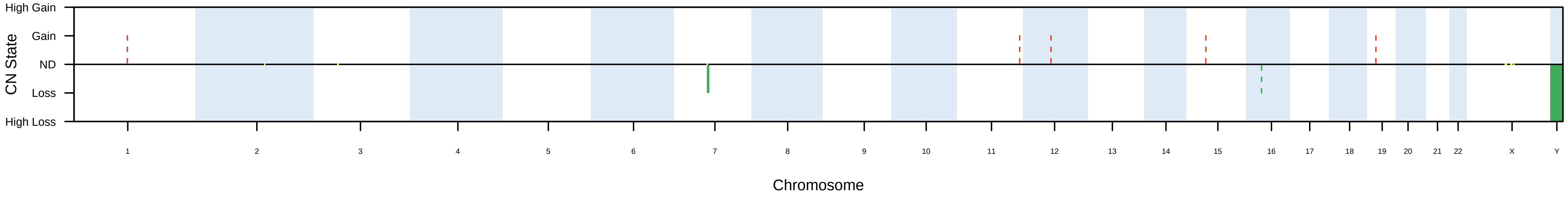

CN Agreement: TSB00071. GW−CN−Call−Agreement=99.5% GW−LOH−Call−Agreement=98.9%

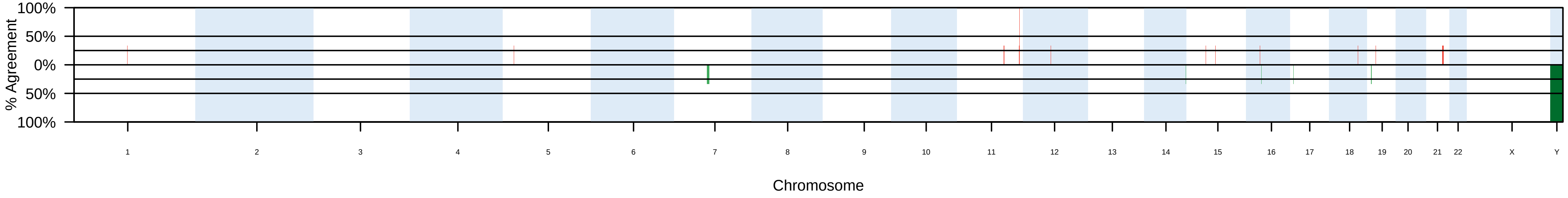

TSB00072−LabA Ploidy=2 %AC=homogeneous MAPD=0.245 ndSNPQC=39.6

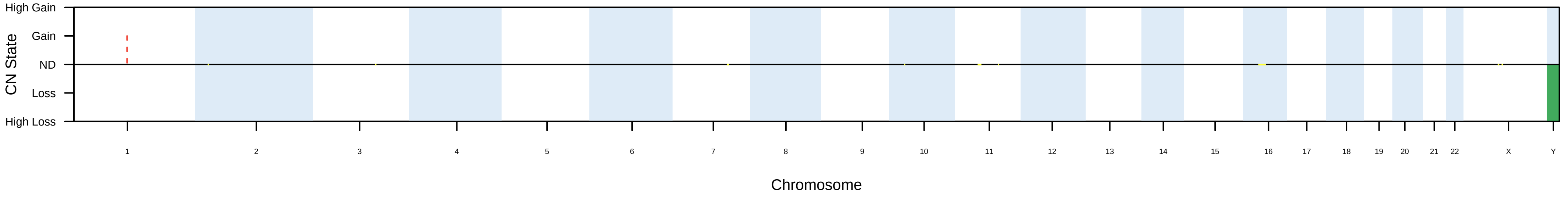

TSB00072−LabB Ploidy=2 %AC=homogeneous MAPD=0.234 ndSNPQC=36.9

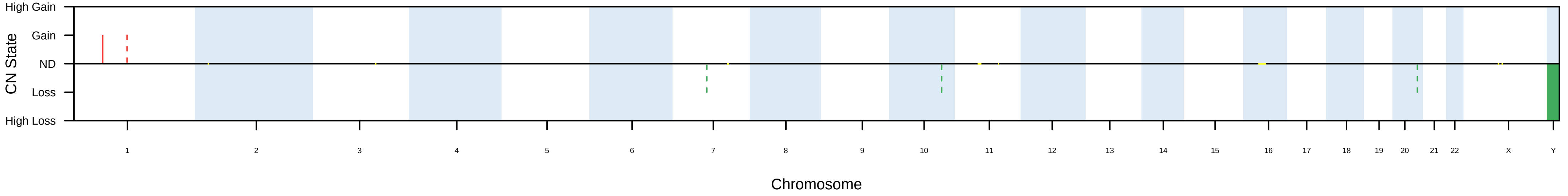

TSB00072−LabC Ploidy=2 %AC=homogeneous MAPD=0.239 ndSNPQC=47.7

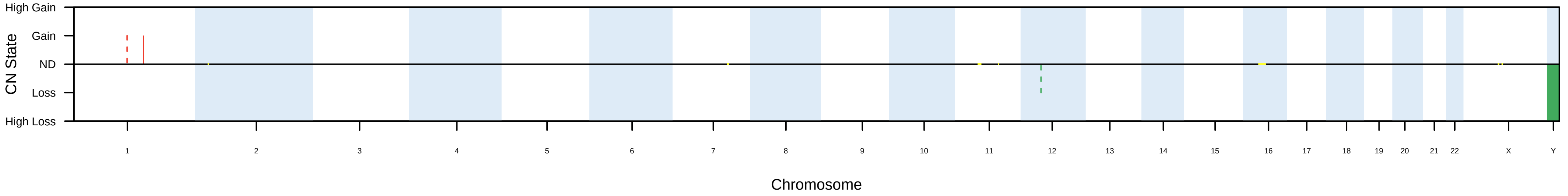

CN Agreement: TSB00072. GW−CN−Call−Agreement=99.8% GW−LOH−Call−Agreement=99.8%

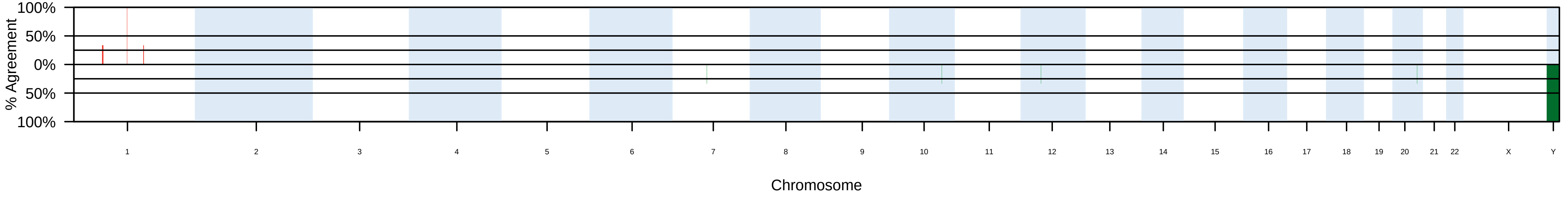

TSB00073−LabA Ploidy=2 %AC=homogeneous MAPD=0.262 ndSNPQC=32.3

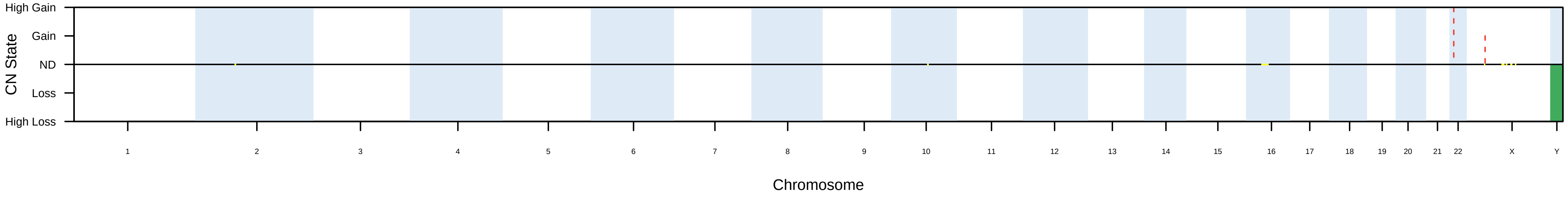

TSB00073−LabB Ploidy=2 %AC=homogeneous MAPD=0.296 ndSNPQC=24.7

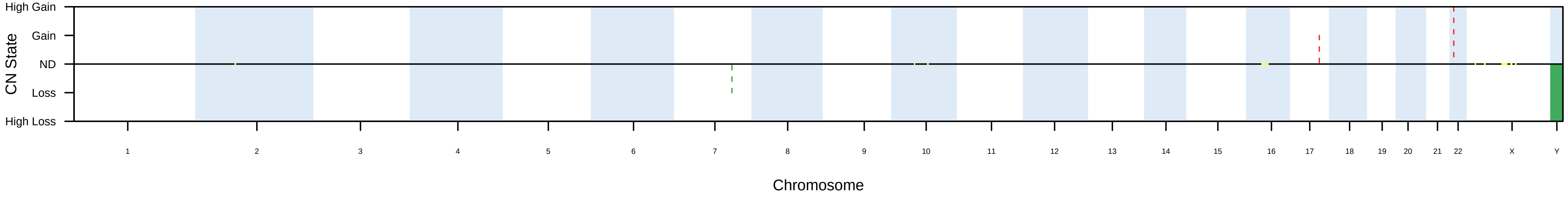

TSB00073−LabC Ploidy=2 %AC=homogeneous MAPD=0.273 ndSNPQC=33

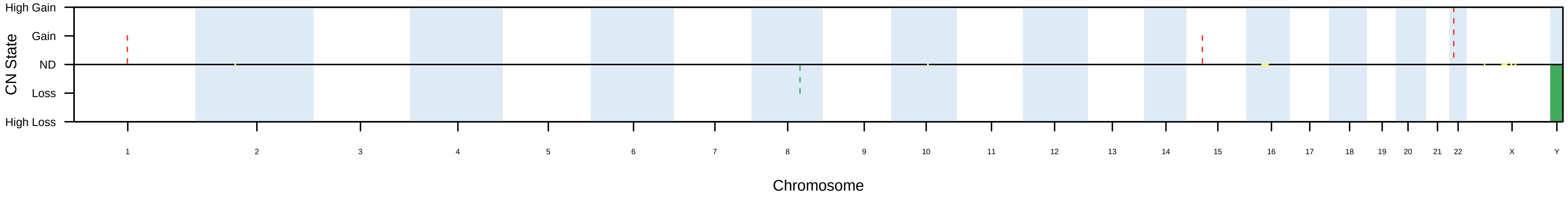

CN Agreement: TSB00073. GW−CN−Call−Agreement=99.9% GW−LOH−Call−Agreement=99.7%

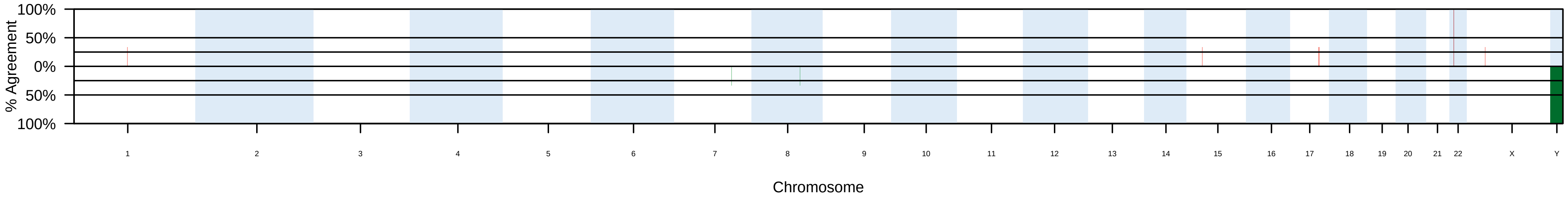

TSB00074−LabA Ploidy=2 %AC=homogeneous MAPD=0.226 ndSNPQC=48.4

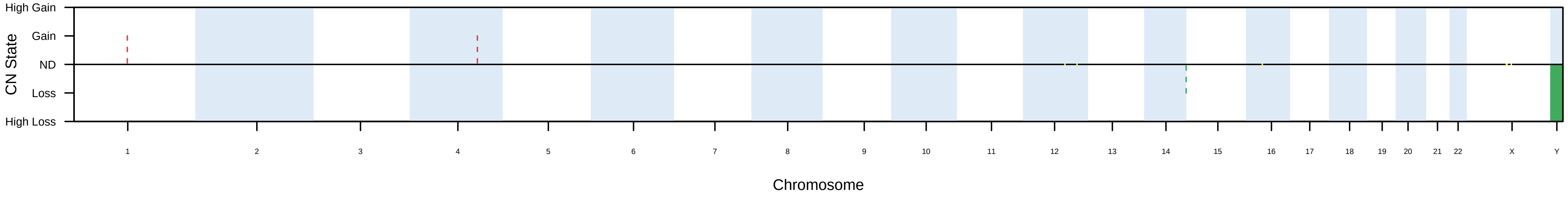

TSB00074−LabB Ploidy=2 %AC=homogeneous MAPD=0.223 ndSNPQC=39.2

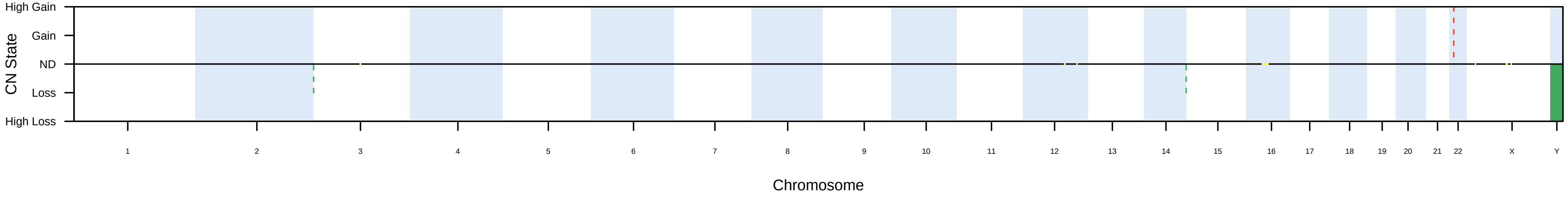

TSB00074−LabC Ploidy=2 %AC=homogeneous MAPD=0.225 ndSNPQC=51.1

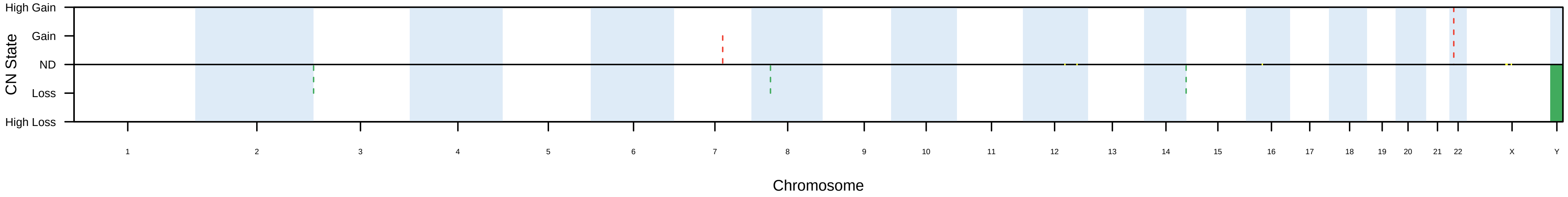

CN Agreement: TSB00074. GW−CN−Call−Agreement=100% GW−LOH−Call−Agreement=99.4%

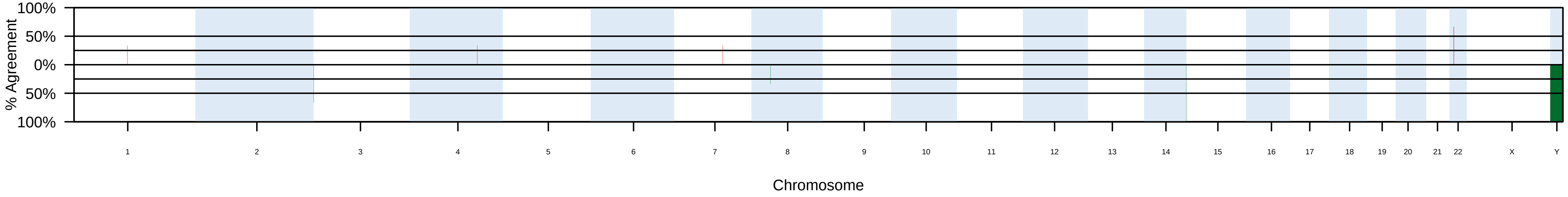

TSB00076−LabA Ploidy=2 %AC=homogeneous MAPD=0.279 ndSNPQC=27.3

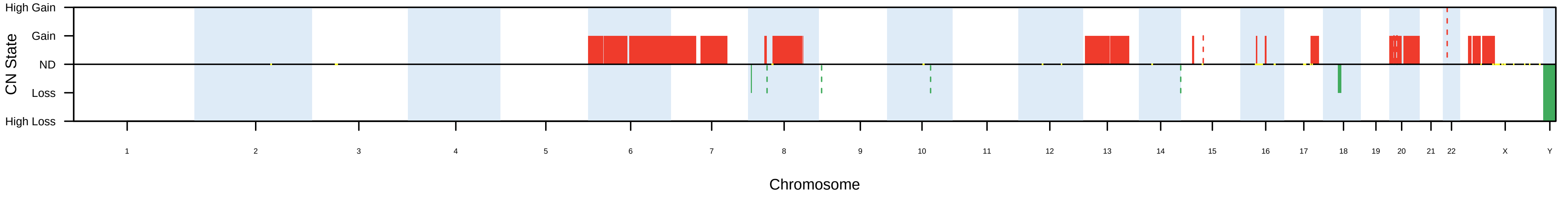

TSB00076−LabB Ploidy=2 %AC=homogeneous MAPD=0.305 ndSNPQC=25.8

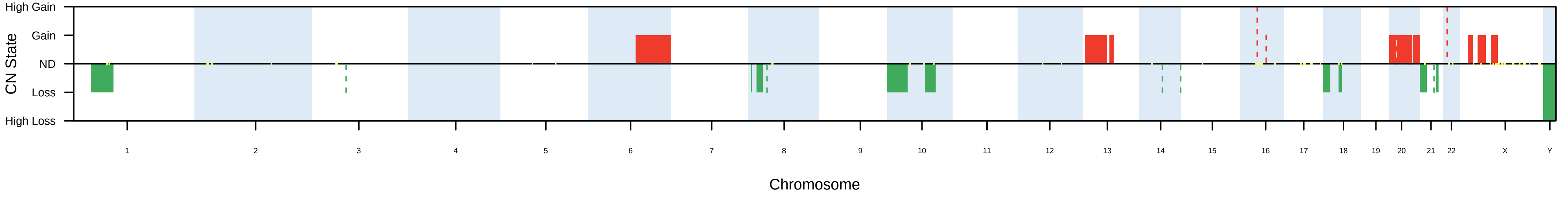

TSB00076−LabC Ploidy=2 %AC=homogeneous MAPD=0.285 ndSNPQC=23

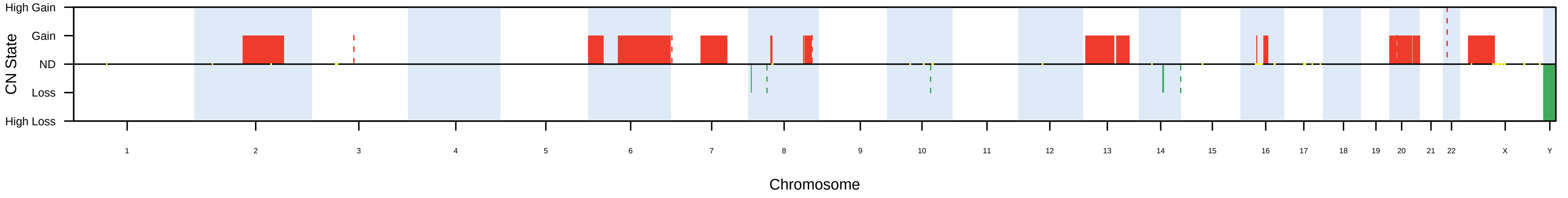

CN Agreement: TSB00076. GW−CN−Call−Agreement=76.9% GW−LOH−Call−Agreement=97%

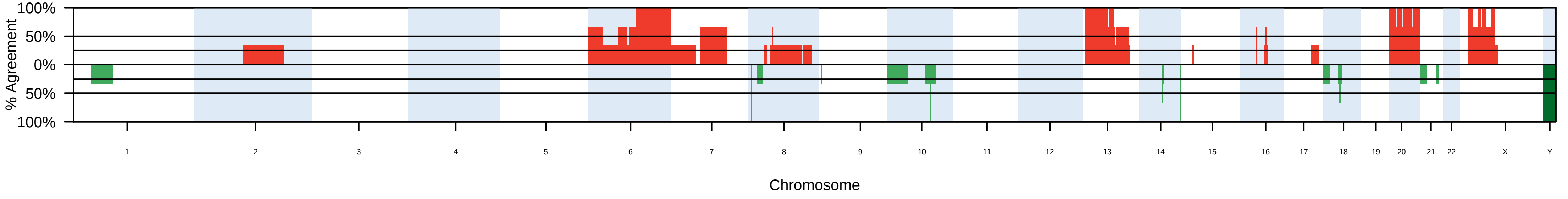

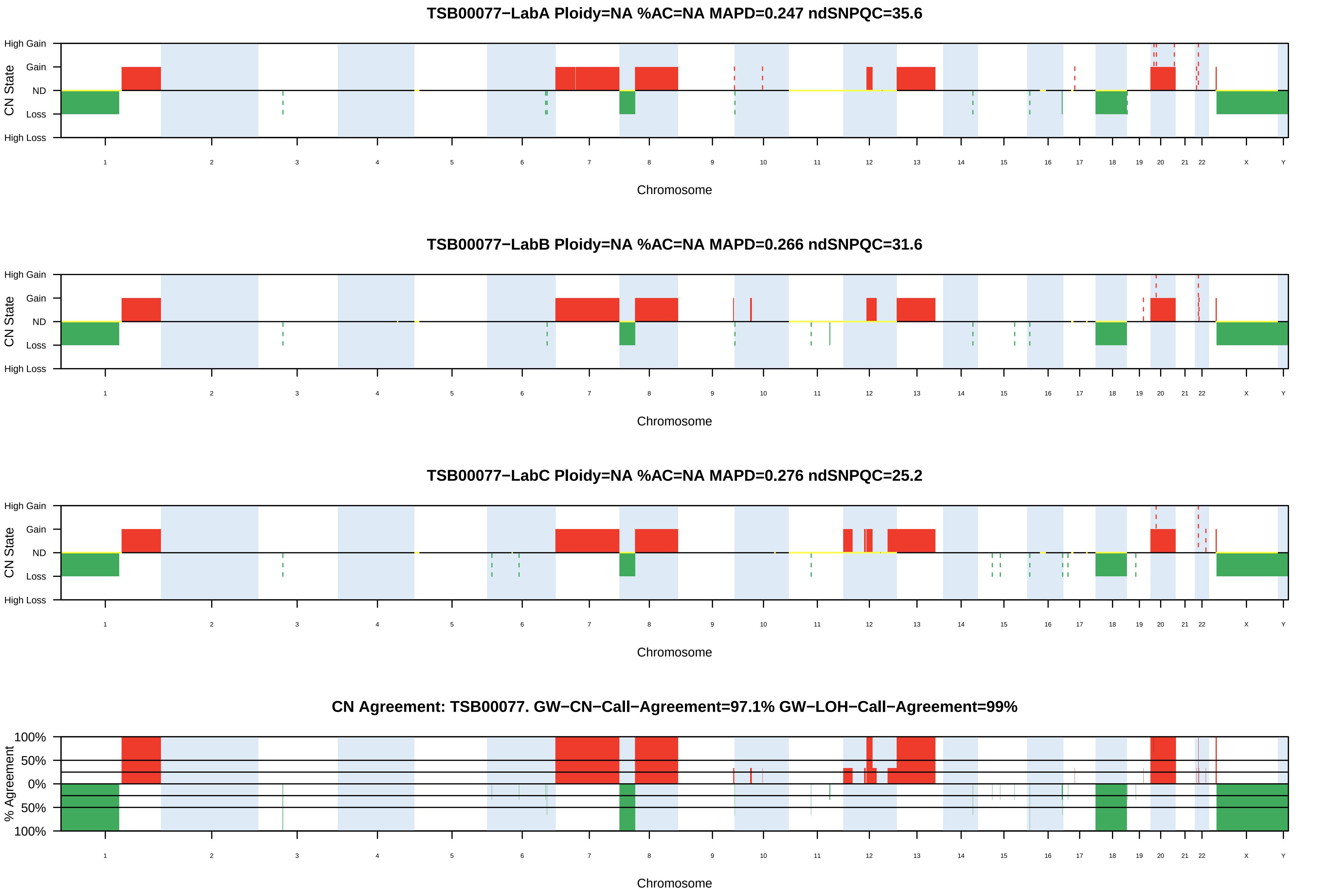
TSB00077−LabA Ploidy=NA %AC=NA MAPD=0.247 ndSNPQC=35.6
High Gain
Gain
ND
Loss
High Loss
CN State
1 2 3 4 5 6 7 8 9 10 11 12 13 14 15 16 17 18 19 20 21 22 X Y
Chromosome
TSB00077−LabB Ploidy=NA %AC=NA MAPD=0.266 ndSNPQC=31.6
High Gain
Gain
ND
Loss
High Loss
CN State
1 2 3 4 5 6 7 8 9 10 11 12 13 14 15 16 17 18 19 20 21 22 X Y
Chromosome
TSB00077−LabC Ploidy=NA %AC=NA MAPD=0.276 ndSNPQC=25.2
High Gain
Gain
ND
Loss
High Loss
CN State
1 2 3 4 5 6 7 8 9 10 11 12 13 14 15 16 17 18 19 20 21 22 X Y
Chromosome
CN Agreement: TSB00077. GW−CN−Call−Agreement=97.1% GW−LOH−Call−Agreement=99%
100%
50%
0%
50%
100%
% Agreement
1 2 3 4 5 6 7 8 9 10 11 12 13 14 15 16 17 18 19 20 21 22 X Y
Chromosome

TSB00078−LabA Ploidy=NA %AC=NA MAPD=0.332 ndSNPQC=23.9

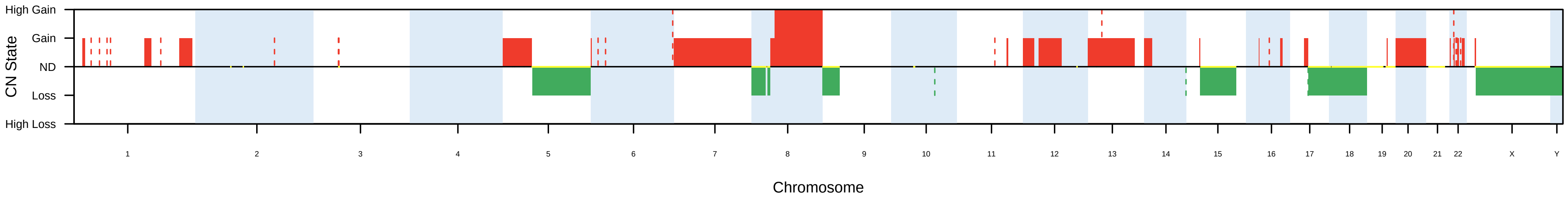

TSB00078−LabB Ploidy=2 %AC=45 MAPD=0.274 ndSNPQC=34.4

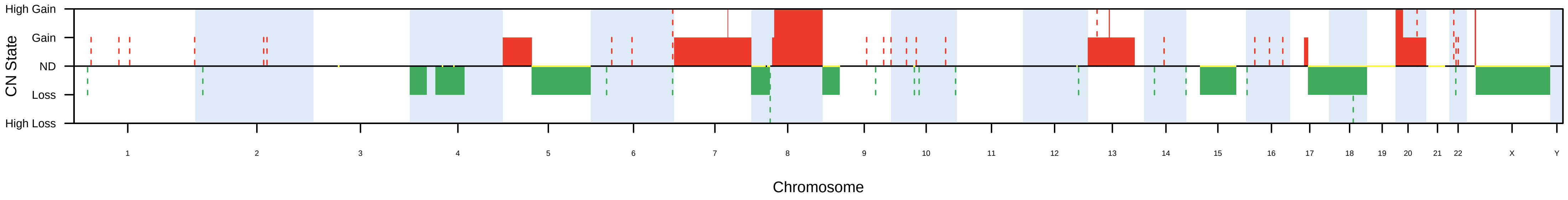

TSB00078−LabC Ploidy=2 %AC=45 MAPD=0.288 ndSNPQC=26.3

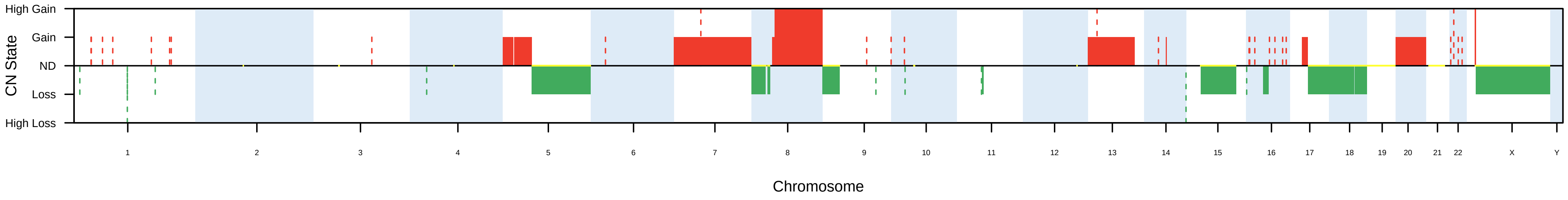

CN Agreement: TSB00078. GW−CN−Call−Agreement=88.1% GW−LOH−Call−Agreement=99.3%

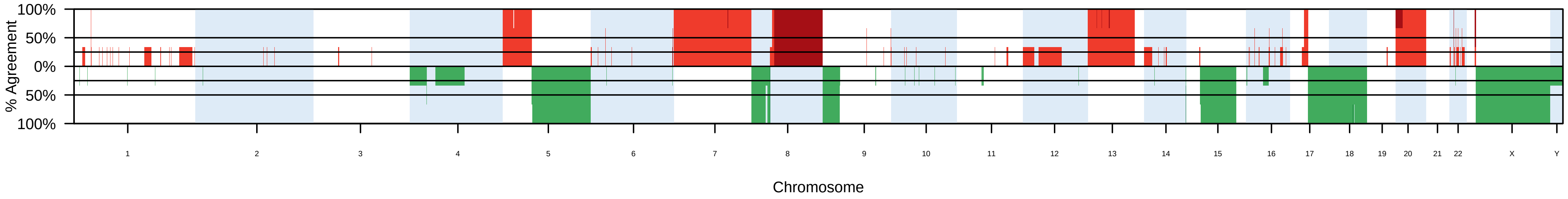

TSB00079−LabA Ploidy=NA %AC=NA MAPD=0.226 ndSNPQC=39

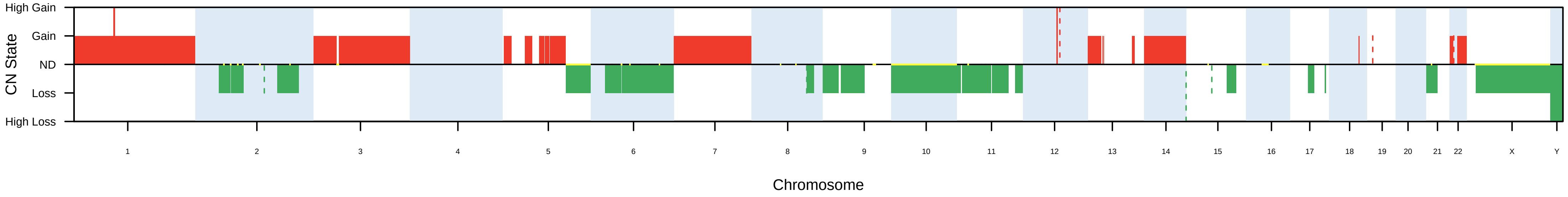

TSB00079−LabB Ploidy=NA %AC=NA MAPD=0.229 ndSNPQC=37

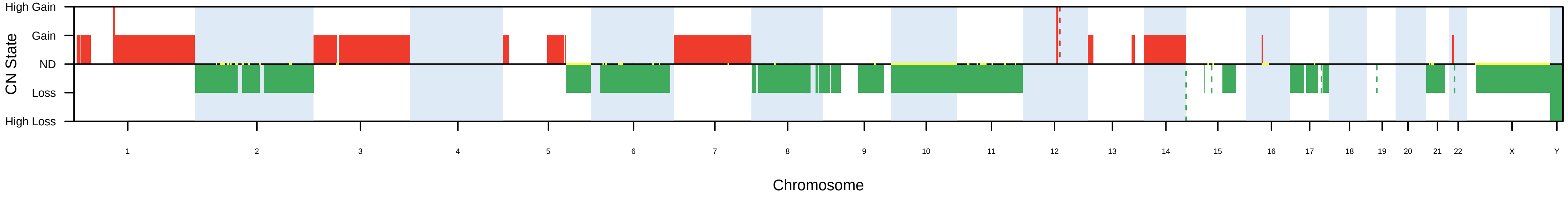

TSB00079−LabC Ploidy=NA %AC=NA MAPD=0.234 ndSNPQC=33.6

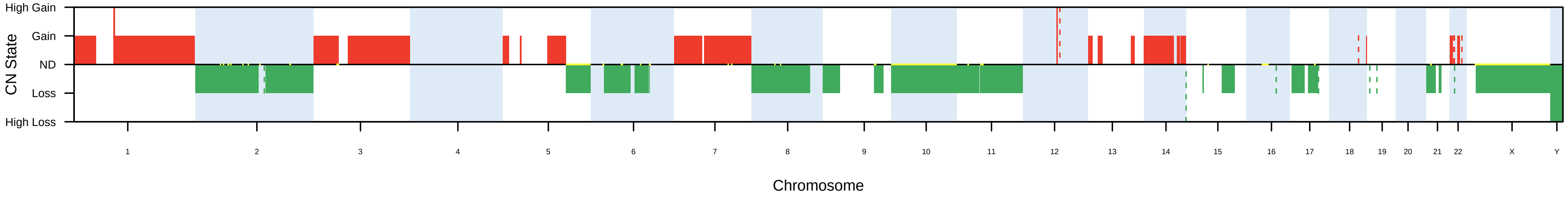

CN Agreement: TSB00079. GW−CN−Call−Agreement=74.3% GW−LOH−Call−Agreement=95.9%

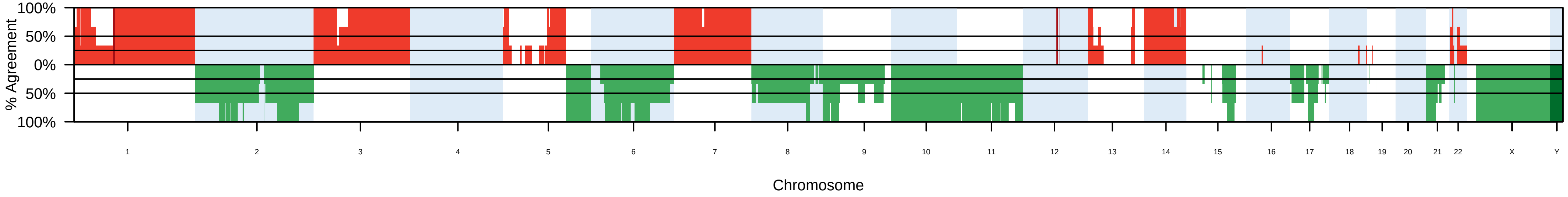

TSB00080−LabA Ploidy=NA %AC=NA MAPD=0.234 ndSNPQC=41.7

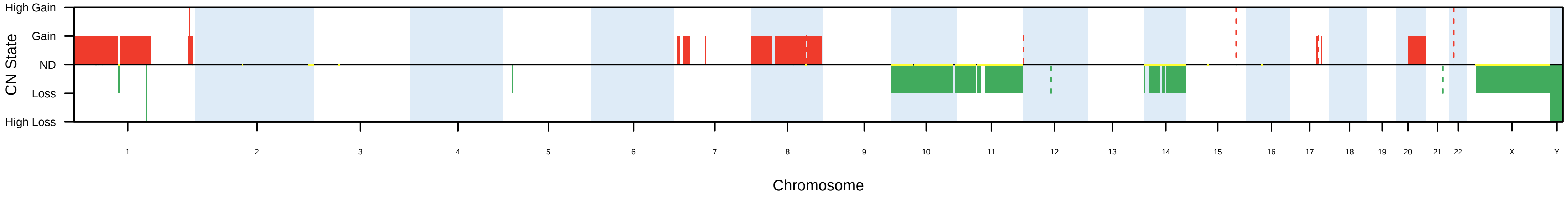

TSB00080−LabB Ploidy=2 %AC=30 MAPD=0.221 ndSNPQC=38.2

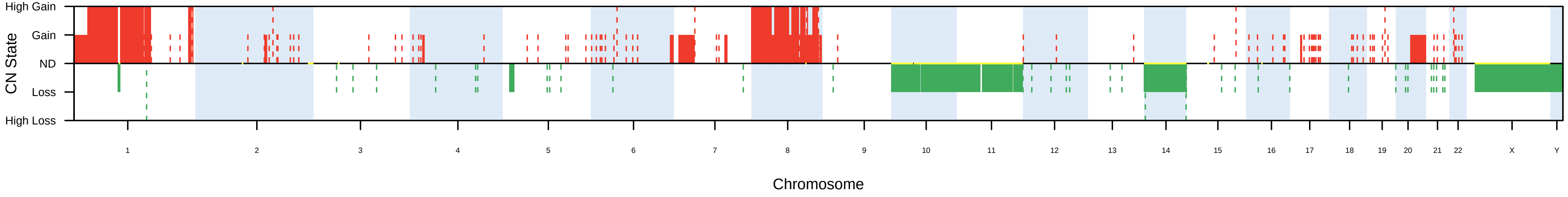

TSB00080−LabC Ploidy=NA %AC=NA MAPD=0.279 ndSNPQC=40.4

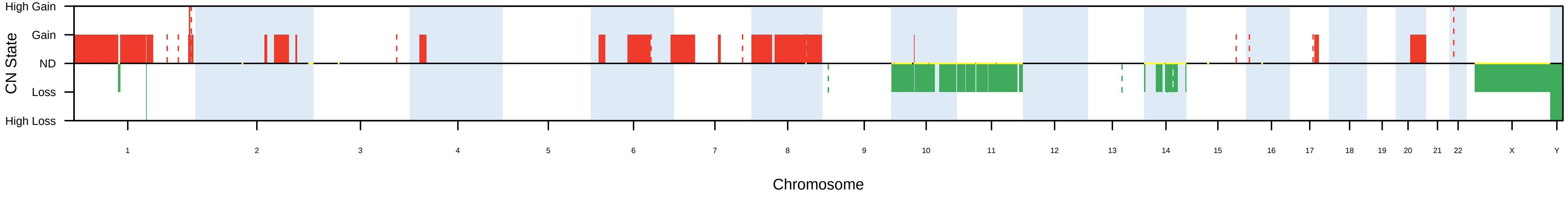

CN Agreement: TSB00080. GW−CN−Call−Agreement=80.6% GW−LOH−Call−Agreement=99.2%

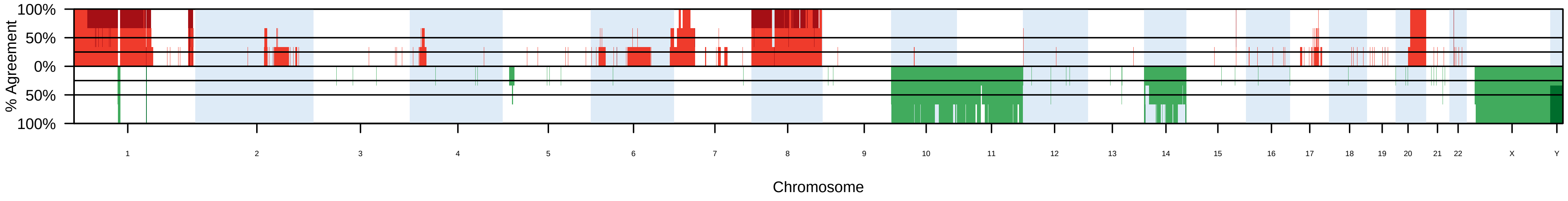

TSB00081−LabA Ploidy=NA %AC=NA MAPD=0.259 ndSNPQC=38.3

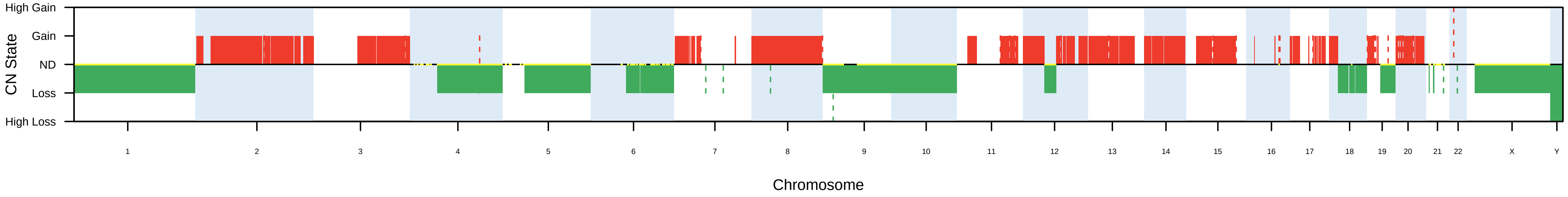

TSB00081−LabB Ploidy=NA %AC=NA MAPD=0.255 ndSNPQC=33.5

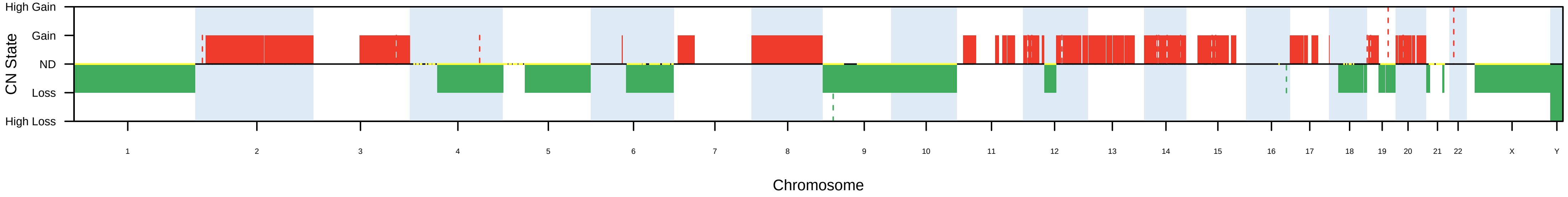

TSB00081−LabC Ploidy=NA %AC=NA MAPD=0.286 ndSNPQC=33.8

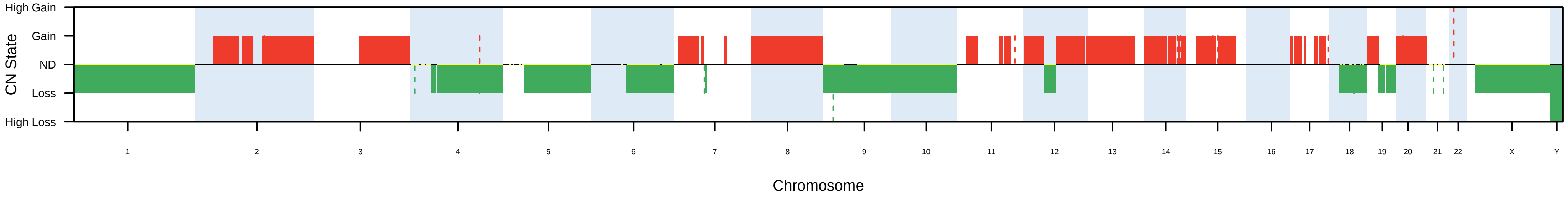

CN Agreement: TSB00081. GW−CN−Call−Agreement=87.5% GW−LOH−Call−Agreement=95.5%

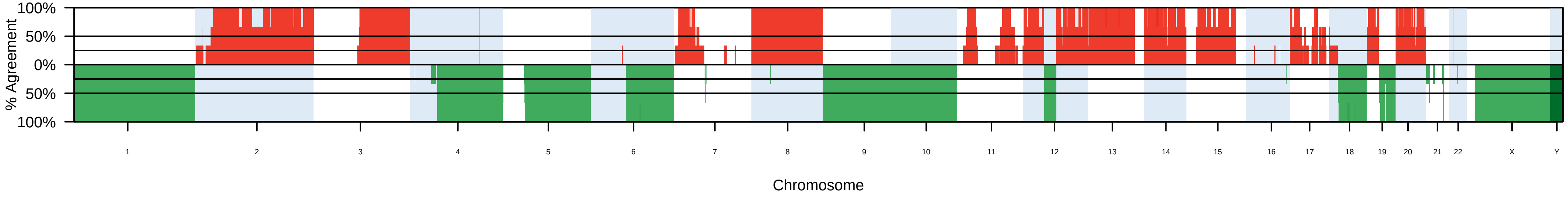

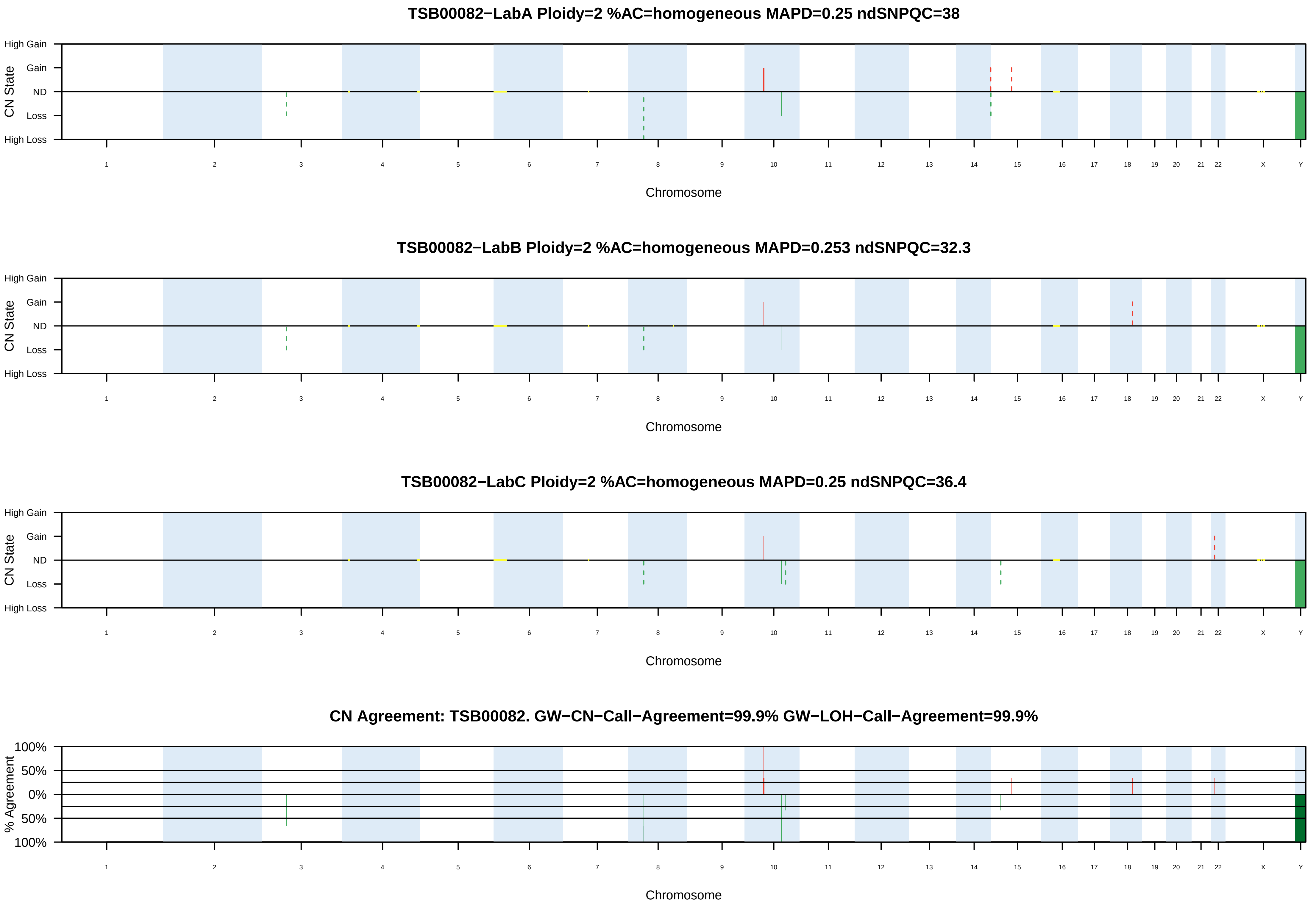

TSB00082−LabA Ploidy=2 %AC=homogeneous MAPD=0.25 ndSNPQC=38
TSB00082−LabB Ploidy=2 %AC=homogeneous MAPD=0.253 ndSNPQC=32.3
TSB00082−LabC Ploidy=2 %AC=homogeneous MAPD=0.25 ndSNPQC=36.4
CN State
High Gain
Gain
ND
Loss
High Loss
Chromosome
1 2 3 4 5 6 7 8 9 10 11 12 13 14 15 16 17 18 19 20 21 22 X Y
CN Agreement: TSB00082. GW−CN−Call−Agreement=99.9% GW−LOH−Call−Agreement=99.9%
% Agreement
100%
50%
0%
50%
100%

TSB00083−LabA Ploidy=NA %AC=NA MAPD=0.239 ndSNPQC=36.6

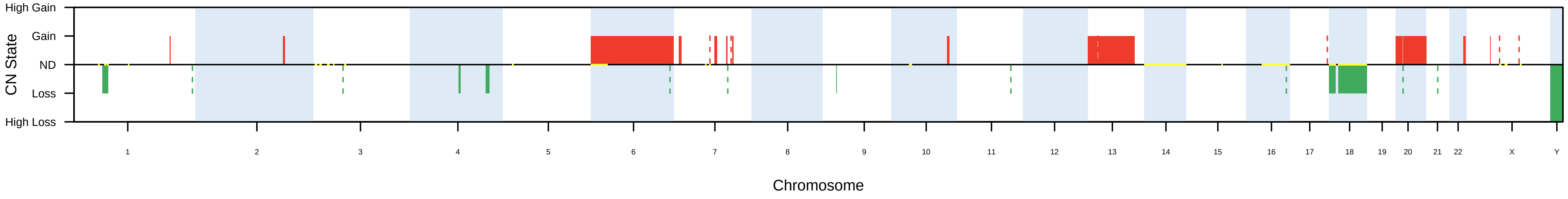

TSB00083−LabB Ploidy=NA %AC=NA MAPD=0.243 ndSNPQC=34.3

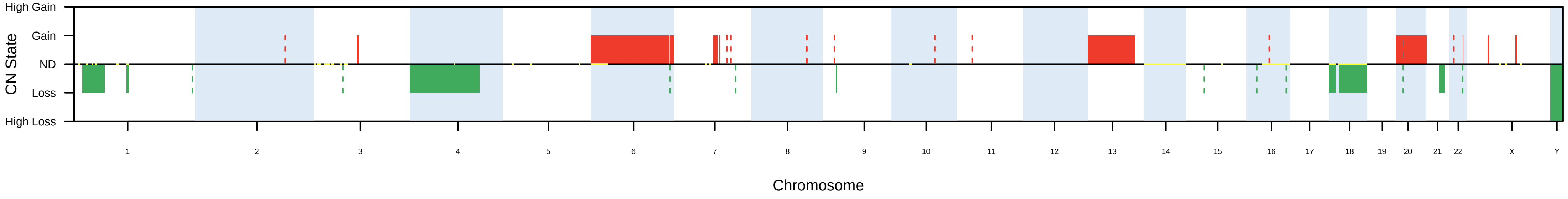

TSB00083−LabC Ploidy=NA %AC=NA MAPD=0.251 ndSNPQC=33.5

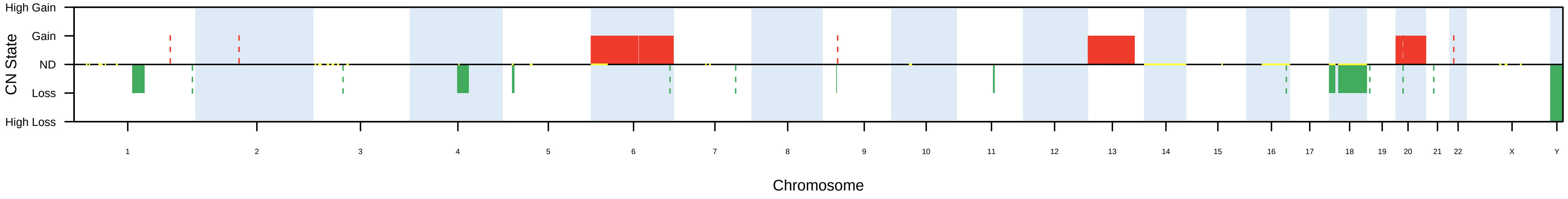

CN Agreement: TSB00083. GW−CN−Call−Agreement=90.1% GW−LOH−Call−Agreement=96.8%

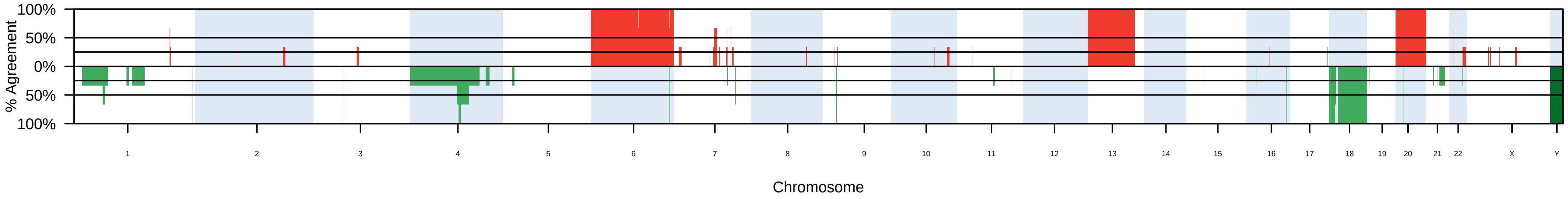

TSB00084−LabA Ploidy=2 %AC=40 MAPD=0.254 ndSNPQC=35.8

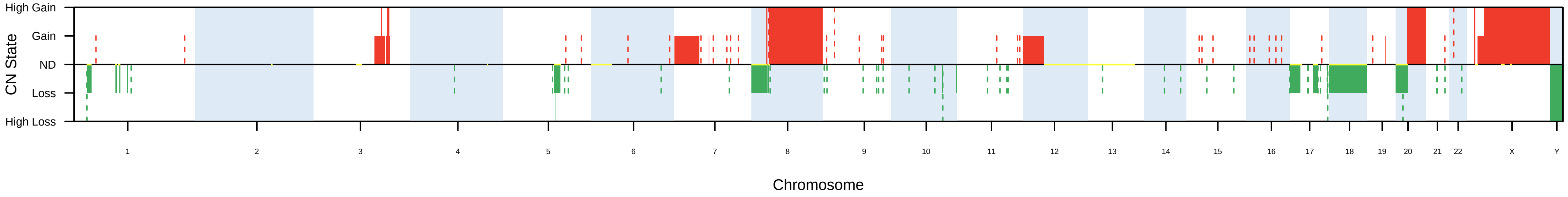

TSB00084−LabB Ploidy=2 %AC=40 MAPD=0.265 ndSNPQC=33.5

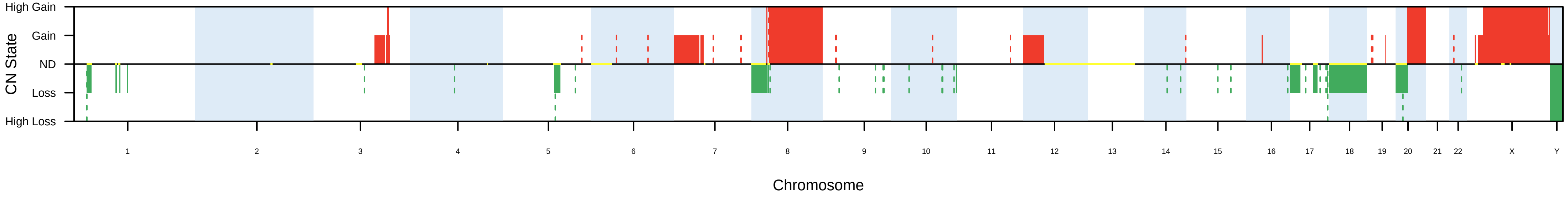

TSB00084−LabC Ploidy=2 %AC=40 MAPD=0.264 ndSNPQC=33.2

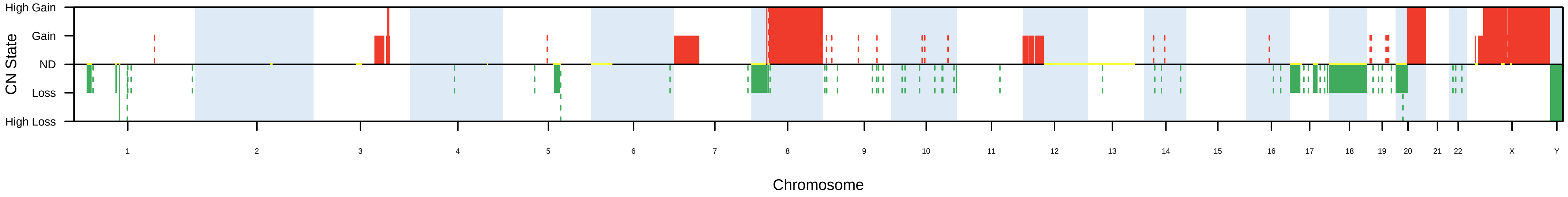

CN Agreement: TSB00084. GW−CN−Call−Agreement=97.4% GW−LOH−Call−Agreement=99.7%

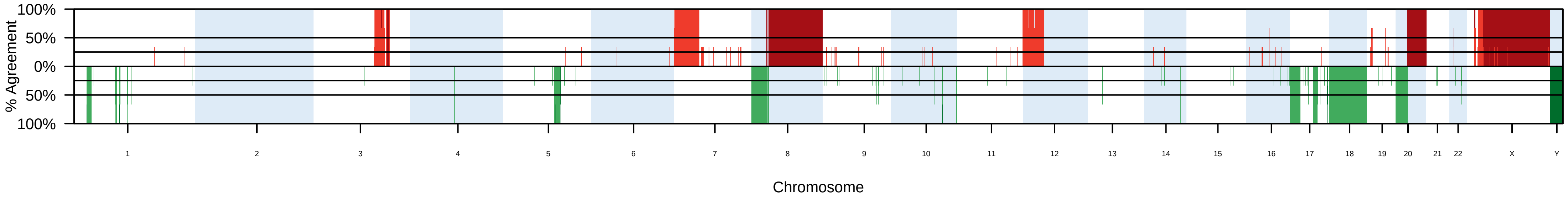

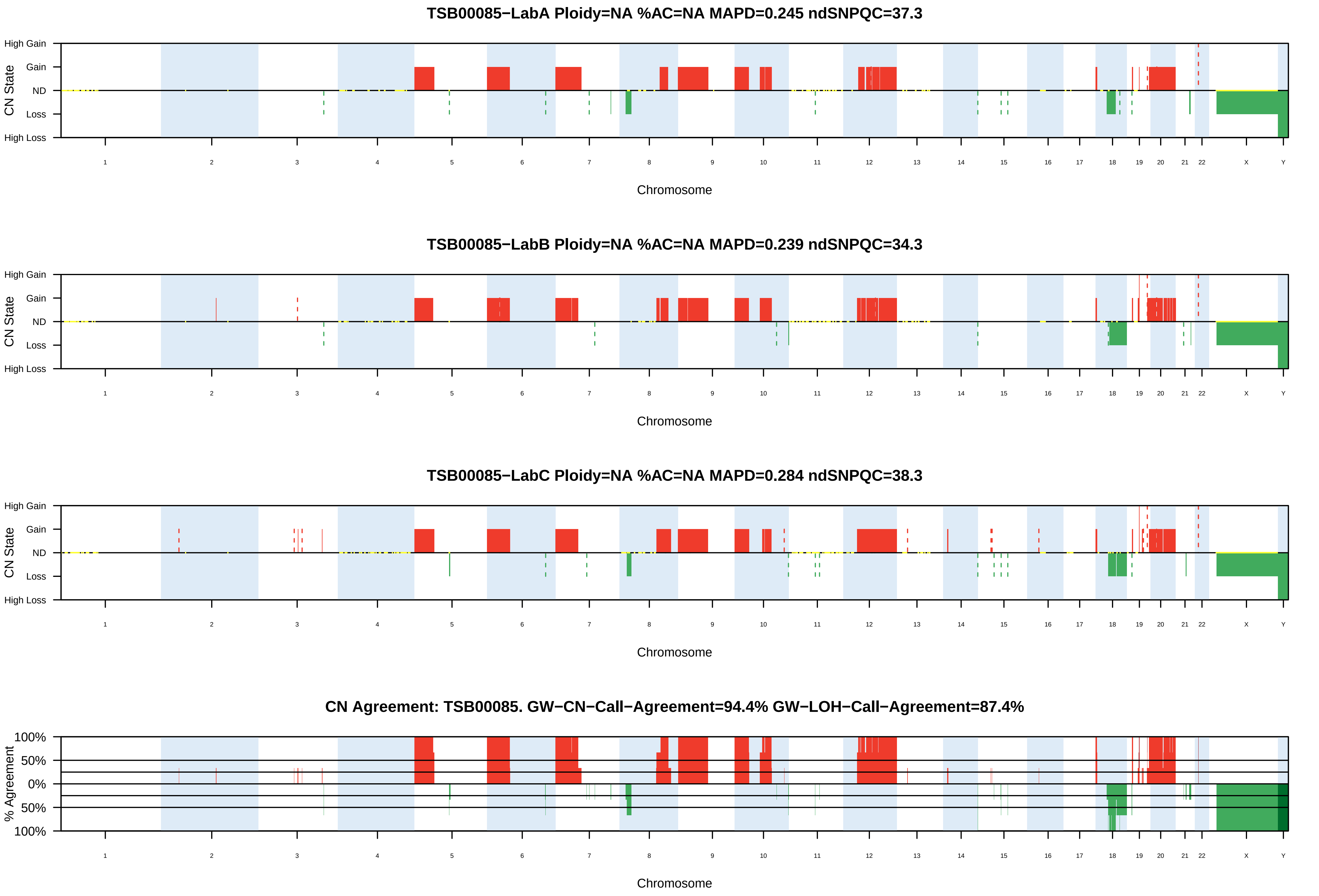

TSB00085−LabA Ploidy=NA %AC=NA MAPD=0.245 ndSNPQC=37.3
TSB00085−LabB Ploidy=NA %AC=NA MAPD=0.239 ndSNPQC=34.3
TSB00085−LabC Ploidy=NA %AC=NA MAPD=0.284 ndSNPQC=38.3
CN Agreement: TSB00085. GW−CN−Call−Agreement=94.4% GW−LOH−Call−Agreement=87.4%
CN State
High Gain
Gain
ND
Loss
High Loss
% Agreement
100%
50%
0%
Chromosome
1 2 3 4 5 6 7 8 9 10 11 12 13 14 15 16 17 18 19 20 21 22 X Y

TSB00086−LabA Ploidy=2 %AC=homogeneous MAPD=0.24 ndSNPQC=43.6

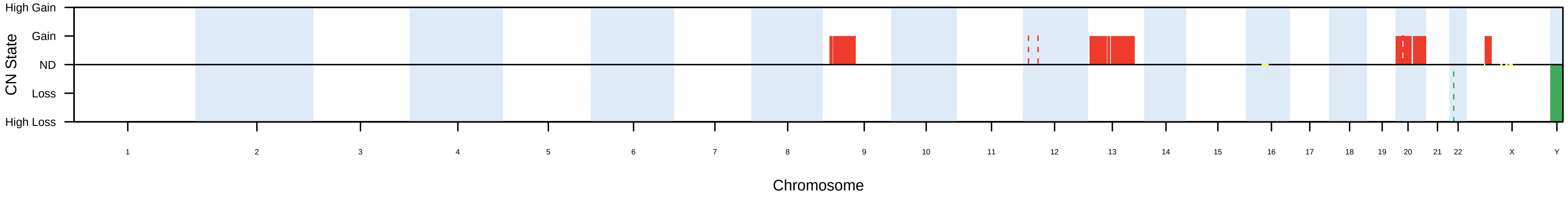

TSB00086−LabB Ploidy=2 %AC=homogeneous MAPD=0.211 ndSNPQC=46.8

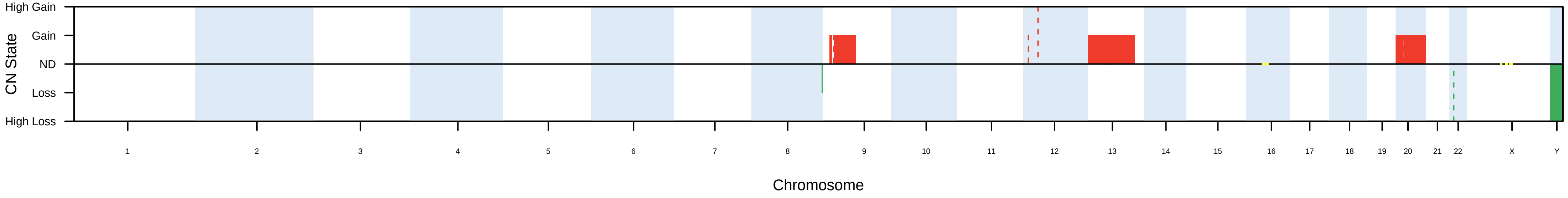

TSB00086−LabC Ploidy=2 %AC=homogeneous MAPD=0.263 ndSNPQC=51.5

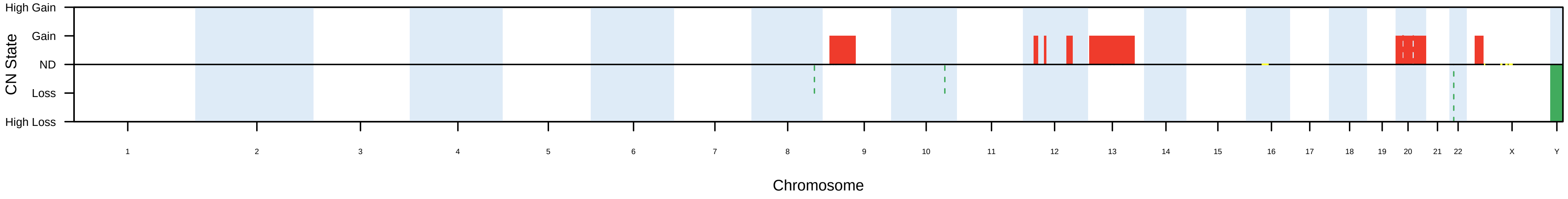

CN Agreement: TSB00086. GW−CN−Call−Agreement=97.1% GW−LOH−Call−Agreement=99.9%

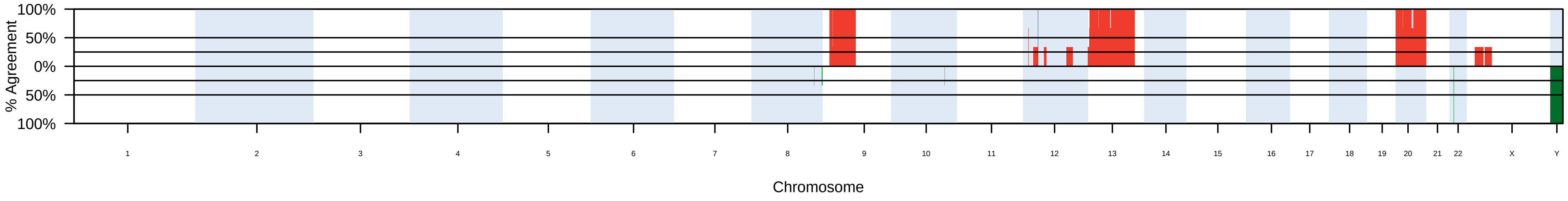

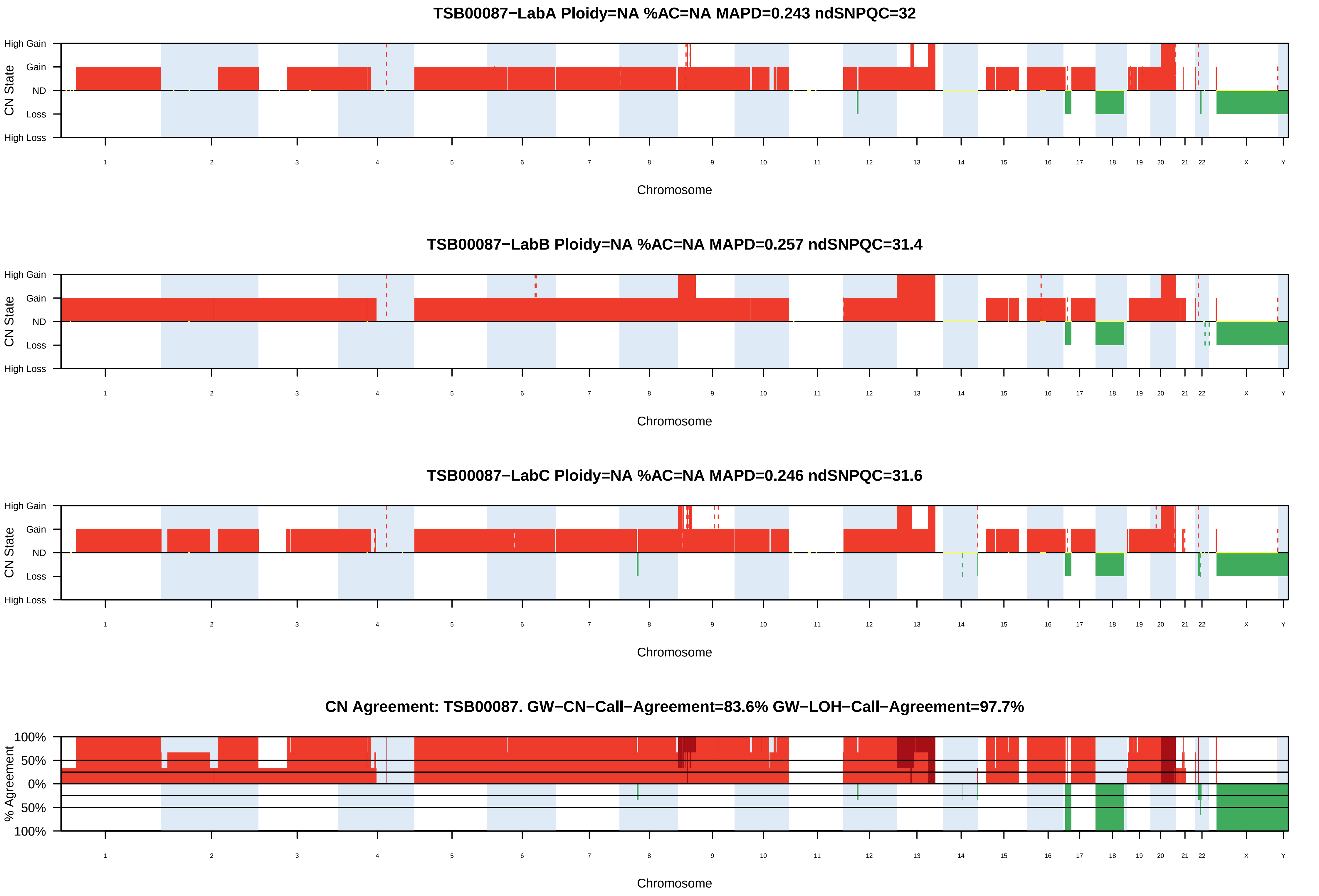
TSB00087−LabA Ploidy=NA %AC=NA MAPD=0.243 ndSNPQC=32
TSB00087−LabB Ploidy=NA %AC=NA MAPD=0.257 ndSNPQC=31.4
TSB00087−LabC Ploidy=NA %AC=NA MAPD=0.246 ndSNPQC=31.6
CN State
High Gain
Gain
ND
Loss
High Loss
Chromosome
1 2 3 4 5 6 7 8 9 10 11 12 13 14 15 16 17 18 19 20 21 22 X Y
CN Agreement: TSB00087. GW−CN−Call−Agreement=83.6% GW−LOH−Call−Agreement=97.7%
% Agreement
100%
50%
0%
50%
100%

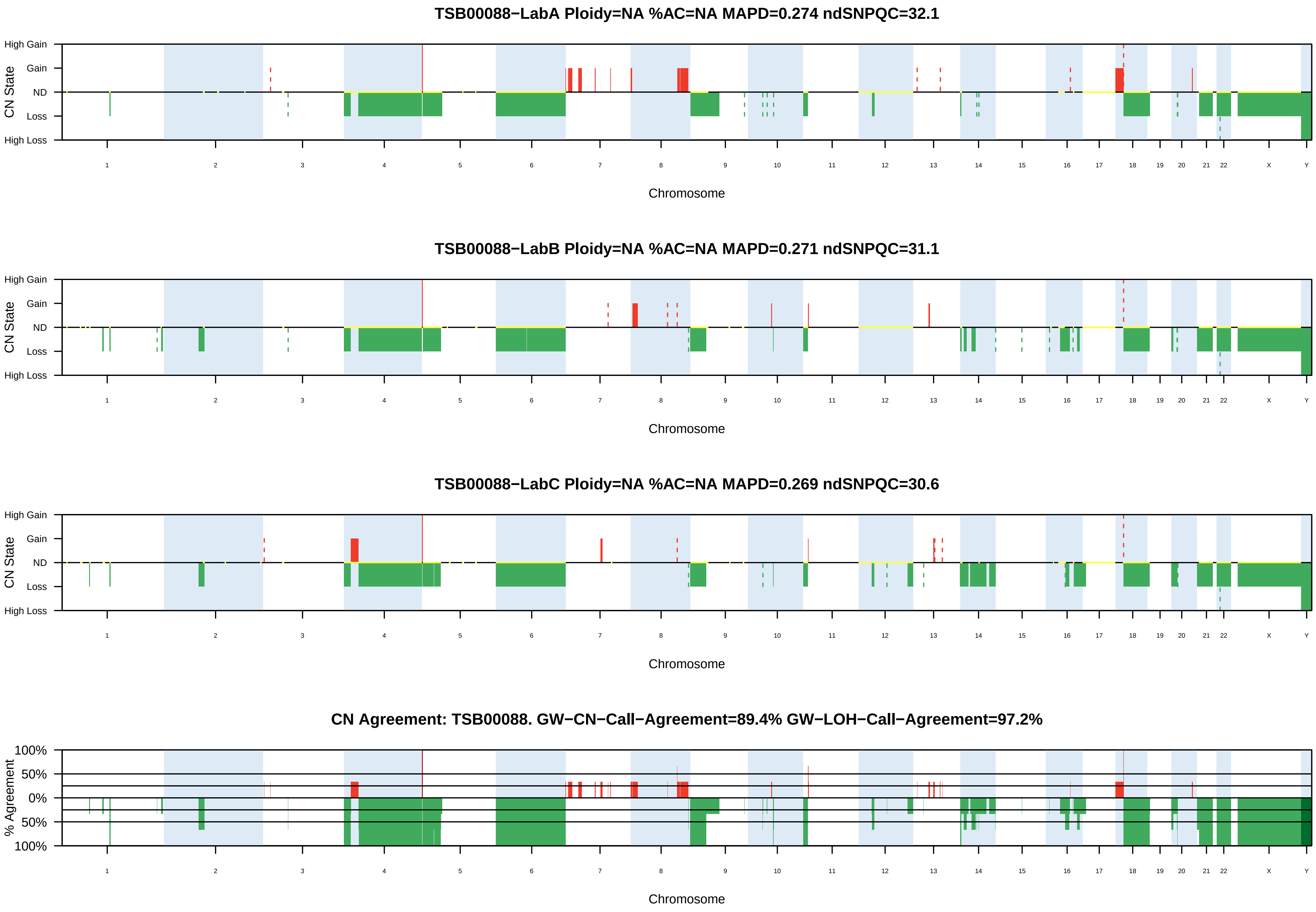
TSB00088−LabA Ploidy=NA %AC=NA MAPD=0.274 ndSNPQC=32.1
CN State
High Gain
Gain
ND
Loss
High Loss
1 2 3 4 5 6 7 8 9 10 11 12 13 14 15 16 17 18 19 20 21 22 X Y
Chromosome
TSB00088−LabB Ploidy=NA %AC=NA MAPD=0.271 ndSNPQC=31.1
CN State
High Gain
Gain
ND
Loss
High Loss
1 2 3 4 5 6 7 8 9 10 11 12 13 14 15 16 17 18 19 20 21 22 X Y
Chromosome
TSB00088−LabC Ploidy=NA %AC=NA MAPD=0.269 ndSNPQC=30.6
CN State
High Gain
Gain
ND
Loss
High Loss
1 2 3 4 5 6 7 8 9 10 11 12 13 14 15 16 17 18 19 20 21 22 X Y
Chromosome
CN Agreement: TSB00088. GW−CN−Call−Agreement=89.4% GW−LOH−Call−Agreement=97.2%
% Agreement
100%
50%
0%
50%
100%
1 2 3 4 5 6 7 8 9 10 11 12 13 14 15 16 17 18 19 20 21 22 X Y
Chromosome

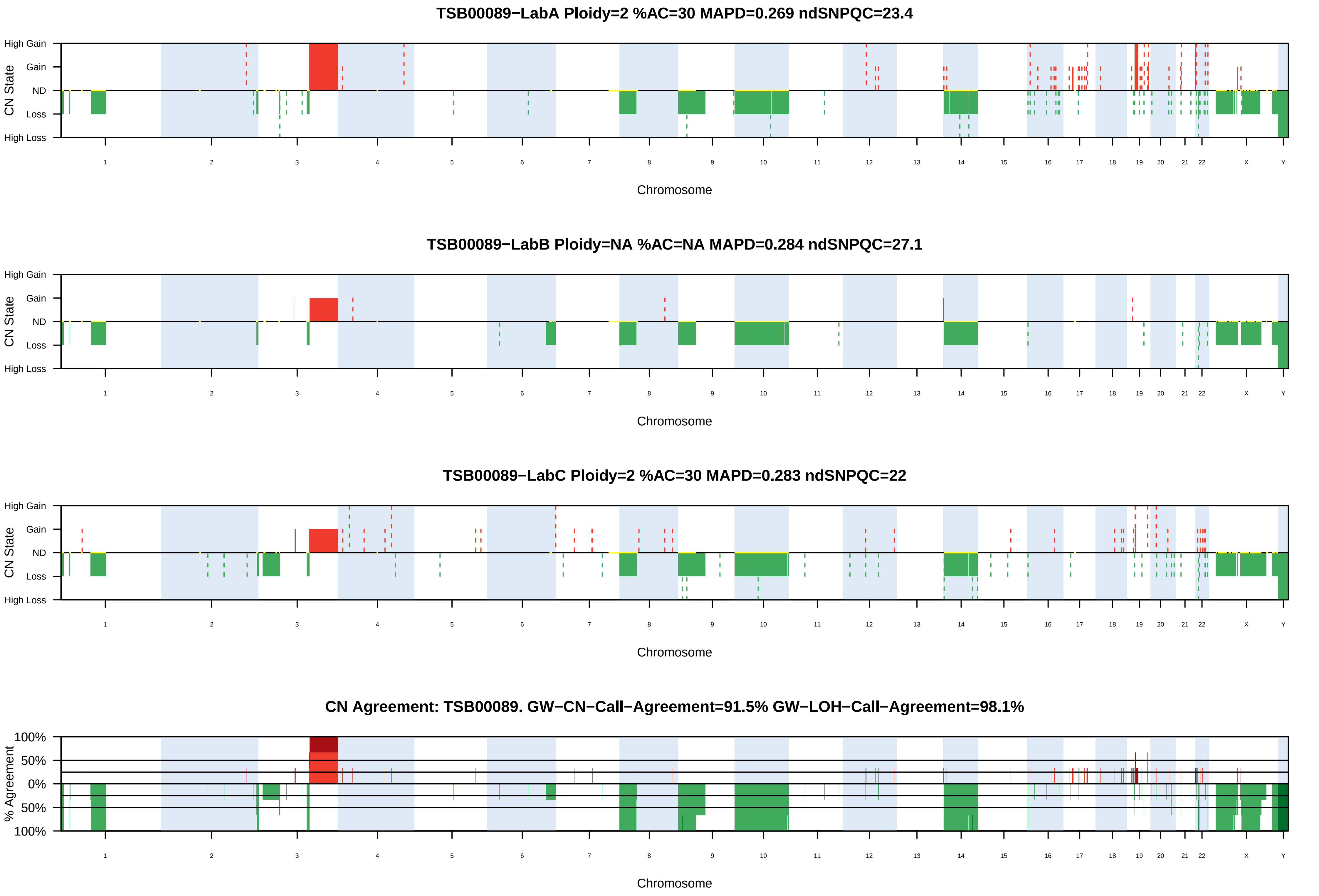
TSB00089−LabA Ploidy=2 %AC=30 MAPD=0.269 ndSNPQC=23.4
TSB00089−LabB Ploidy=NA %AC=NA MAPD=0.284 ndSNPQC=27.1
TSB00089−LabC Ploidy=2 %AC=30 MAPD=0.283 ndSNPQC=22
CN Agreement: TSB00089. GW−CN−Call−Agreement=91.5% GW−LOH−Call−Agreement=98.1%
CN State
High Gain
Gain
ND
Loss
High Loss
% Agreement
100%
50%
0%
Chromosome
1
2
3
4
5
6
7
8
9
10
11
12
13
14
15
16
17
18
19
20
21
22
X
Y

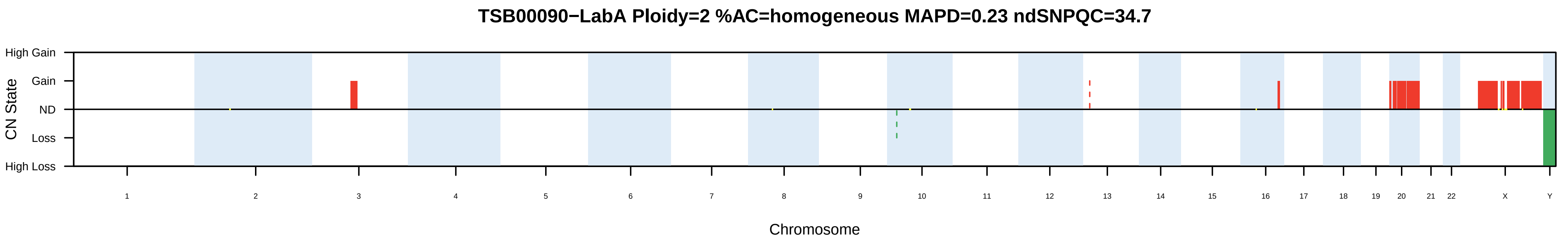
TSB00090−LabA Ploidy=2 %AC=homogeneous MAPD=0.23 ndSNPQC=34.7
CN State
High Gain
Gain
ND
Loss
High Loss
1 2 3 4 5 6 7 8 9 10 11 12 13 14 15 16 17 18 19 20 21 22 X Y
Chromosome

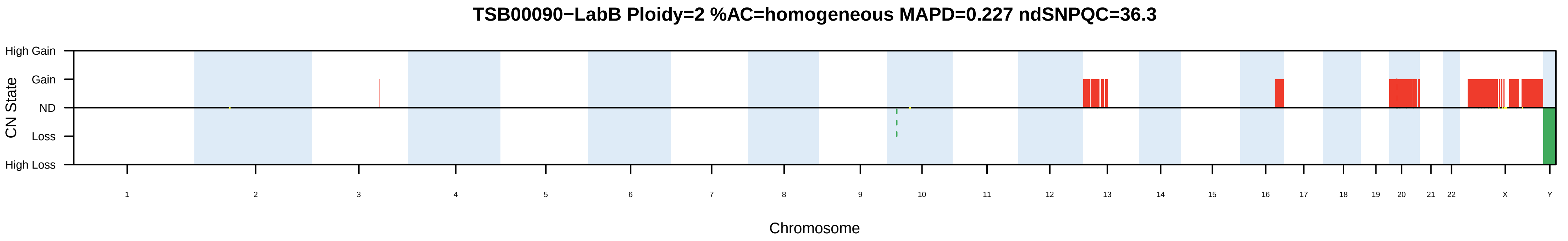
TSB00090−LabB Ploidy=2 %AC=homogeneous MAPD=0.227 ndSNPQC=36.3
CN State
High Gain
Gain
ND
Loss
High Loss
1 2 3 4 5 6 7 8 9 10 11 12 13 14 15 16 17 18 19 20 21 22 X Y
Chromosome

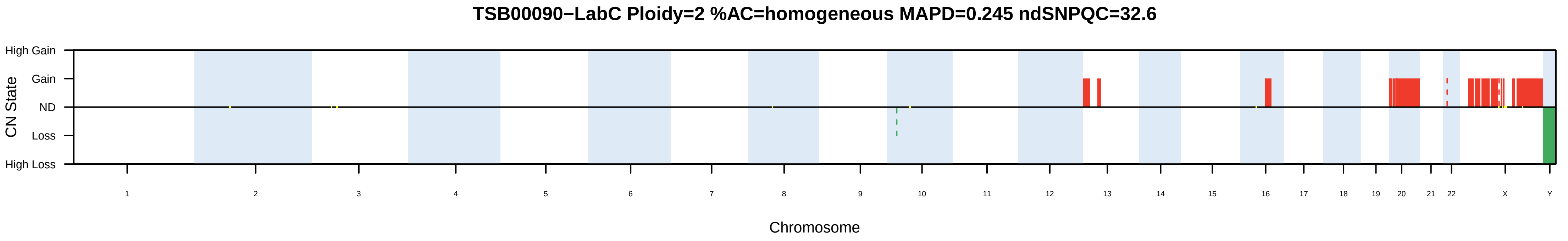
TSB00090−LabC Ploidy=2 %AC=homogeneous MAPD=0.245 ndSNPQC=32.6
CN State
High Gain
Gain
ND
Loss
High Loss
1 2 3 4 5 6 7 8 9 10 11 12 13 14 15 16 17 18 19 20 21 22 X Y
Chromosome

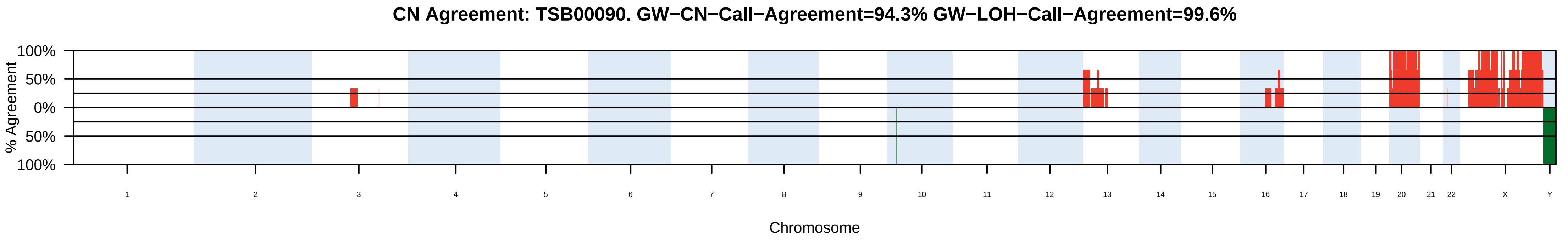
CN Agreement: TSB00090. GW−CN−Call−Agreement=94.3% GW−LOH−Call−Agreement=99.6%
% Agreement
100%
50%
0%
50%
100%
1 2 3 4 5 6 7 8 9 10 11 12 13 14 15 16 17 18 19 20 21 22 X Y
Chromosome

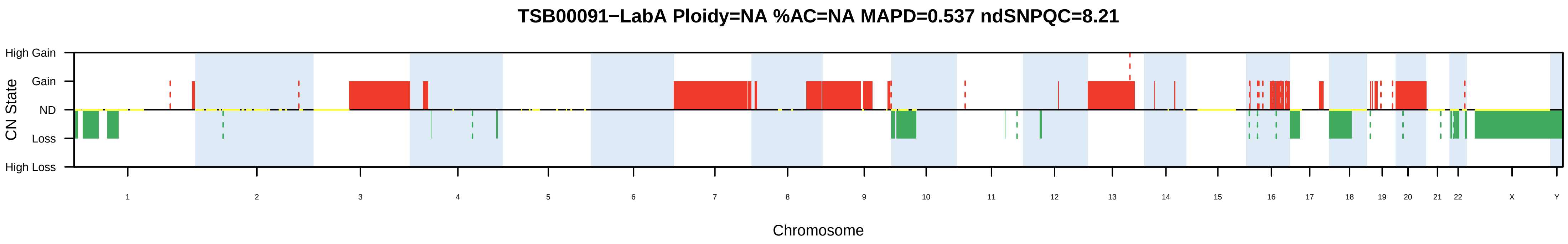
TSB00091−LabA Ploidy=NA %AC=NA MAPD=0.537 ndSNPQC=8.21
High Gain
Gain
ND
Loss
High Loss
CN State
1 2 3 4 5 6 7 8 9 10 11 12 13 14 15 16 17 18 19 20 21 22 X Y
Chromosome

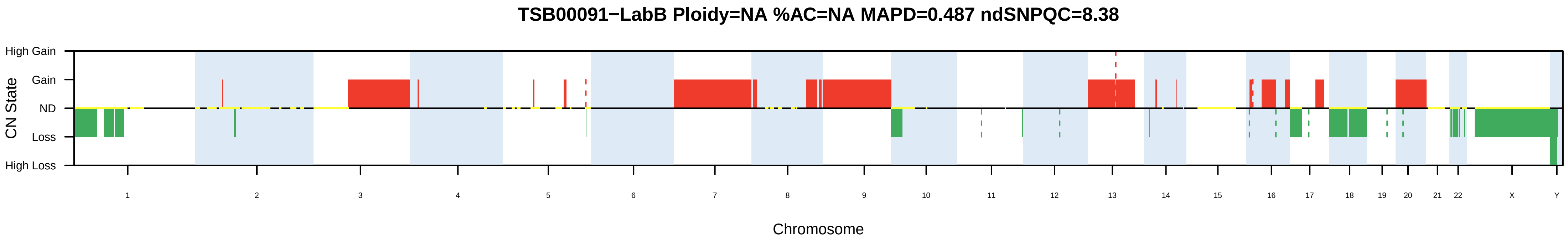
TSB00091−LabB Ploidy=NA %AC=NA MAPD=0.487 ndSNPQC=8.38
High Gain
Gain
ND
Loss
High Loss
CN State
1 2 3 4 5 6 7 8 9 10 11 12 13 14 15 16 17 18 19 20 21 22 X Y
Chromosome

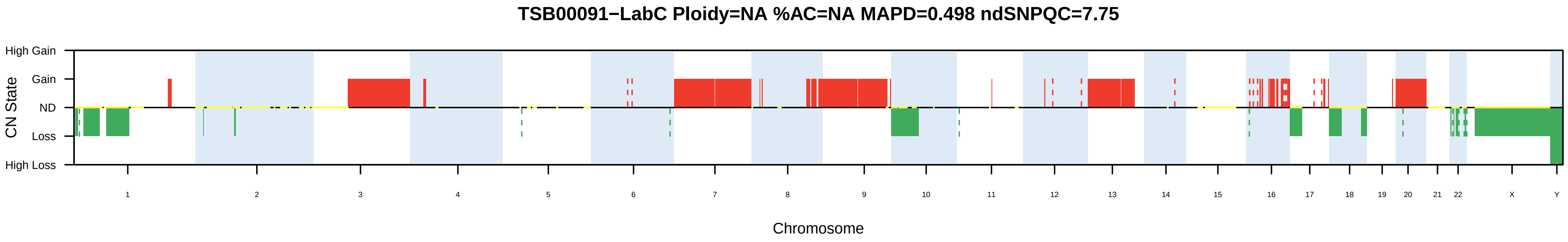
TSB00091−LabC Ploidy=NA %AC=NA MAPD=0.498 ndSNPQC=7.75
High Gain
Gain
ND
Loss
High Loss
CN State
1 2 3 4 5 6 7 8 9 10 11 12 13 14 15 16 17 18 19 20 21 22 X Y
Chromosome

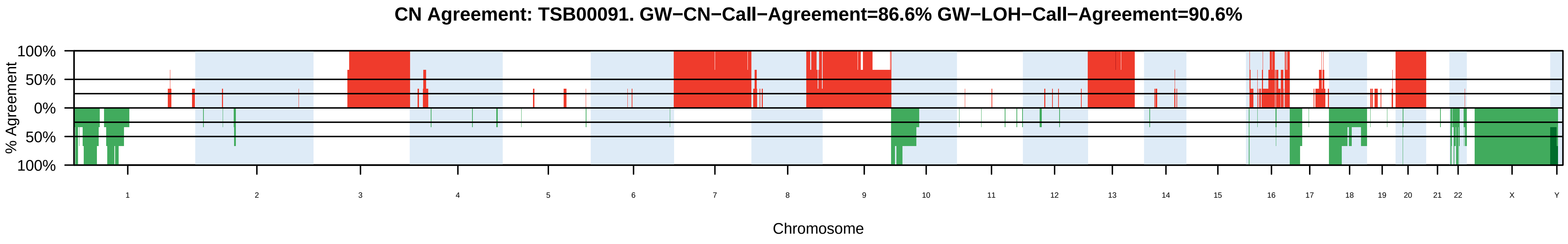
CN Agreement: TSB00091. GW−CN−Call−Agreement=86.6% GW−LOH−Call−Agreement=90.6%
100%
50%
0%
50%
100%
% Agreement
1 2 3 4 5 6 7 8 9 10 11 12 13 14 15 16 17 18 19 20 21 22 X Y
Chromosome

TSB00092−LabA Ploidy=2 %AC=homogeneous MAPD=0.218 ndSNPQC=43.4

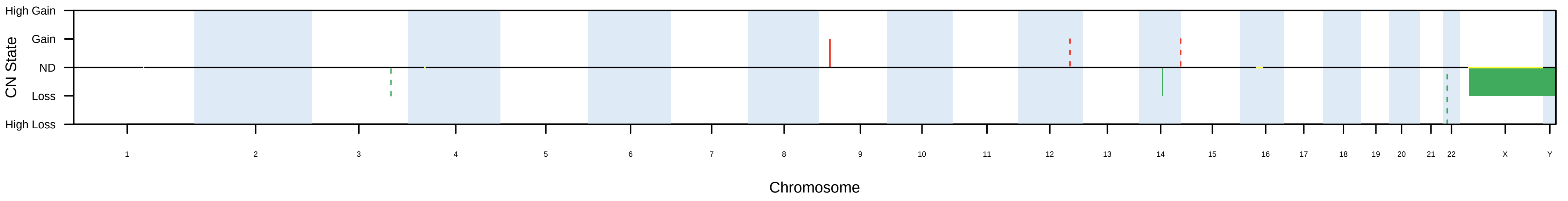

TSB00092−LabB Ploidy=2 %AC=homogeneous MAPD=0.237 ndSNPQC=42.4

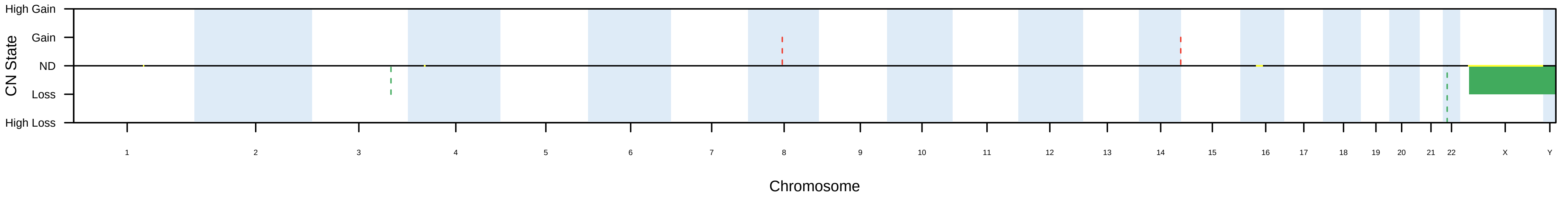

TSB00092−LabC Ploidy=2 %AC=homogeneous MAPD=0.228 ndSNPQC=40.1

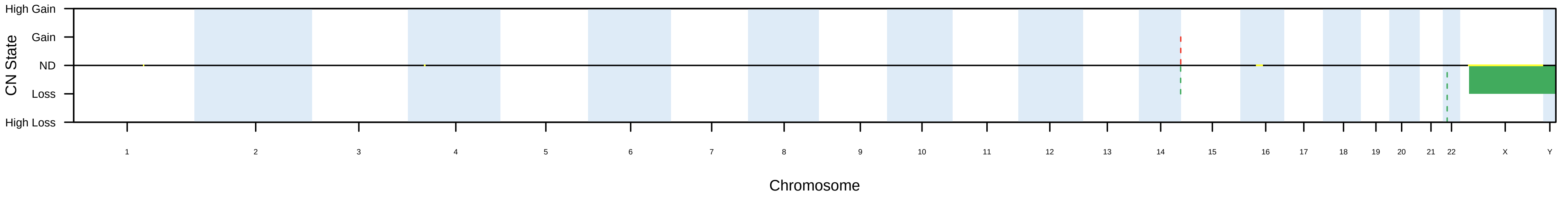

CN Agreement: TSB00092. GW−CN−Call−Agreement=99.8% GW−LOH−Call−Agreement=100%

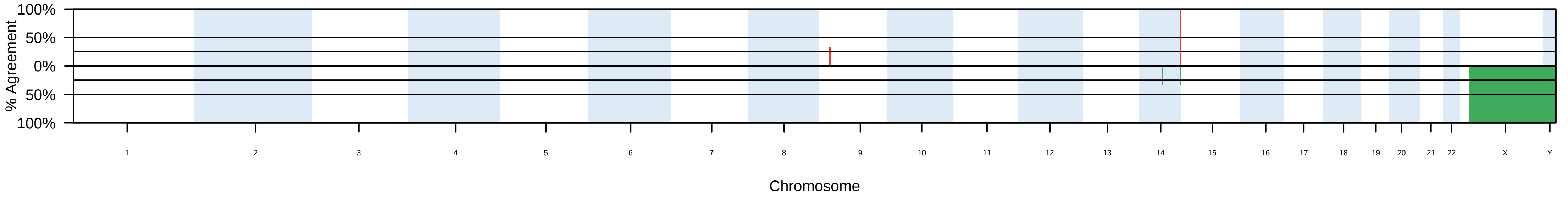

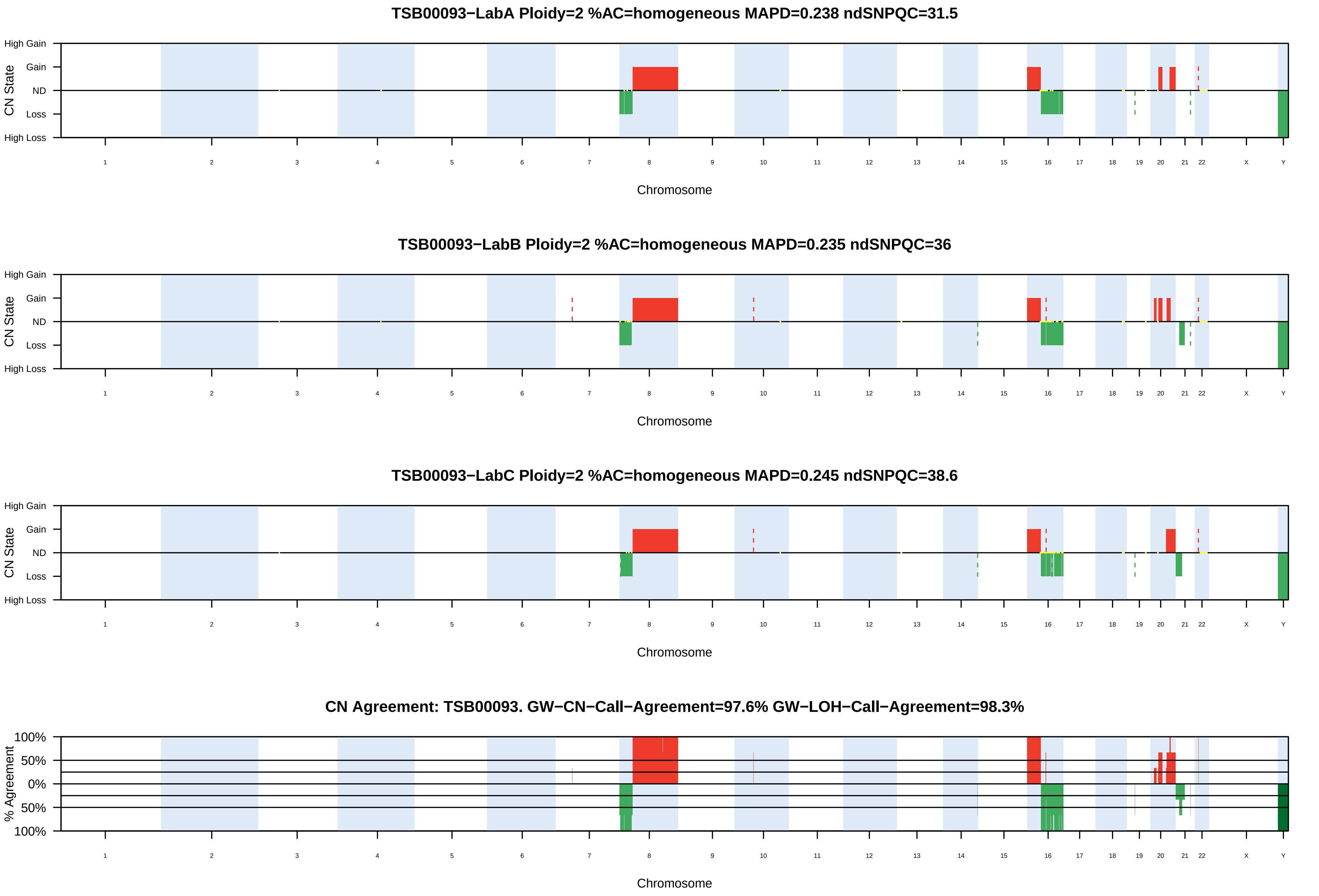
TSB00093−LabA Ploidy=2 %AC=homogeneous MAPD=0.238 ndSNPQC=31.5
TSB00093−LabB Ploidy=2 %AC=homogeneous MAPD=0.235 ndSNPQC=36
TSB00093−LabC Ploidy=2 %AC=homogeneous MAPD=0.245 ndSNPQC=38.6
CN State
High Gain
Gain
ND
Loss
High Loss
Chromosome
1
2
3
4
5
6
7
8
9
10
11
12
13
14
15
16
17
18
19
20
21
22
X
Y
CN Agreement: TSB00093. GW−CN−Call−Agreement=97.6% GW−LOH−Call−Agreement=98.3%
% Agreement
100%
50%
0%
50%
100%

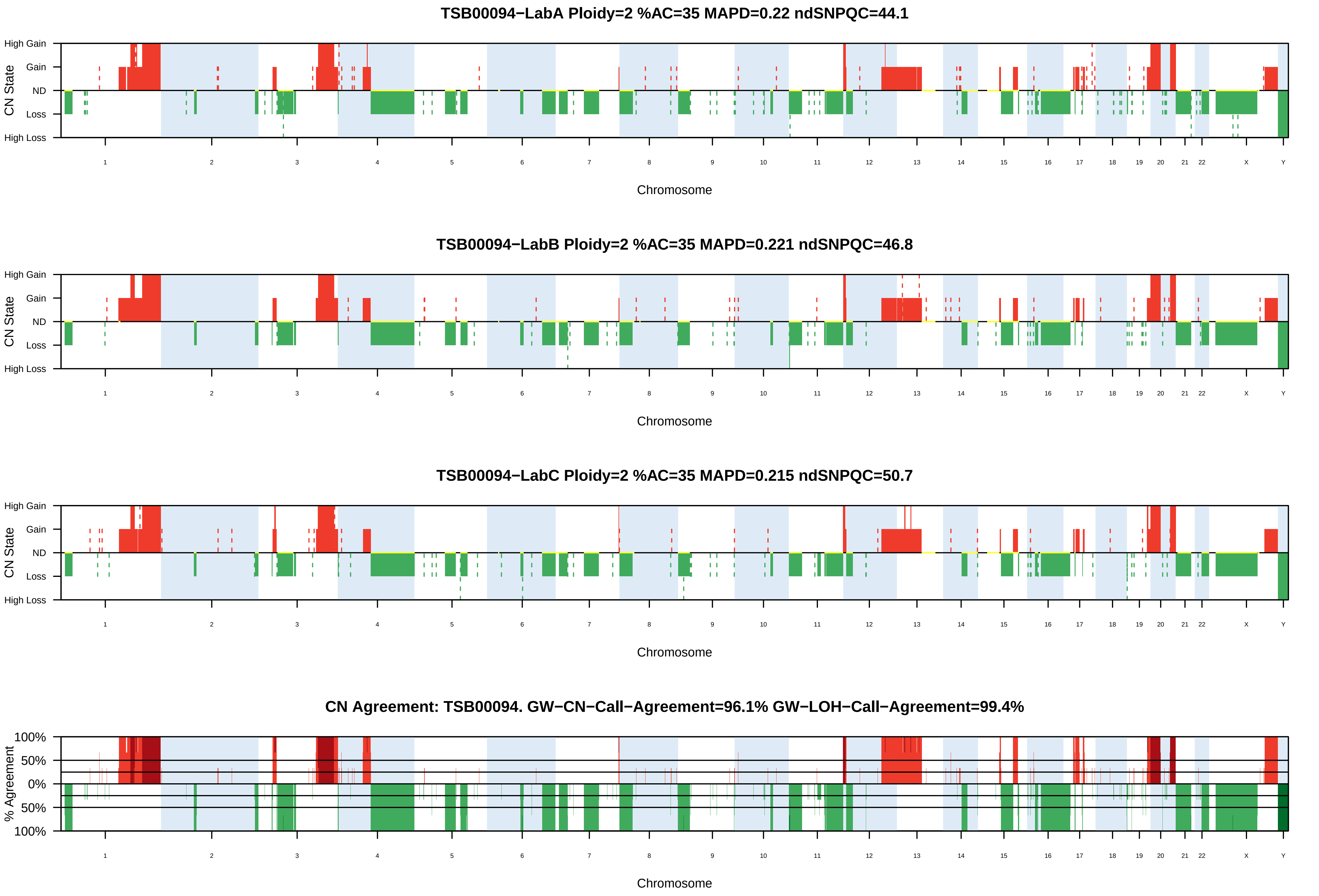
TSB00094−LabA Ploidy=2 %AC=35 MAPD=0.22 ndSNPQC=44.1
High Gain
Gain
ND
Loss
High Loss
CN State
1 2 3 4 5 6 7 8 9 10 11 12 13 14 15 16 17 18 19 20 21 22 X Y
Chromosome
TSB00094−LabB Ploidy=2 %AC=35 MAPD=0.221 ndSNPQC=46.8
High Gain
Gain
ND
Loss
High Loss
CN State
1 2 3 4 5 6 7 8 9 10 11 12 13 14 15 16 17 18 19 20 21 22 X Y
Chromosome
TSB00094−LabC Ploidy=2 %AC=35 MAPD=0.215 ndSNPQC=50.7
High Gain
Gain
ND
Loss
High Loss
CN State
1 2 3 4 5 6 7 8 9 10 11 12 13 14 15 16 17 18 19 20 21 22 X Y
Chromosome
CN Agreement: TSB00094. GW−CN−Call−Agreement=96.1% GW−LOH−Call−Agreement=99.4%
100%
50%
0%
50%
100%
% Agreement
1 2 3 4 5 6 7 8 9 10 11 12 13 14 15 16 17 18 19 20 21 22 X Y
Chromosome

TSB00095−LabA Ploidy=2 %AC=40 MAPD=0.219 ndSNPQC=44.4

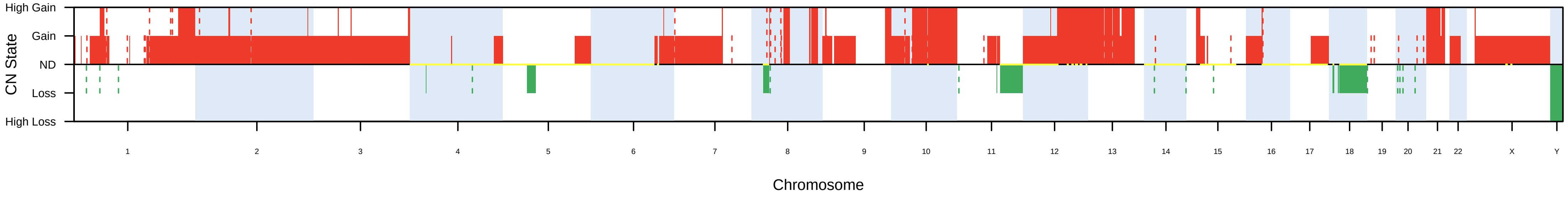

TSB00095−LabB Ploidy=2 %AC=40 MAPD=0.223 ndSNPQC=44.1

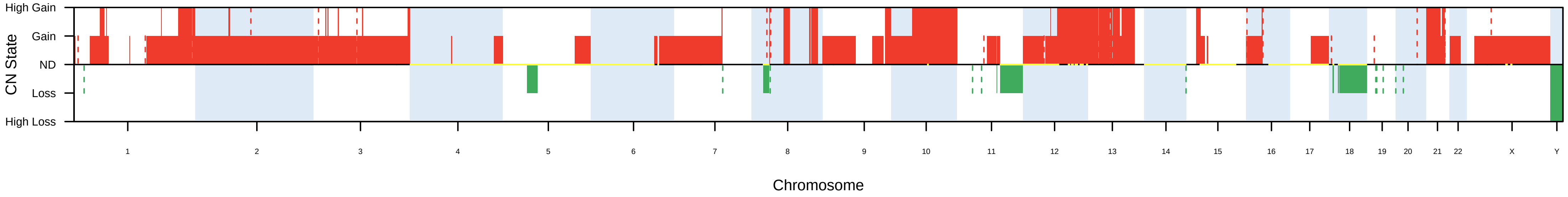

TSB00095−LabC Ploidy=2 %AC=45 MAPD=0.208 ndSNPQC=48.3

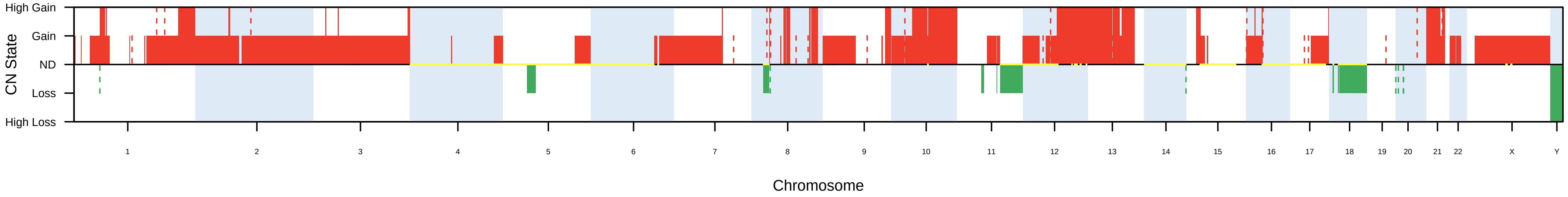

CN Agreement: TSB00095. GW−CN−Call−Agreement=95.8% GW−LOH−Call−Agreement=98.5%

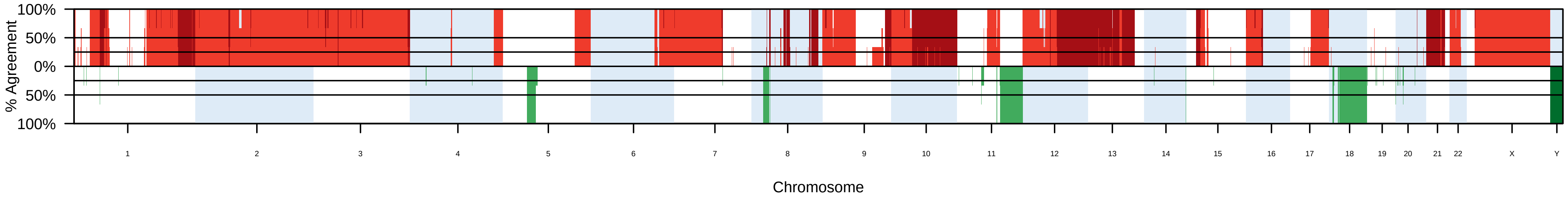

TSB00096−LabA Ploidy=2 %AC=homogeneous MAPD=0.272 ndSNPQC=26.2

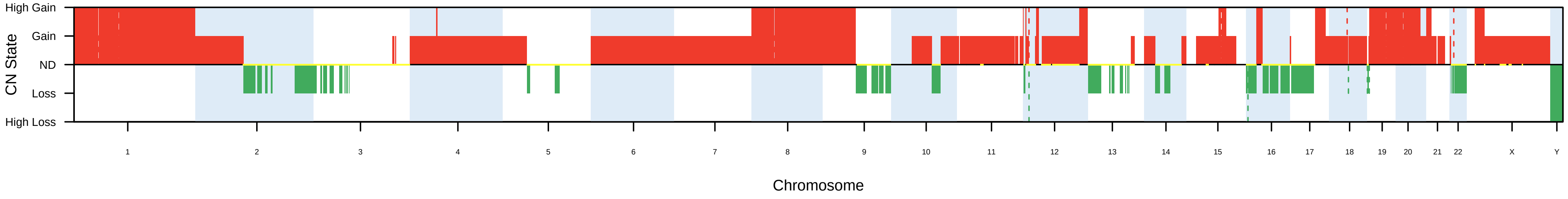

TSB00096−LabB Ploidy=2 %AC=70 MAPD=0.257 ndSNPQC=27.7

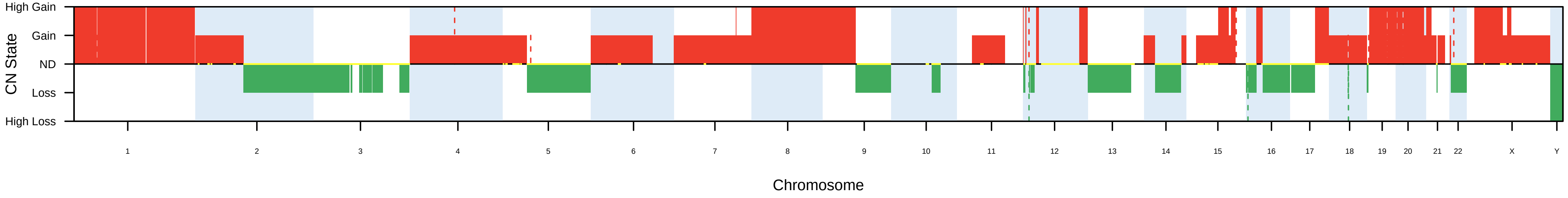

TSB00096−LabC Ploidy=2 %AC=75 MAPD=0.277 ndSNPQC=31.8

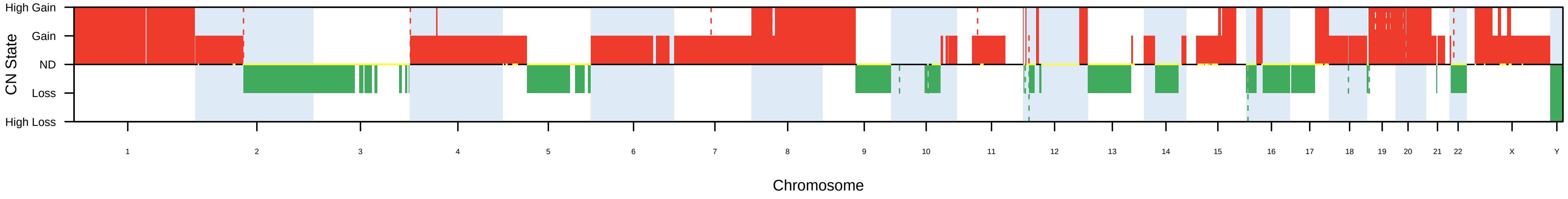

CN Agreement: TSB00096. GW−CN−Call−Agreement=71% GW−LOH−Call−Agreement=95.3%

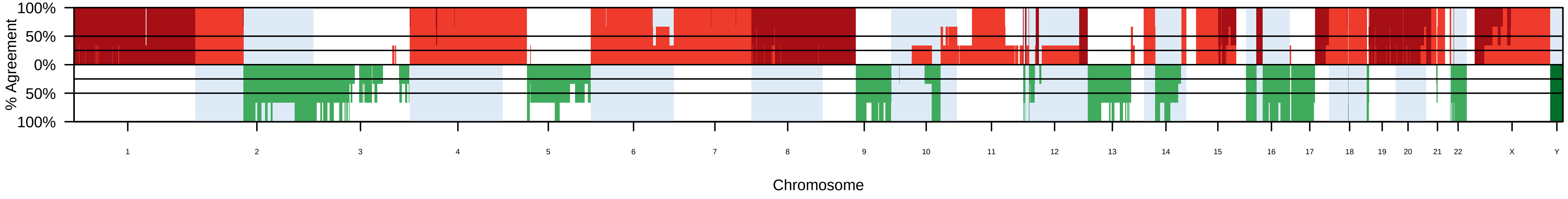

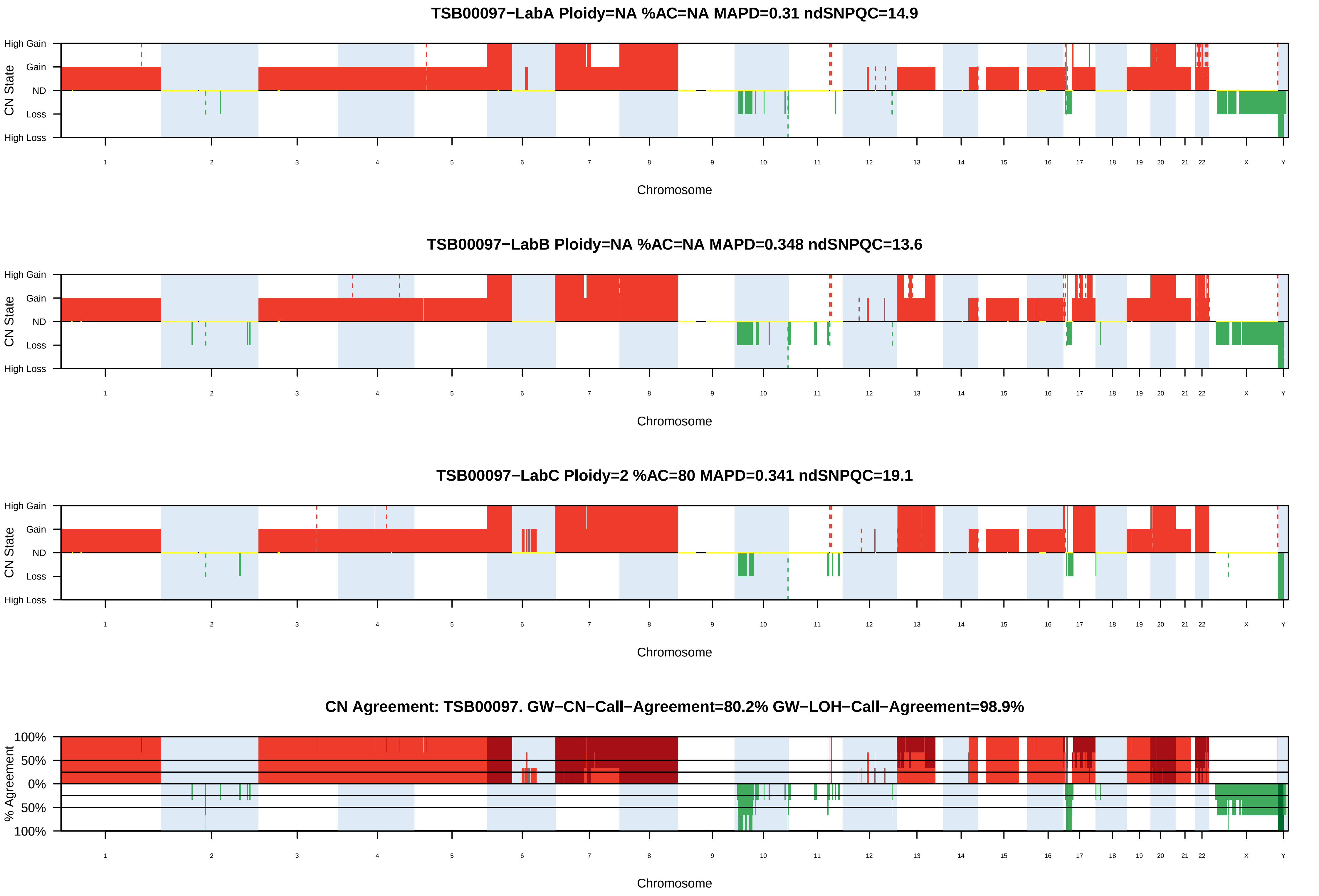
TSB00097−LabA Ploidy=NA %AC=NA MAPD=0.31 ndSNPQC=14.9
High Gain
Gain
ND
Loss
High Loss
CN State
1 2 3 4 5 6 7 8 9 10 11 12 13 14 15 16 17 18 19 20 21 22 X Y
Chromosome
TSB00097−LabB Ploidy=NA %AC=NA MAPD=0.348 ndSNPQC=13.6
High Gain
Gain
ND
Loss
High Loss
CN State
1 2 3 4 5 6 7 8 9 10 11 12 13 14 15 16 17 18 19 20 21 22 X Y
Chromosome
TSB00097−LabC Ploidy=2 %AC=80 MAPD=0.341 ndSNPQC=19.1
High Gain
Gain
ND
Loss
High Loss
CN State
1 2 3 4 5 6 7 8 9 10 11 12 13 14 15 16 17 18 19 20 21 22 X Y
Chromosome
CN Agreement: TSB00097. GW−CN−Call−Agreement=80.2% GW−LOH−Call−Agreement=98.9%
100%
50%
0%
50%
100%
% Agreement
1 2 3 4 5 6 7 8 9 10 11 12 13 14 15 16 17 18 19 20 21 22 X Y
Chromosome

TSB00098−LabA Ploidy=NA %AC=NA MAPD=0.261 ndSNPQC=27.6

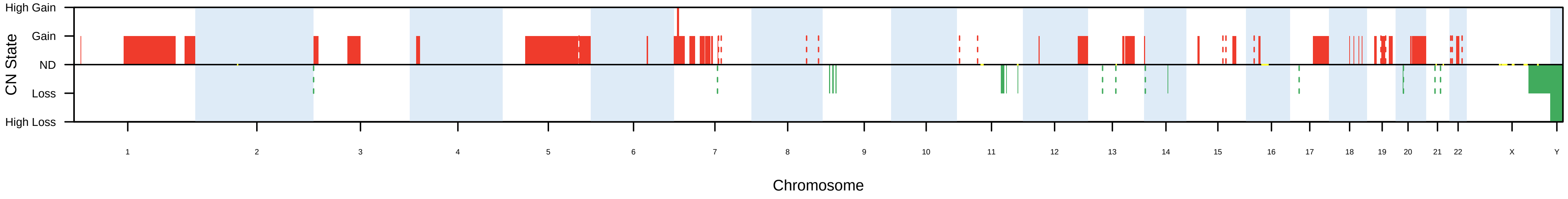

TSB00098−LabB Ploidy=NA %AC=NA MAPD=0.285 ndSNPQC=26.1

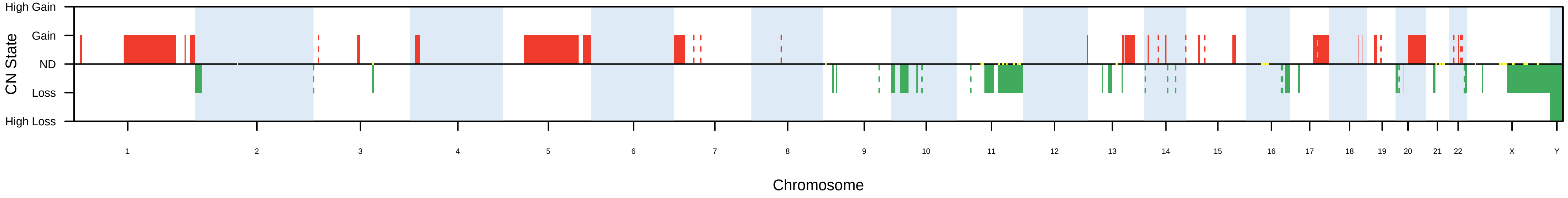

TSB00098−LabC Ploidy=NA %AC=NA MAPD=0.269 ndSNPQC=30.4

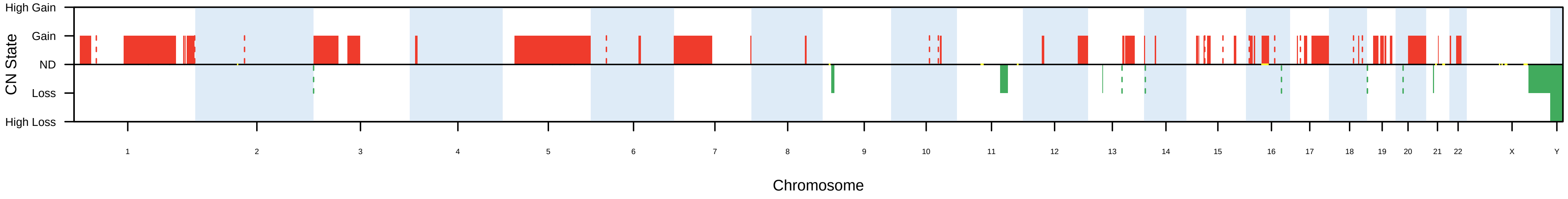

CN Agreement: TSB00098. GW−CN−Call−Agreement=80.3% GW−LOH−Call−Agreement=98%

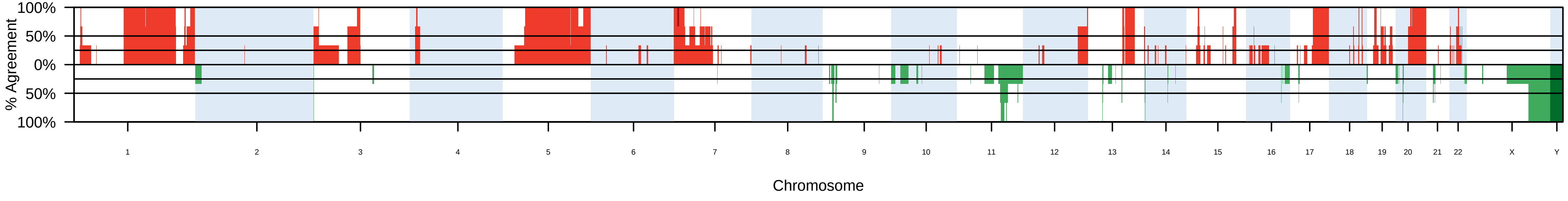

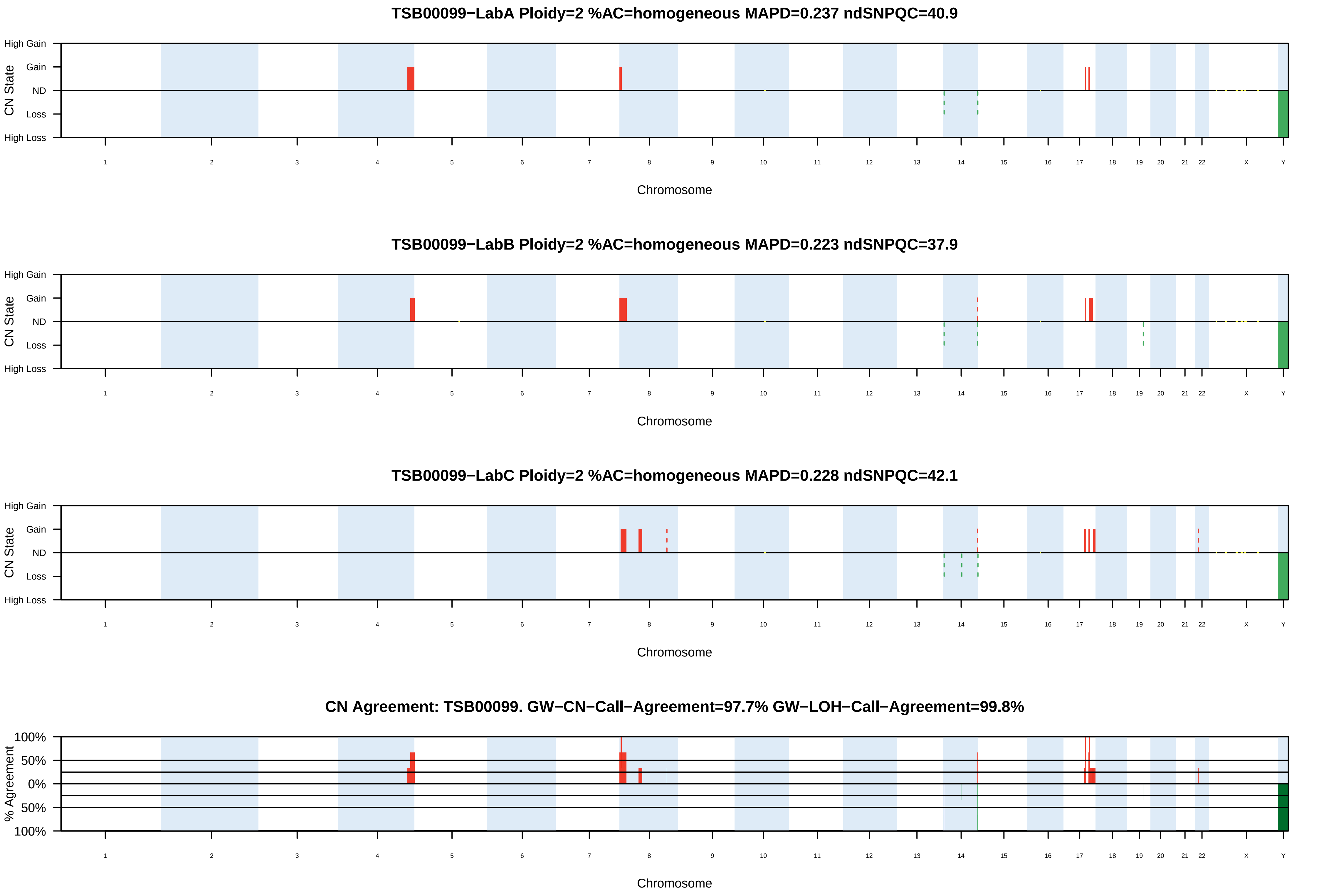

TSB00099−LabA Ploidy=2 %AC=homogeneous MAPD=0.237 ndSNPQC=40.9
High Gain
Gain
ND
Loss
High Loss
CN State
1 2 3 4 5 6 7 8 9 10 11 12 13 14 15 16 17 18 19 20 21 22 X Y
Chromosome
TSB00099−LabB Ploidy=2 %AC=homogeneous MAPD=0.223 ndSNPQC=37.9
High Gain
Gain
ND
Loss
High Loss
CN State
1 2 3 4 5 6 7 8 9 10 11 12 13 14 15 16 17 18 19 20 21 22 X Y
Chromosome
TSB00099−LabC Ploidy=2 %AC=homogeneous MAPD=0.228 ndSNPQC=42.1
High Gain
Gain
ND
Loss
High Loss
CN State
1 2 3 4 5 6 7 8 9 10 11 12 13 14 15 16 17 18 19 20 21 22 X Y
Chromosome
CN Agreement: TSB00099. GW−CN−Call−Agreement=97.7% GW−LOH−Call−Agreement=99.8%
100%
50%
0%
50%
100%
% Agreement
1 2 3 4 5 6 7 8 9 10 11 12 13 14 15 16 17 18 19 20 21 22 X Y
Chromosome

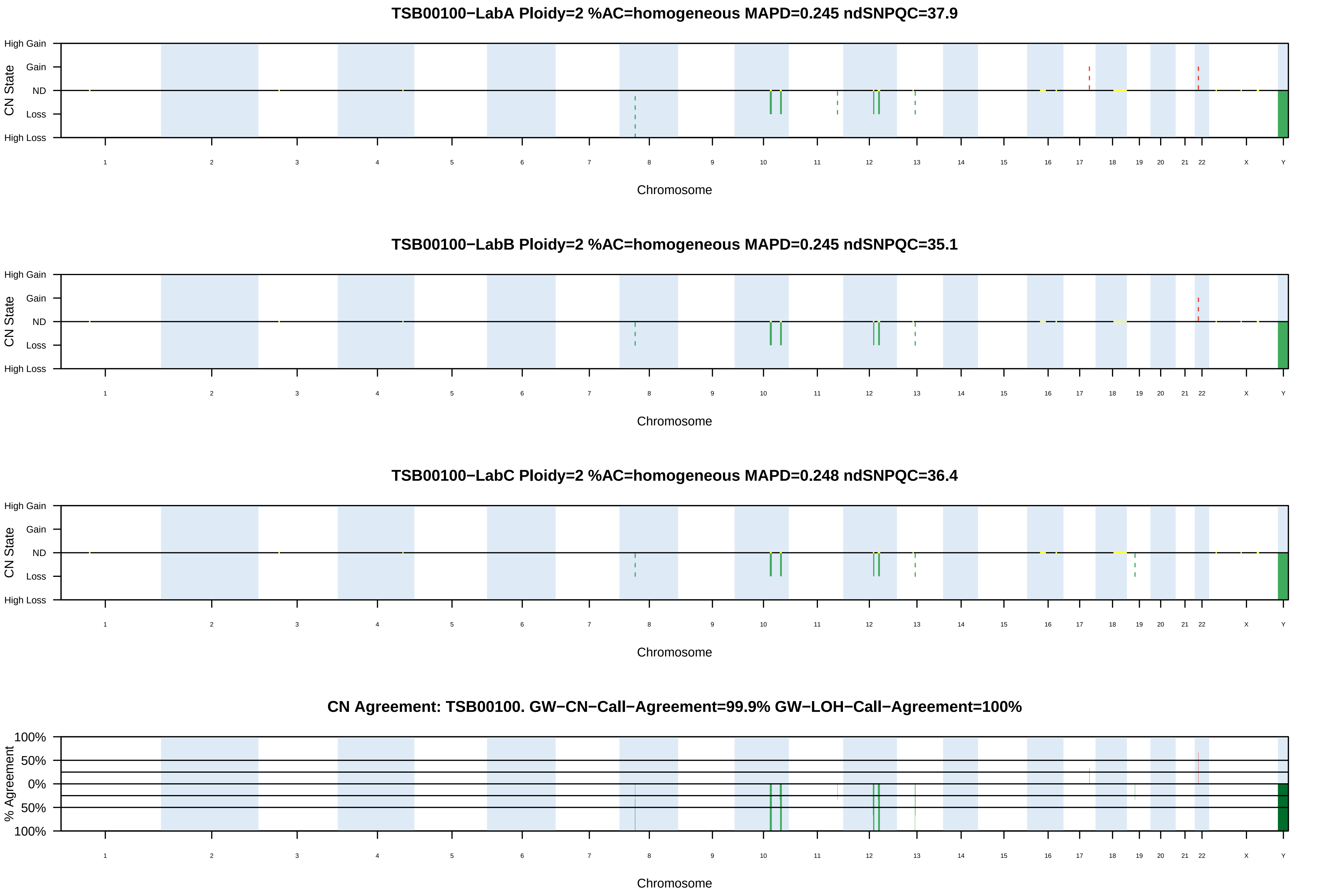

TSB00100−LabA Ploidy=2 %AC=homogeneous MAPD=0.245 ndSNPQC=37.9
TSB00100−LabB Ploidy=2 %AC=homogeneous MAPD=0.245 ndSNPQC=35.1
TSB00100−LabC Ploidy=2 %AC=homogeneous MAPD=0.248 ndSNPQC=36.4
CN State
High Gain
Gain
ND
Loss
High Loss
Chromosome
1
2
3
4
5
6
7
8
9
10
11
12
13
14
15
16
17
18
19
20
21
22
X
Y
CN Agreement: TSB00100. GW−CN−Call−Agreement=99.9% GW−LOH−Call−Agreement=100%
% Agreement
100%
50%
0%
50%
100%

TSB00101−LabA Ploidy=NA %AC=NA MAPD=0.234 ndSNPQC=40.4

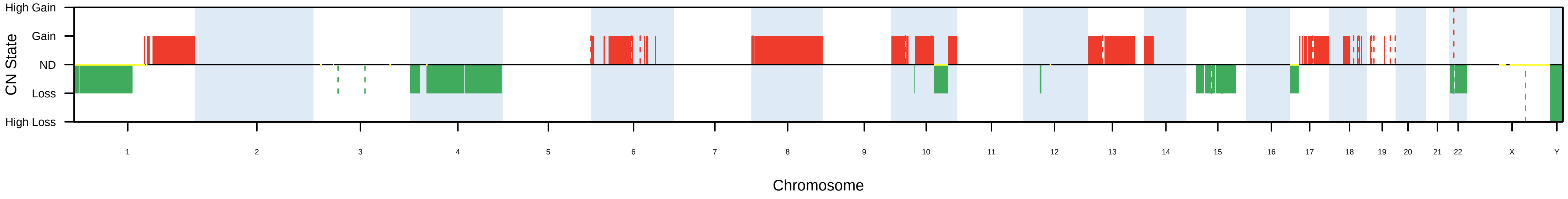

TSB00101−LabB Ploidy=NA %AC=NA MAPD=0.232 ndSNPQC=38.3

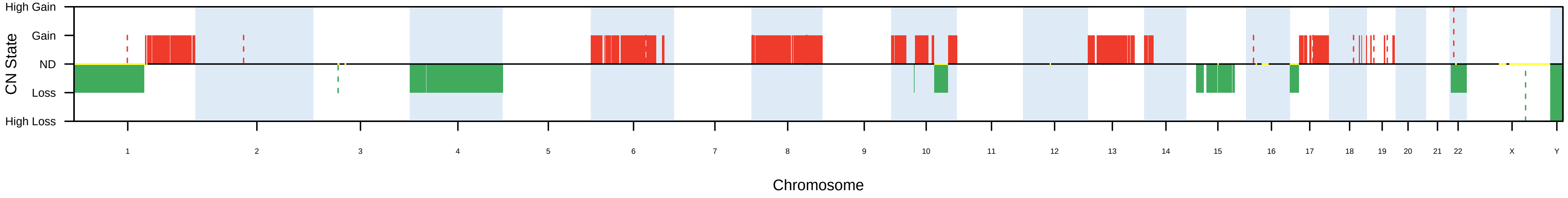

TSB00101−LabC Ploidy=NA %AC=NA MAPD=0.232 ndSNPQC=40.1

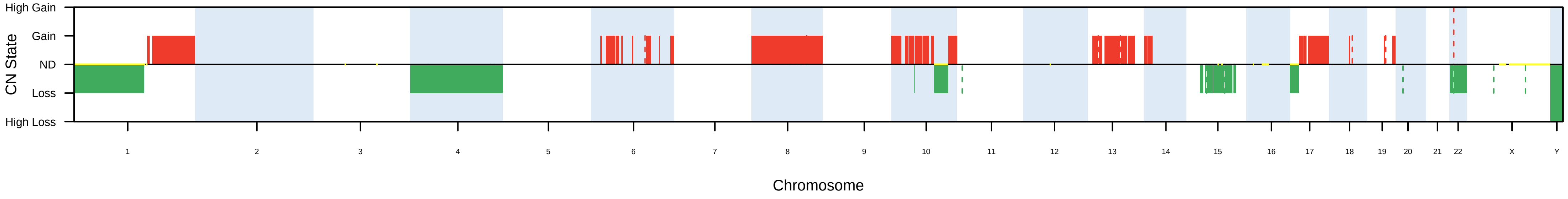

CN Agreement: TSB00101. GW−CN−Call−Agreement=88.9% GW−LOH−Call−Agreement=98.3%

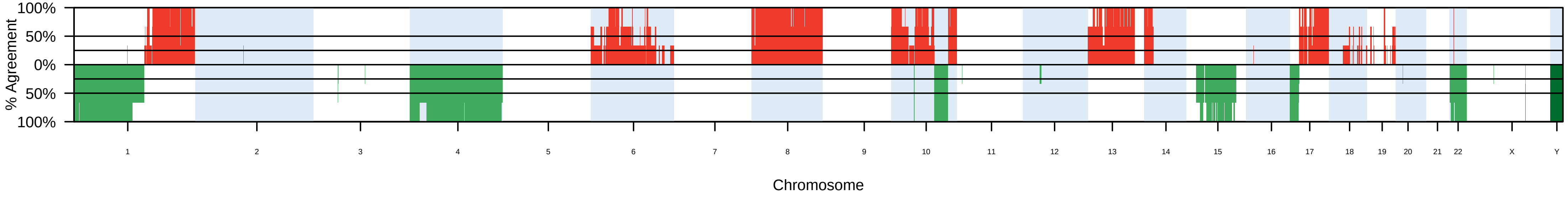

TSB00102−LabA Ploidy=2 %AC=40 MAPD=0.255 ndSNPQC=32.7

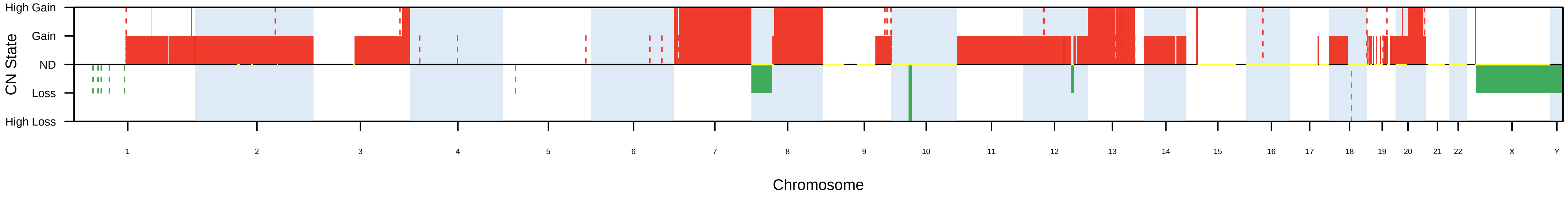

TSB00102−LabB Ploidy=2 %AC=45 MAPD=0.266 ndSNPQC=28

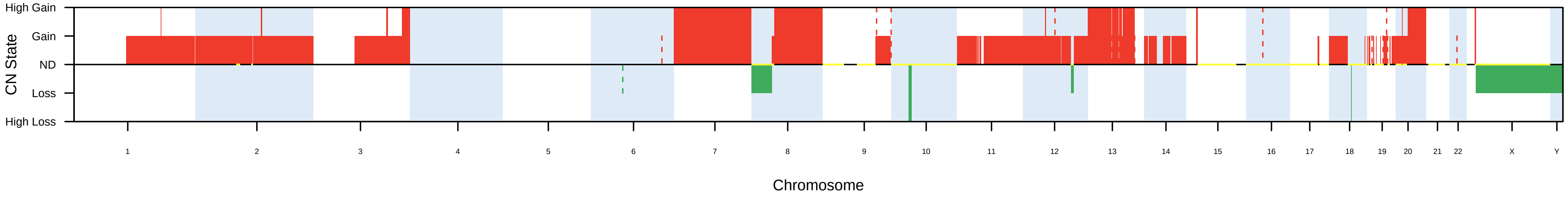

TSB00102−LabC Ploidy=2 %AC=40 MAPD=0.277 ndSNPQC=26.4

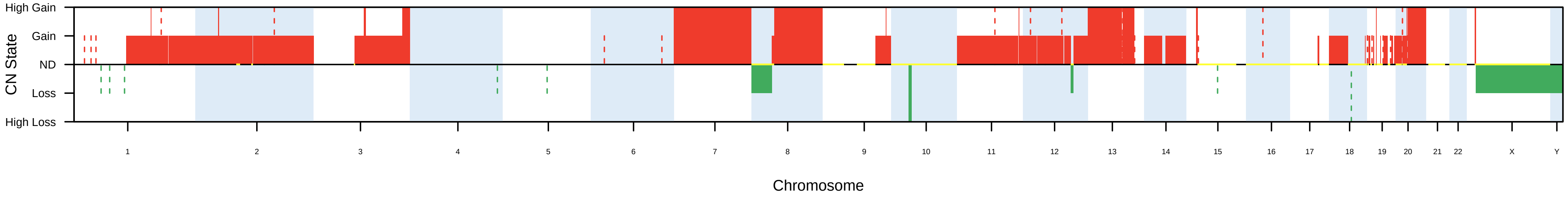

CN Agreement: TSB00102. GW−CN−Call−Agreement=96.1% GW−LOH−Call−Agreement=99.6%

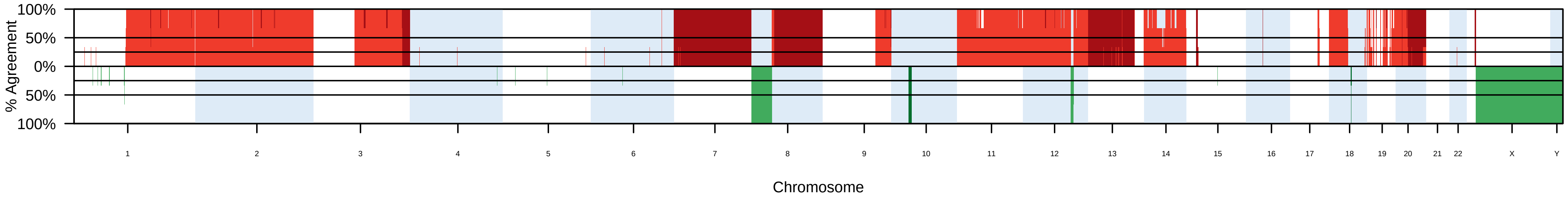

TSB00103−LabA Ploidy=2 %AC=homogeneous MAPD=0.237 ndSNPQC=27.1

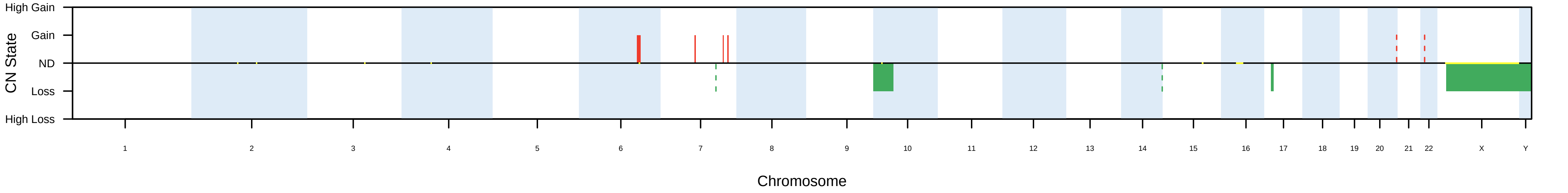

TSB00103−LabB Ploidy=2 %AC=homogeneous MAPD=0.262 ndSNPQC=22.7

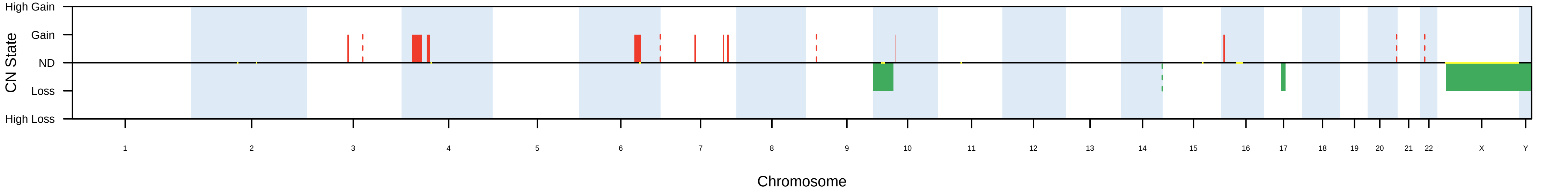

TSB00103−LabC Ploidy=2 %AC=homogeneous MAPD=0.252 ndSNPQC=25.5

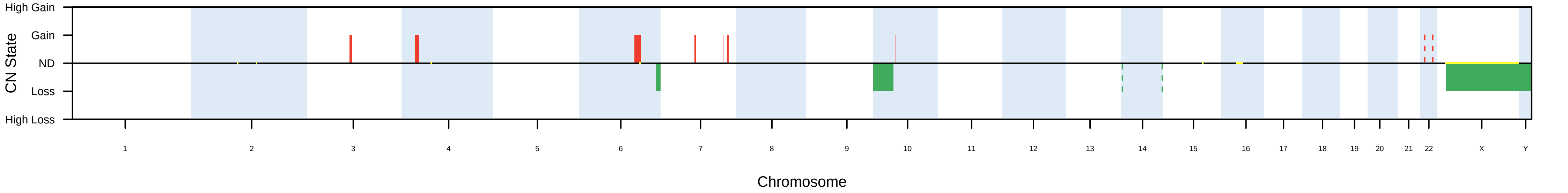

CN Agreement: TSB00103. GW−CN−Call−Agreement=97.4% GW−LOH−Call−Agreement=99.6%

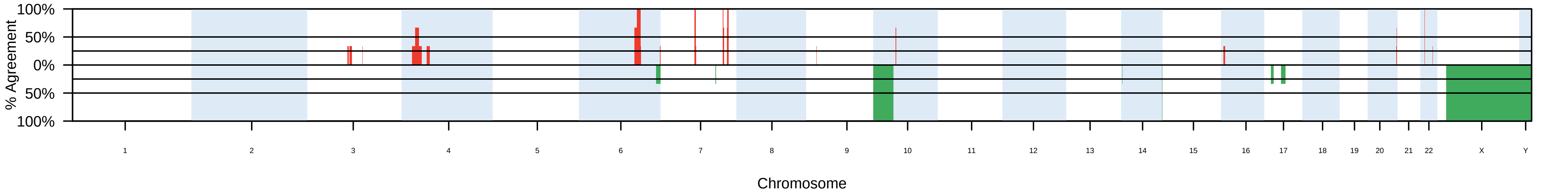

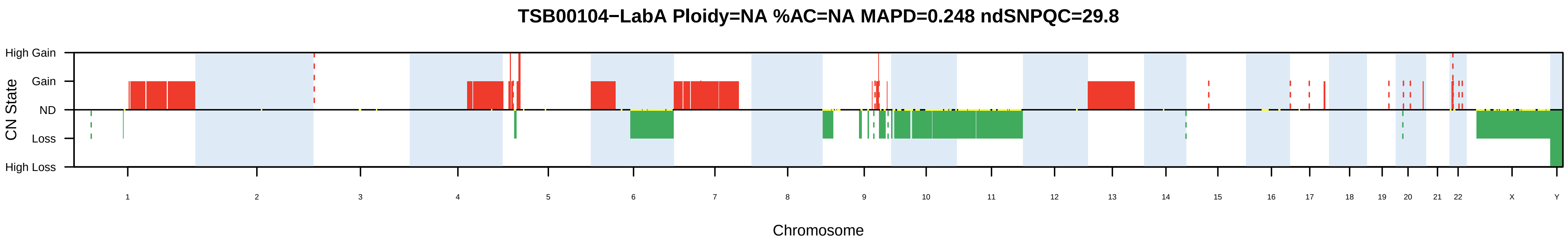
TSB00104−LabA Ploidy=NA %AC=NA MAPD=0.248 ndSNPQC=29.8
CN State
High Gain
Gain
ND
Loss
High Loss
1 2 3 4 5 6 7 8 9 10 11 12 13 14 15 16 17 18 19 20 21 22 X Y
Chromosome

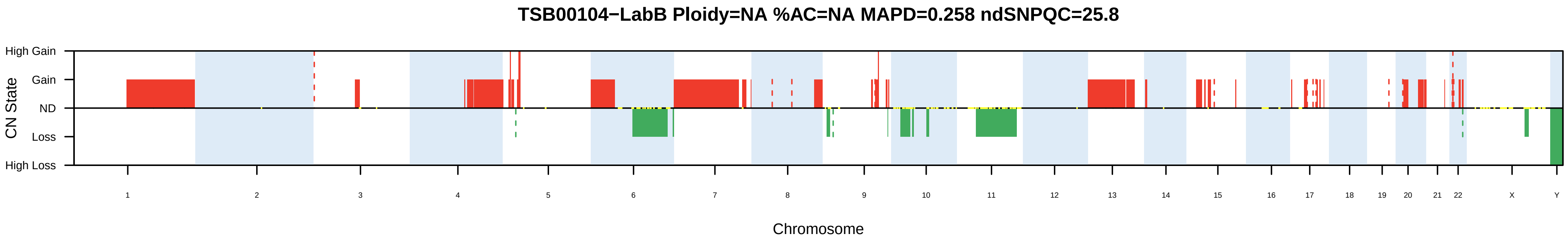
TSB00104−LabB Ploidy=NA %AC=NA MAPD=0.258 ndSNPQC=25.8
CN State
High Gain
Gain
ND
Loss
High Loss
1 2 3 4 5 6 7 8 9 10 11 12 13 14 15 16 17 18 19 20 21 22 X Y
Chromosome

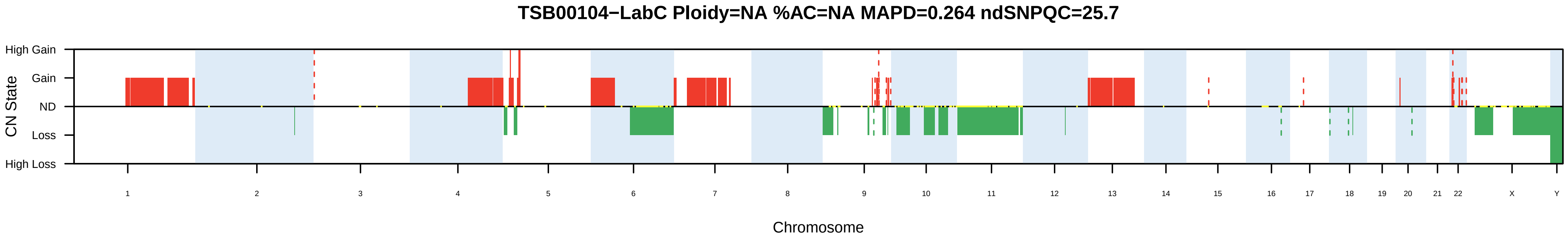
TSB00104−LabC Ploidy=NA %AC=NA MAPD=0.264 ndSNPQC=25.7
CN State
High Gain
Gain
ND
Loss
High Loss
1 2 3 4 5 6 7 8 9 10 11 12 13 14 15 16 17 18 19 20 21 22 X Y
Chromosome

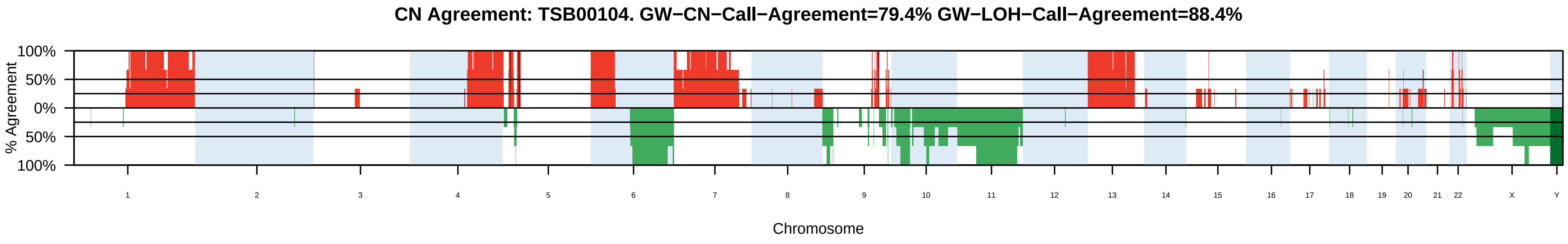
CN Agreement: TSB00104. GW−CN−Call−Agreement=79.4% GW−LOH−Call−Agreement=88.4%
% Agreement
100%
50%
0%
50%
100%
1 2 3 4 5 6 7 8 9 10 11 12 13 14 15 16 17 18 19 20 21 22 X Y
Chromosome

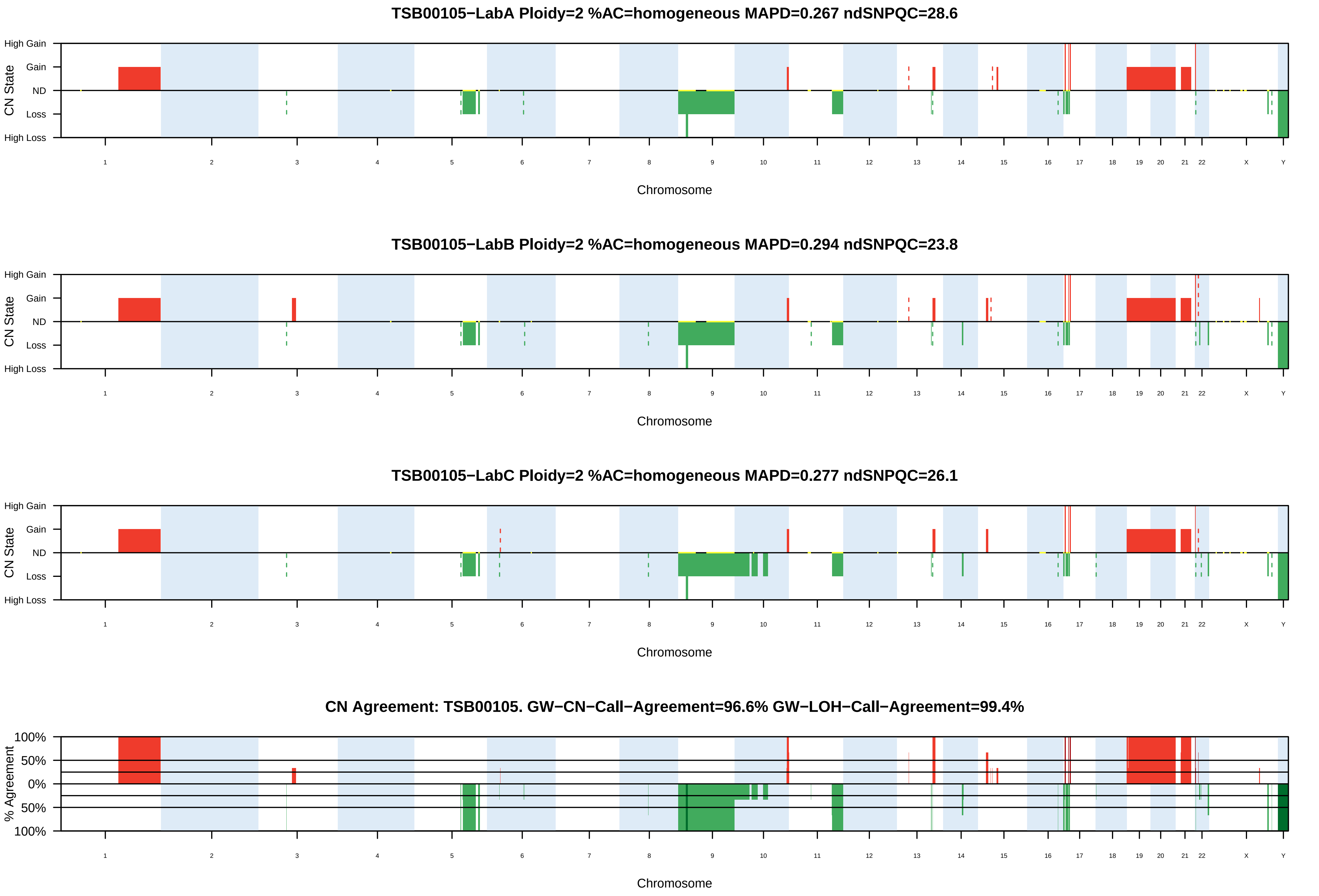
TSB00105−LabA Ploidy=2 %AC=homogeneous MAPD=0.267 ndSNPQC=28.6
CN State
High Gain
Gain
ND
Loss
High Loss
1
2
3
4
5
6
7
8
9
10
11
12
13
14
15
16
17
18
19
20
21
22
X
Y
Chromosome
TSB00105−LabB Ploidy=2 %AC=homogeneous MAPD=0.294 ndSNPQC=23.8
CN State
Chromosome
TSB00105−LabC Ploidy=2 %AC=homogeneous MAPD=0.277 ndSNPQC=26.1
CN State
Chromosome
CN Agreement: TSB00105. GW−CN−Call−Agreement=96.6% GW−LOH−Call−Agreement=99.4%
% Agreement
100%
50%
0%
50%
100%
Chromosome

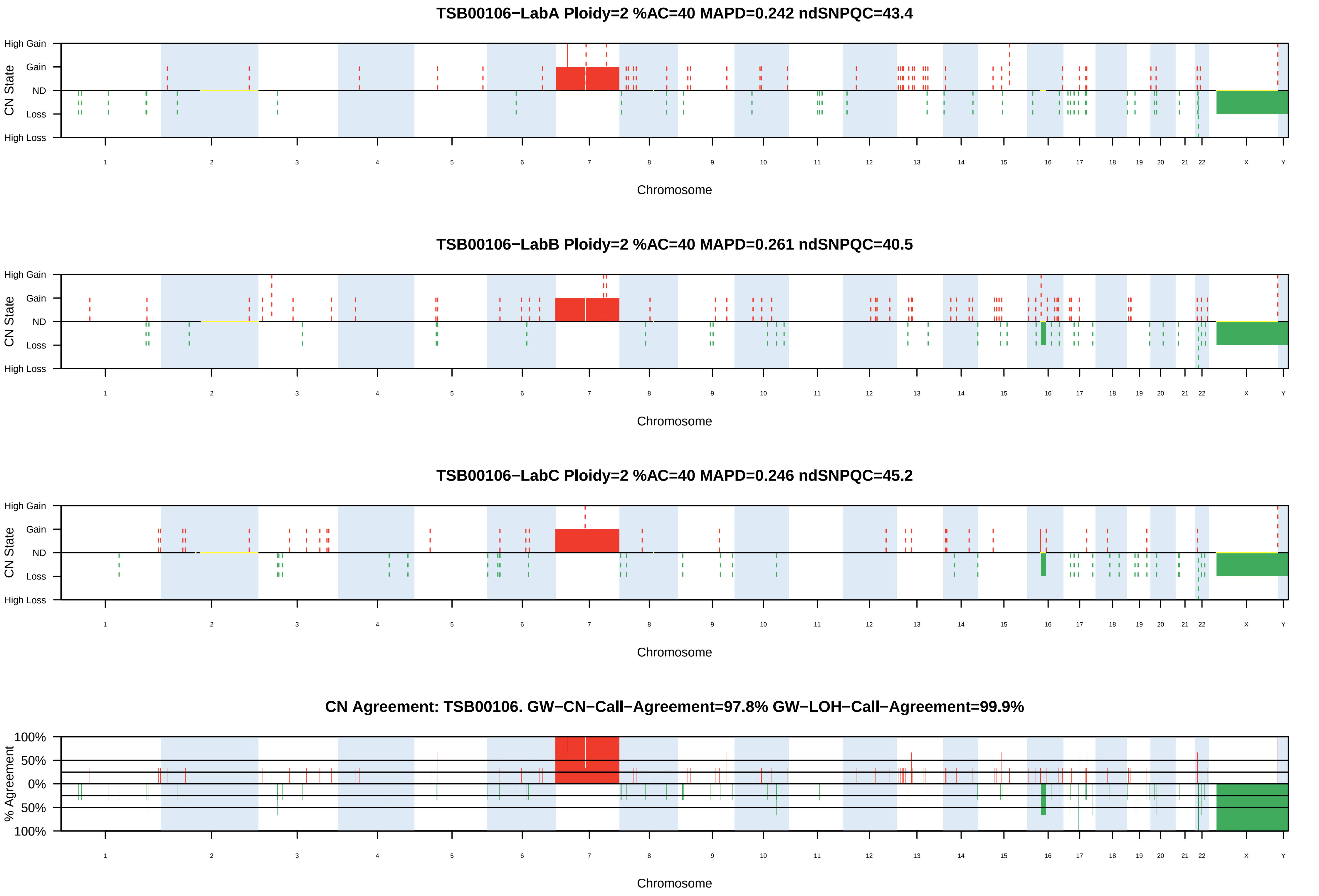

TSB00106−LabA Ploidy=2 %AC=40 MAPD=0.242 ndSNPQC=43.4
CN State
High Gain
Gain
ND
Loss
High Loss
1 2 3 4 5 6 7 8 9 10 11 12 13 14 15 16 17 18 19 20 21 22 X Y
Chromosome
TSB00106−LabB Ploidy=2 %AC=40 MAPD=0.261 ndSNPQC=40.5
CN State
High Gain
Gain
ND
Loss
High Loss
1 2 3 4 5 6 7 8 9 10 11 12 13 14 15 16 17 18 19 20 21 22 X Y
Chromosome
TSB00106−LabC Ploidy=2 %AC=40 MAPD=0.246 ndSNPQC=45.2
CN State
High Gain
Gain
ND
Loss
High Loss
1 2 3 4 5 6 7 8 9 10 11 12 13 14 15 16 17 18 19 20 21 22 X Y
Chromosome
CN Agreement: TSB00106. GW−CN−Call−Agreement=97.8% GW−LOH−Call−Agreement=99.9%
% Agreement
100%
50%
0%
50%
100%
1 2 3 4 5 6 7 8 9 10 11 12 13 14 15 16 17 18 19 20 21 22 X Y
Chromosome

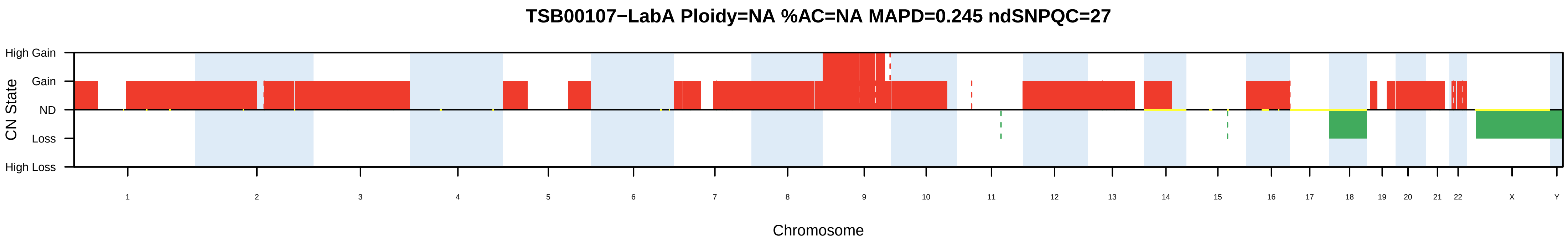
TSB00107−LabA Ploidy=NA %AC=NA MAPD=0.245 ndSNPQC=27
High Gain
Gain
ND
Loss
High Loss
CN State
1 2 3 4 5 6 7 8 9 10 11 12 13 14 15 16 17 18 19 20 21 22 X Y
Chromosome

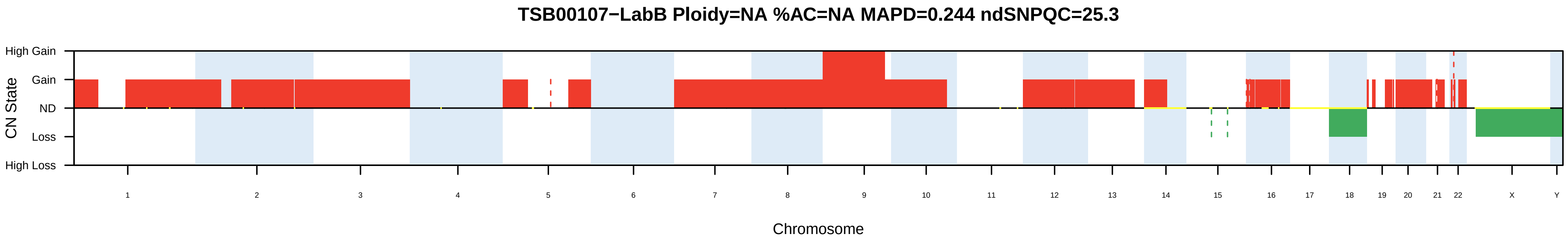
TSB00107−LabB Ploidy=NA %AC=NA MAPD=0.244 ndSNPQC=25.3
High Gain
Gain
ND
Loss
High Loss
CN State
1 2 3 4 5 6 7 8 9 10 11 12 13 14 15 16 17 18 19 20 21 22 X Y
Chromosome

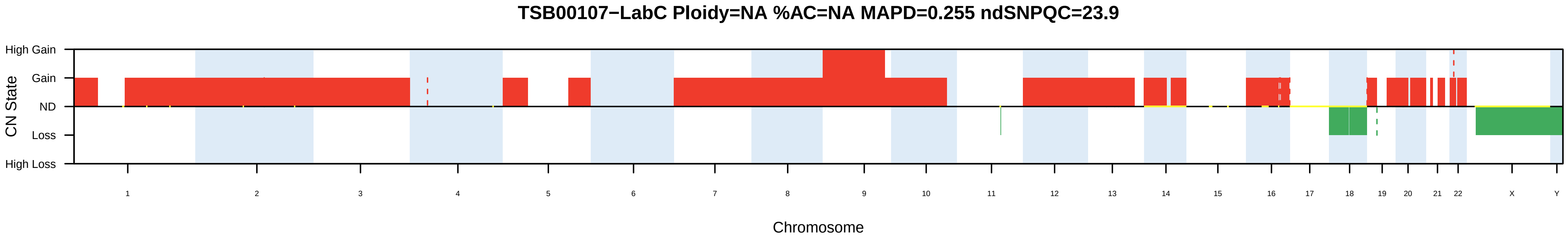
TSB00107−LabC Ploidy=NA %AC=NA MAPD=0.255 ndSNPQC=23.9
High Gain
Gain
ND
Loss
High Loss
CN State
1 2 3 4 5 6 7 8 9 10 11 12 13 14 15 16 17 18 19 20 21 22 X Y
Chromosome

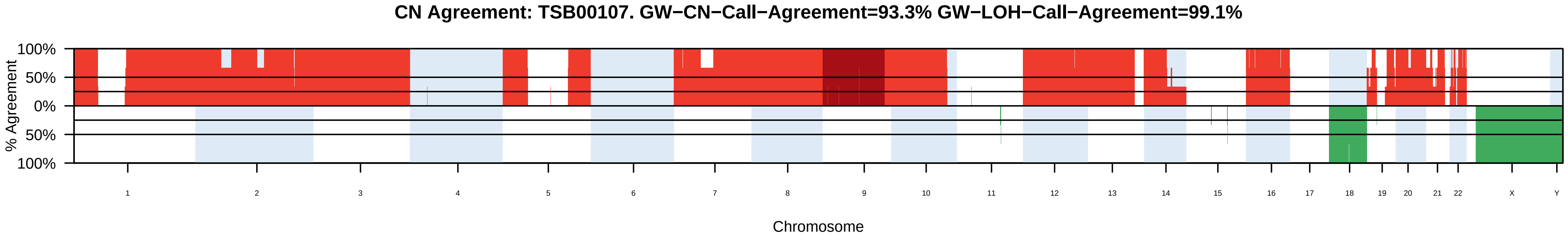
CN Agreement: TSB00107. GW−CN−Call−Agreement=93.3% GW−LOH−Call−Agreement=99.1%
100%
50%
0%
50%
100%
% Agreement
1 2 3 4 5 6 7 8 9 10 11 12 13 14 15 16 17 18 19 20 21 22 X Y
Chromosome

TSB00108−LabA Ploidy=NA %AC=NA MAPD=0.32 ndSNPQC=19.2

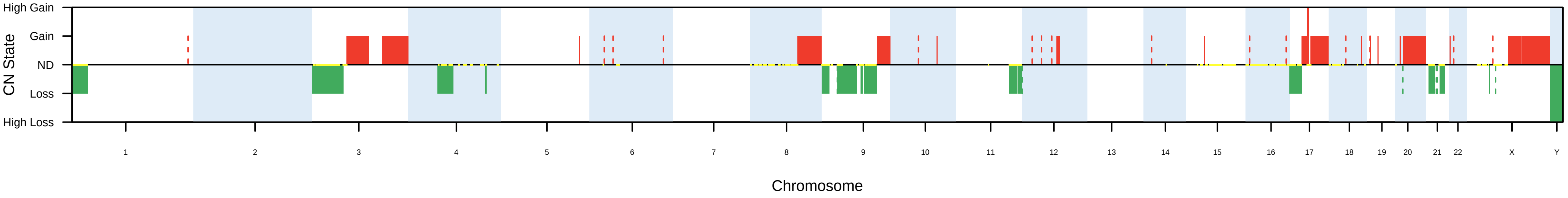

TSB00108−LabB Ploidy=NA %AC=NA MAPD=0.326 ndSNPQC=16

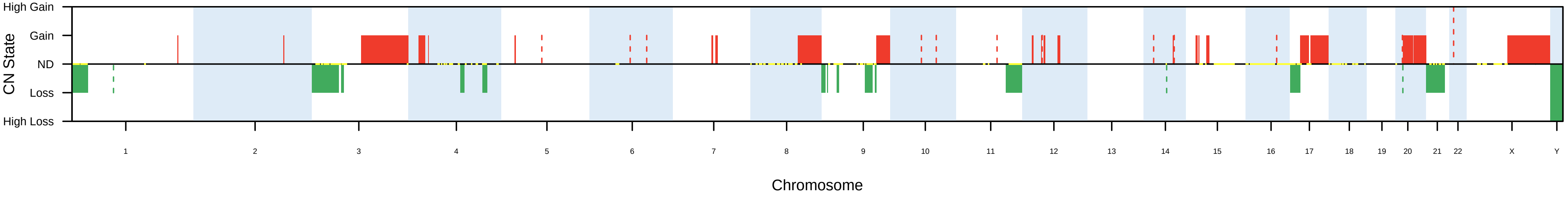

TSB00108−LabC Ploidy=NA %AC=NA MAPD=0.334 ndSNPQC=15.9

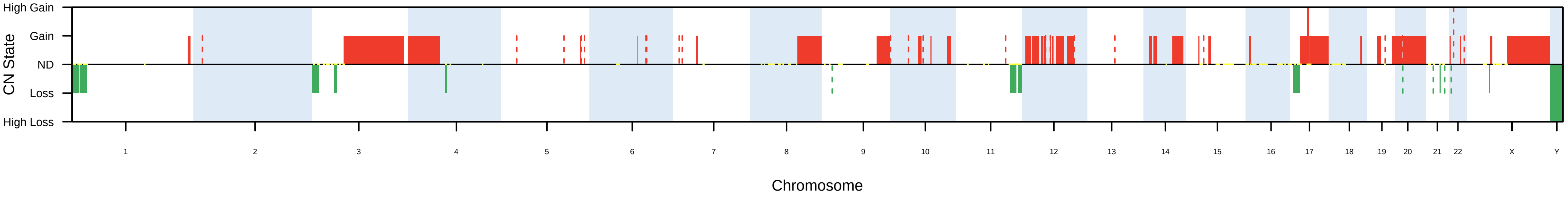

CN Agreement: TSB00108. GW−CN−Call−Agreement=80.2% GW−LOH−Call−Agreement=87.6%

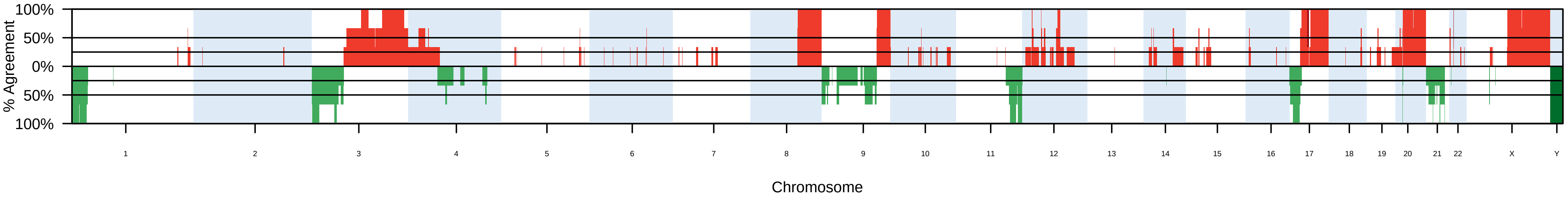

TSB00109−LabA Ploidy=NA %AC=NA MAPD=0.247 ndSNPQC=28.4

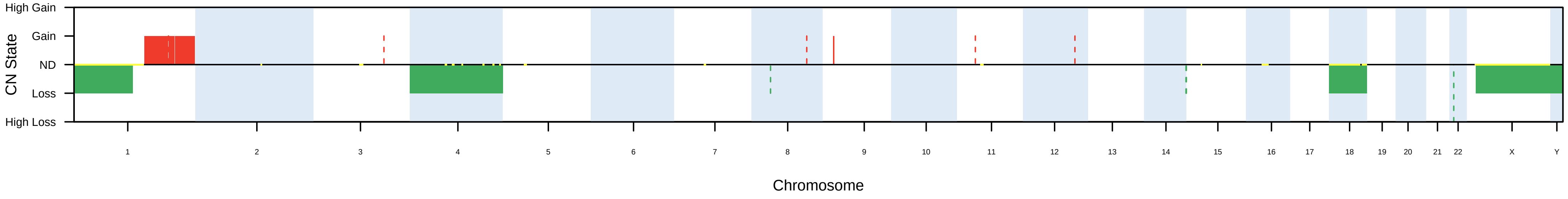

TSB00109−LabB Ploidy=NA %AC=NA MAPD=0.27 ndSNPQC=23.6

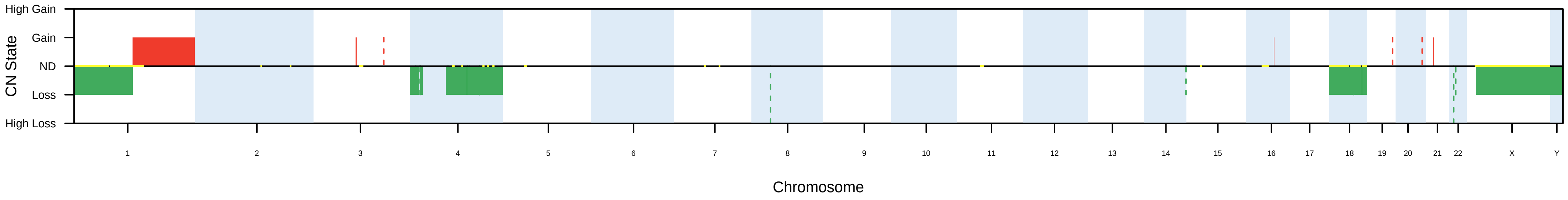

TSB00109−LabC Ploidy=NA %AC=NA MAPD=0.265 ndSNPQC=26.1

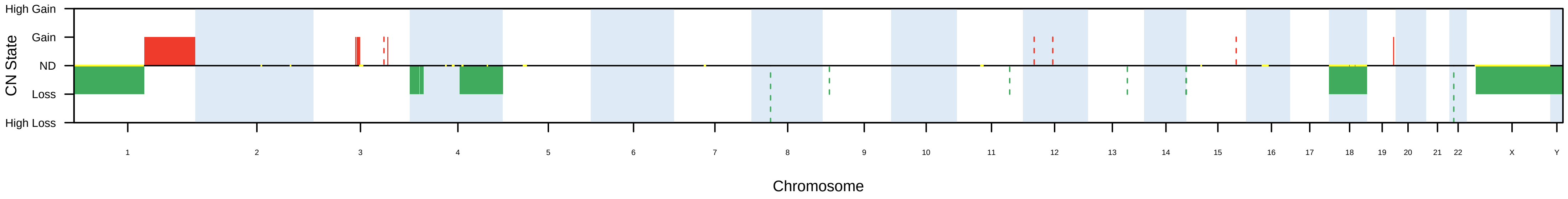

CN Agreement: TSB00109. GW−CN−Call−Agreement=96.5% GW−LOH−Call−Agreement=98.6%

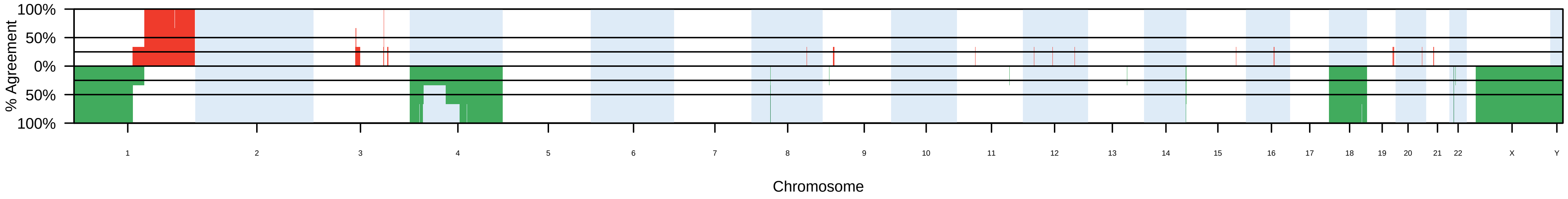

TSB00110−LabA Ploidy=2 %AC=45 MAPD=0.246 ndSNPQC=35.7

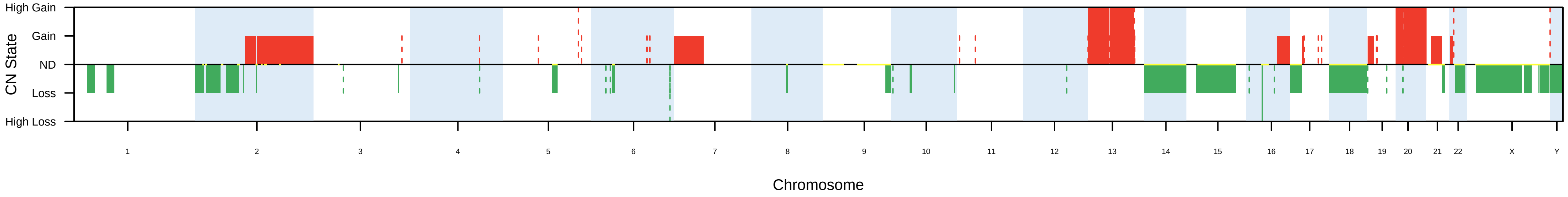

TSB00110−LabB Ploidy=2 %AC=50 MAPD=0.272 ndSNPQC=32.5

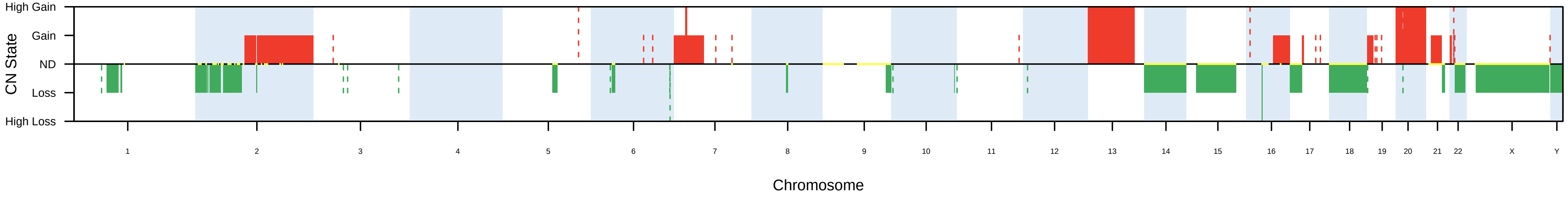

TSB00110−LabC Ploidy=2 %AC=50 MAPD=0.263 ndSNPQC=36.2

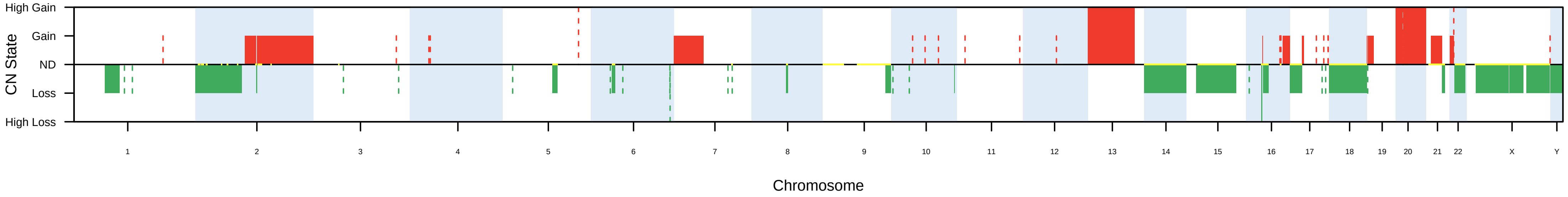

CN Agreement: TSB00110. GW−CN−Call−Agreement=94.5% GW−LOH−Call−Agreement=97.1%

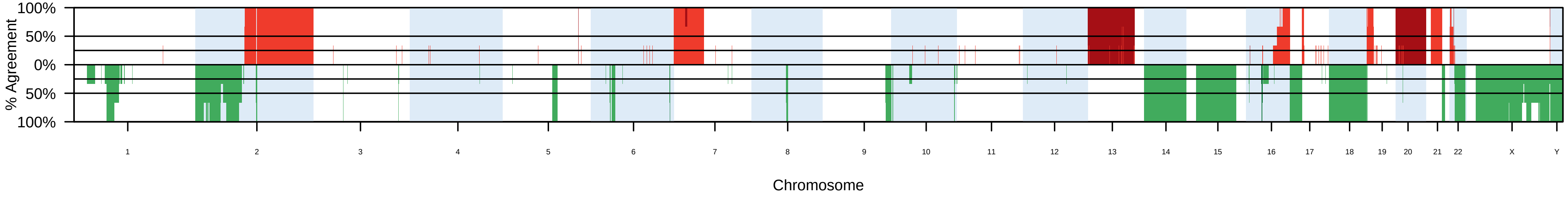

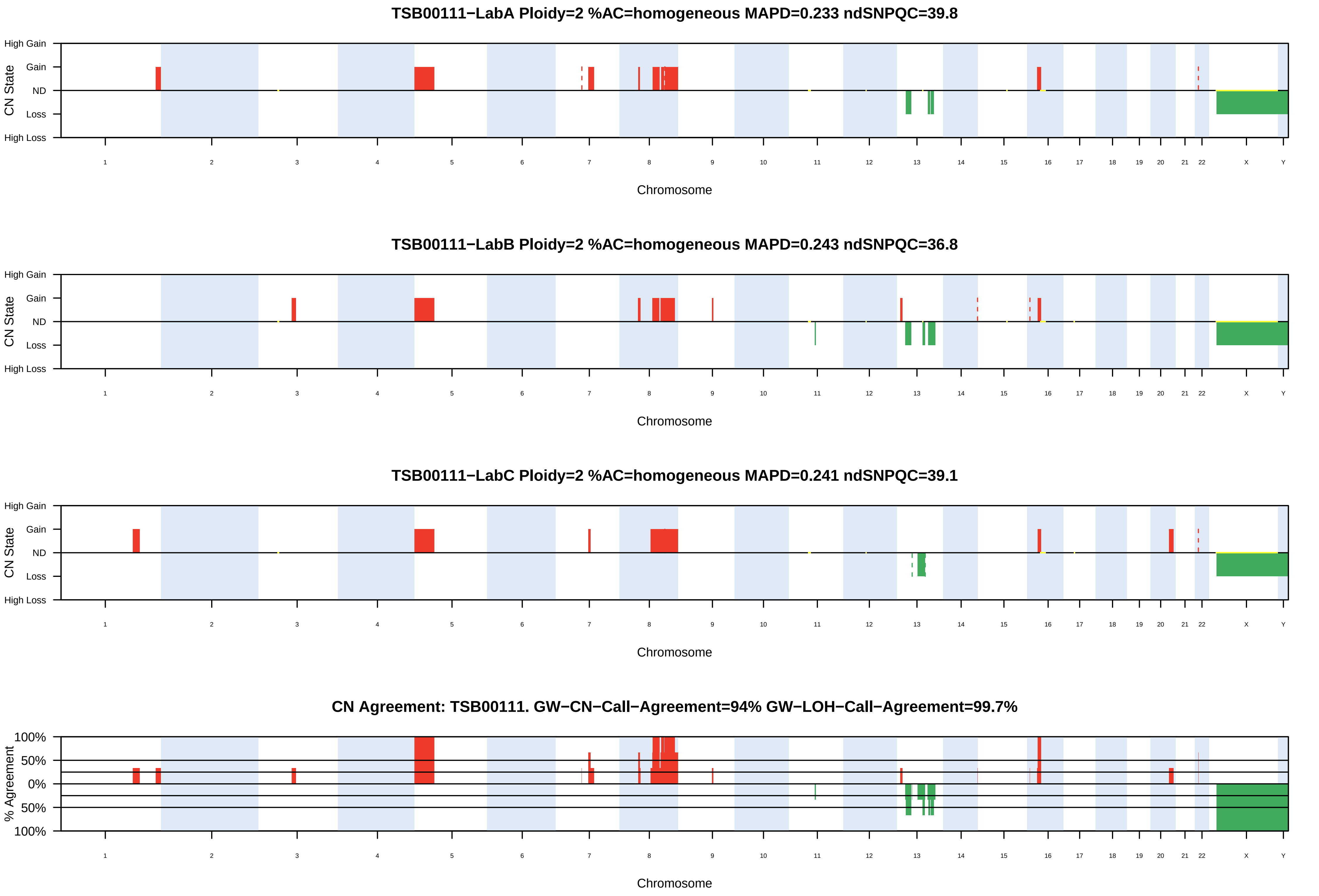
TSB00111−LabA Ploidy=2 %AC=homogeneous MAPD=0.233 ndSNPQC=39.8
TSB00111−LabB Ploidy=2 %AC=homogeneous MAPD=0.243 ndSNPQC=36.8
TSB00111−LabC Ploidy=2 %AC=homogeneous MAPD=0.241 ndSNPQC=39.1
CN State
High Gain
Gain
ND
Loss
High Loss
Chromosome
1 2 3 4 5 6 7 8 9 10 11 12 13 14 15 16 17 18 19 20 21 22 X Y
CN Agreement: TSB00111. GW−CN−Call−Agreement=94% GW−LOH−Call−Agreement=99.7%
% Agreement
100%
50%
0%
50%
100%
Chromosome

TSB00112−LabA Ploidy=NA %AC=NA MAPD=0.239 ndSNPQC=35.5

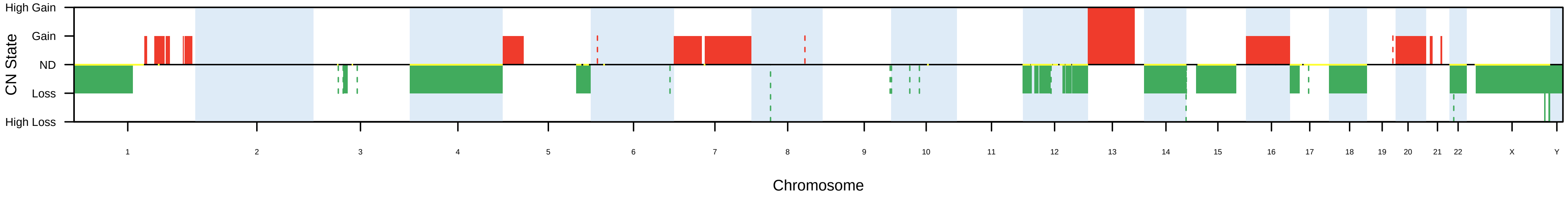

TSB00112−LabB Ploidy=NA %AC=NA MAPD=0.238 ndSNPQC=30.9

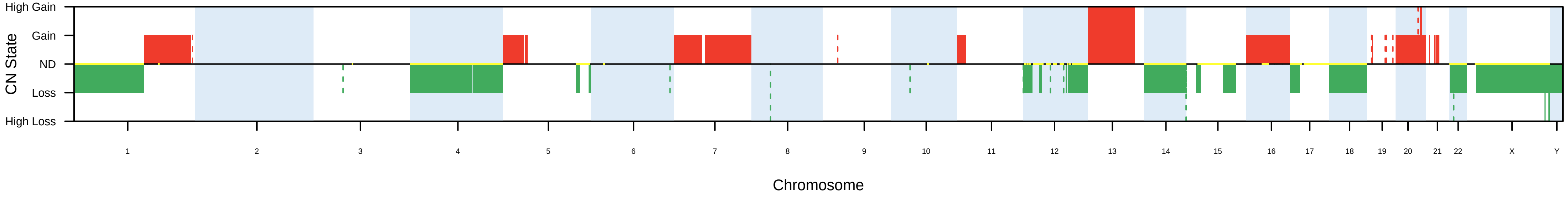

TSB00112−LabC Ploidy=NA %AC=NA MAPD=0.249 ndSNPQC=34.6

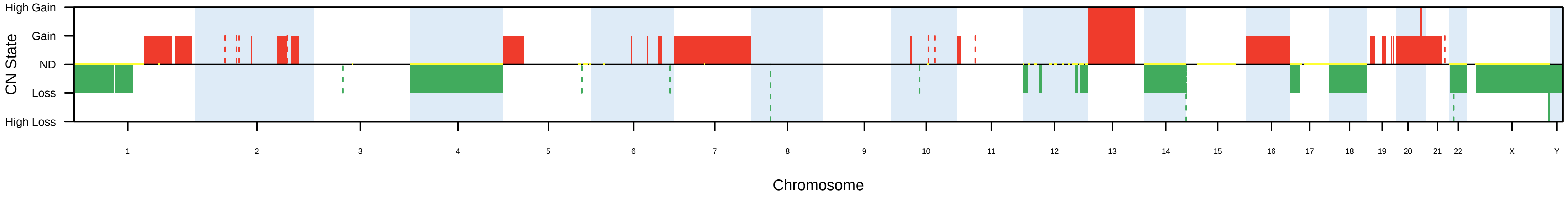

CN Agreement: TSB00112. GW−CN−Call−Agreement=86.4% GW−LOH−Call−Agreement=96.1%

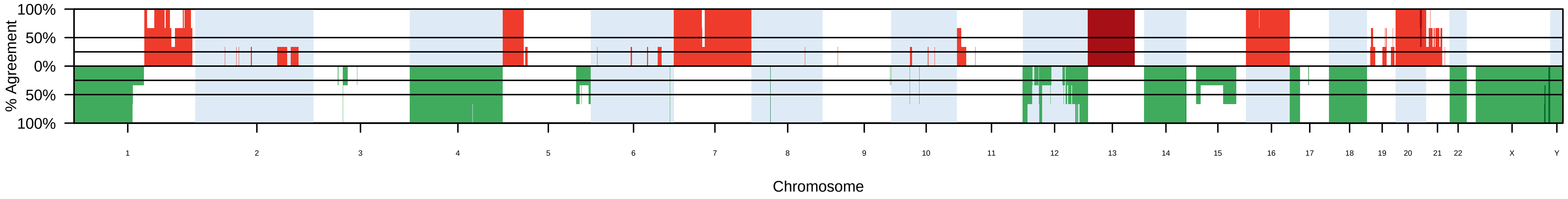

TSB00113−LabA Ploidy=2 %AC=40 MAPD=0.256 ndSNPQC=31.8

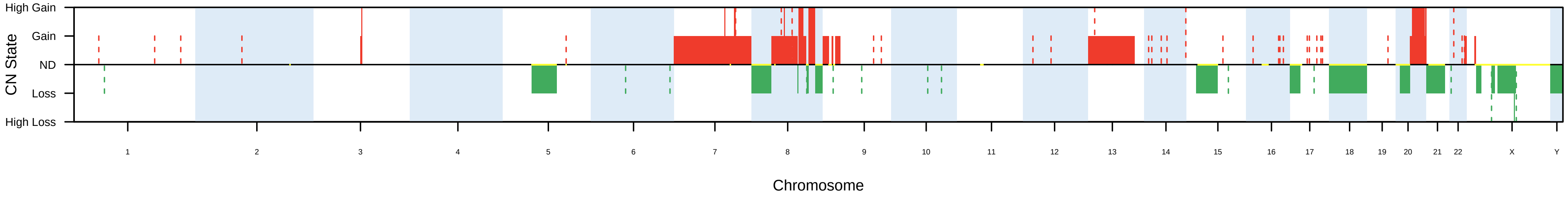

TSB00113−LabB Ploidy=2 %AC=40 MAPD=0.258 ndSNPQC=28.4

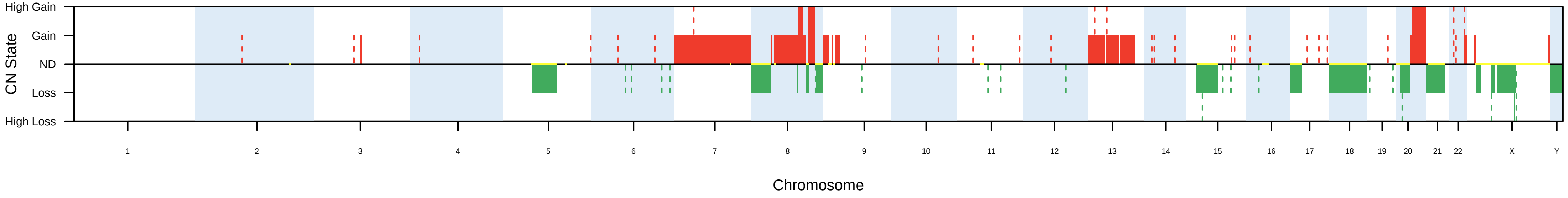

TSB00113−LabC Ploidy=2 %AC=40 MAPD=0.27 ndSNPQC=29.6

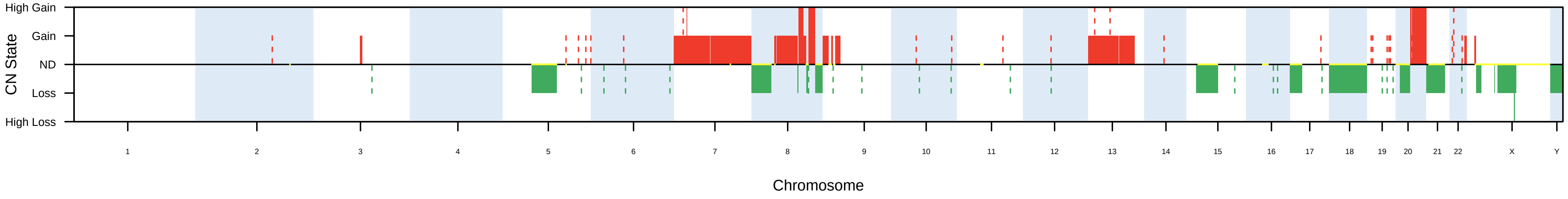

CN Agreement: TSB00113. GW−CN−Call−Agreement=97.3% GW−LOH−Call−Agreement=99.9%

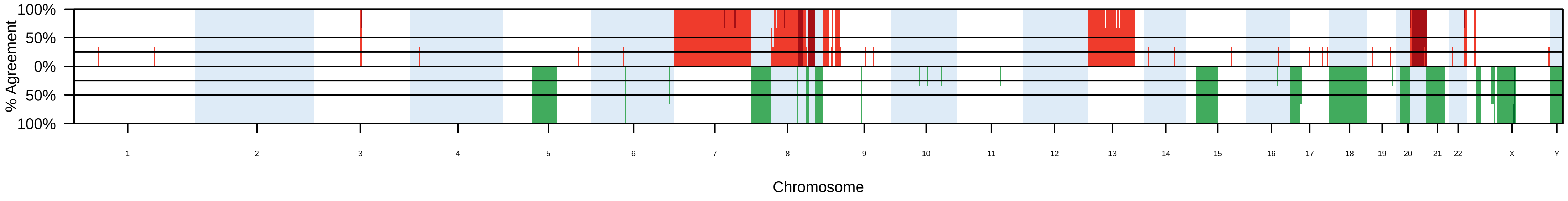

TSB00114−LabA Ploidy=NA %AC=NA MAPD=0.249 ndSNPQC=34.4

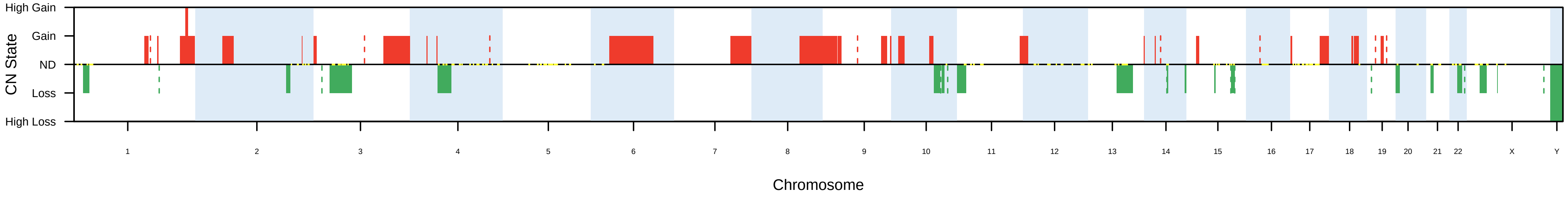

TSB00114−LabB Ploidy=2 %AC=homogeneous MAPD=0.247 ndSNPQC=31.7

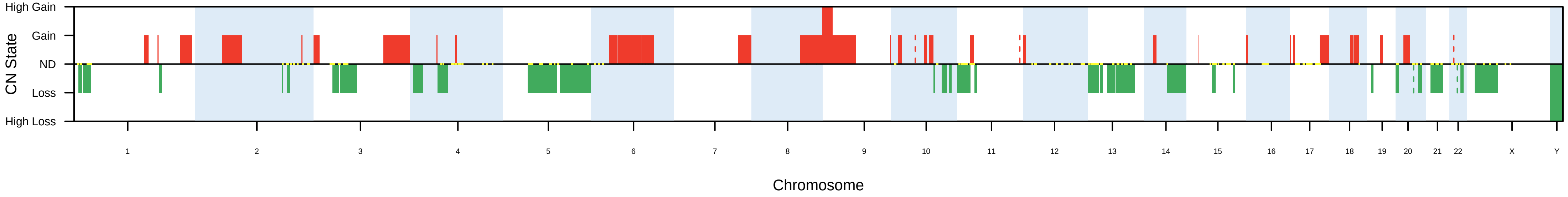

TSB00114−LabC Ploidy=NA %AC=NA MAPD=0.265 ndSNPQC=30.1

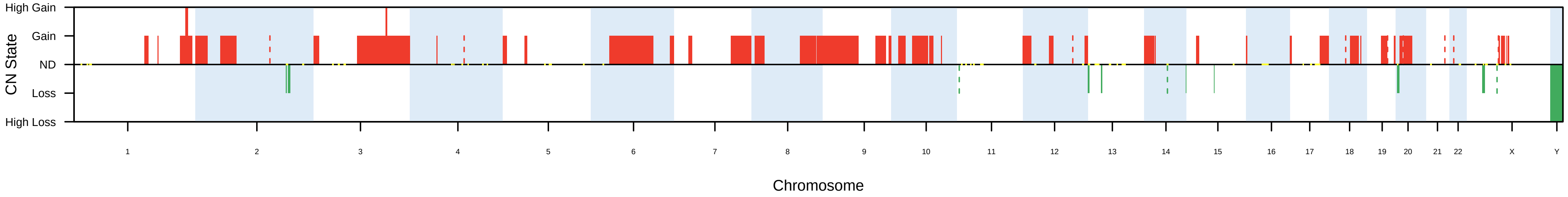

CN Agreement: TSB00114. GW−CN−Call−Agreement=65.6% GW−LOH−Call−Agreement=83.8%

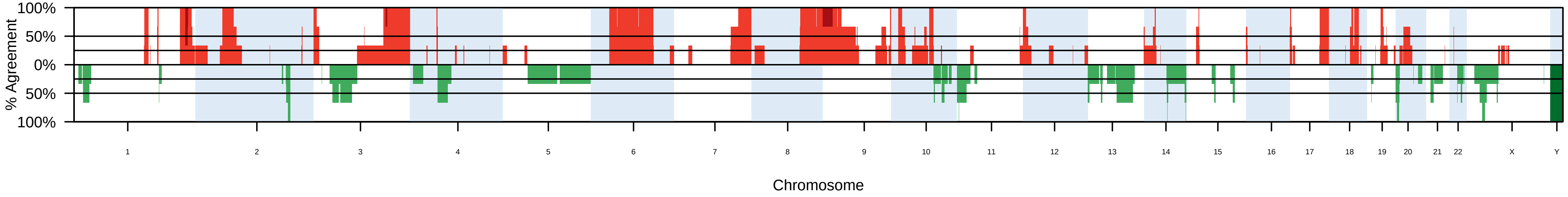

TSB00115−LabA Ploidy=2 %AC=40 MAPD=0.249 ndSNPQC=35

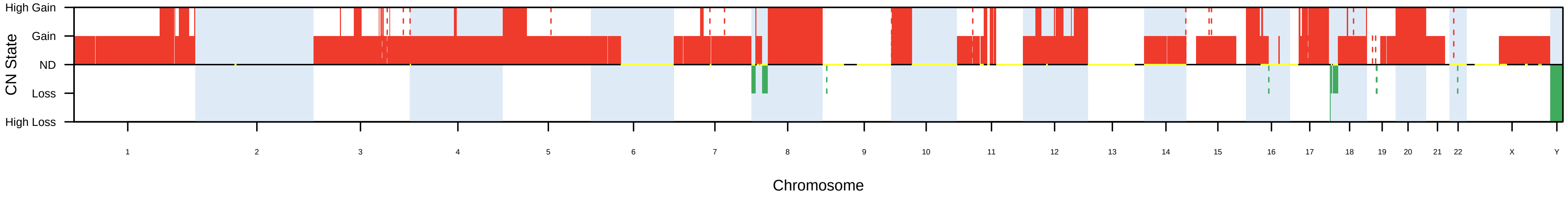

TSB00115−LabB Ploidy=2 %AC=45 MAPD=0.253 ndSNPQC=32.6

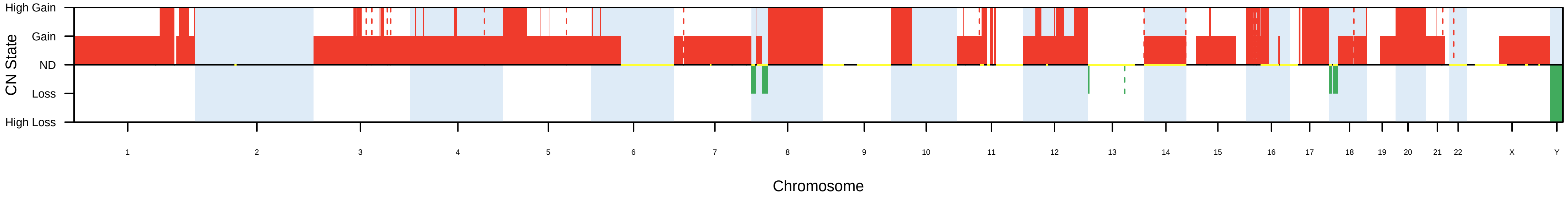

TSB00115−LabC Ploidy=NA %AC=NA MAPD=0.259 ndSNPQC=34.5

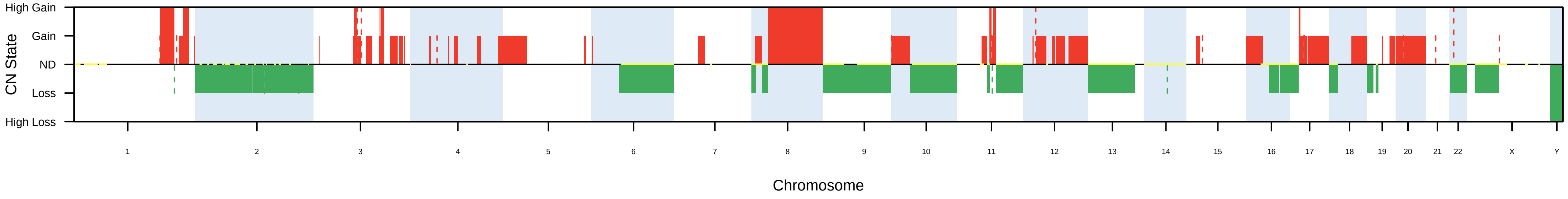

CN Agreement: TSB00115. GW−CN−Call−Agreement=14.3% GW−LOH−Call−Agreement=95%

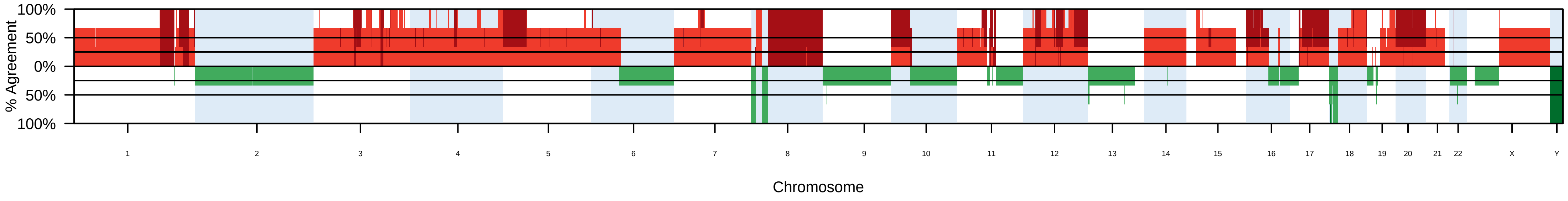

TSB00116−LabA Ploidy=2 %AC=30 MAPD=0.242 ndSNPQC=39.1

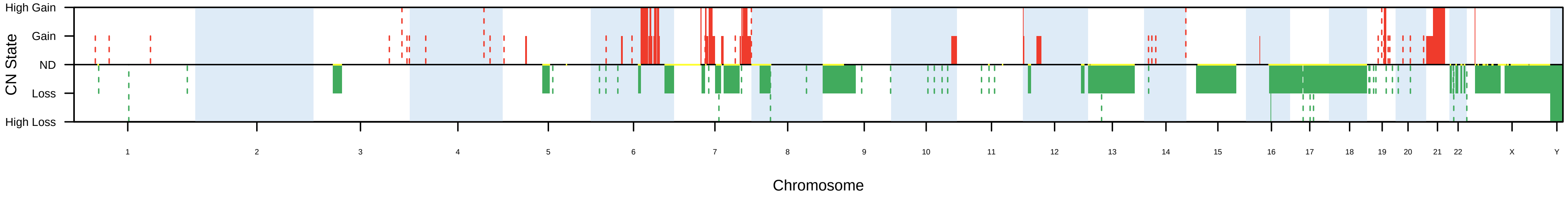

TSB00116−LabB Ploidy=2 %AC=35 MAPD=0.235 ndSNPQC=40

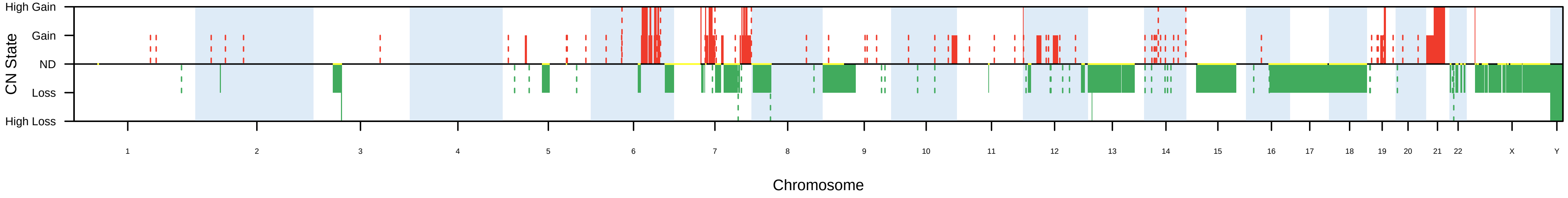

TSB00116−LabC Ploidy=2 %AC=30 MAPD=0.233 ndSNPQC=42.1

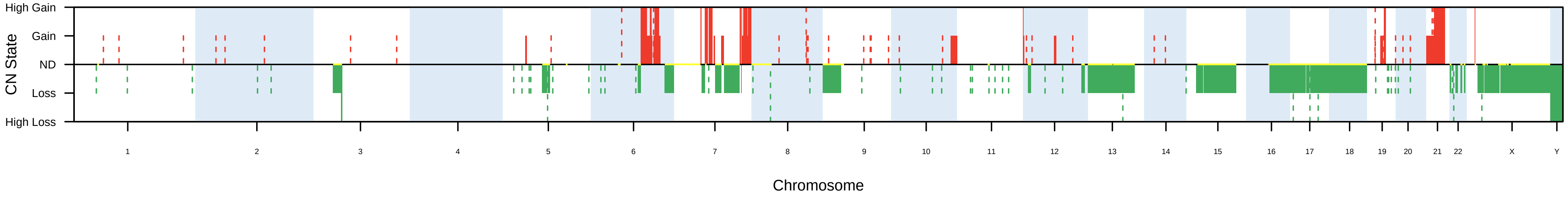

CN Agreement: TSB00116. GW−CN−Call−Agreement=93% GW−LOH−Call−Agreement=98.5%

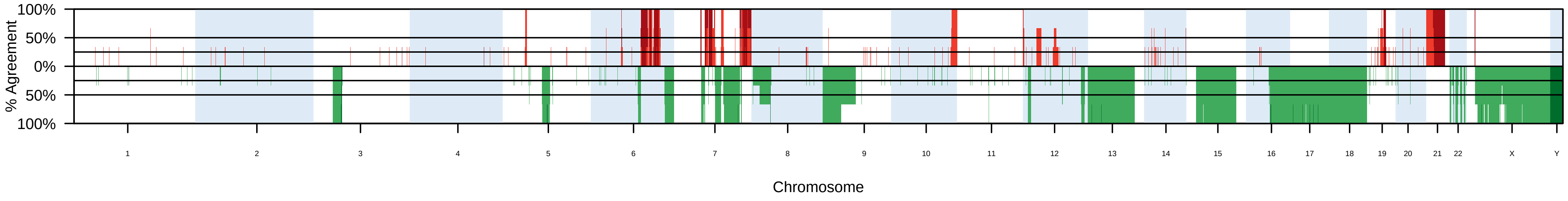

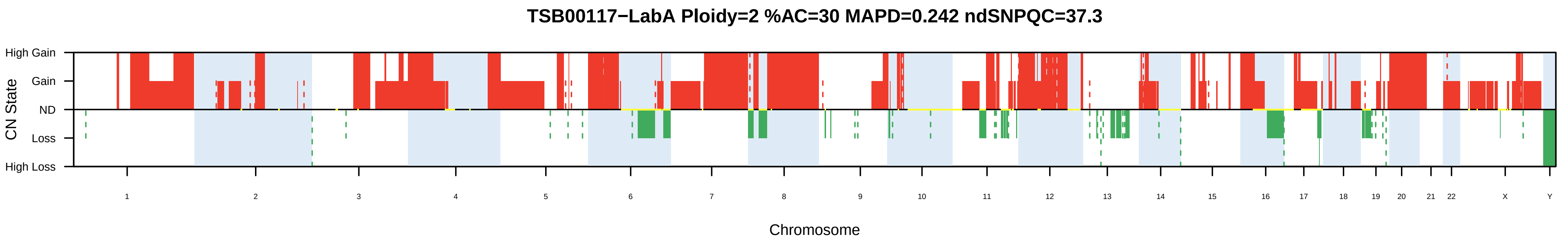
TSB00117−LabA Ploidy=2 %AC=30 MAPD=0.242 ndSNPQC=37.3
CN State
High Gain
Gain
ND
Loss
High Loss
1 2 3 4 5 6 7 8 9 10 11 12 13 14 15 16 17 18 19 20 21 22 X Y
Chromosome

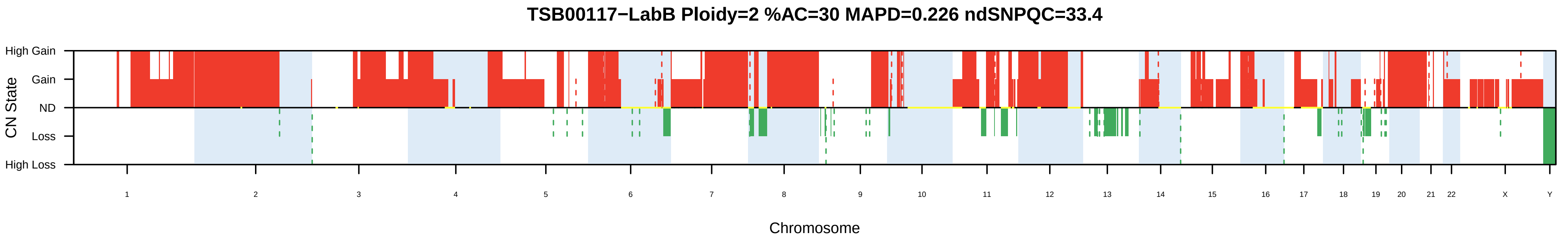
TSB00117−LabB Ploidy=2 %AC=30 MAPD=0.226 ndSNPQC=33.4
CN State
High Gain
Gain
ND
Loss
High Loss
1 2 3 4 5 6 7 8 9 10 11 12 13 14 15 16 17 18 19 20 21 22 X Y
Chromosome

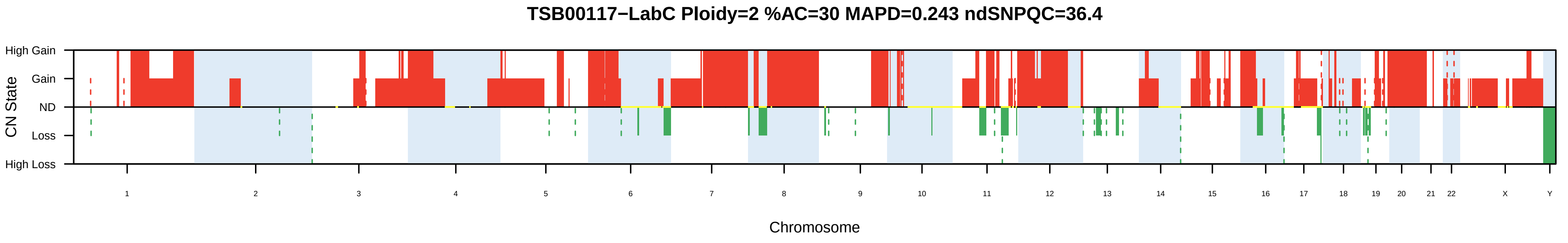
TSB00117−LabC Ploidy=2 %AC=30 MAPD=0.243 ndSNPQC=36.4
CN State
High Gain
Gain
ND
Loss
High Loss
1 2 3 4 5 6 7 8 9 10 11 12 13 14 15 16 17 18 19 20 21 22 X Y
Chromosome

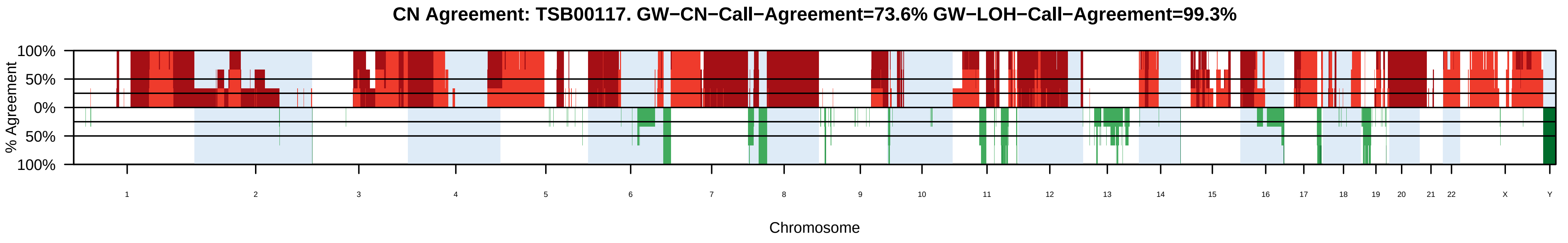
CN Agreement: TSB00117. GW−CN−Call−Agreement=73.6% GW−LOH−Call−Agreement=99.3%
% Agreement
100%
50%
0%
50%
100%
1 2 3 4 5 6 7 8 9 10 11 12 13 14 15 16 17 18 19 20 21 22 X Y
Chromosome

TSB00118−LabA Ploidy=2 %AC=55 MAPD=0.241 ndSNPQC=40.5

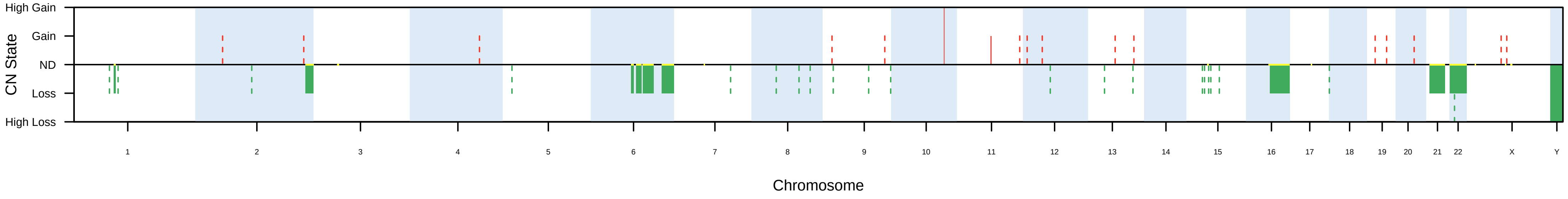

TSB00118−LabB Ploidy=2 %AC=55 MAPD=0.241 ndSNPQC=34.5

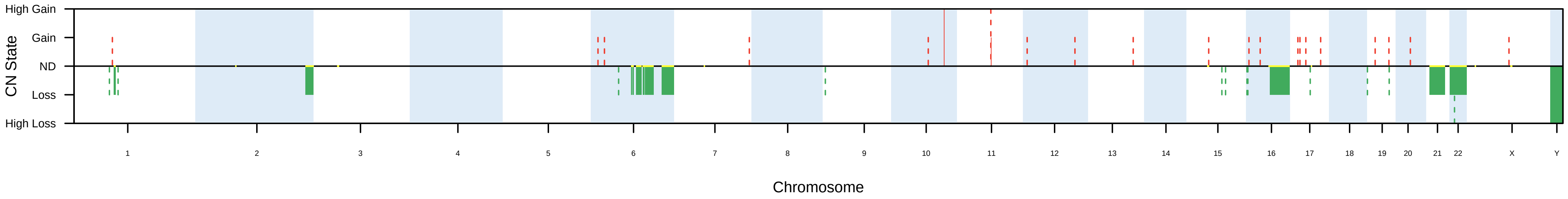

TSB00118−LabC Ploidy=2 %AC=55 MAPD=0.248 ndSNPQC=39.9

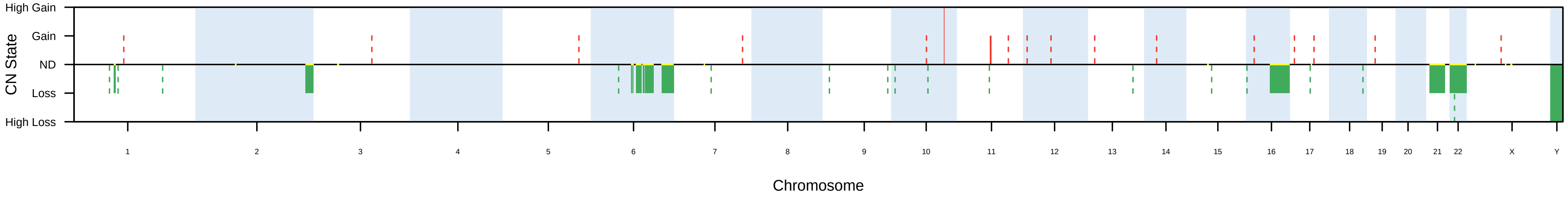

CN Agreement: TSB00118. GW−CN−Call−Agreement=99.1% GW−LOH−Call−Agreement=99.6%

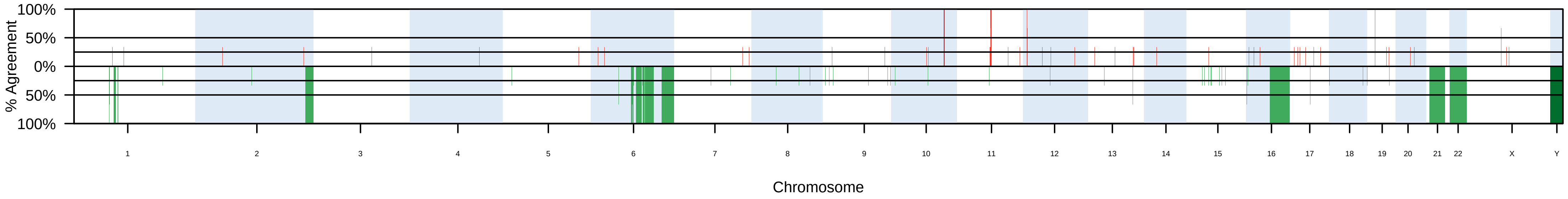

TSB00119−LabA Ploidy=2 %AC=55 MAPD=0.222 ndSNPQC=47.5

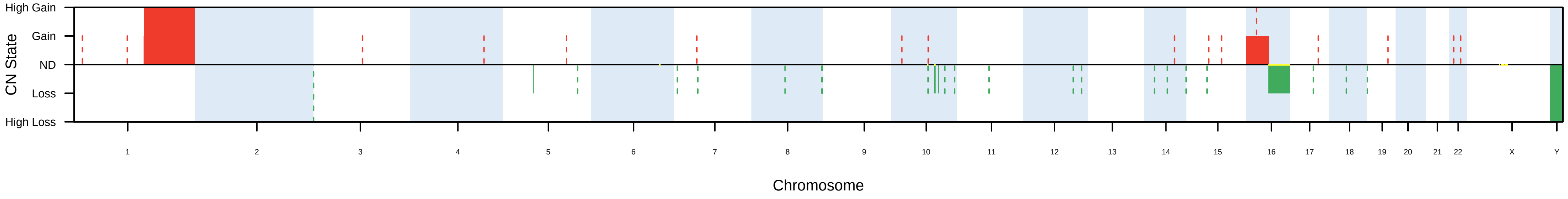

TSB00119−LabB Ploidy=2 %AC=55 MAPD=0.226 ndSNPQC=44

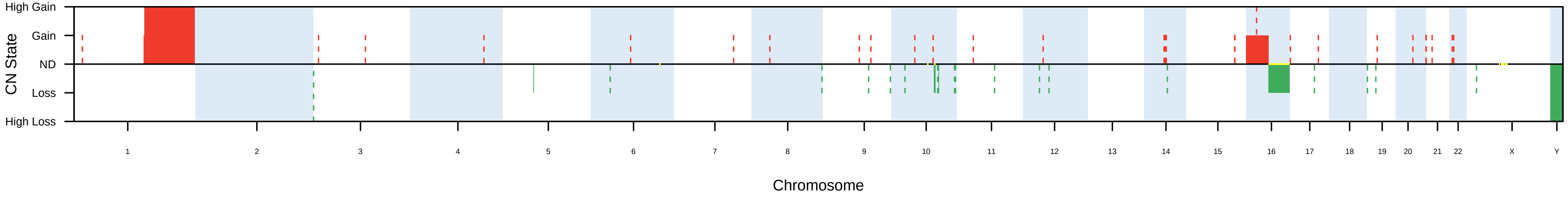

TSB00119−LabC Ploidy=2 %AC=50 MAPD=0.221 ndSNPQC=50.7

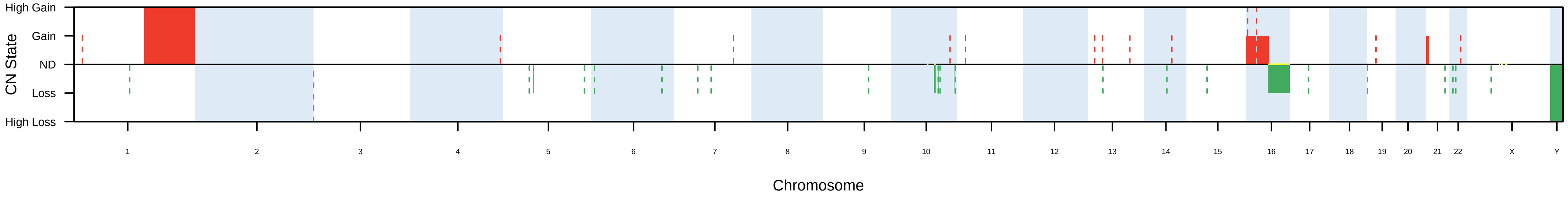

CN Agreement: TSB00119. GW−CN−Call−Agreement=99% GW−LOH−Call−Agreement=99.8%

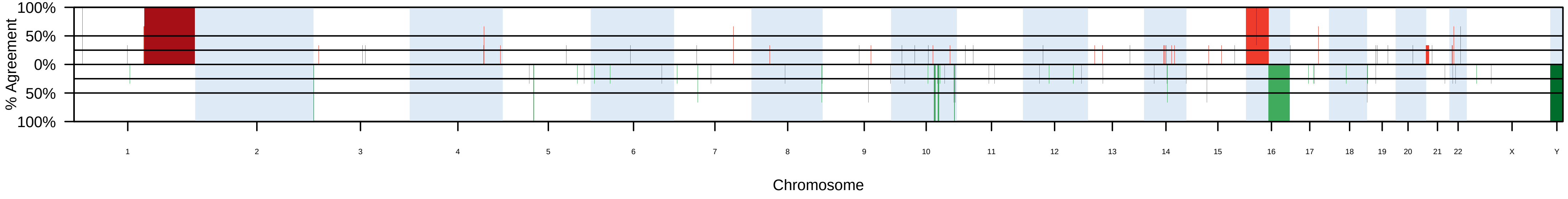

TSB00142−LabA Ploidy=2 %AC=homogeneous MAPD=0.263 ndSNPQC=33

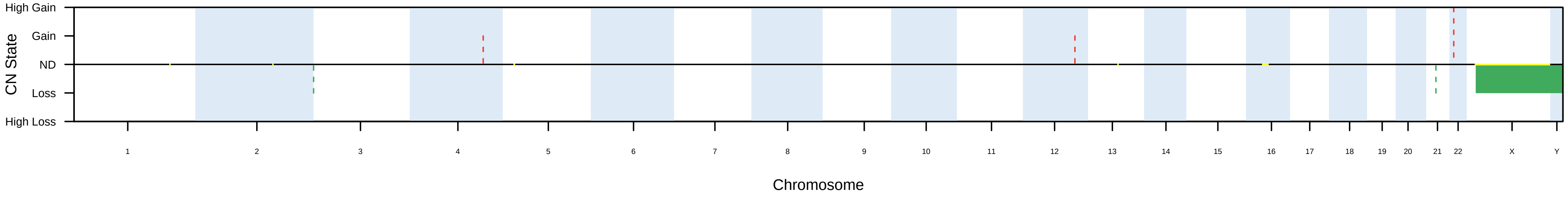

TSB00142−LabB Ploidy=2 %AC=homogeneous MAPD=0.258 ndSNPQC=32.2

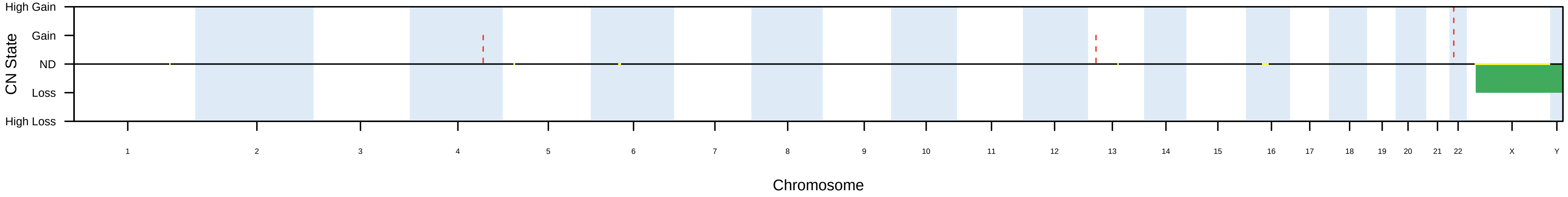

TSB00142−LabC Ploidy=2 %AC=homogeneous MAPD=0.244 ndSNPQC=32.2

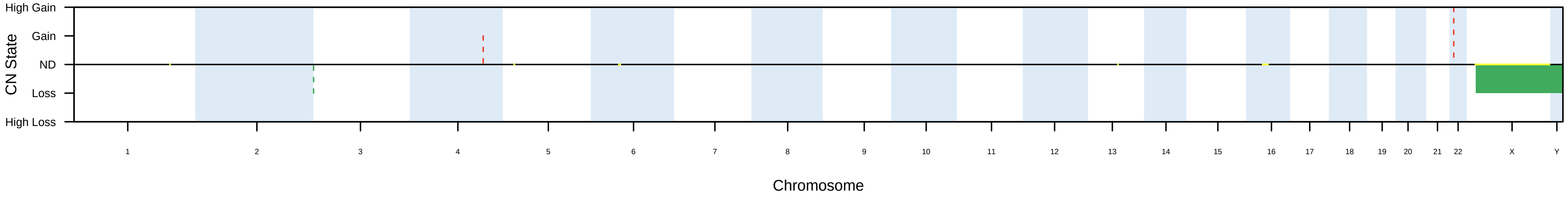

CN Agreement: TSB00142. GW−CN−Call−Agreement=100% GW−LOH−Call−Agreement=99.7%

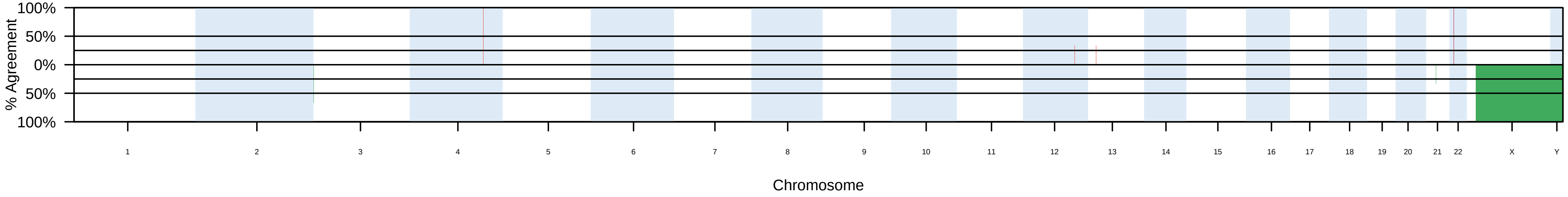

TSB00143−LabA Ploidy=2 %AC=homogeneous MAPD=0.27 ndSNPQC=36.6

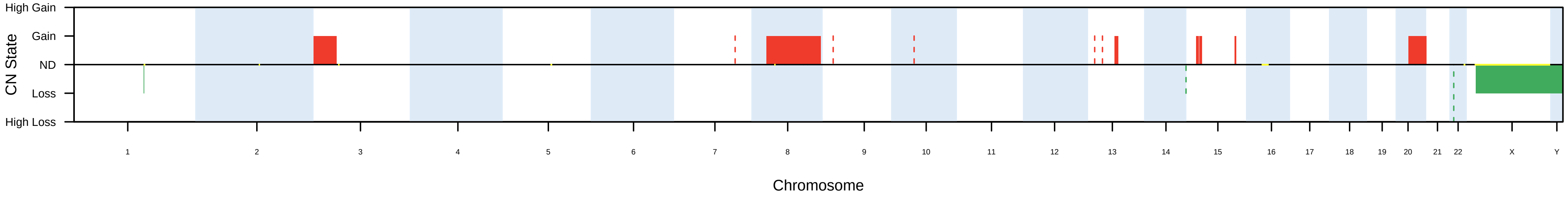

TSB00143−LabB Ploidy=2 %AC=homogeneous MAPD=0.263 ndSNPQC=34.8

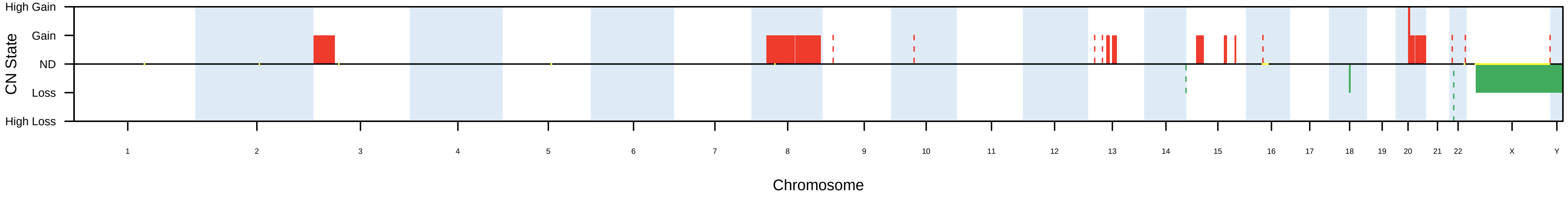

TSB00143−LabC Ploidy=2 %AC=homogeneous MAPD=0.262 ndSNPQC=37.1

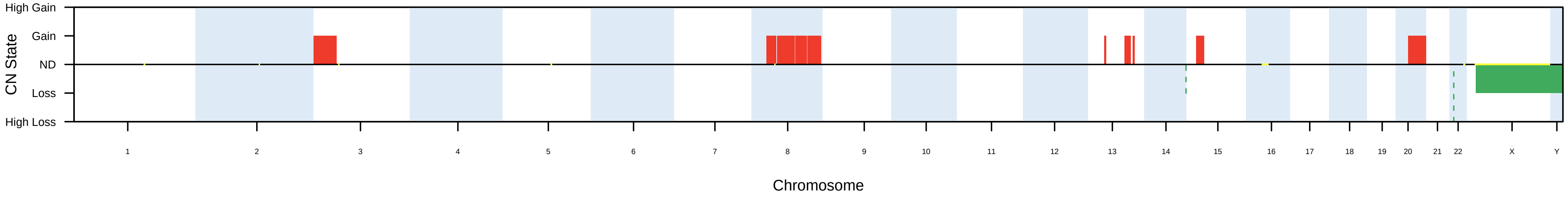

CN Agreement: TSB00143. GW−CN−Call−Agreement=97.3% GW−LOH−Call−Agreement=100%

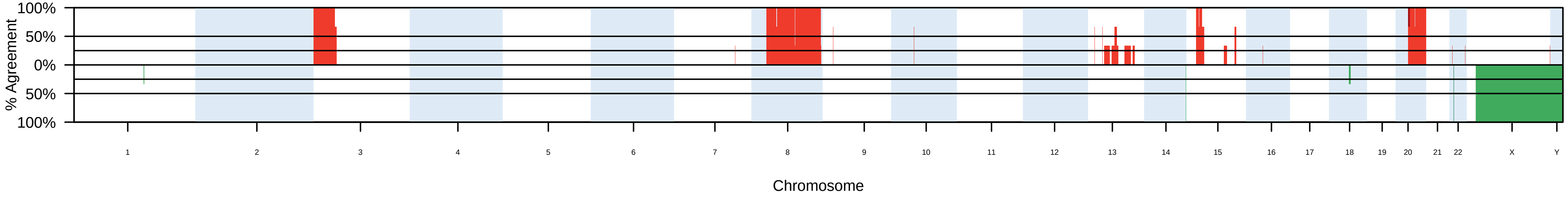

TSB00144−LabA Ploidy=2 %AC=homogeneous MAPD=0.263 ndSNPQC=26

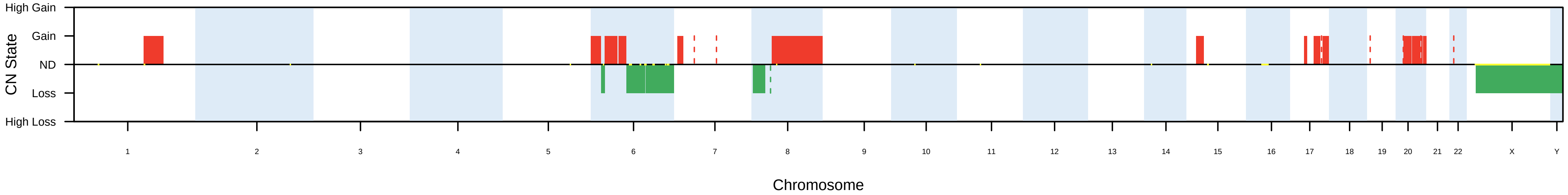

TSB00144−LabB Ploidy=2 %AC=homogeneous MAPD=0.264 ndSNPQC=24.4

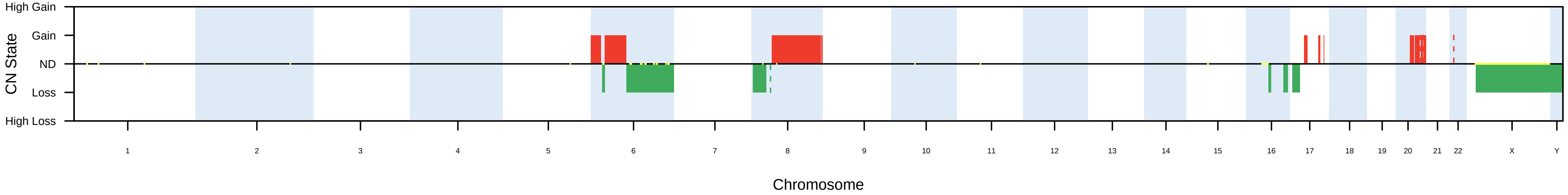

TSB00144−LabC Ploidy=2 %AC=homogeneous MAPD=0.264 ndSNPQC=23.3

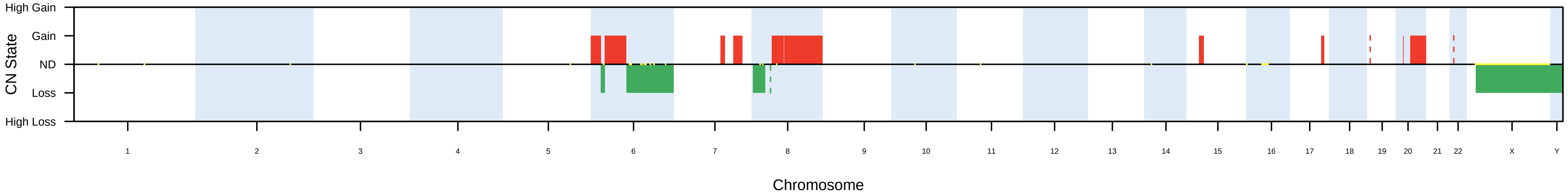

CN Agreement: TSB00144. GW−CN−Call−Agreement=93.2% GW−LOH−Call−Agreement=98.5%

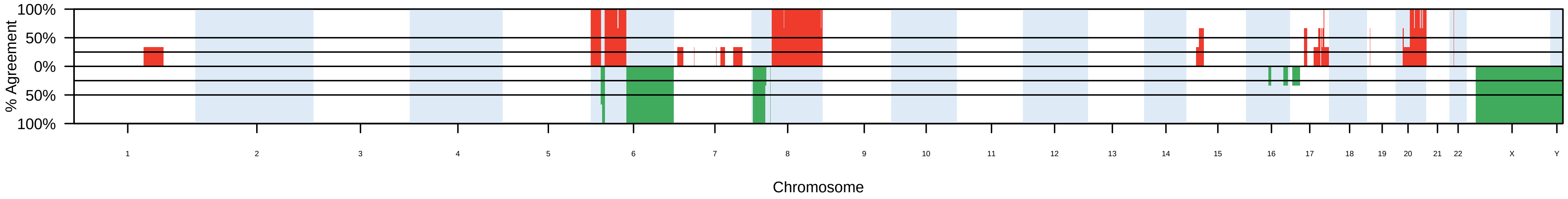

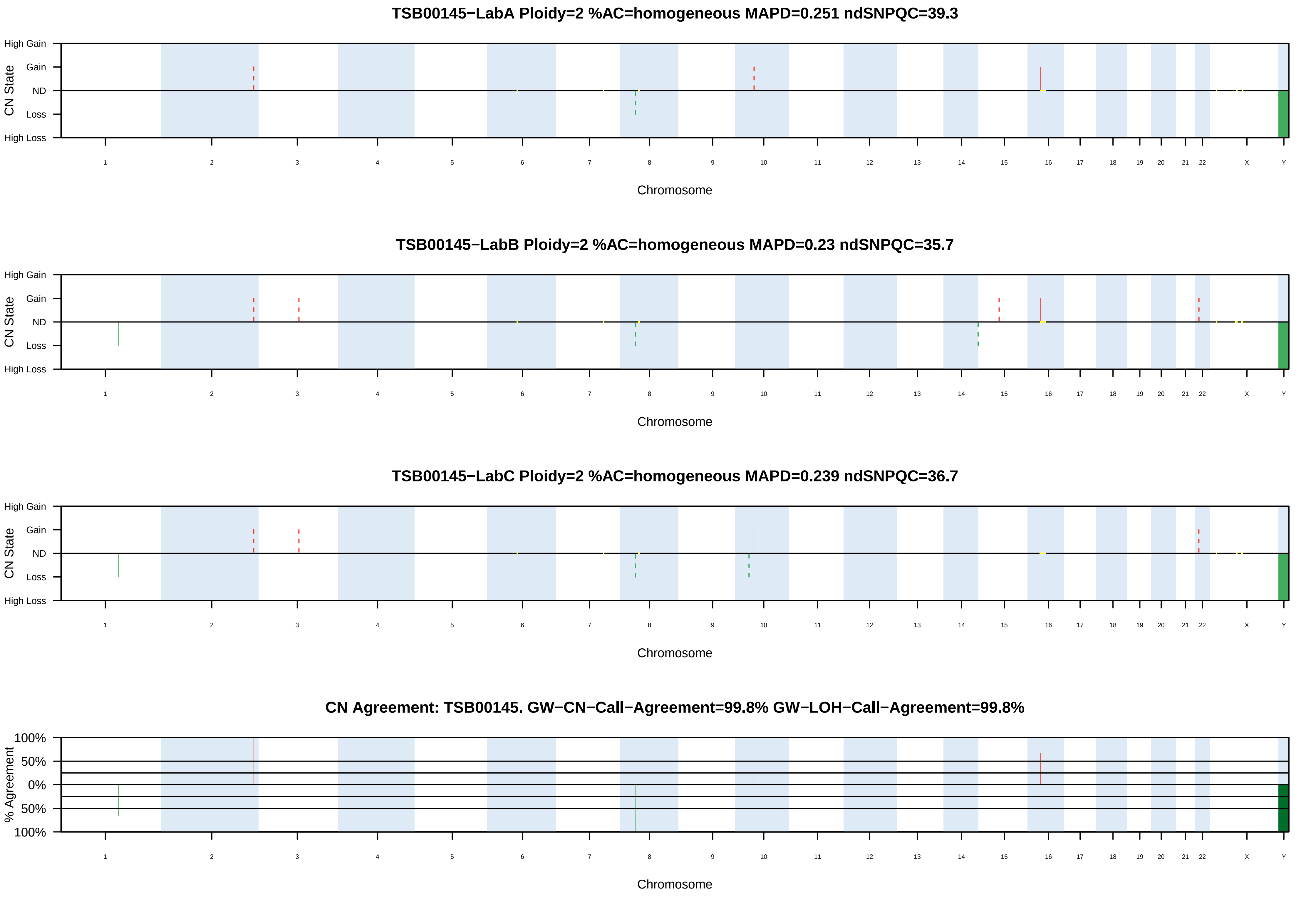
TSB00145−LabA Ploidy=2 %AC=homogeneous MAPD=0.251 ndSNPQC=39.3
High Gain
Gain
ND
Loss
High Loss
CN State
1 2 3 4 5 6 7 8 9 10 11 12 13 14 15 16 17 18 19 20 21 22 X Y
Chromosome
TSB00145−LabB Ploidy=2 %AC=homogeneous MAPD=0.23 ndSNPQC=35.7
High Gain
Gain
ND
Loss
High Loss
CN State
1 2 3 4 5 6 7 8 9 10 11 12 13 14 15 16 17 18 19 20 21 22 X Y
Chromosome
TSB00145−LabC Ploidy=2 %AC=homogeneous MAPD=0.239 ndSNPQC=36.7
High Gain
Gain
ND
Loss
High Loss
CN State
1 2 3 4 5 6 7 8 9 10 11 12 13 14 15 16 17 18 19 20 21 22 X Y
Chromosome
CN Agreement: TSB00145. GW−CN−Call−Agreement=99.8% GW−LOH−Call−Agreement=99.8%
100%
50%
0%
50%
100%
% Agreement
1 2 3 4 5 6 7 8 9 10 11 12 13 14 15 16 17 18 19 20 21 22 X Y
Chromosome

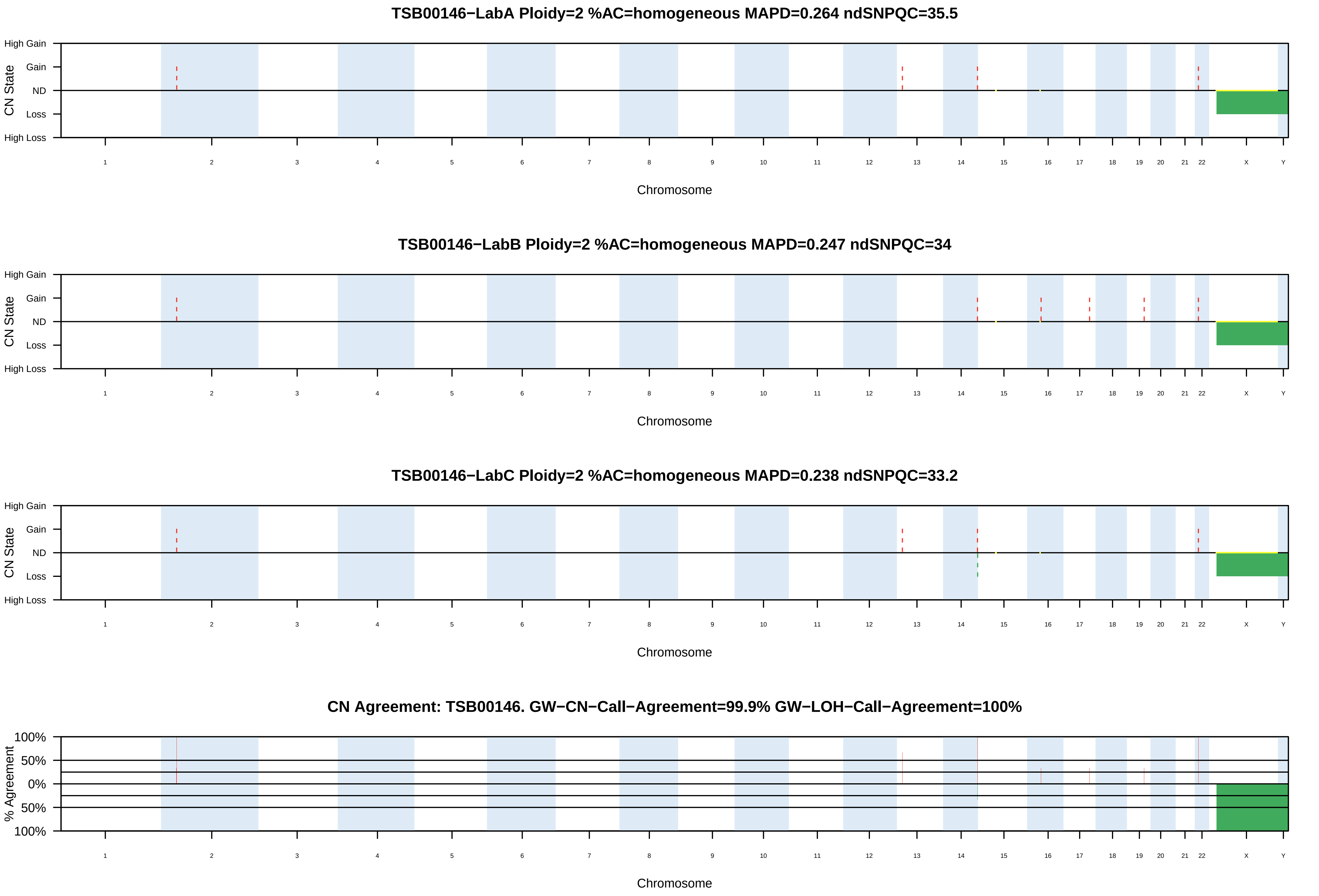

TSB00146−LabA Ploidy=2 %AC=homogeneous MAPD=0.264 ndSNPQC=35.5
TSB00146−LabB Ploidy=2 %AC=homogeneous MAPD=0.247 ndSNPQC=34
TSB00146−LabC Ploidy=2 %AC=homogeneous MAPD=0.238 ndSNPQC=33.2
CN Agreement: TSB00146. GW−CN−Call−Agreement=99.9% GW−LOH−Call−Agreement=100%
CN State
High Gain
Gain
ND
Loss
High Loss
% Agreement
100%
50%
0%
50%
100%
Chromosome
1 2 3 4 5 6 7 8 9 10 11 12 13 14 15 16 17 18 19 20 21 22 X Y

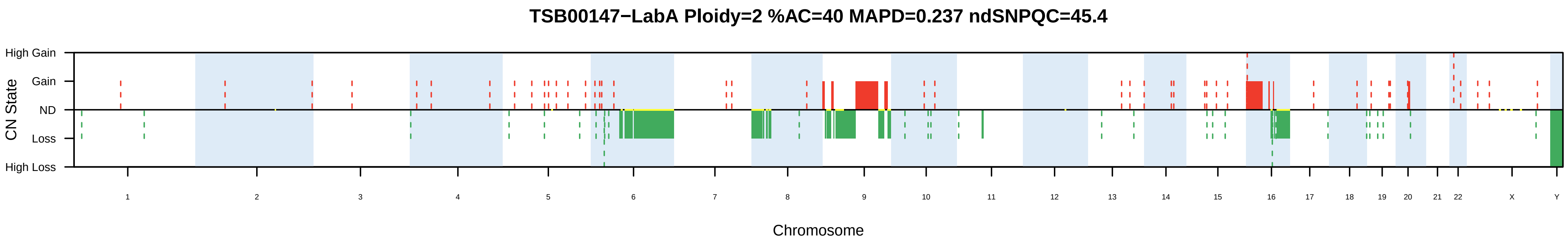
TSB00147−LabA Ploidy=2 %AC=40 MAPD=0.237 ndSNPQC=45.4
High Gain
Gain
ND
Loss
High Loss
CN State
1 2 3 4 5 6 7 8 9 10 11 12 13 14 15 16 17 18 19 20 21 22 X Y
Chromosome

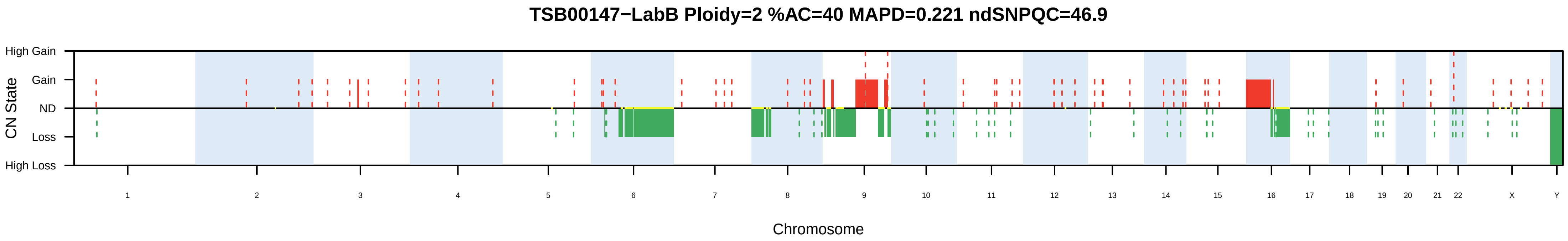
TSB00147−LabB Ploidy=2 %AC=40 MAPD=0.221 ndSNPQC=46.9
High Gain
Gain
ND
Loss
High Loss
CN State
1 2 3 4 5 6 7 8 9 10 11 12 13 14 15 16 17 18 19 20 21 22 X Y
Chromosome

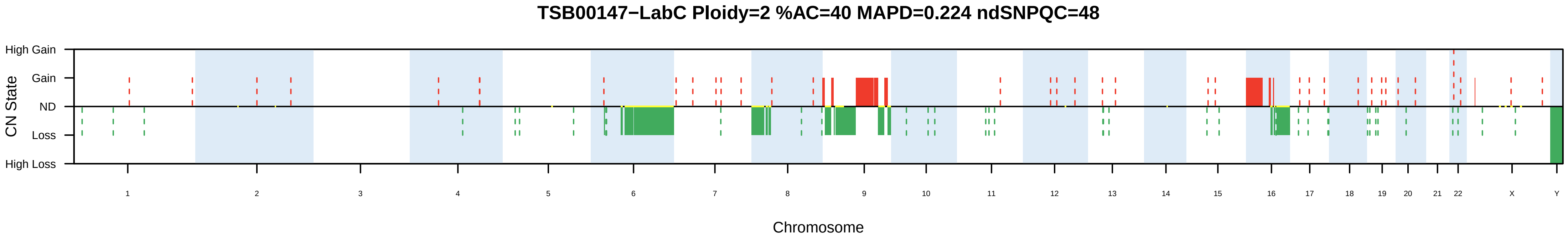
TSB00147−LabC Ploidy=2 %AC=40 MAPD=0.224 ndSNPQC=48
High Gain
Gain
ND
Loss
High Loss
CN State
1 2 3 4 5 6 7 8 9 10 11 12 13 14 15 16 17 18 19 20 21 22 X Y
Chromosome

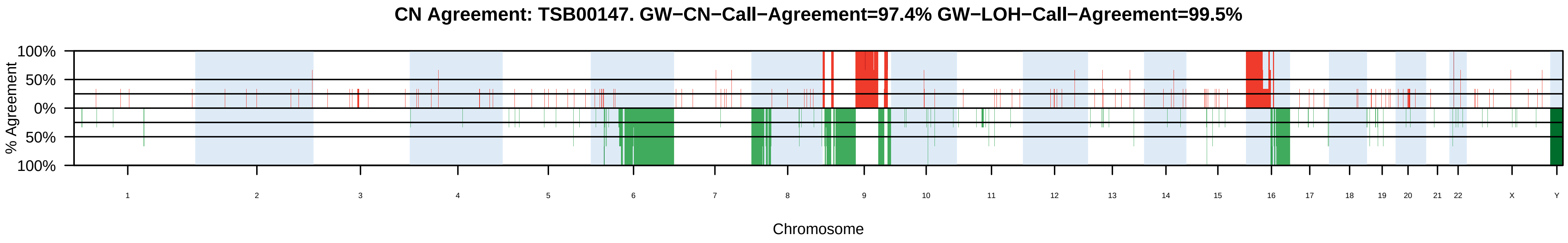
CN Agreement: TSB00147. GW−CN−Call−Agreement=97.4% GW−LOH−Call−Agreement=99.5%
100%
50%
0%
50%
100%
% Agreement
1 2 3 4 5 6 7 8 9 10 11 12 13 14 15 16 17 18 19 20 21 22 X Y
Chromosome

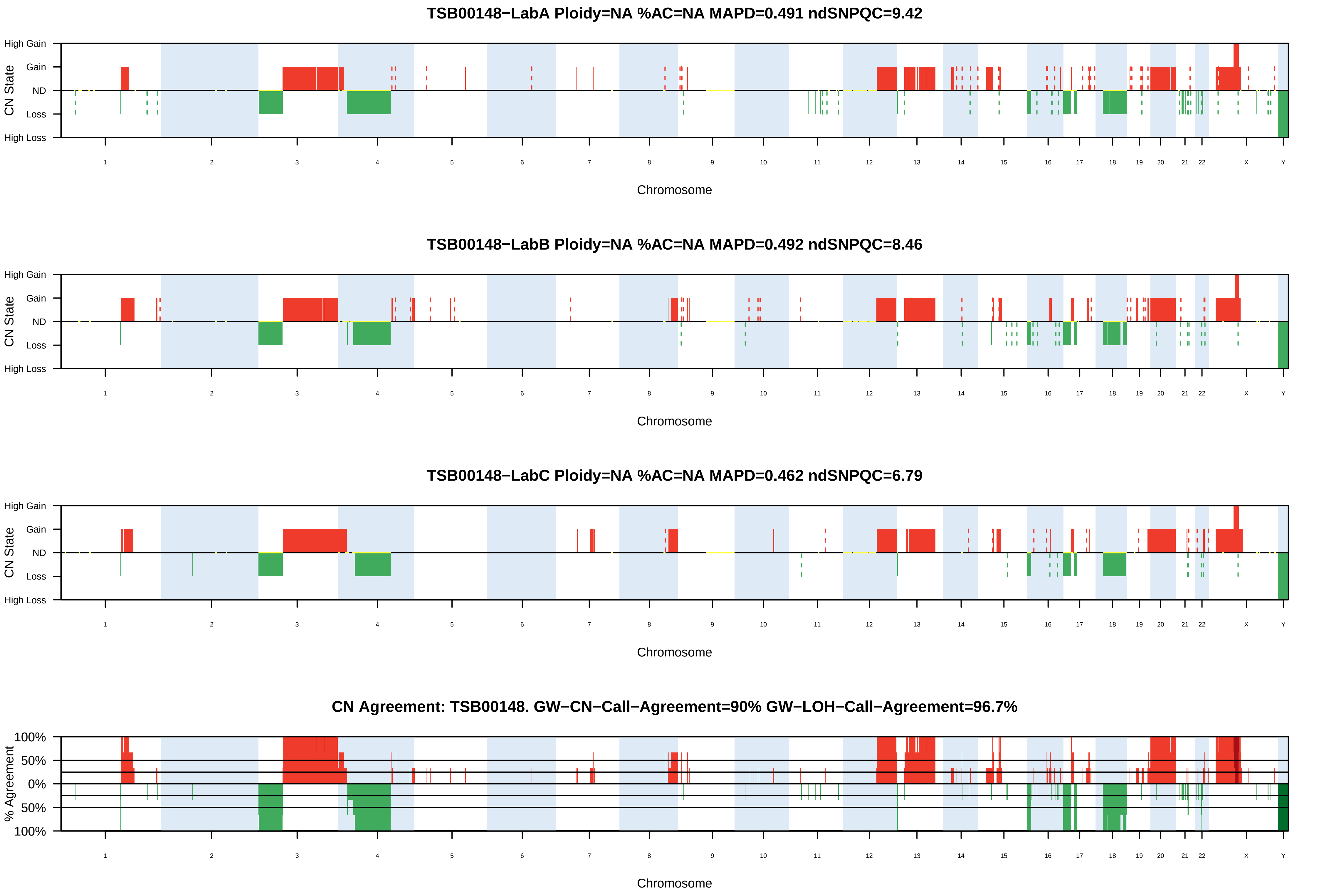
TSB00148−LabA Ploidy=NA %AC=NA MAPD=0.491 ndSNPQC=9.42
High Gain
Gain
ND
Loss
High Loss
CN State
1 2 3 4 5 6 7 8 9 10 11 12 13 14 15 16 17 18 19 20 21 22 X Y
Chromosome
TSB00148−LabB Ploidy=NA %AC=NA MAPD=0.492 ndSNPQC=8.46
High Gain
Gain
ND
Loss
High Loss
CN State
1 2 3 4 5 6 7 8 9 10 11 12 13 14 15 16 17 18 19 20 21 22 X Y
Chromosome
TSB00148−LabC Ploidy=NA %AC=NA MAPD=0.462 ndSNPQC=6.79
High Gain
Gain
ND
Loss
High Loss
CN State
1 2 3 4 5 6 7 8 9 10 11 12 13 14 15 16 17 18 19 20 21 22 X Y
Chromosome
CN Agreement: TSB00148. GW−CN−Call−Agreement=90% GW−LOH−Call−Agreement=96.7%
100%
50%
0%
50%
100%
% Agreement
1 2 3 4 5 6 7 8 9 10 11 12 13 14 15 16 17 18 19 20 21 22 X Y
Chromosome

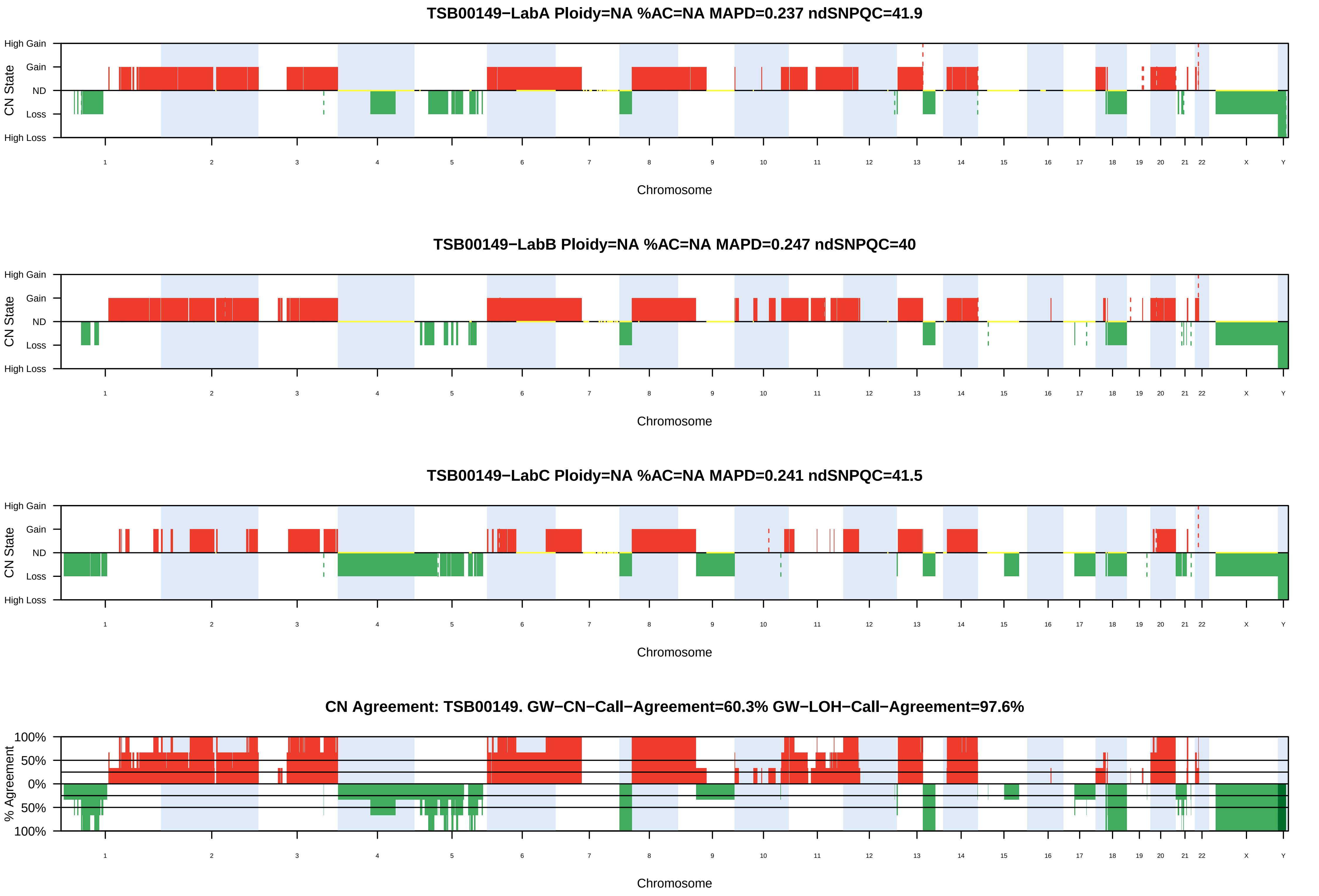

TSB00149−LabA Ploidy=NA %AC=NA MAPD=0.237 ndSNPQC=41.9
TSB00149−LabB Ploidy=NA %AC=NA MAPD=0.247 ndSNPQC=40
TSB00149−LabC Ploidy=NA %AC=NA MAPD=0.241 ndSNPQC=41.5
CN Agreement: TSB00149. GW−CN−Call−Agreement=60.3% GW−LOH−Call−Agreement=97.6%
CN State
High Gain
Gain
ND
Loss
High Loss
% Agreement
100%
50%
0%
50%
100%
1 2 3 4 5 6 7 8 9 10 11 12 13 14 15 16 17 18 19 20 21 22 X Y
Chromosome

TSB00150−LabA Ploidy=2 %AC=45 MAPD=0.269 ndSNPQC=29.5

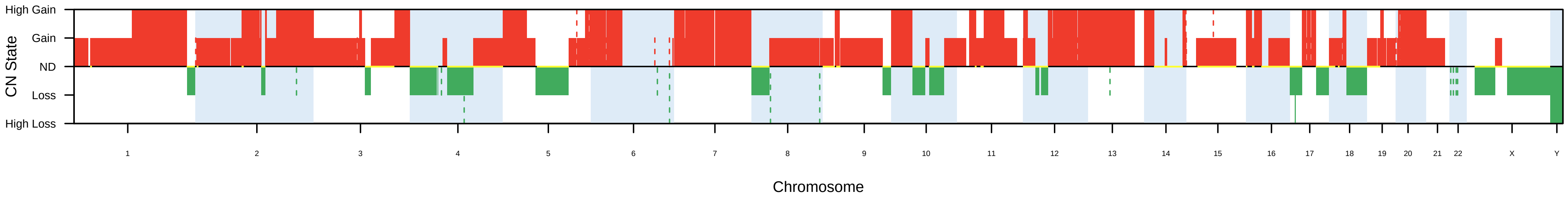

TSB00150−LabB Ploidy=NA %AC=NA MAPD=0.246 ndSNPQC=31.2

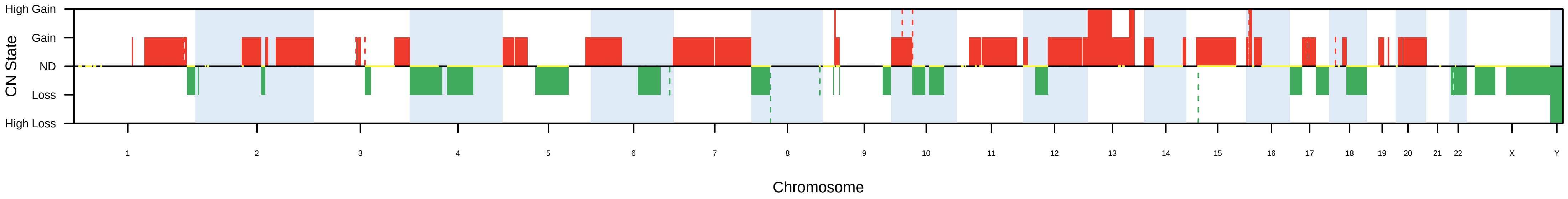

TSB00150−LabC Ploidy=2 %AC=45 MAPD=0.247 ndSNPQC=33

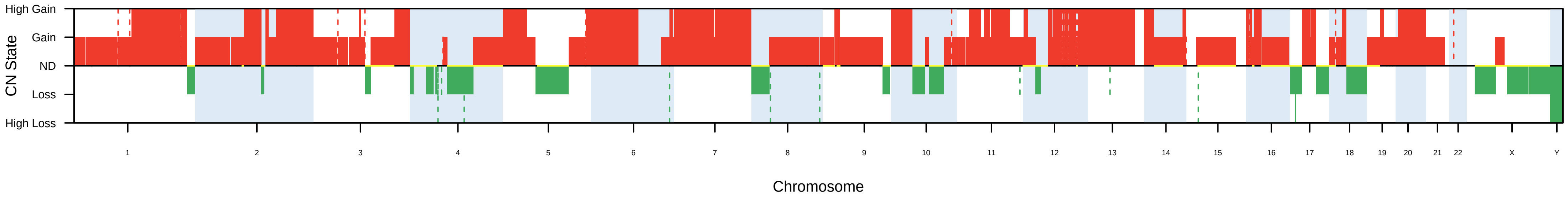

CN Agreement: TSB00150. GW−CN−Call−Agreement=25.4% GW−LOH−Call−Agreement=96.4%

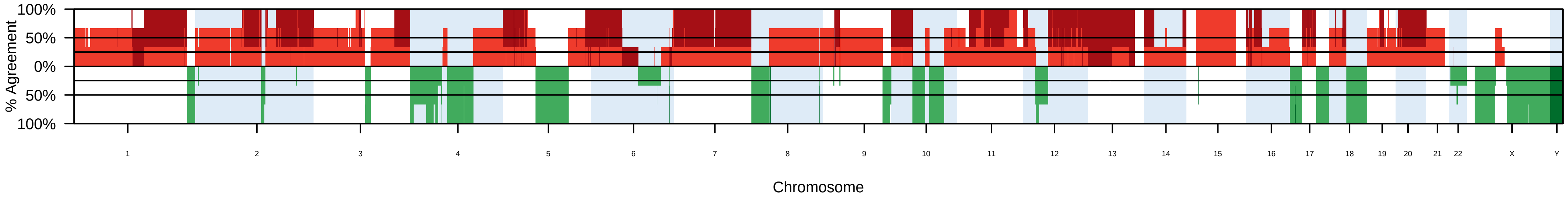

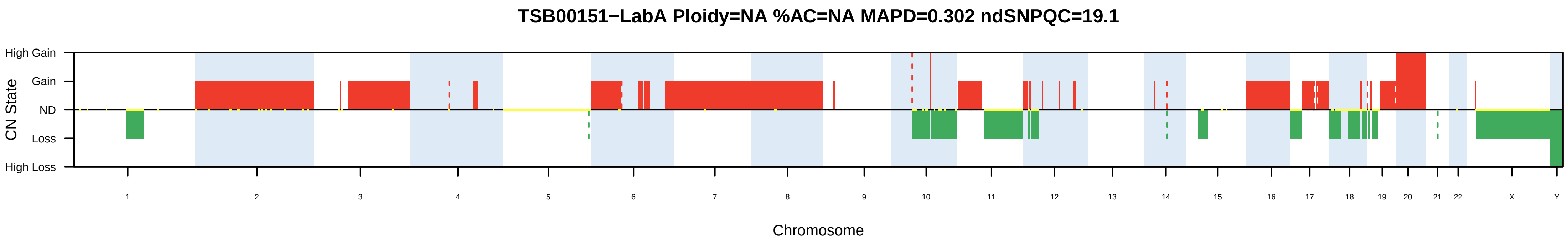

TSB00151−LabA Ploidy=NA %AC=NA MAPD=0.302 ndSNPQC=19.1
High Gain
Gain
ND
Loss
High Loss
CN State
1 2 3 4 5 6 7 8 9 10 11 12 13 14 15 16 17 18 19 20 21 22 X Y
Chromosome

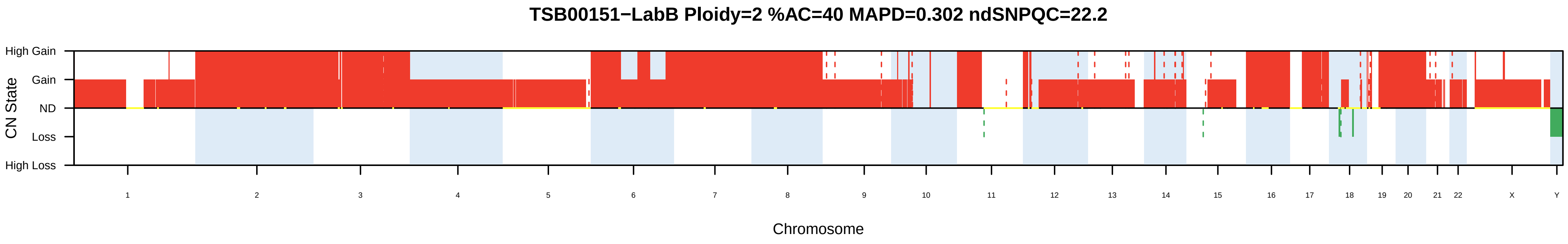

TSB00151−LabB Ploidy=2 %AC=40 MAPD=0.302 ndSNPQC=22.2
High Gain
Gain
ND
Loss
High Loss
CN State
1 2 3 4 5 6 7 8 9 10 11 12 13 14 15 16 17 18 19 20 21 22 X Y
Chromosome

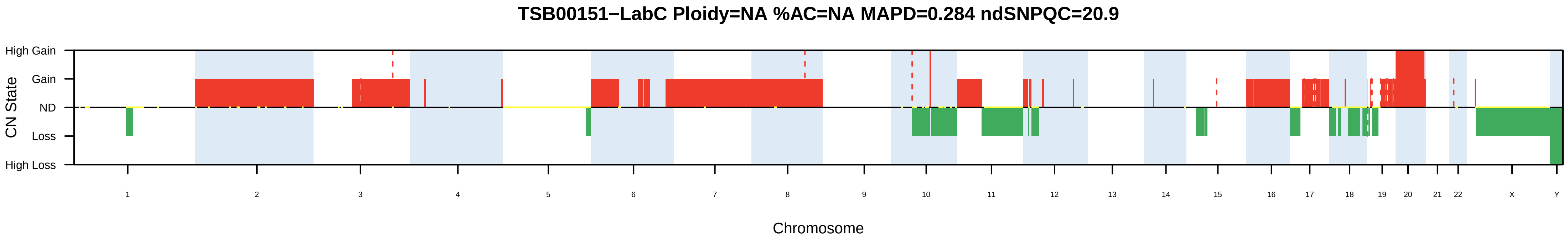

TSB00151−LabC Ploidy=NA %AC=NA MAPD=0.284 ndSNPQC=20.9
High Gain
Gain
ND
Loss
High Loss
CN State
1 2 3 4 5 6 7 8 9 10 11 12 13 14 15 16 17 18 19 20 21 22 X Y
Chromosome

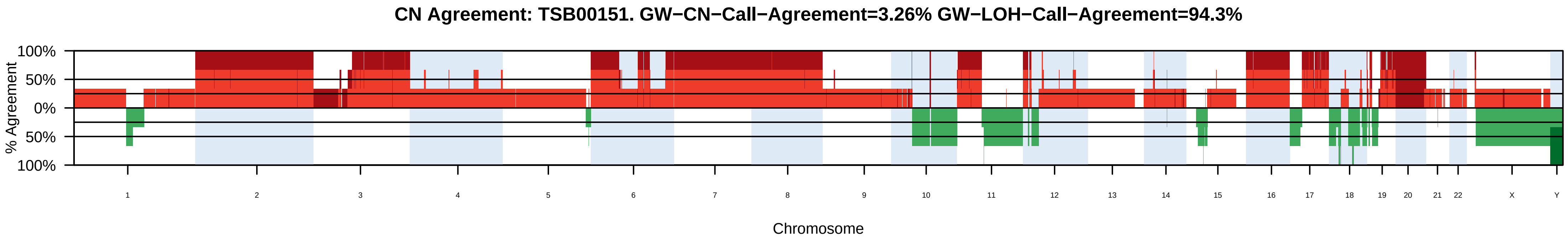

CN Agreement: TSB00151. GW−CN−Call−Agreement=3.26% GW−LOH−Call−Agreement=94.3%
100%
50%
0%
50%
100%
% Agreement
1 2 3 4 5 6 7 8 9 10 11 12 13 14 15 16 17 18 19 20 21 22 X Y
Chromosome

TSB00152−LabA Ploidy=NA %AC=NA MAPD=0.27 ndSNPQC=23.8

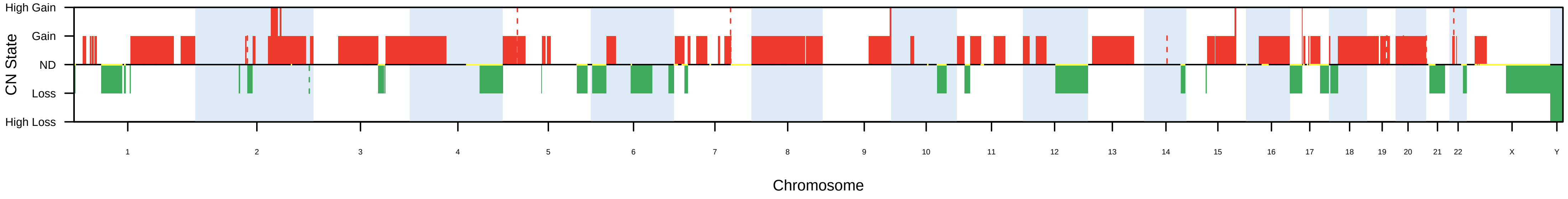

TSB00152−LabB Ploidy=2 %AC=30 MAPD=0.261 ndSNPQC=23.7

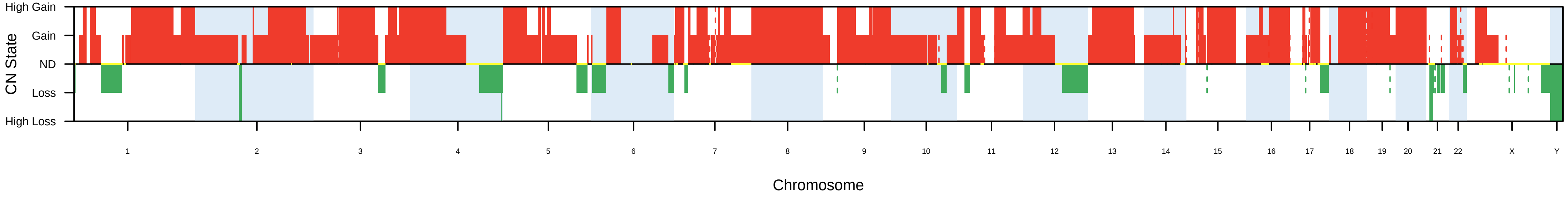

TSB00152−LabC Ploidy=2 %AC=30 MAPD=0.265 ndSNPQC=22.6

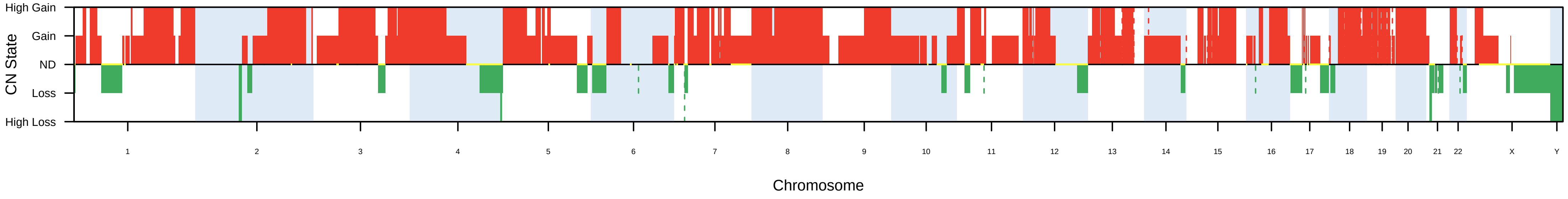

CN Agreement: TSB00152. GW−CN−Call−Agreement=15.7% GW−LOH−Call−Agreement=98.1%

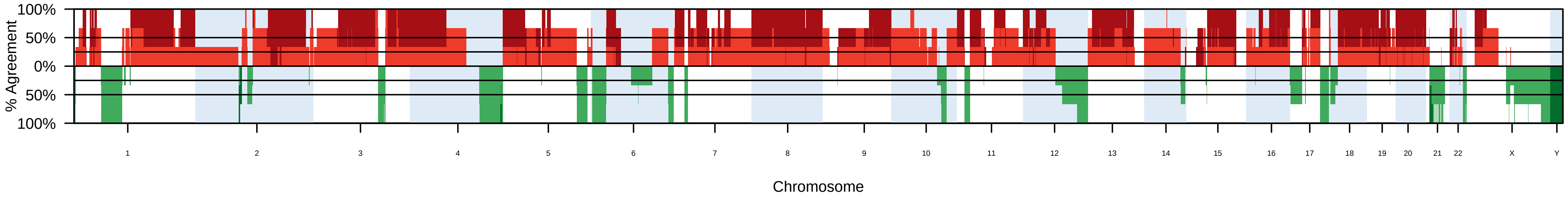

TSB00153−LabA Ploidy=NA %AC=NA MAPD=0.319 ndSNPQC=14.9

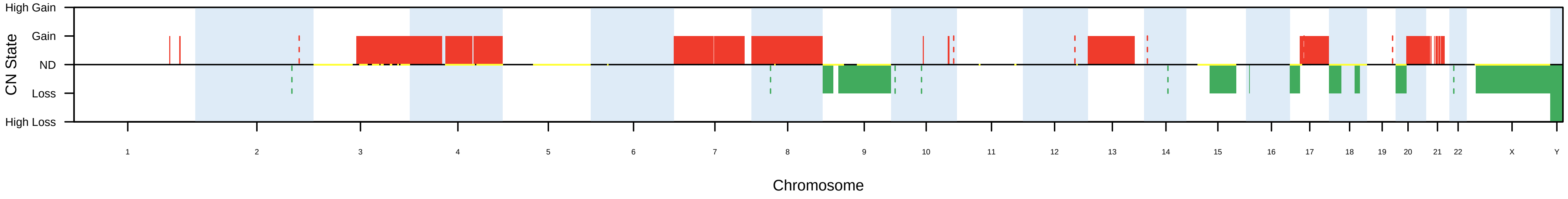

TSB00153−LabB Ploidy=NA %AC=NA MAPD=0.298 ndSNPQC=19.3

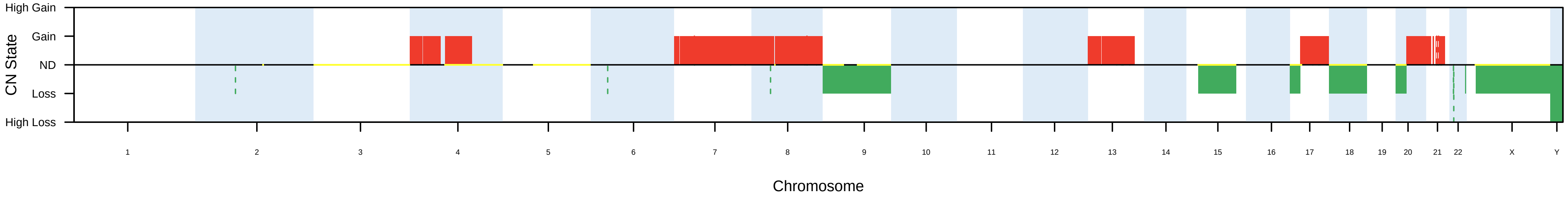

TSB00153−LabC Ploidy=NA %AC=NA MAPD=0.293 ndSNPQC=18.6

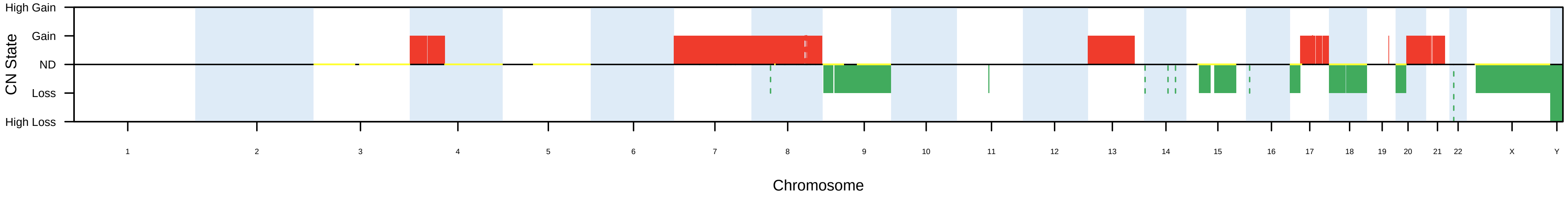

CN Agreement: TSB00153. GW−CN−Call−Agreement=86.4% GW−LOH−Call−Agreement=97.6%

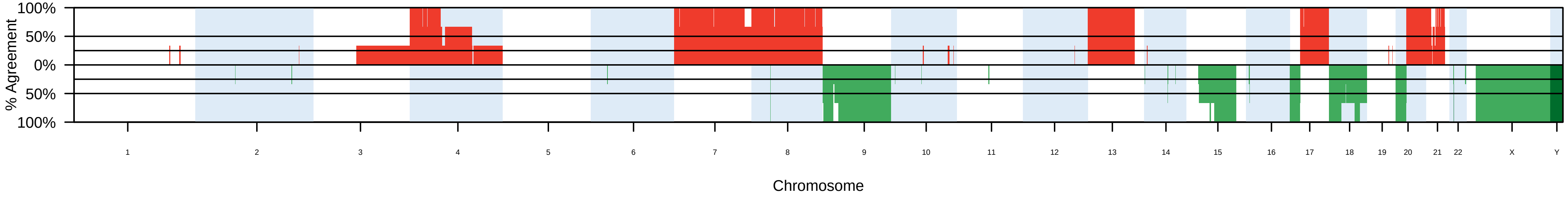

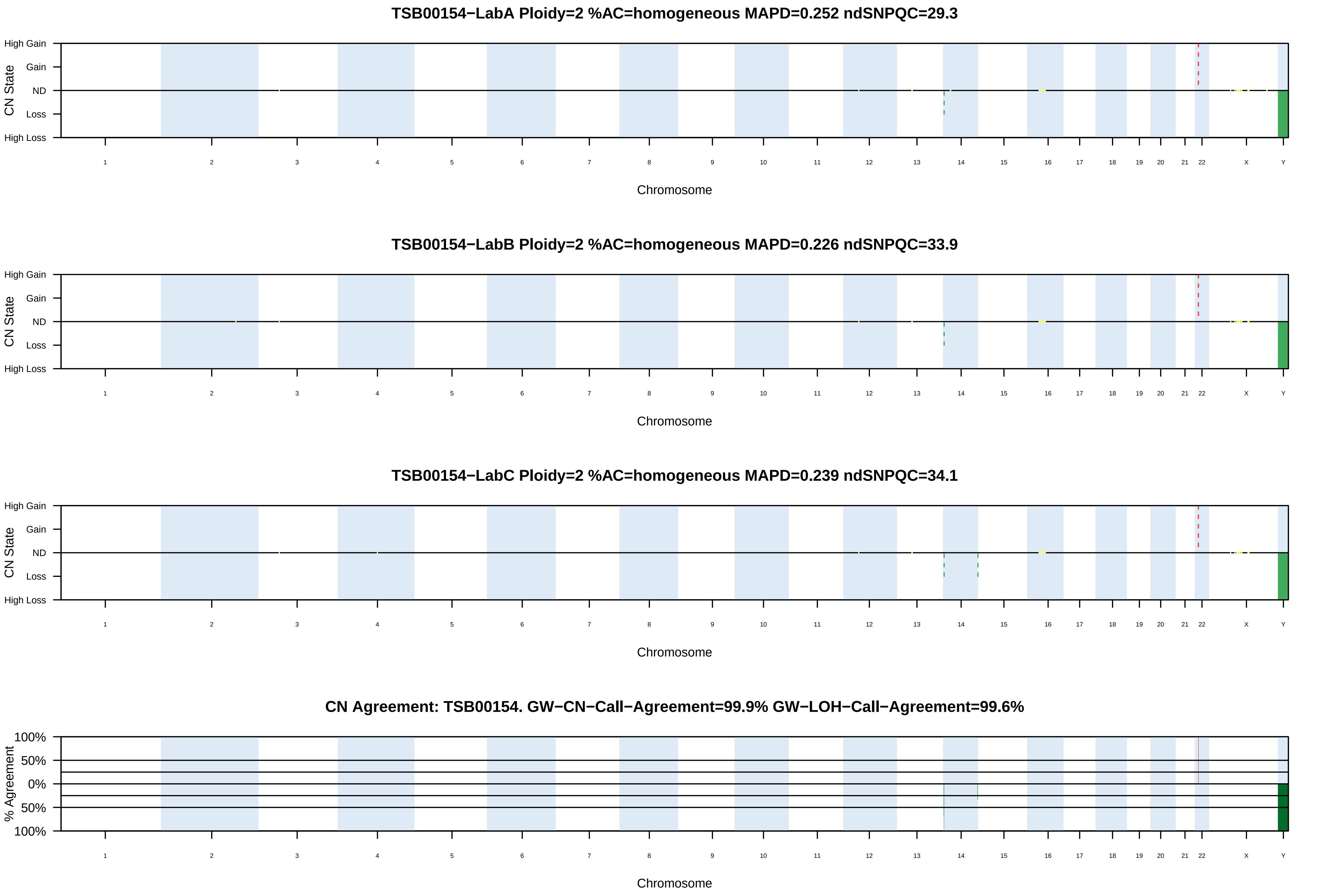

TSB00154−LabA Ploidy=2 %AC=homogeneous MAPD=0.252 ndSNPQC=29.3
TSB00154−LabB Ploidy=2 %AC=homogeneous MAPD=0.226 ndSNPQC=33.9
TSB00154−LabC Ploidy=2 %AC=homogeneous MAPD=0.239 ndSNPQC=34.1
CN Agreement: TSB00154. GW−CN−Call−Agreement=99.9% GW−LOH−Call−Agreement=99.6%
CN State
High Gain
Gain
ND
Loss
High Loss
% Agreement
100%
50%
0%
50%
100%
Chromosome
1 2 3 4 5 6 7 8 9 10 11 12 13 14 15 16 17 18 19 20 21 22 X Y

TSB00155−LabA Ploidy=2 %AC=60 MAPD=0.26 ndSNPQC=32

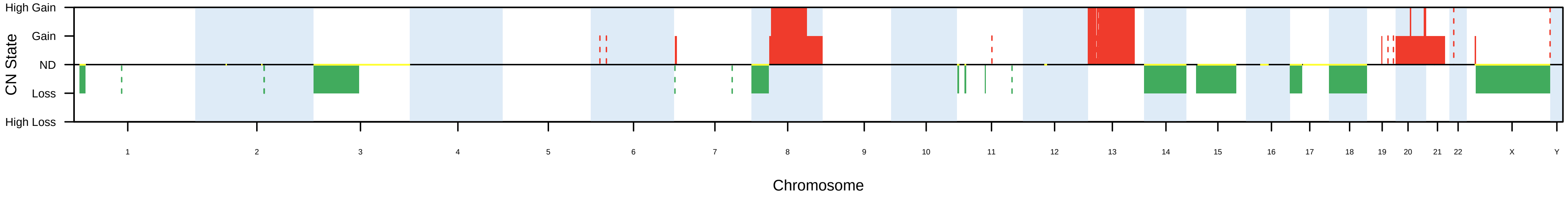

TSB00155−LabB Ploidy=2 %AC=60 MAPD=0.261 ndSNPQC=35.3

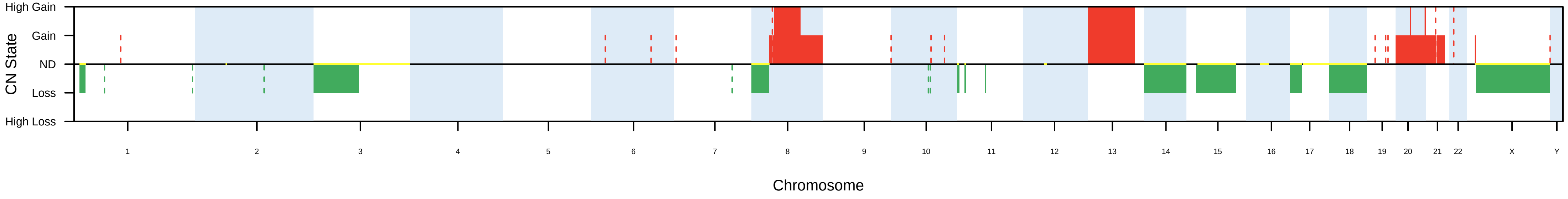

TSB00155−LabC Ploidy=2 %AC=60 MAPD=0.256 ndSNPQC=36.6

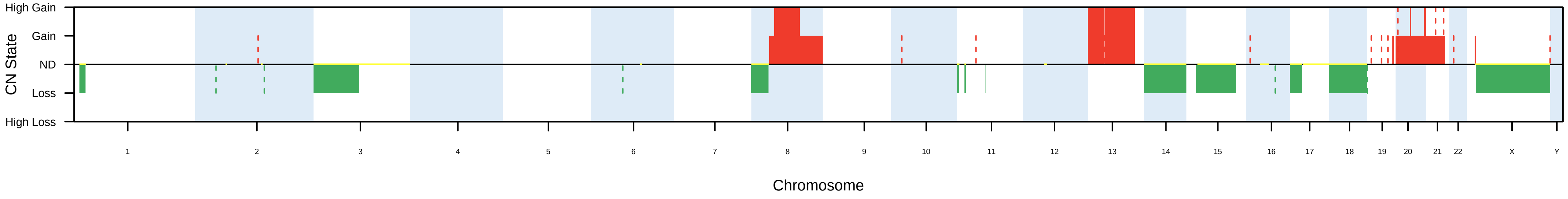

CN Agreement: TSB00155. GW−CN−Call−Agreement=97.9% GW−LOH−Call−Agreement=99.8%

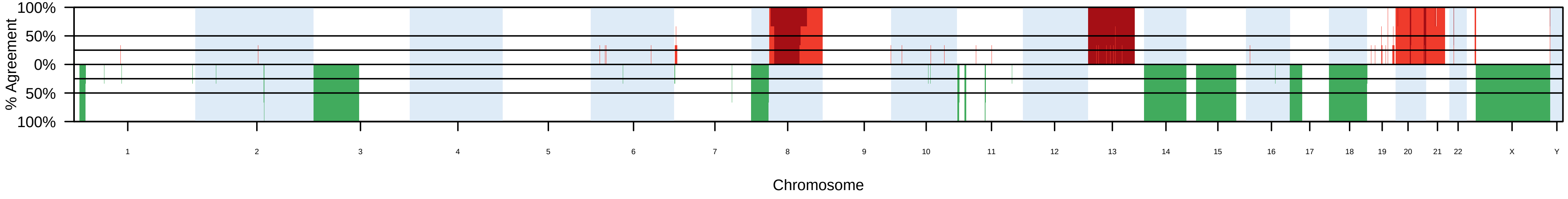

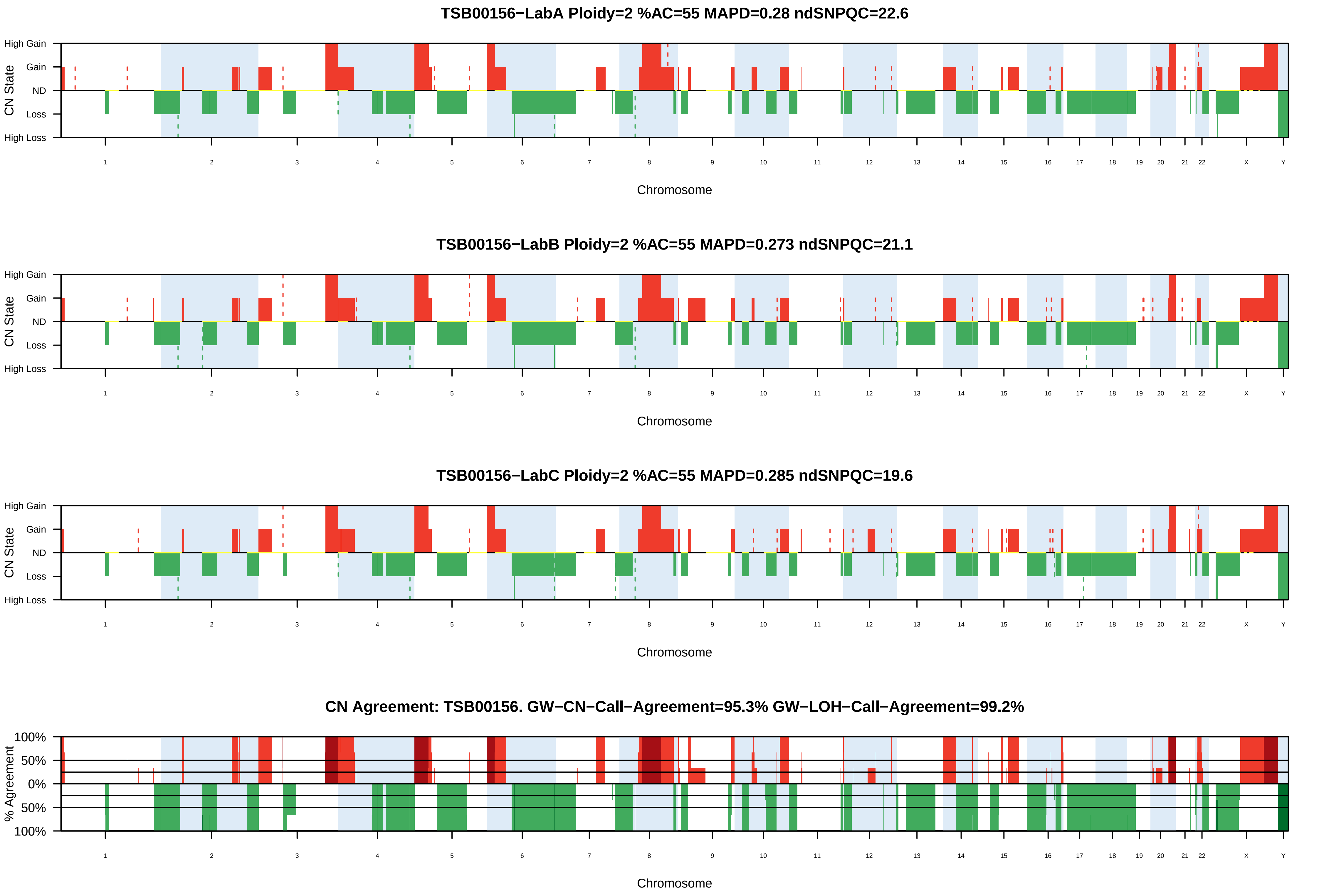
TSB00156−LabA Ploidy=2 %AC=55 MAPD=0.28 ndSNPQC=22.6
TSB00156−LabB Ploidy=2 %AC=55 MAPD=0.273 ndSNPQC=21.1
TSB00156−LabC Ploidy=2 %AC=55 MAPD=0.285 ndSNPQC=19.6
CN Agreement: TSB00156. GW−CN−Call−Agreement=95.3% GW−LOH−Call−Agreement=99.2%
CN State
High Gain
Gain
ND
Loss
High Loss
% Agreement
100%
50%
0%
Chromosome
1 2 3 4 5 6 7 8 9 10 11 12 13 14 15 16 17 18 19 20 21 22 X Y

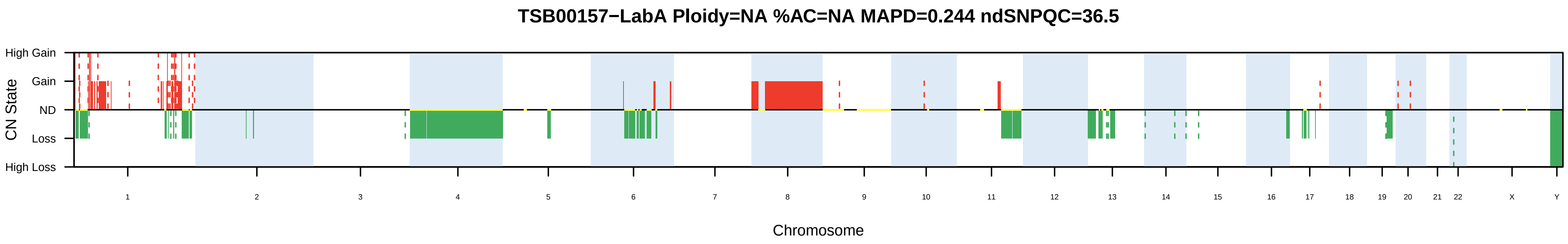
TSB00157−LabA Ploidy=NA %AC=NA MAPD=0.244 ndSNPQC=36.5
High Gain
Gain
ND
Loss
High Loss
CN State
1 2 3 4 5 6 7 8 9 10 11 12 13 14 15 16 17 18 19 20 21 22 X Y
Chromosome

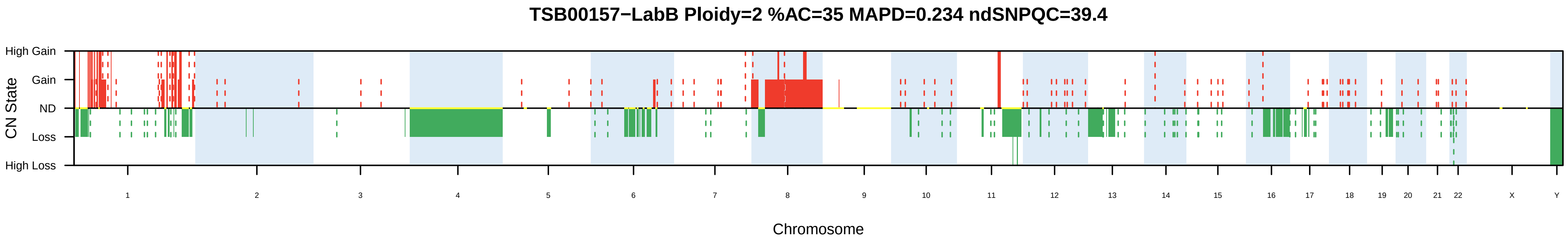
TSB00157−LabB Ploidy=2 %AC=35 MAPD=0.234 ndSNPQC=39.4
High Gain
Gain
ND
Loss
High Loss
CN State
1 2 3 4 5 6 7 8 9 10 11 12 13 14 15 16 17 18 19 20 21 22 X Y
Chromosome

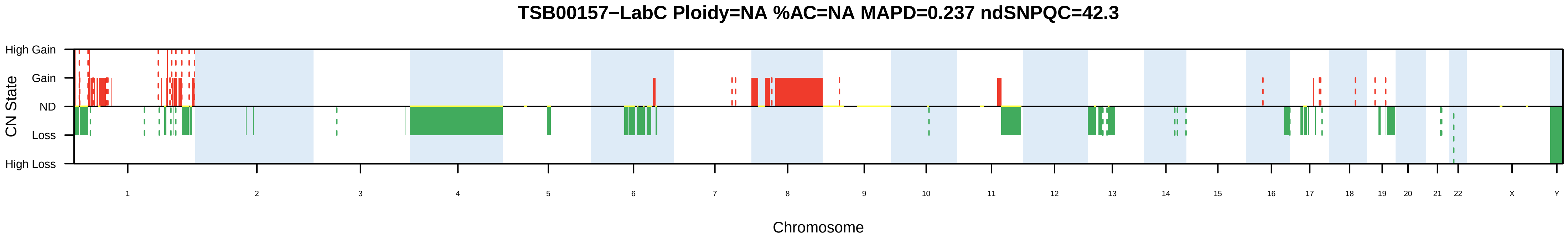
TSB00157−LabC Ploidy=NA %AC=NA MAPD=0.237 ndSNPQC=42.3
High Gain
Gain
ND
Loss
High Loss
CN State
1 2 3 4 5 6 7 8 9 10 11 12 13 14 15 16 17 18 19 20 21 22 X Y
Chromosome

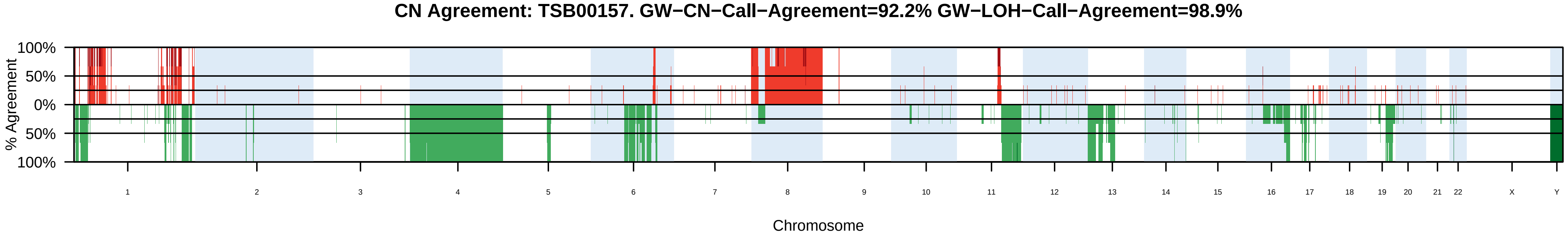
CN Agreement: TSB00157. GW−CN−Call−Agreement=92.2% GW−LOH−Call−Agreement=98.9%
100%
50%
0%
50%
100%
% Agreement
1 2 3 4 5 6 7 8 9 10 11 12 13 14 15 16 17 18 19 20 21 22 X Y
Chromosome

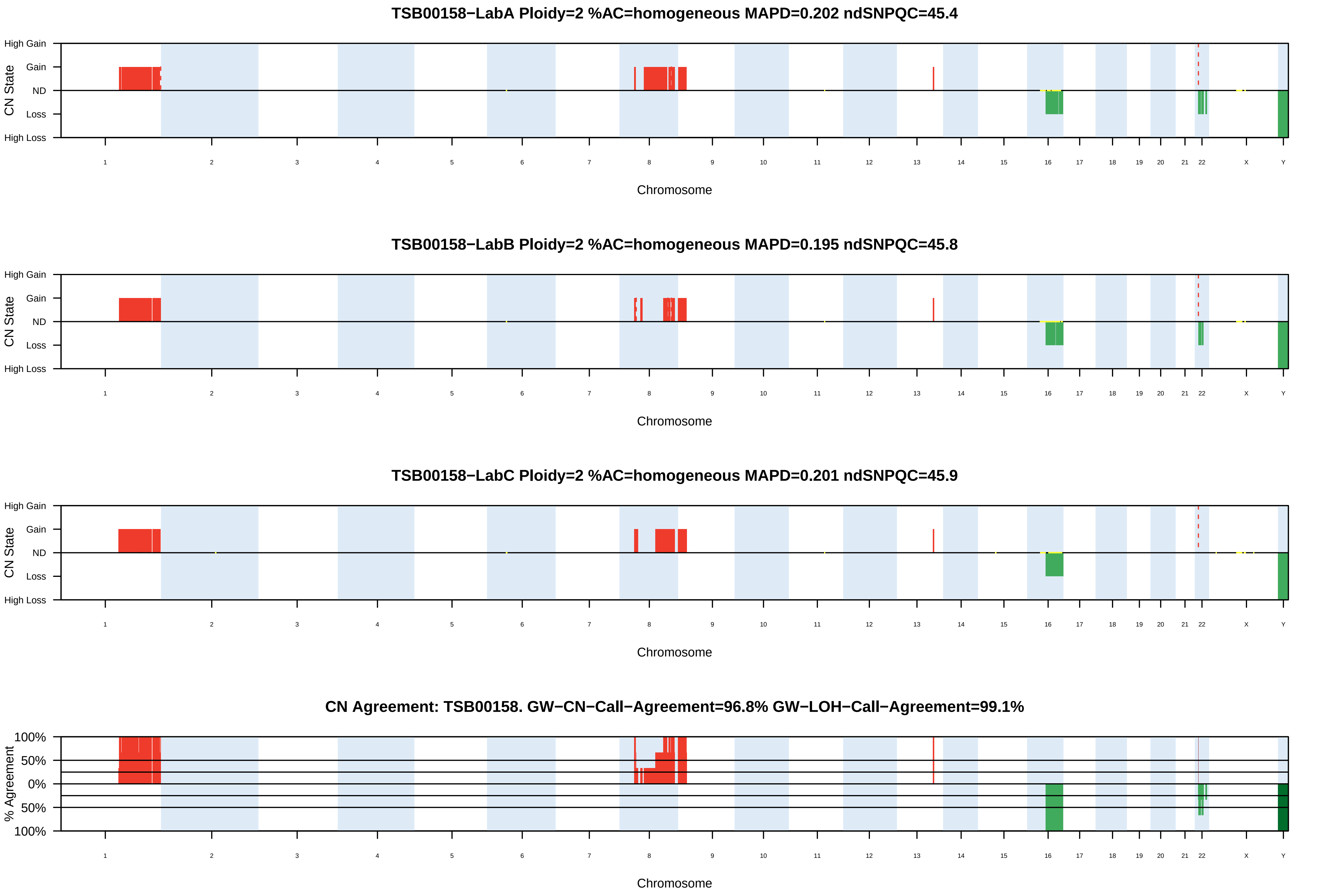
TSB00158−LabA Ploidy=2 %AC=homogeneous MAPD=0.202 ndSNPQC=45.4
TSB00158−LabB Ploidy=2 %AC=homogeneous MAPD=0.195 ndSNPQC=45.8
TSB00158−LabC Ploidy=2 %AC=homogeneous MAPD=0.201 ndSNPQC=45.9
CN Agreement: TSB00158. GW−CN−Call−Agreement=96.8% GW−LOH−Call−Agreement=99.1%
CN State
High Gain
Gain
ND
Loss
High Loss
% Agreement
100%
50%
0%
50%
100%
Chromosome
1
2
3
4
5
6
7
8
9
10
11
12
13
14
15
16
17
18
19
20
21
22
X
Y

TSB00159−LabA Ploidy=NA %AC=NA MAPD=0.251 ndSNPQC=36.7

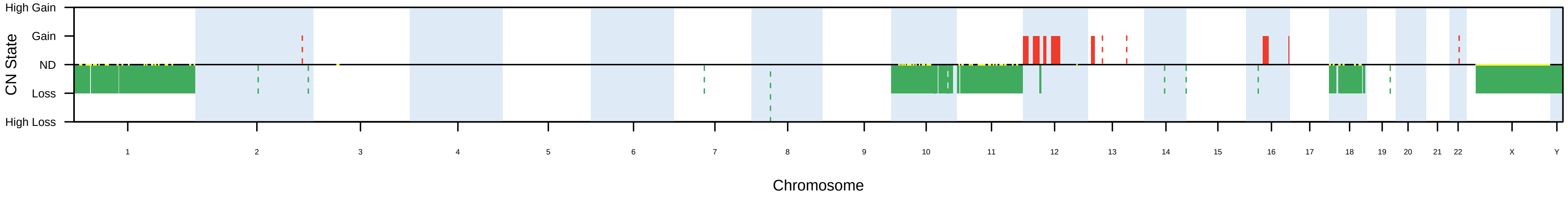

TSB00159−LabB Ploidy=2 %AC=20 MAPD=0.252 ndSNPQC=37.4

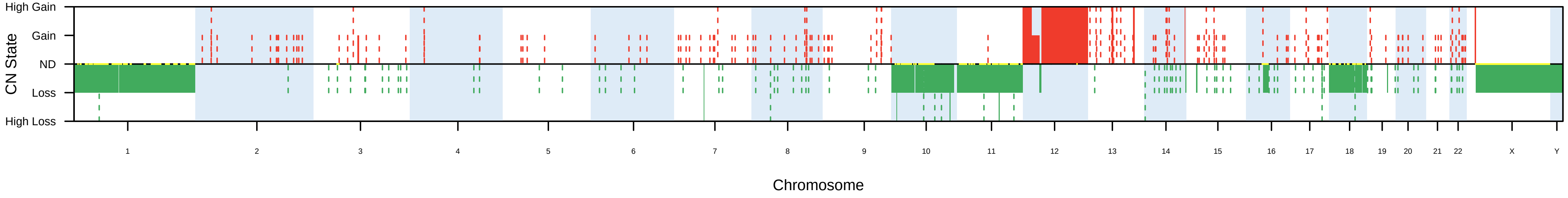

TSB00159−LabC Ploidy=NA %AC=NA MAPD=0.289 ndSNPQC=11.5

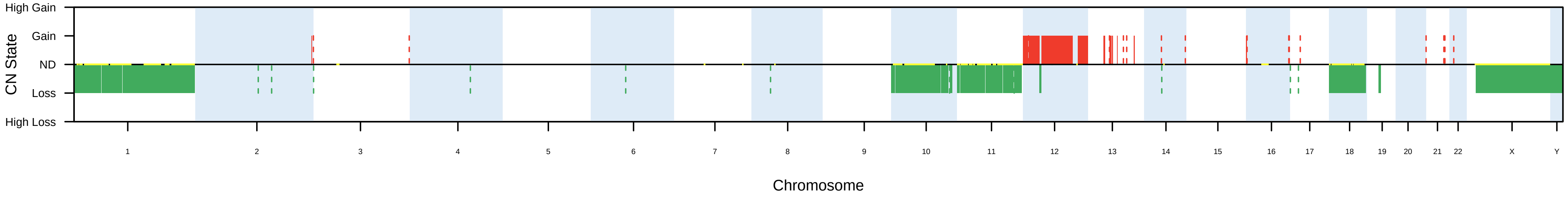

CN Agreement: TSB00159. GW−CN−Call−Agreement=90.2% GW−LOH−Call−Agreement=88.9%

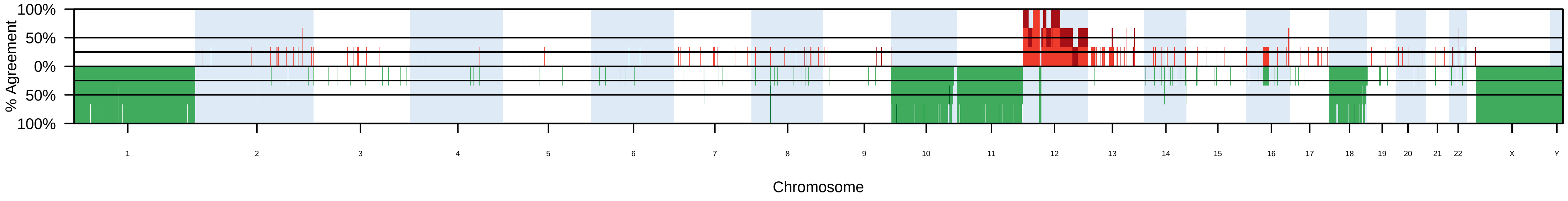

TSB00160−LabA Ploidy=2 %AC=homogeneous MAPD=0.233 ndSNPQC=33.1

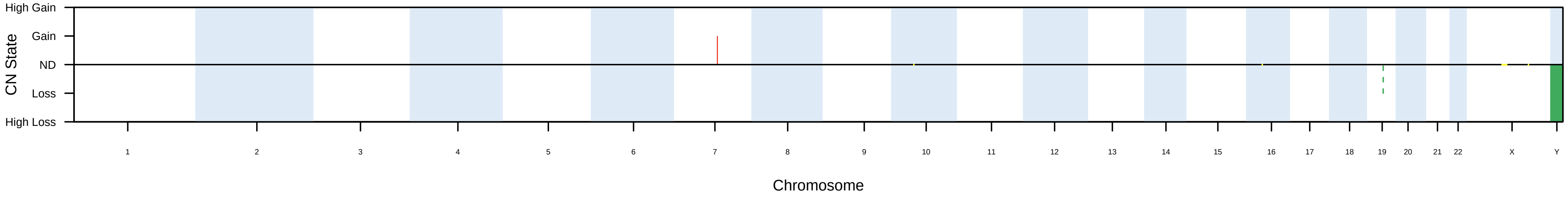

TSB00160−LabB Ploidy=2 %AC=homogeneous MAPD=0.223 ndSNPQC=32.8

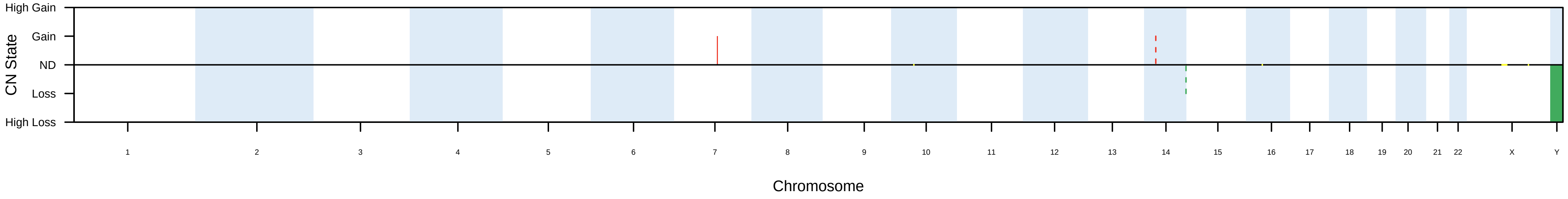

TSB00160−LabC Ploidy=2 %AC=homogeneous MAPD=0.221 ndSNPQC=35.1

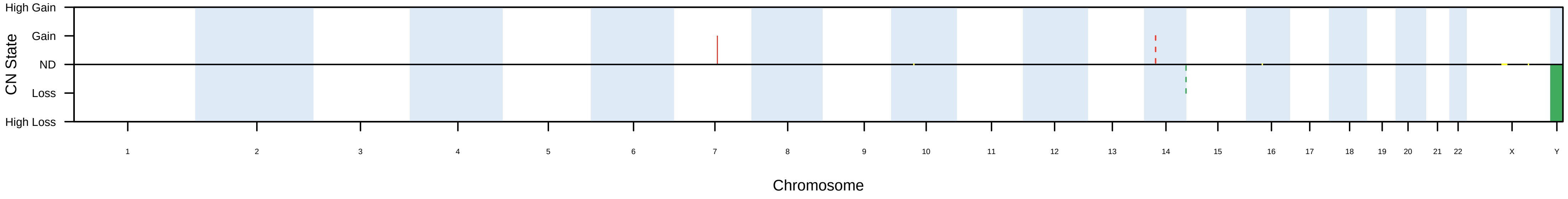

CN Agreement: TSB00160. GW−CN−Call−Agreement=100% GW−LOH−Call−Agreement=100%

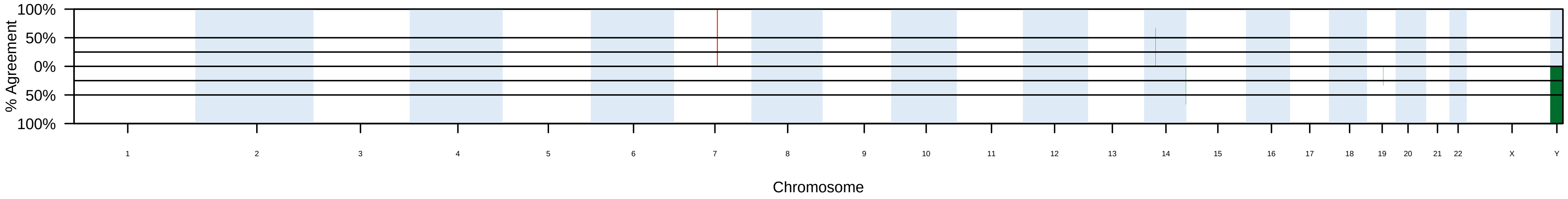

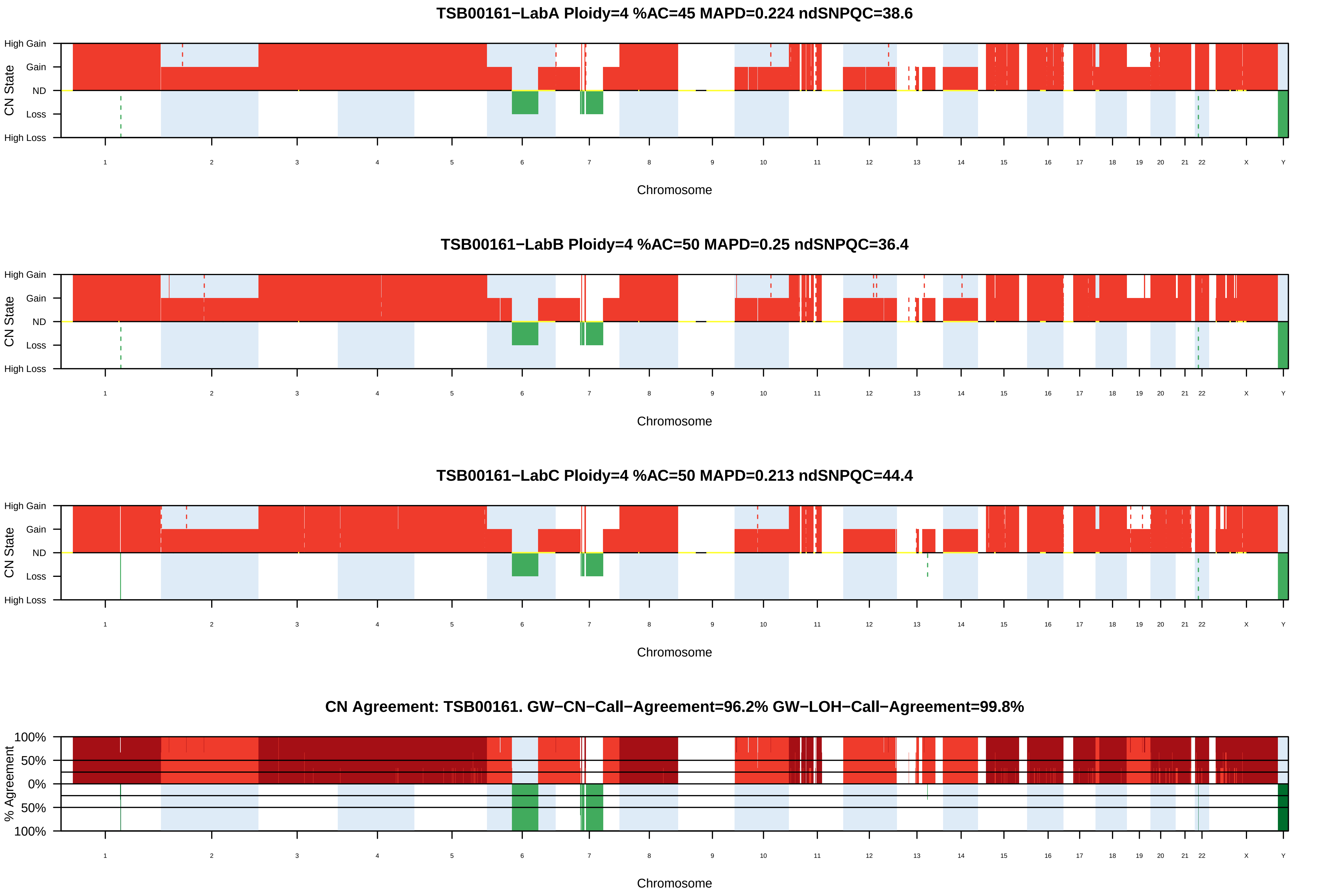
TSB00161−LabA Ploidy=4 %AC=45 MAPD=0.224 ndSNPQC=38.6
High Gain
Gain
ND
Loss
High Loss
CN State
1
2
3
4
5
6
7
8
9
10
11
12
13
14
15
16
17
18
19
20
21
22
X
Y
Chromosome
TSB00161−LabB Ploidy=4 %AC=50 MAPD=0.25 ndSNPQC=36.4
High Gain
Gain
ND
Loss
High Loss
CN State
1
2
3
4
5
6
7
8
9
10
11
12
13
14
15
16
17
18
19
20
21
22
X
Y
Chromosome
TSB00161−LabC Ploidy=4 %AC=50 MAPD=0.213 ndSNPQC=44.4
High Gain
Gain
ND
Loss
High Loss
CN State
1
2
3
4
5
6
7
8
9
10
11
12
13
14
15
16
17
18
19
20
21
22
X
Y
Chromosome
CN Agreement: TSB00161. GW−CN−Call−Agreement=96.2% GW−LOH−Call−Agreement=99.8%
100%
50%
0%
50%
100%
% Agreement
1
2
3
4
5
6
7
8
9
10
11
12
13
14
15
16
17
18
19
20
21
22
X
Y
Chromosome

TSB00162−LabA Ploidy=2 %AC=30 MAPD=0.238 ndSNPQC=25.1

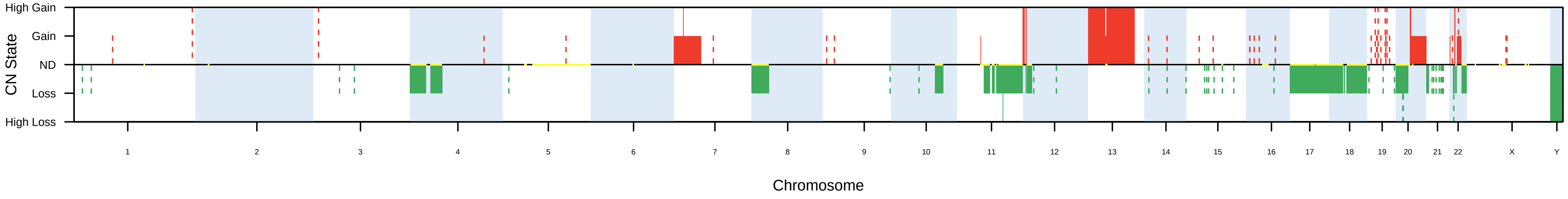

TSB00162−LabB Ploidy=2 %AC=30 MAPD=0.233 ndSNPQC=30.8

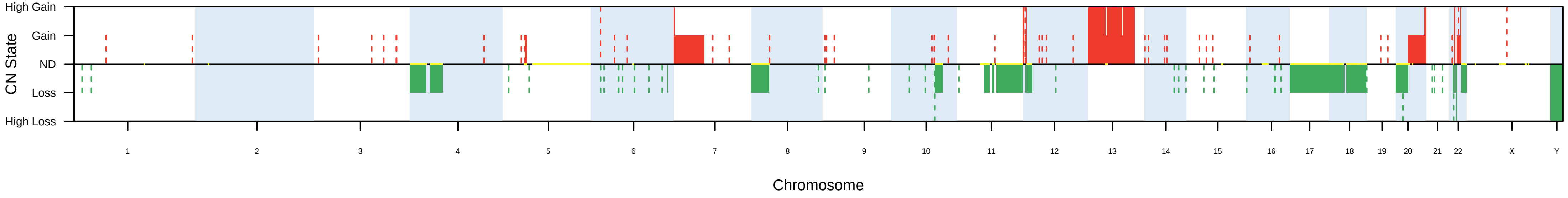

TSB00162−LabC Ploidy=2 %AC=30 MAPD=0.232 ndSNPQC=33

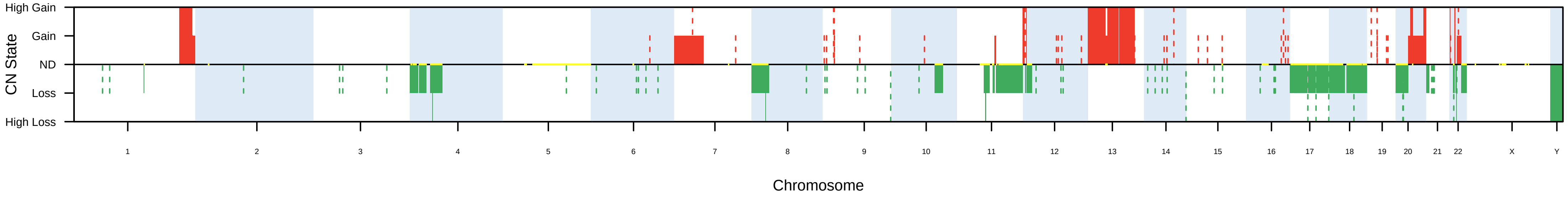

CN Agreement: TSB00162. GW−CN−Call−Agreement=94.9% GW−LOH−Call−Agreement=99%

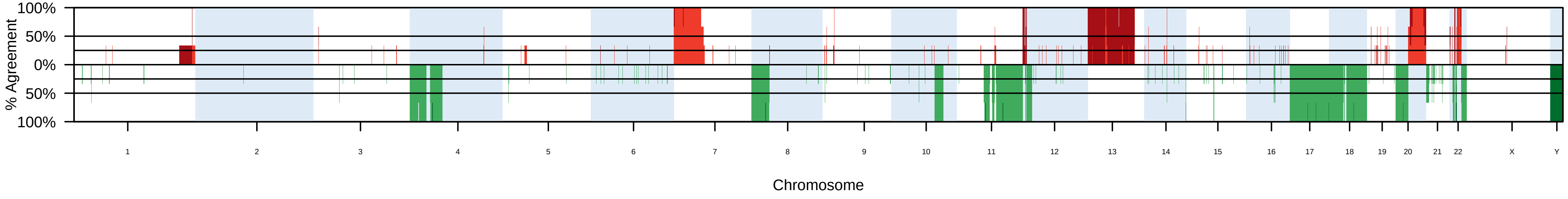

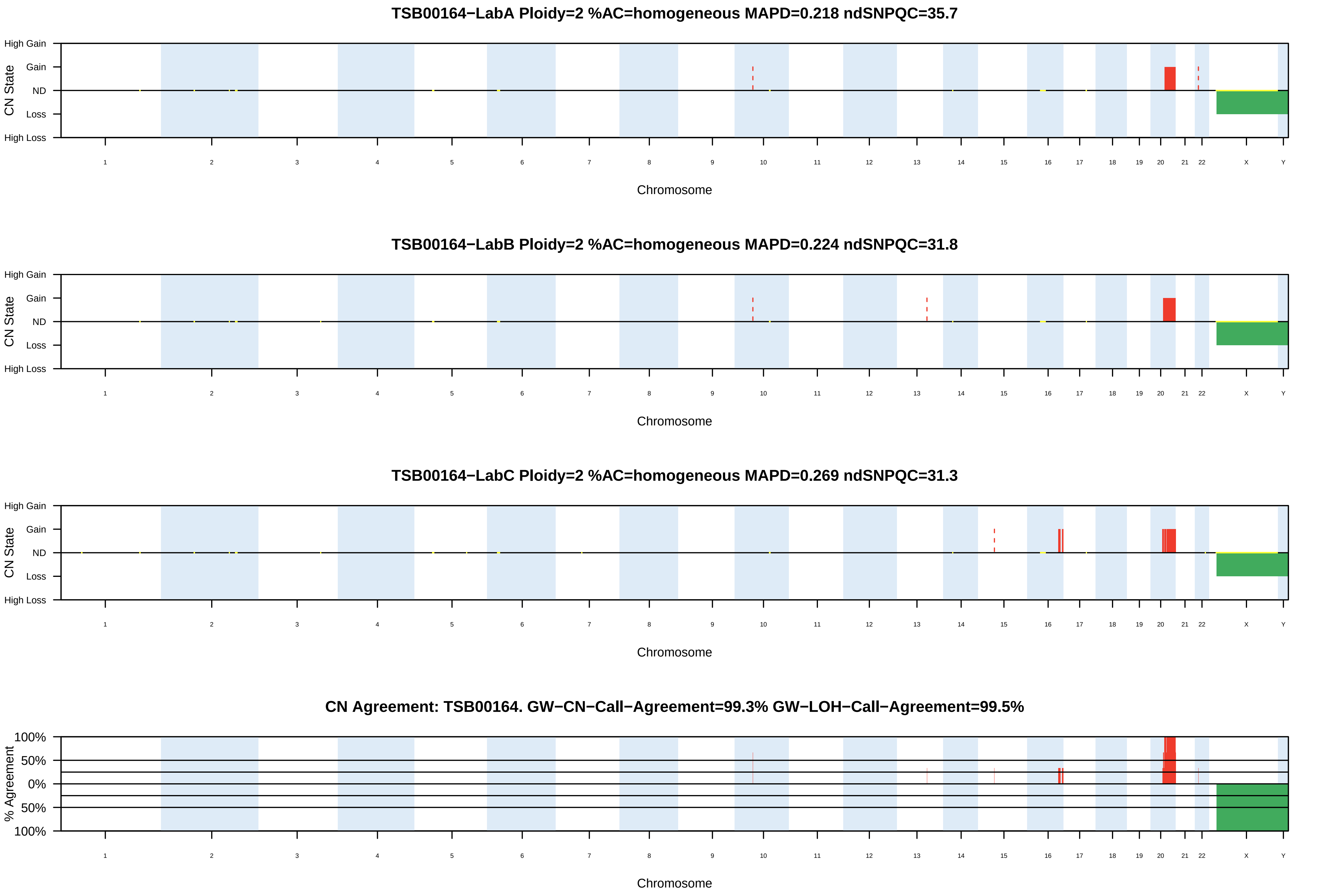

TSB00164−LabA Ploidy=2 %AC=homogeneous MAPD=0.218 ndSNPQC=35.7
High Gain
Gain
ND
Loss
High Loss
CN State
1 2 3 4 5 6 7 8 9 10 11 12 13 14 15 16 17 18 19 20 21 22 X Y
Chromosome
TSB00164−LabB Ploidy=2 %AC=homogeneous MAPD=0.224 ndSNPQC=31.8
High Gain
Gain
ND
Loss
High Loss
CN State
1 2 3 4 5 6 7 8 9 10 11 12 13 14 15 16 17 18 19 20 21 22 X Y
Chromosome
TSB00164−LabC Ploidy=2 %AC=homogeneous MAPD=0.269 ndSNPQC=31.3
High Gain
Gain
ND
Loss
High Loss
CN State
1 2 3 4 5 6 7 8 9 10 11 12 13 14 15 16 17 18 19 20 21 22 X Y
Chromosome
CN Agreement: TSB00164. GW−CN−Call−Agreement=99.3% GW−LOH−Call−Agreement=99.5%
100%
50%
0%
50%
100%
% Agreement
1 2 3 4 5 6 7 8 9 10 11 12 13 14 15 16 17 18 19 20 21 22 X Y
Chromosome

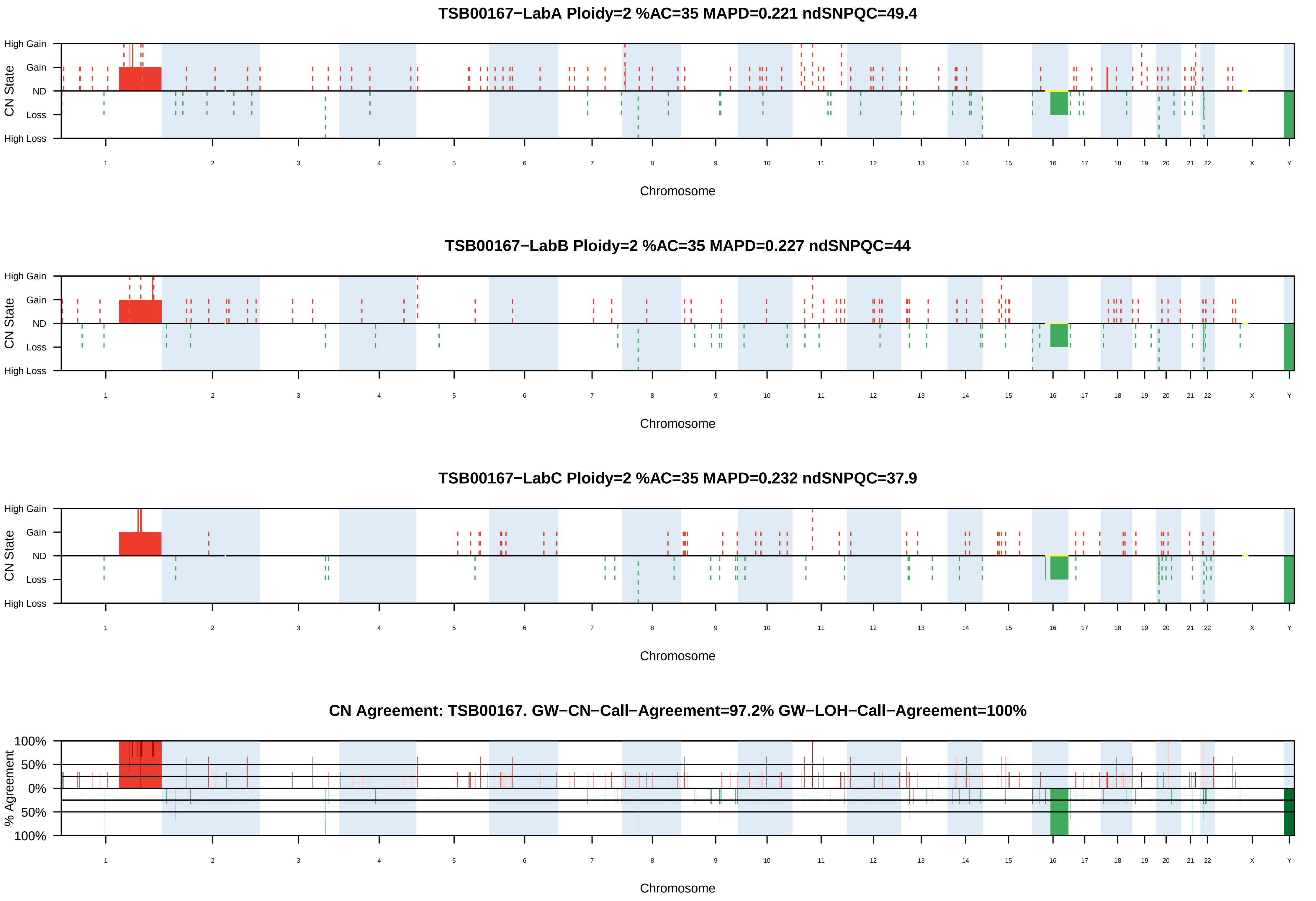
TSB00167−LabA Ploidy=2 %AC=35 MAPD=0.221 ndSNPQC=49.4
High Gain
Gain
ND
Loss
High Loss
CN State
1 2 3 4 5 6 7 8 9 10 11 12 13 14 15 16 17 18 19 20 21 22 X Y
Chromosome
TSB00167−LabB Ploidy=2 %AC=35 MAPD=0.227 ndSNPQC=44
High Gain
Gain
ND
Loss
High Loss
CN State
1 2 3 4 5 6 7 8 9 10 11 12 13 14 15 16 17 18 19 20 21 22 X Y
Chromosome
TSB00167−LabC Ploidy=2 %AC=35 MAPD=0.232 ndSNPQC=37.9
High Gain
Gain
ND
Loss
High Loss
CN State
1 2 3 4 5 6 7 8 9 10 11 12 13 14 15 16 17 18 19 20 21 22 X Y
Chromosome
CN Agreement: TSB00167. GW−CN−Call−Agreement=97.2% GW−LOH−Call−Agreement=100%
100%
50%
0%
50%
100%
% Agreement
1 2 3 4 5 6 7 8 9 10 11 12 13 14 15 16 17 18 19 20 21 22 X Y
Chromosome

TSB00168−LabA Ploidy=NA %AC=NA MAPD=0.344 ndSNPQC=14.3

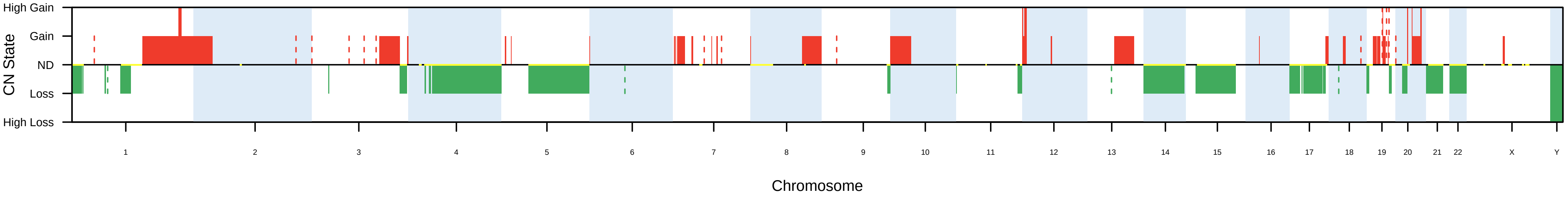

TSB00168−LabB Ploidy=NA %AC=NA MAPD=0.335 ndSNPQC=15.9

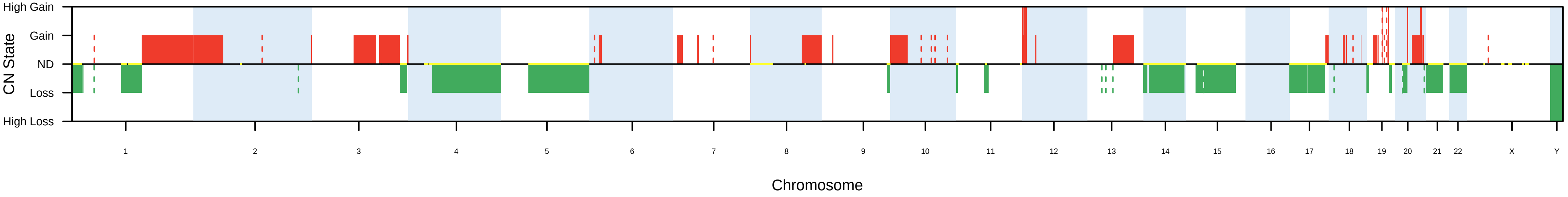

TSB00168−LabC Ploidy=NA %AC=NA MAPD=0.349 ndSNPQC=12.4

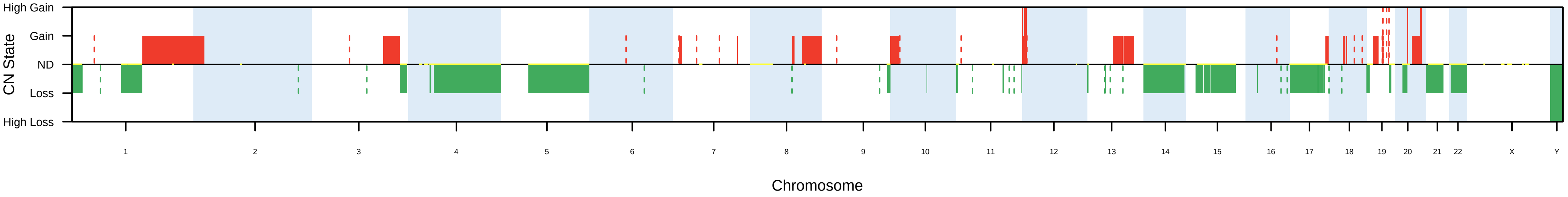

CN Agreement: TSB00168. GW−CN−Call−Agreement=90% GW−LOH−Call−Agreement=98.2%

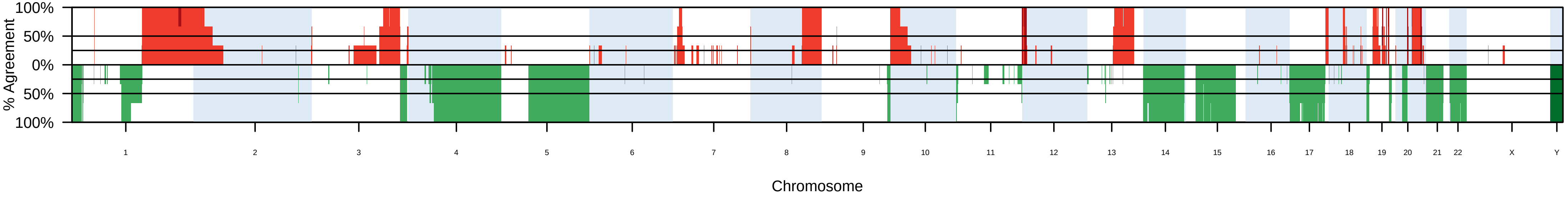

TSB00169−LabA Ploidy=2 %AC=homogeneous MAPD=0.228 ndSNPQC=50.1

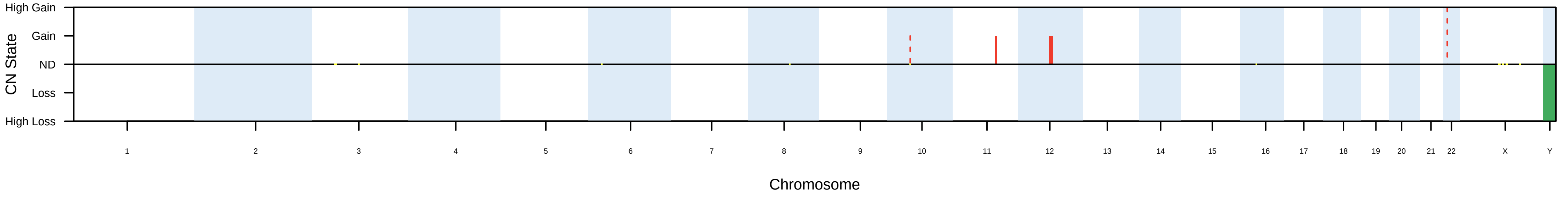

TSB00169−LabB Ploidy=2 %AC=homogeneous MAPD=0.218 ndSNPQC=44.5

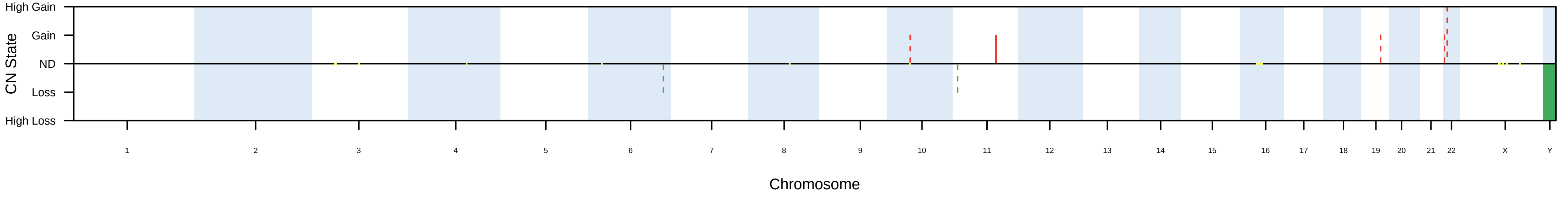

TSB00169−LabC Ploidy=2 %AC=homogeneous MAPD=0.223 ndSNPQC=49.6

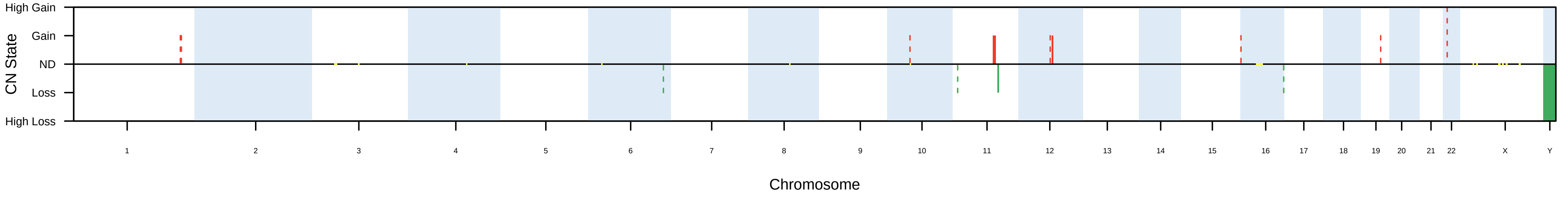

CN Agreement: TSB00169. GW−CN−Call−Agreement=99.2% GW−LOH−Call−Agreement=99.3%

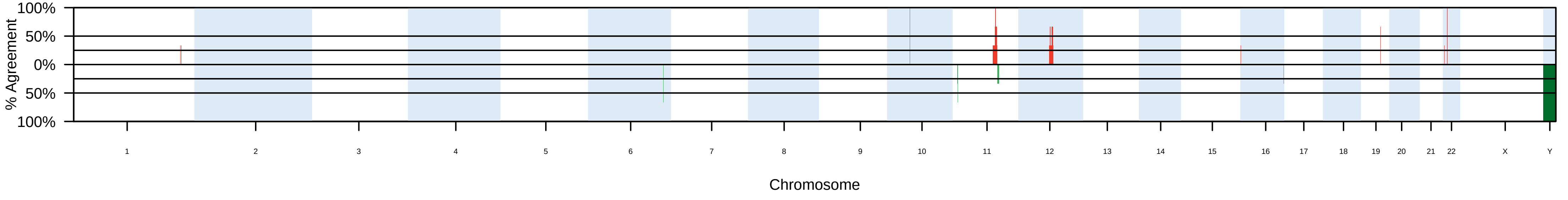

TSB00170−LabA Ploidy=NA %AC=NA MAPD=0.346 ndSNPQC=17

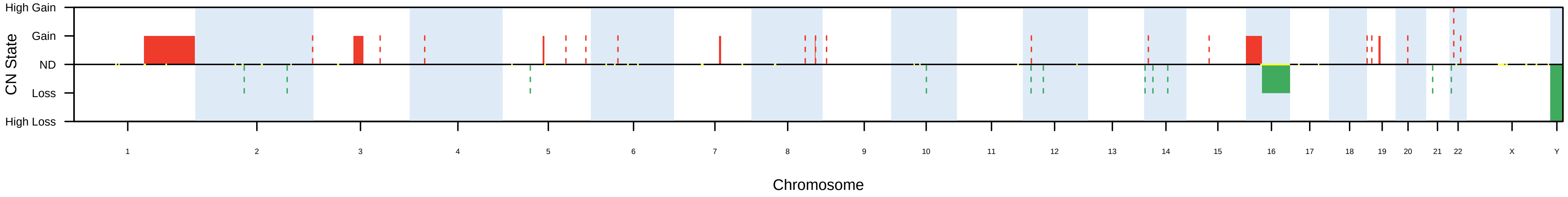

TSB00170−LabB Ploidy=NA %AC=NA MAPD=0.345 ndSNPQC=16

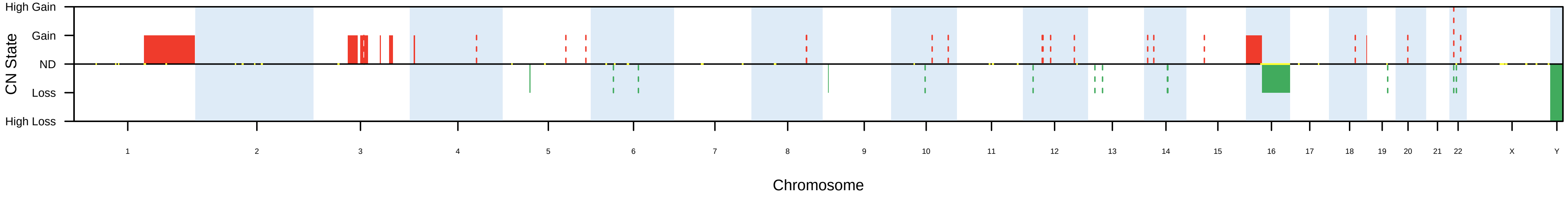

TSB00170−LabC Ploidy=NA %AC=NA MAPD=0.334 ndSNPQC=17.4

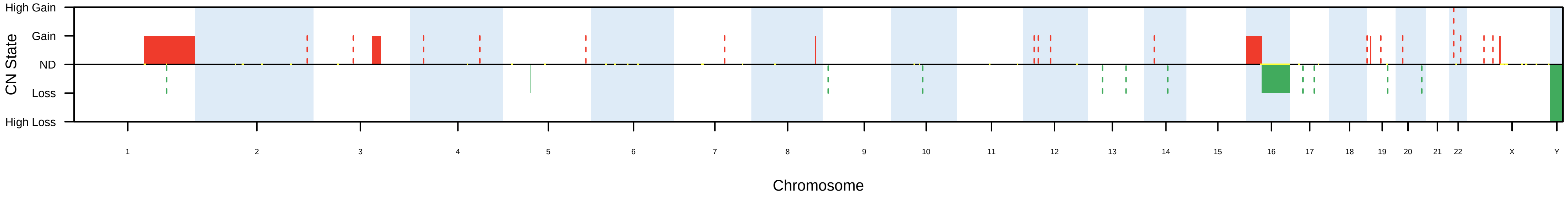

CN Agreement: TSB00170. GW−CN−Call−Agreement=96.2% GW−LOH−Call−Agreement=98.4%

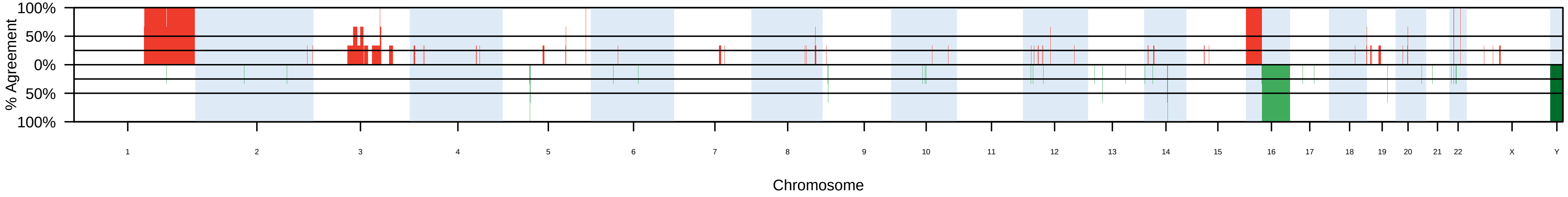

TSB00171−LabA Ploidy=2 %AC=homogeneous MAPD=0.213 ndSNPQC=51.5

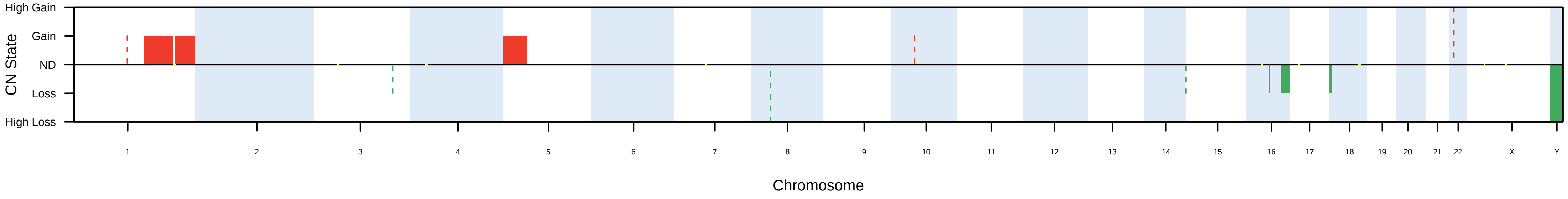

TSB00171−LabB Ploidy=2 %AC=homogeneous MAPD=0.218 ndSNPQC=32.9

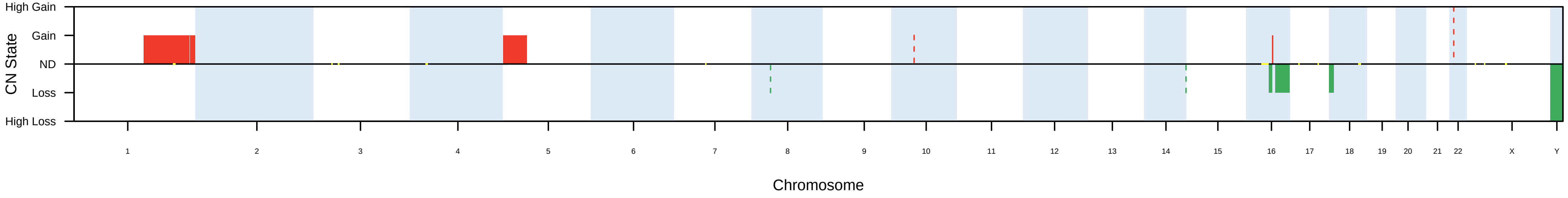

TSB00171−LabC Ploidy=2 %AC=homogeneous MAPD=0.24 ndSNPQC=38.2

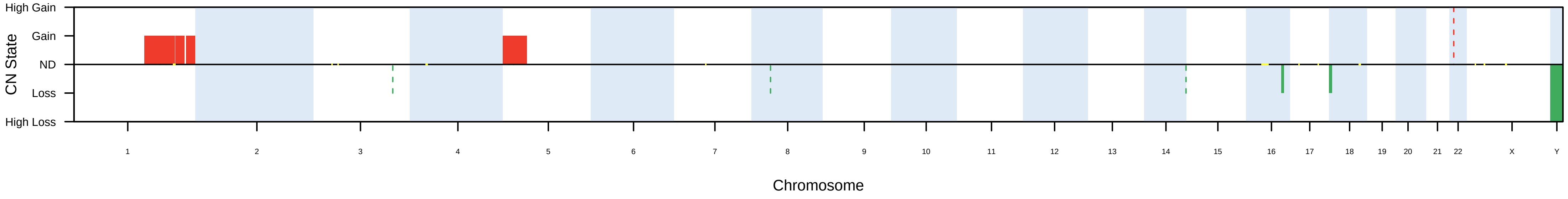

CN Agreement: TSB00171. GW−CN−Call−Agreement=98.2% GW−LOH−Call−Agreement=99.3%

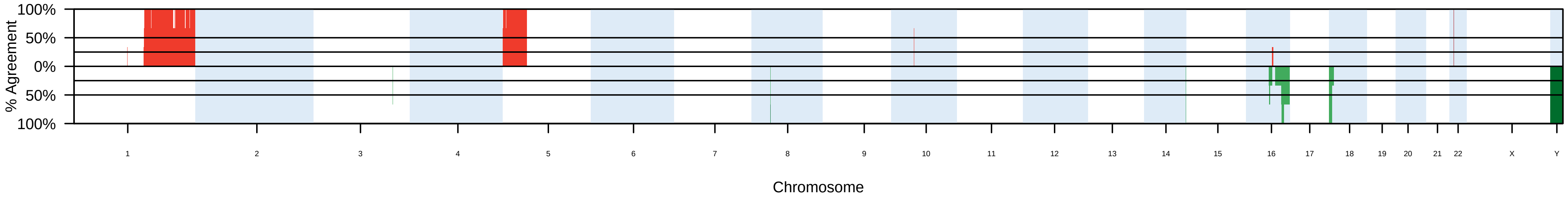

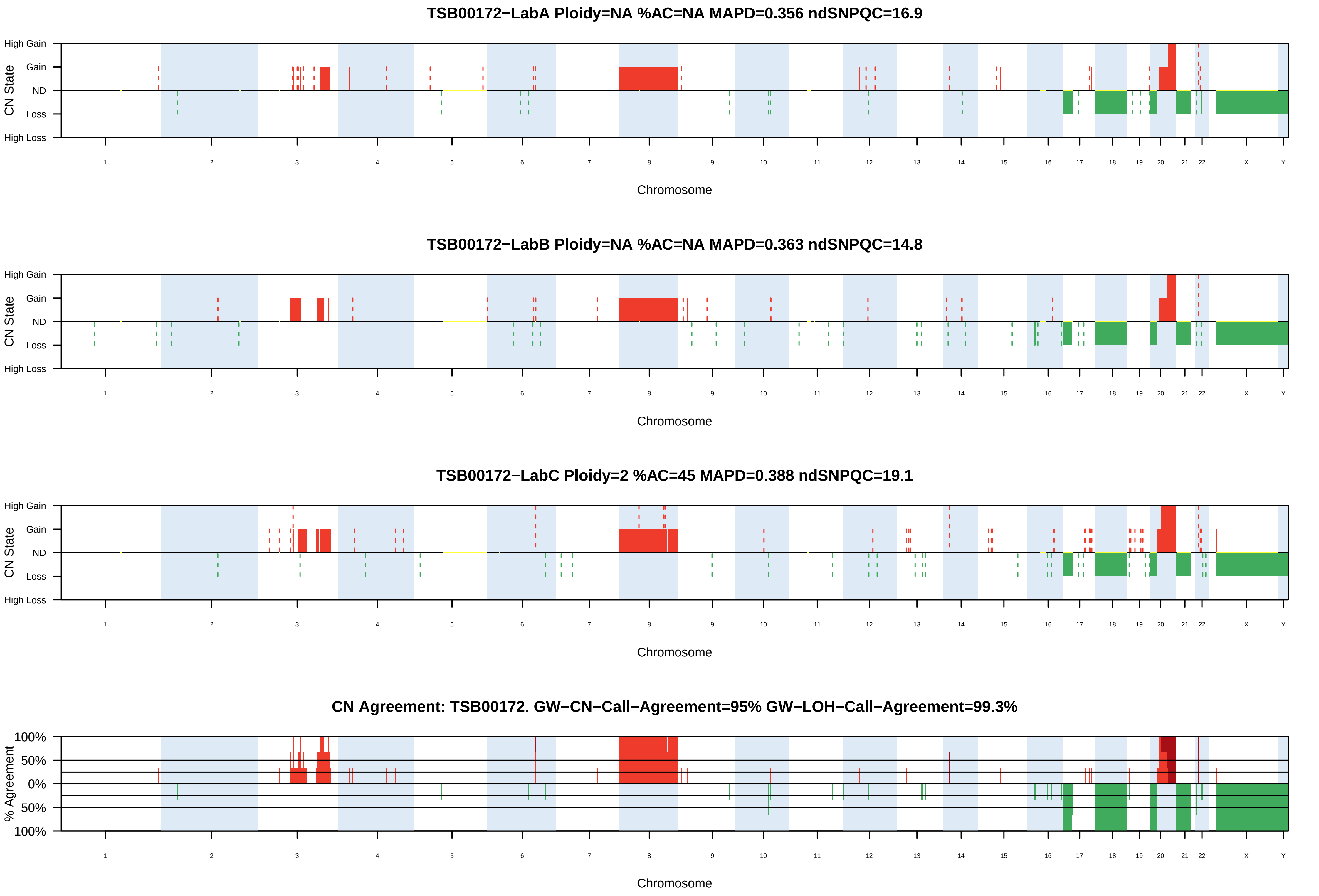
TSB00172−LabA Ploidy=NA %AC=NA MAPD=0.356 ndSNPQC=16.9
CN State
High Gain
Gain
ND
Loss
High Loss
1 2 3 4 5 6 7 8 9 10 11 12 13 14 15 16 17 18 19 20 21 22 X Y
Chromosome
TSB00172−LabB Ploidy=NA %AC=NA MAPD=0.363 ndSNPQC=14.8
CN State
High Gain
Gain
ND
Loss
High Loss
1 2 3 4 5 6 7 8 9 10 11 12 13 14 15 16 17 18 19 20 21 22 X Y
Chromosome
TSB00172−LabC Ploidy=2 %AC=45 MAPD=0.388 ndSNPQC=19.1
CN State
High Gain
Gain
ND
Loss
High Loss
1 2 3 4 5 6 7 8 9 10 11 12 13 14 15 16 17 18 19 20 21 22 X Y
Chromosome
CN Agreement: TSB00172. GW−CN−Call−Agreement=95% GW−LOH−Call−Agreement=99.3%
% Agreement
100%
50%
0%
50%
100%
1 2 3 4 5 6 7 8 9 10 11 12 13 14 15 16 17 18 19 20 21 22 X Y
Chromosome

TSB00173−LabA Ploidy=2 %AC=30 MAPD=0.212 ndSNPQC=45.6

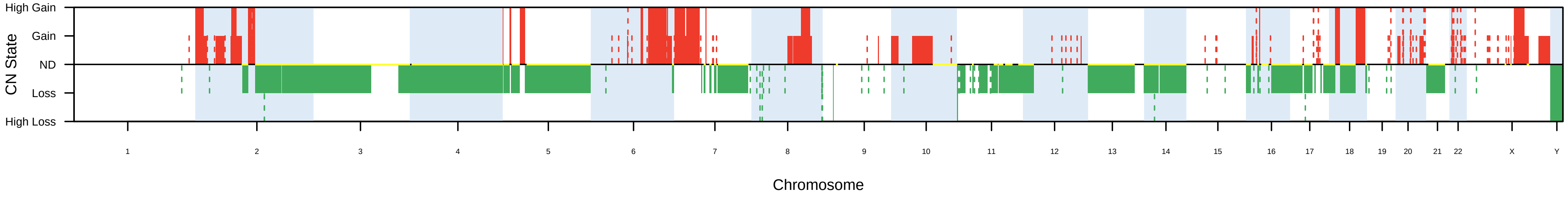

TSB00173−LabB Ploidy=NA %AC=NA MAPD=0.254 ndSNPQC=20.8

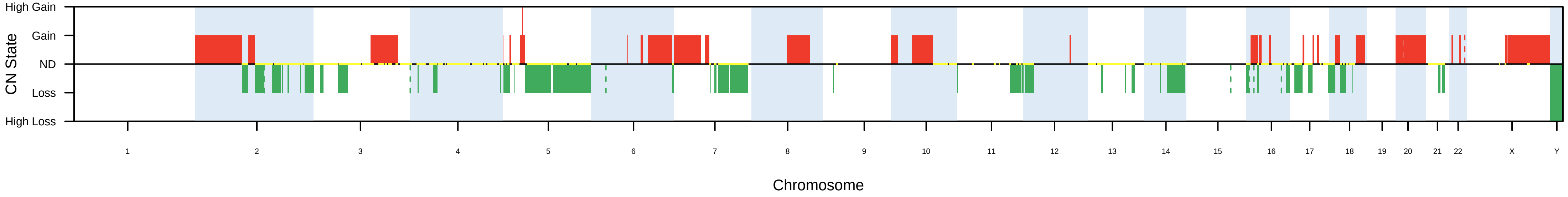

TSB00173−LabC Ploidy=2 %AC=30 MAPD=0.237 ndSNPQC=43.8

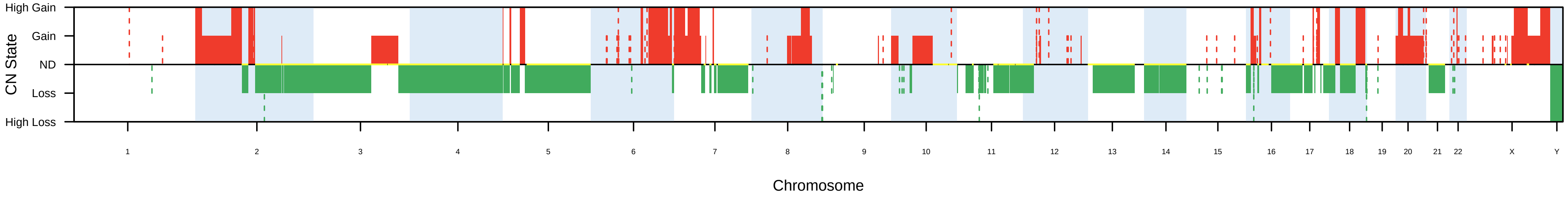

CN Agreement: TSB00173. GW−CN−Call−Agreement=56.2% GW−LOH−Call−Agreement=93.7%

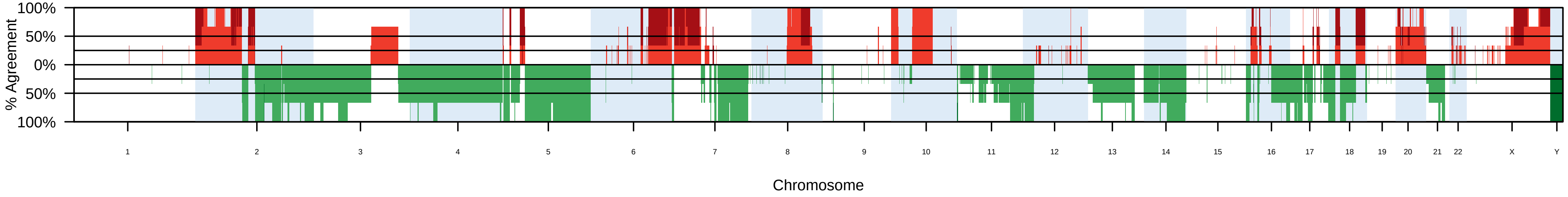

TSB00175−LabA Ploidy=2 %AC=55 MAPD=0.239 ndSNPQC=37.4

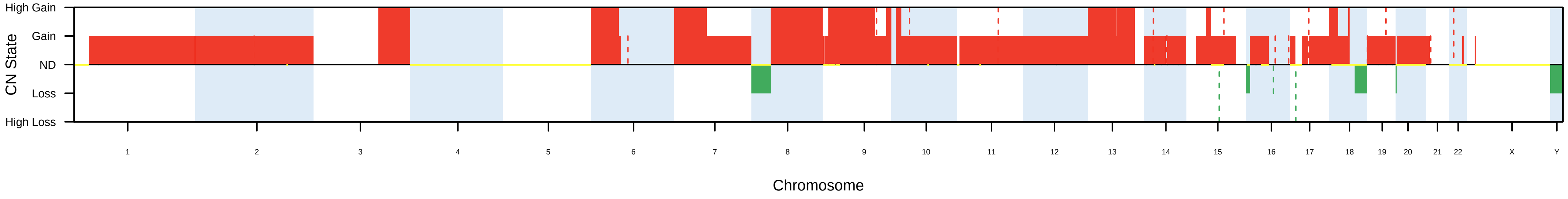

TSB00175−LabB Ploidy=2 %AC=60 MAPD=0.238 ndSNPQC=36.1

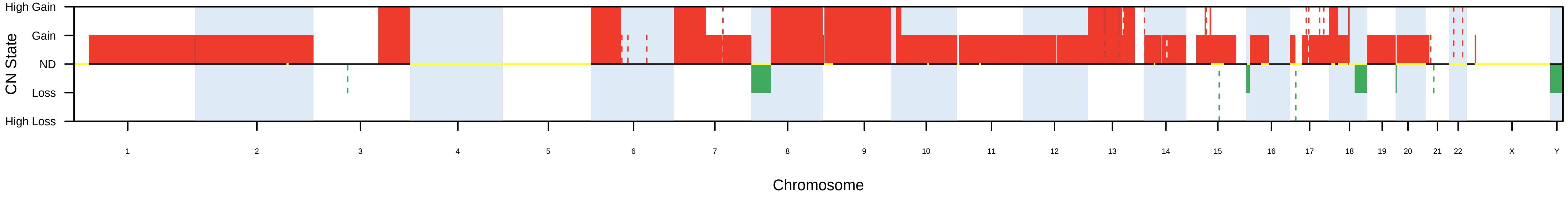

TSB00175−LabC Ploidy=2 %AC=55 MAPD=0.294 ndSNPQC=27.8

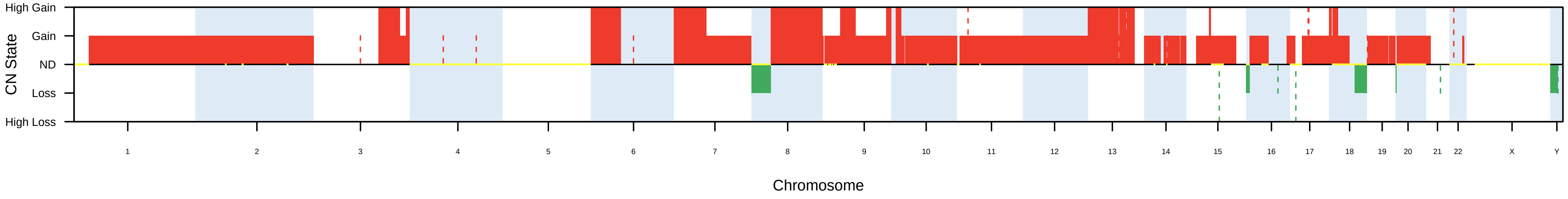

CN Agreement: TSB00175. GW−CN−Call−Agreement=93.4% GW−LOH−Call−Agreement=98.7%

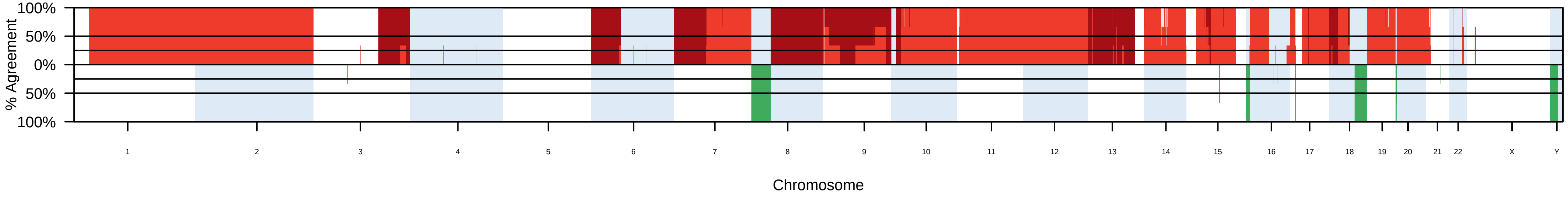

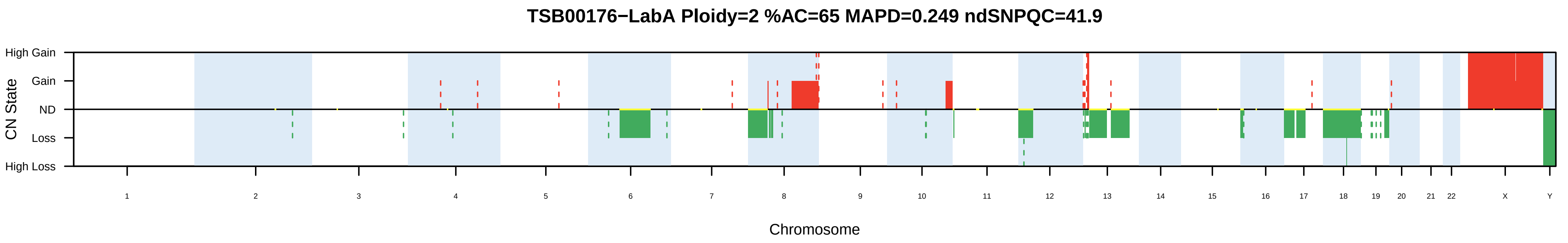
TSB00176−LabA Ploidy=2 %AC=65 MAPD=0.249 ndSNPQC=41.9
CN State
High Gain
Gain
ND
Loss
High Loss
1 2 3 4 5 6 7 8 9 10 11 12 13 14 15 16 17 18 19 20 21 22 X Y
Chromosome

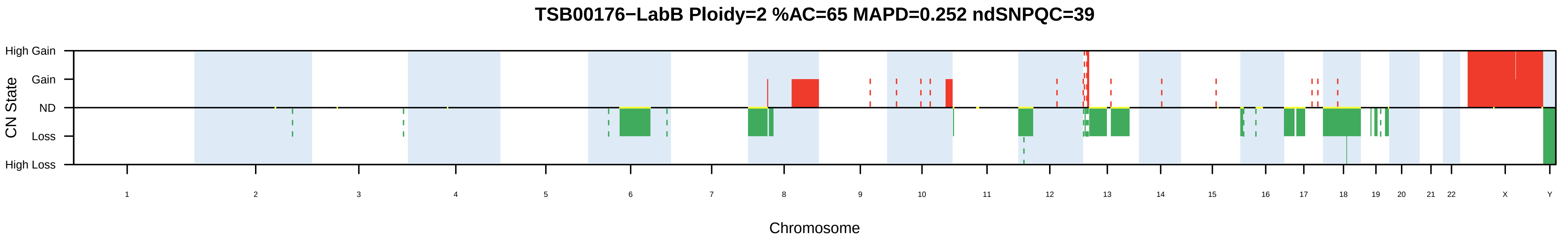
TSB00176−LabB Ploidy=2 %AC=65 MAPD=0.252 ndSNPQC=39
CN State
High Gain
Gain
ND
Loss
High Loss
1 2 3 4 5 6 7 8 9 10 11 12 13 14 15 16 17 18 19 20 21 22 X Y
Chromosome

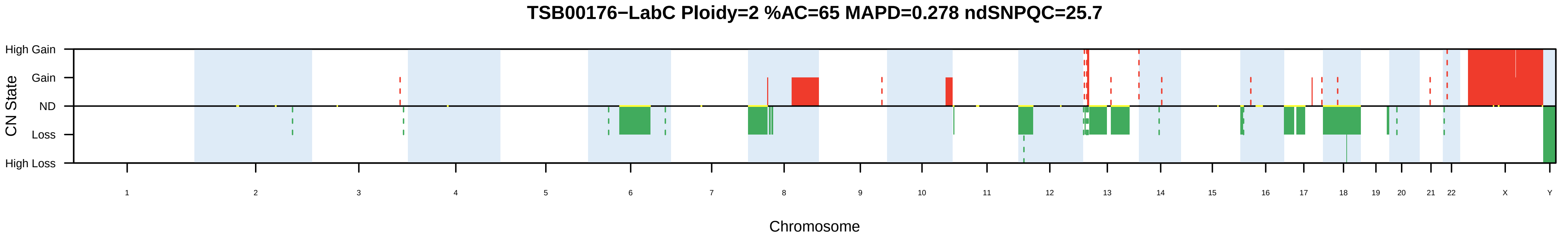
TSB00176−LabC Ploidy=2 %AC=65 MAPD=0.278 ndSNPQC=25.7
CN State
High Gain
Gain
ND
Loss
High Loss
1 2 3 4 5 6 7 8 9 10 11 12 13 14 15 16 17 18 19 20 21 22 X Y
Chromosome

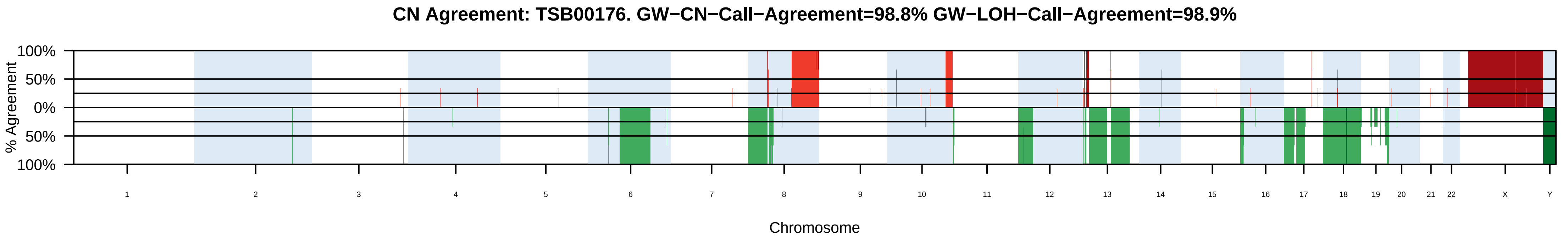
CN Agreement: TSB00176. GW−CN−Call−Agreement=98.8% GW−LOH−Call−Agreement=98.9%
% Agreement
100%
50%
0%
50%
100%
1 2 3 4 5 6 7 8 9 10 11 12 13 14 15 16 17 18 19 20 21 22 X Y
Chromosome

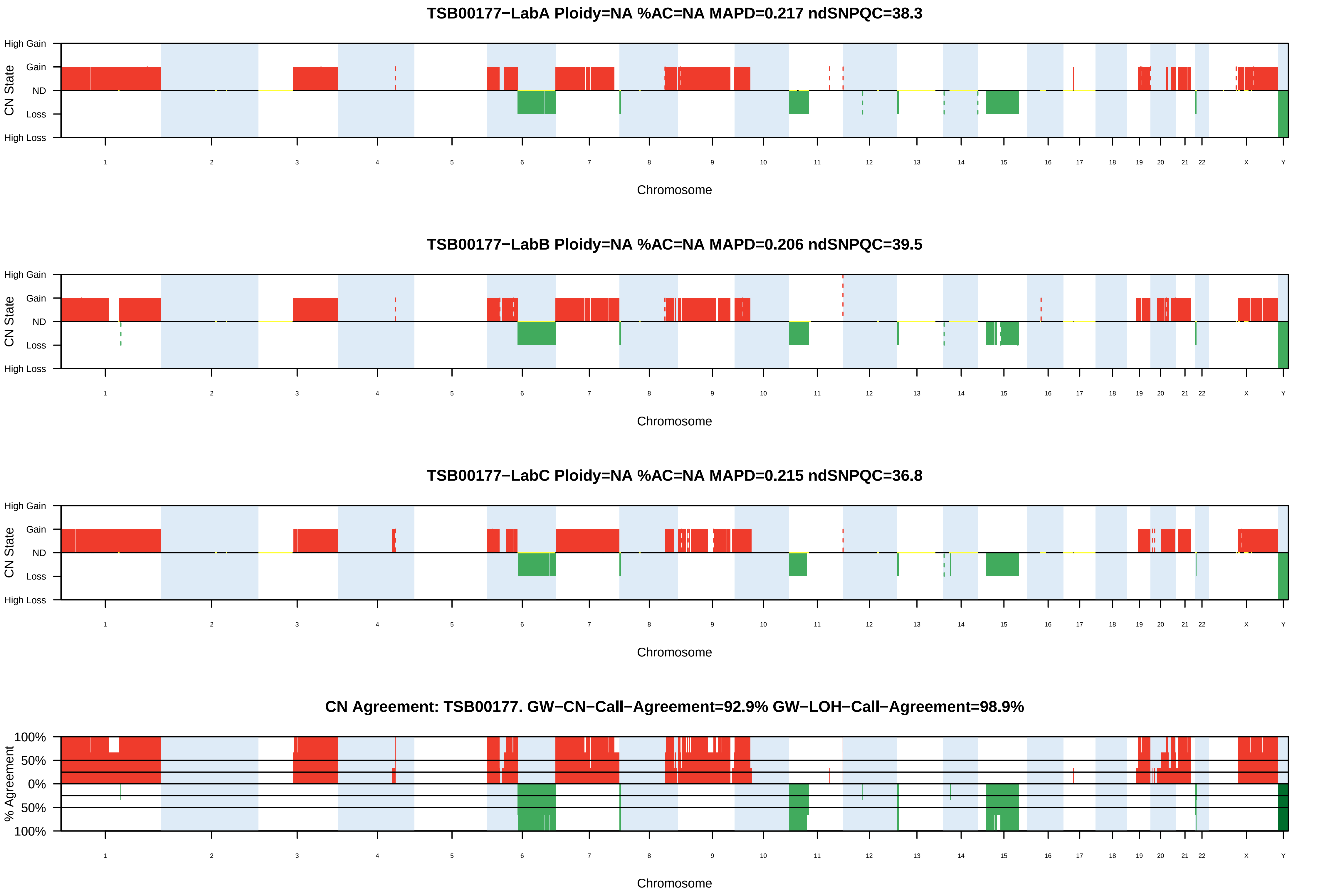
TSB00177−LabA Ploidy=NA %AC=NA MAPD=0.217 ndSNPQC=38.3
TSB00177−LabB Ploidy=NA %AC=NA MAPD=0.206 ndSNPQC=39.5
TSB00177−LabC Ploidy=NA %AC=NA MAPD=0.215 ndSNPQC=36.8
CN Agreement: TSB00177. GW−CN−Call−Agreement=92.9% GW−LOH−Call−Agreement=98.9%
CN State
High Gain
Gain
ND
Loss
High Loss
% Agreement
100%
50%
0%
Chromosome
1
2
3
4
5
6
7
8
9
10
11
12
13
14
15
16
17
18
19
20
21
22
X
Y

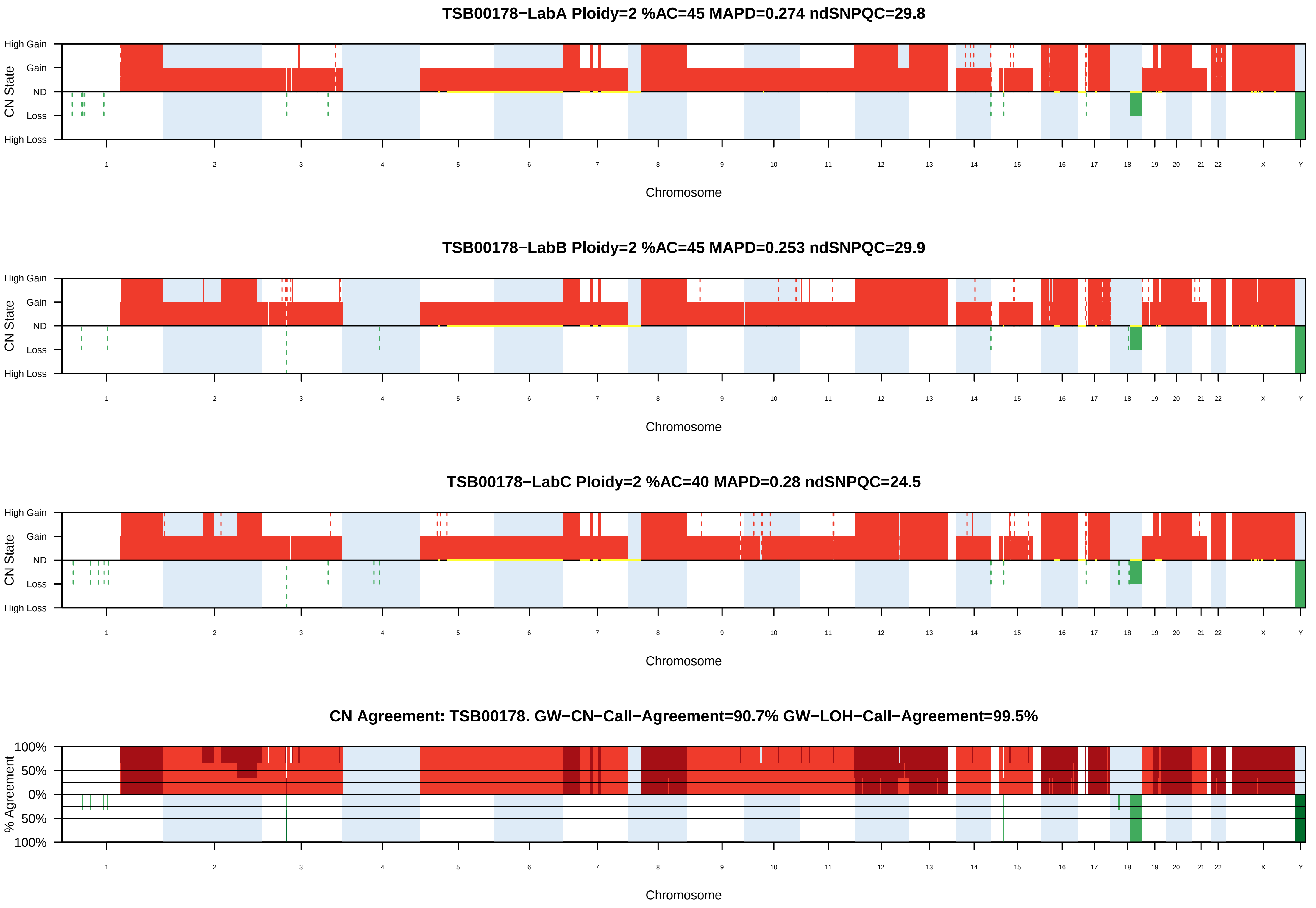
TSB00178−LabA Ploidy=2 %AC=45 MAPD=0.274 ndSNPQC=29.8
CN State
High Gain
Gain
ND
Loss
High Loss
1 2 3 4 5 6 7 8 9 10 11 12 13 14 15 16 17 18 19 20 21 22 X Y
Chromosome
TSB00178−LabB Ploidy=2 %AC=45 MAPD=0.253 ndSNPQC=29.9
CN State
High Gain
Gain
ND
Loss
High Loss
1 2 3 4 5 6 7 8 9 10 11 12 13 14 15 16 17 18 19 20 21 22 X Y
Chromosome
TSB00178−LabC Ploidy=2 %AC=40 MAPD=0.28 ndSNPQC=24.5
CN State
High Gain
Gain
ND
Loss
High Loss
1 2 3 4 5 6 7 8 9 10 11 12 13 14 15 16 17 18 19 20 21 22 X Y
Chromosome
CN Agreement: TSB00178. GW−CN−Call−Agreement=90.7% GW−LOH−Call−Agreement=99.5%
% Agreement
100%
50%
0%
50%
100%
1 2 3 4 5 6 7 8 9 10 11 12 13 14 15 16 17 18 19 20 21 22 X Y
Chromosome

TSB00179−LabA Ploidy=2 %AC=homogeneous MAPD=0.23 ndSNPQC=43.8

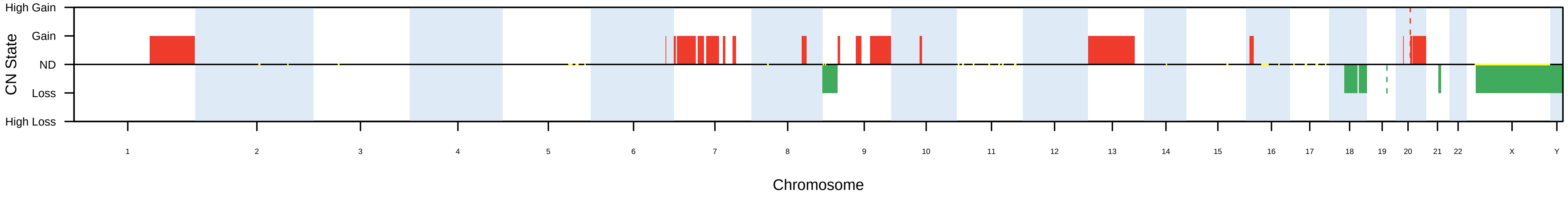

TSB00179−LabB Ploidy=2 %AC=homogeneous MAPD=0.215 ndSNPQC=43.4

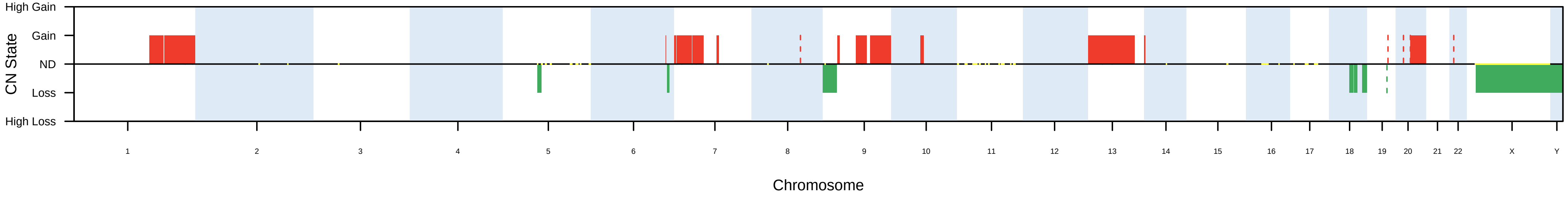

TSB00179−LabC Ploidy=2 %AC=homogeneous MAPD=0.208 ndSNPQC=46.9

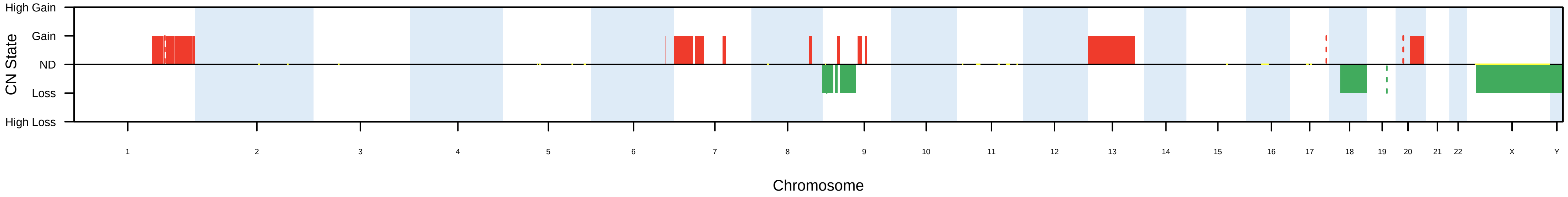

CN Agreement: TSB00179. GW−CN−Call−Agreement=91.6% GW−LOH−Call−Agreement=95.1%

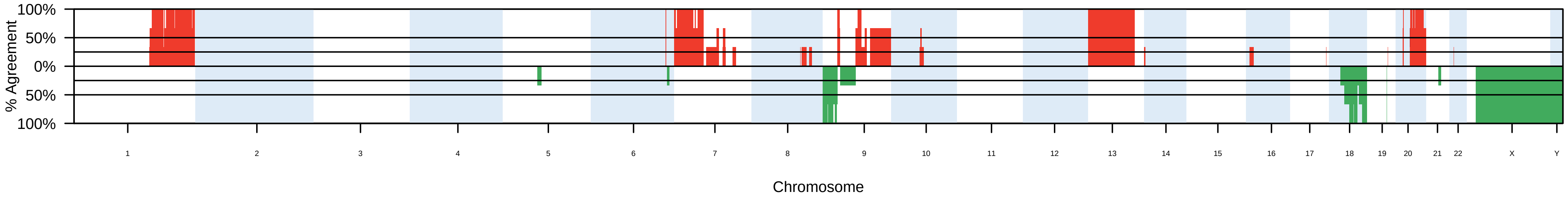

TSB00180−LabA Ploidy=NA %AC=NA MAPD=0.193 ndSNPQC=46.5

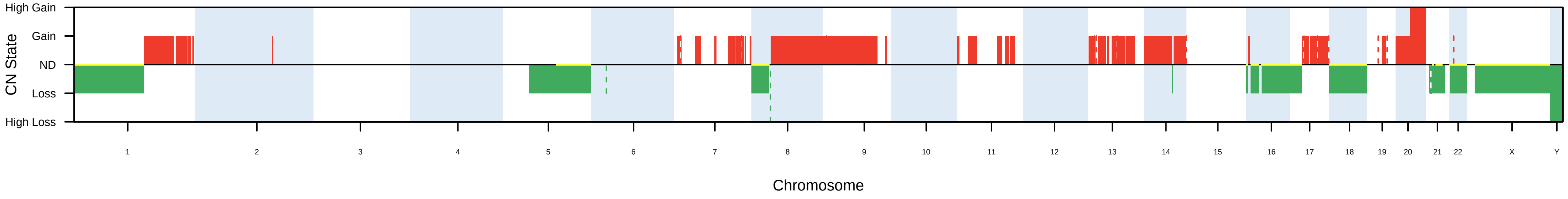

TSB00180−LabB Ploidy=NA %AC=NA MAPD=0.175 ndSNPQC=44.8

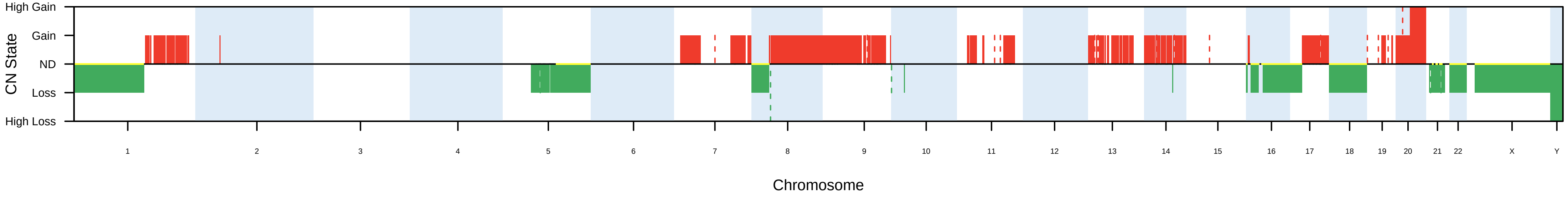

TSB00180−LabC Ploidy=NA %AC=NA MAPD=0.182 ndSNPQC=50.1

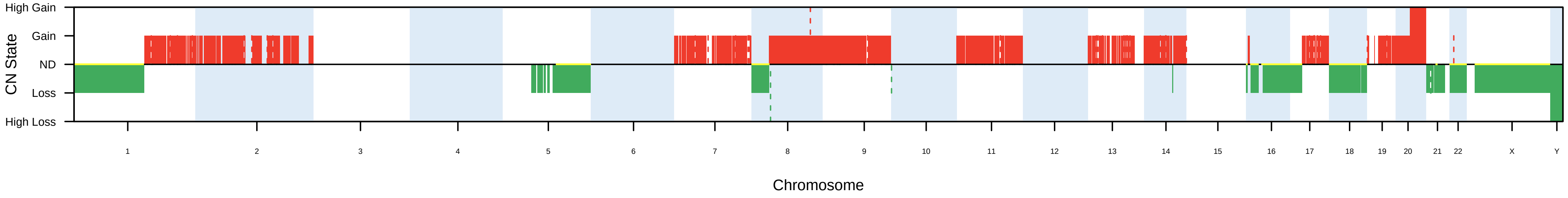

CN Agreement: TSB00180. GW−CN−Call−Agreement=80.8% GW−LOH−Call−Agreement=99.5%

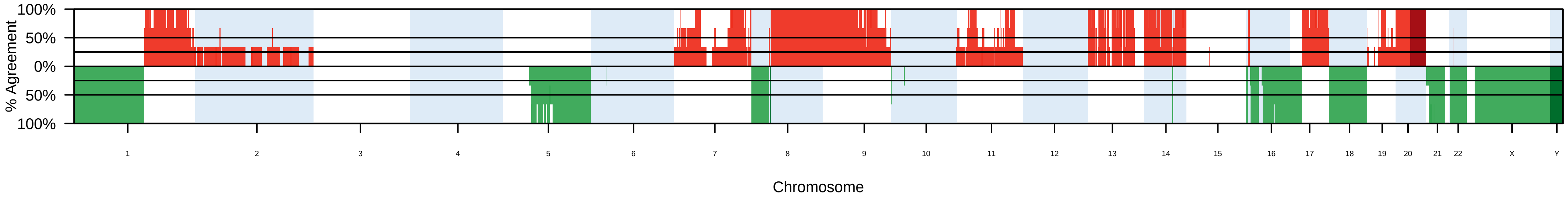

TSB00181−LabA Ploidy=2 %AC=35 MAPD=0.272 ndSNPQC=29.1

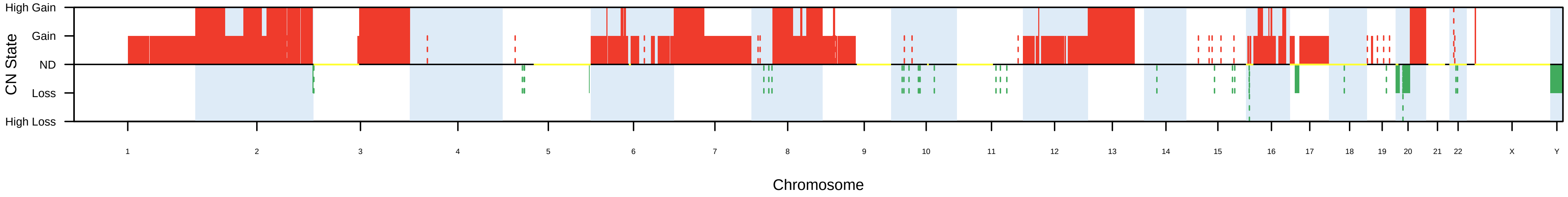

TSB00181−LabB Ploidy=2 %AC=35 MAPD=0.262 ndSNPQC=29.2

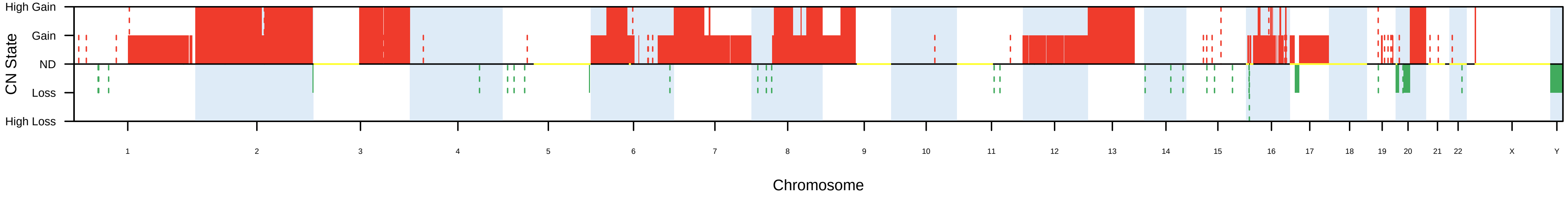

TSB00181−LabC Ploidy=2 %AC=35 MAPD=0.283 ndSNPQC=27.1

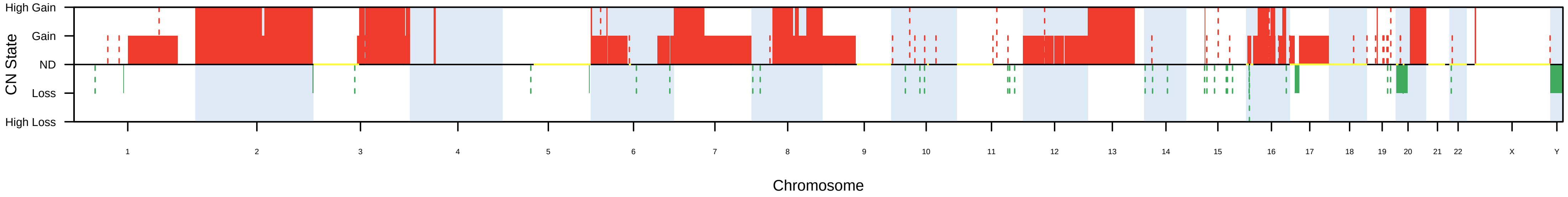

CN Agreement: TSB00181. GW−CN−Call−Agreement=89.6% GW−LOH−Call−Agreement=99.6%

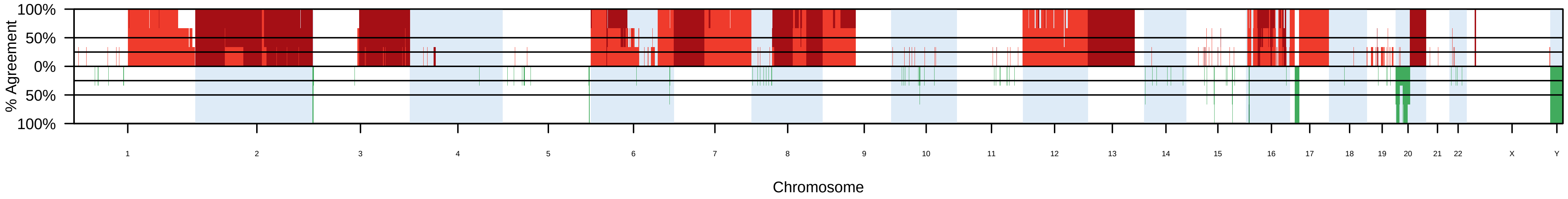

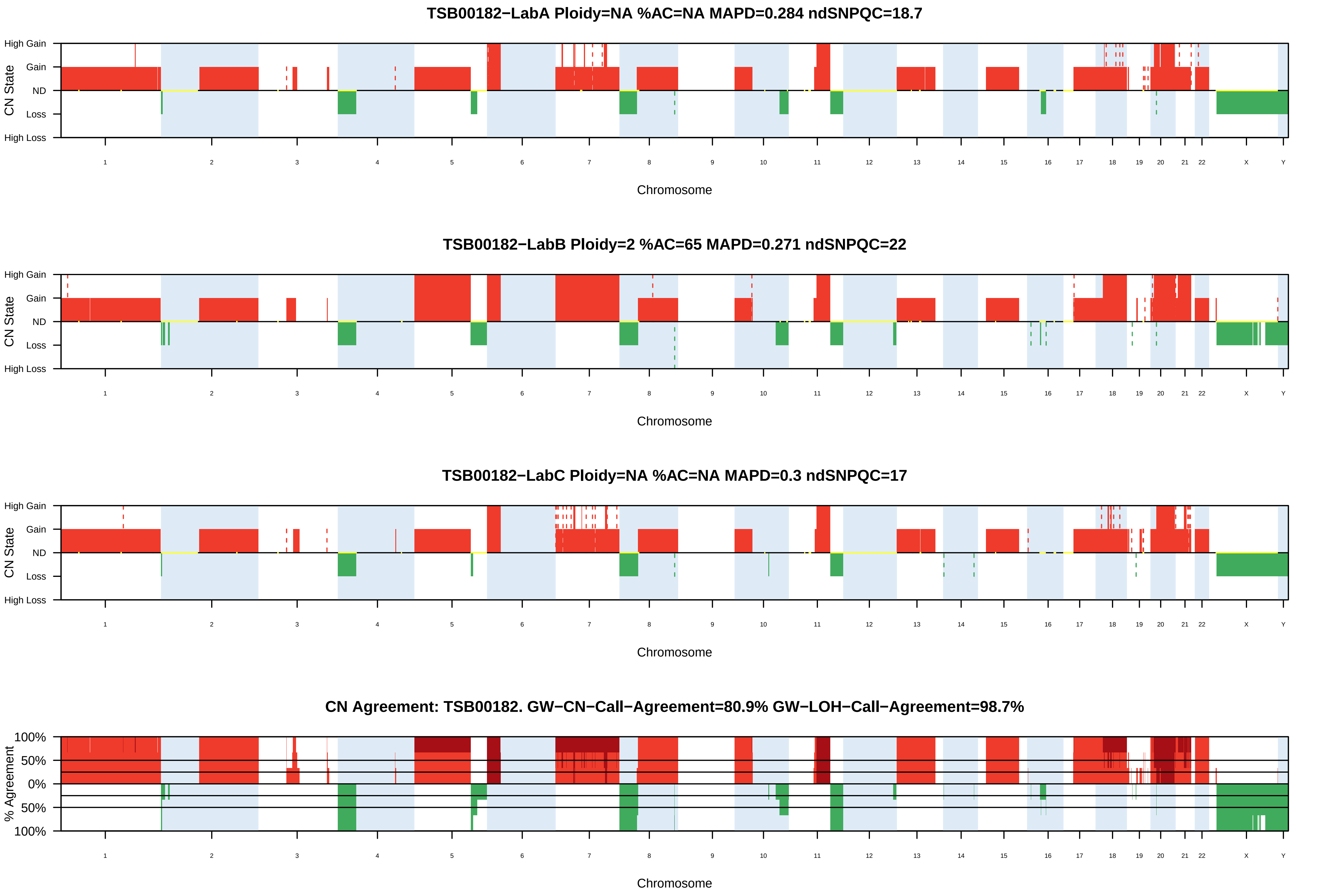

TSB00182−LabA Ploidy=NA %AC=NA MAPD=0.284 ndSNPQC=18.7
TSB00182−LabB Ploidy=2 %AC=65 MAPD=0.271 ndSNPQC=22
TSB00182−LabC Ploidy=NA %AC=NA MAPD=0.3 ndSNPQC=17
CN State
High Gain
Gain
ND
Loss
High Loss
Chromosome
1
2
3
4
5
6
7
8
9
10
11
12
13
14
15
16
17
18
19
20
21
22
X
Y
CN Agreement: TSB00182. GW−CN−Call−Agreement=80.9% GW−LOH−Call−Agreement=98.7%
% Agreement
100%
50%
0%
50%
100%

TSB00183−LabA Ploidy=2 %AC=homogeneous MAPD=0.282 ndSNPQC=28.9

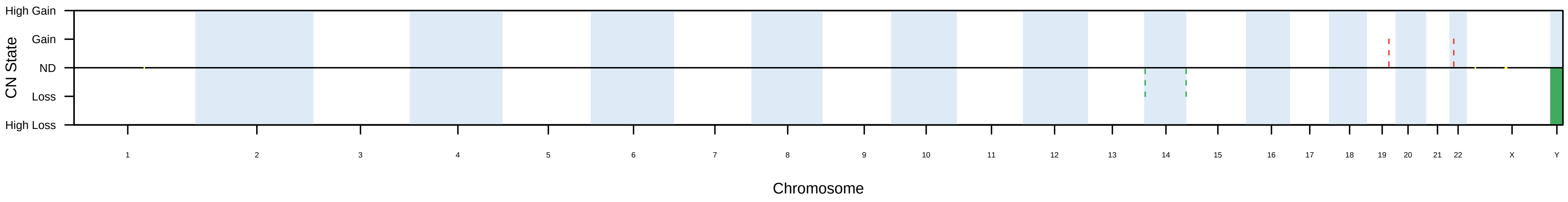

TSB00183−LabB Ploidy=2 %AC=homogeneous MAPD=0.281 ndSNPQC=23.3

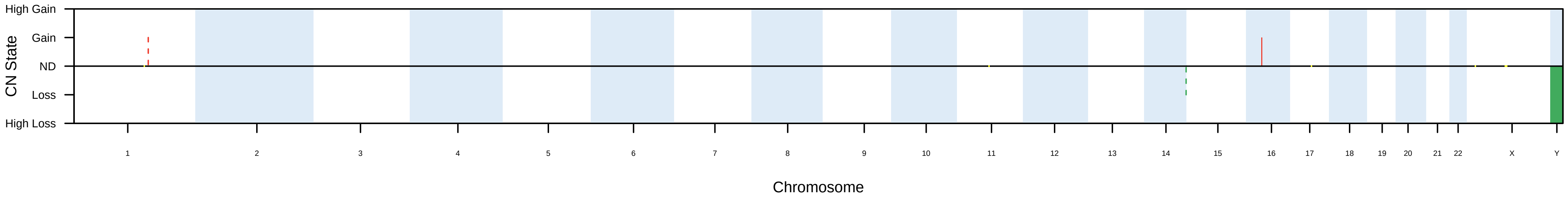

TSB00183−LabC Ploidy=2 %AC=homogeneous MAPD=0.3 ndSNPQC=22.3

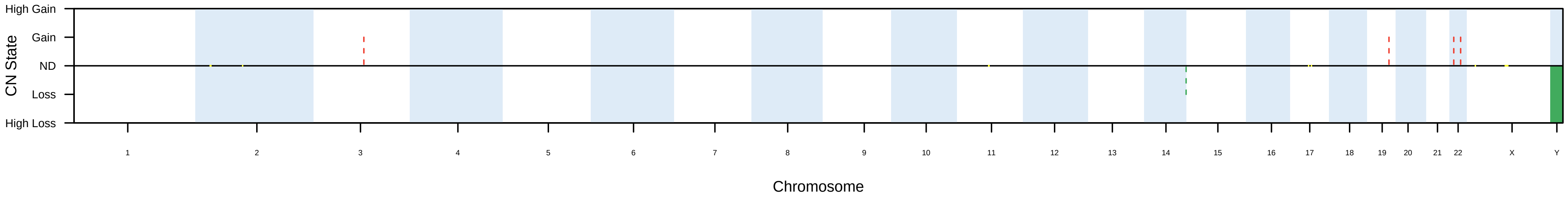

CN Agreement: TSB00183. GW−CN−Call−Agreement=99.9% GW−LOH−Call−Agreement=99.3%

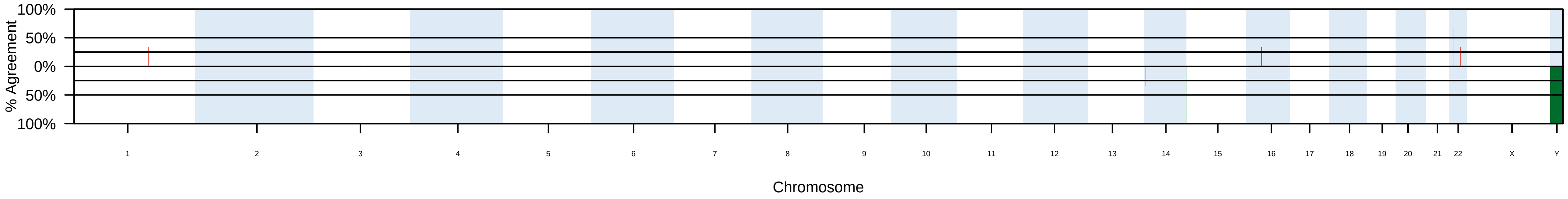

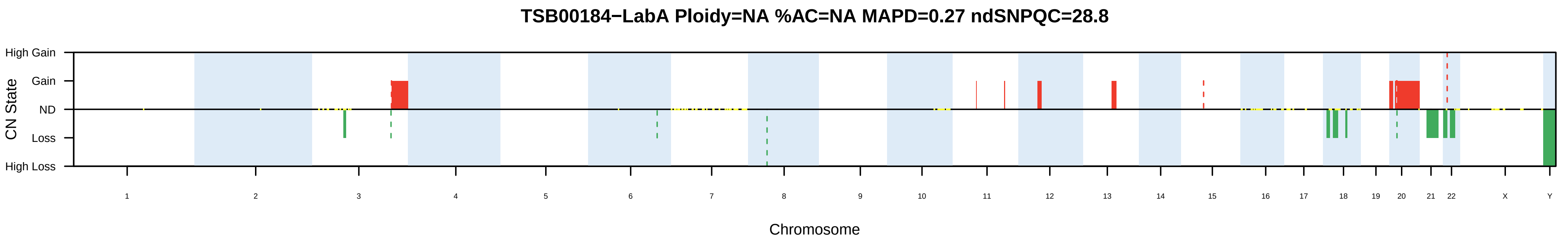
TSB00184−LabA Ploidy=NA %AC=NA MAPD=0.27 ndSNPQC=28.8
High Gain
Gain
ND
Loss
High Loss
CN State
1 2 3 4 5 6 7 8 9 10 11 12 13 14 15 16 17 18 19 20 21 22 X Y
Chromosome

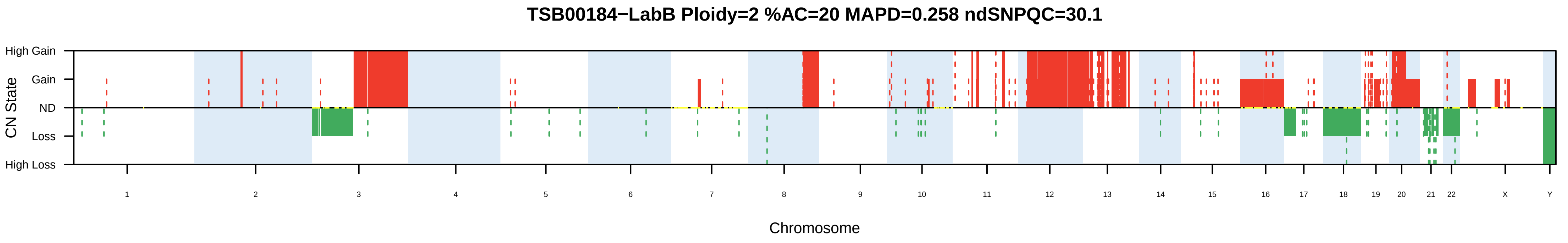
TSB00184−LabB Ploidy=2 %AC=20 MAPD=0.258 ndSNPQC=30.1
High Gain
Gain
ND
Loss
High Loss
CN State
1 2 3 4 5 6 7 8 9 10 11 12 13 14 15 16 17 18 19 20 21 22 X Y
Chromosome

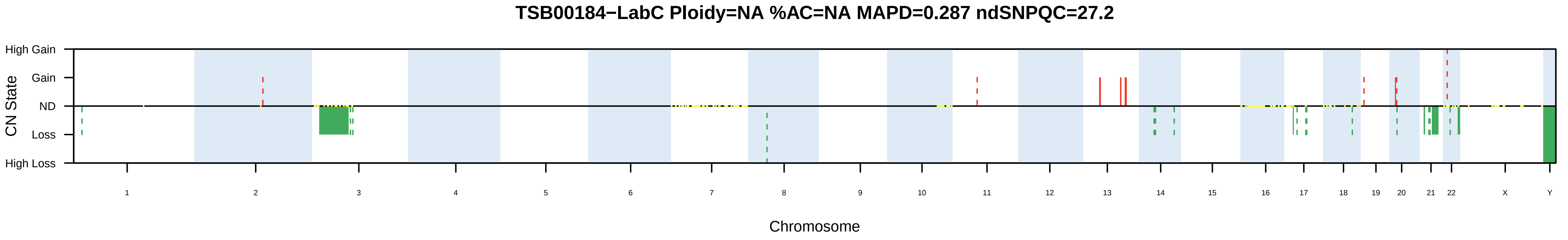
TSB00184−LabC Ploidy=NA %AC=NA MAPD=0.287 ndSNPQC=27.2
High Gain
Gain
ND
Loss
High Loss
CN State
1 2 3 4 5 6 7 8 9 10 11 12 13 14 15 16 17 18 19 20 21 22 X Y
Chromosome

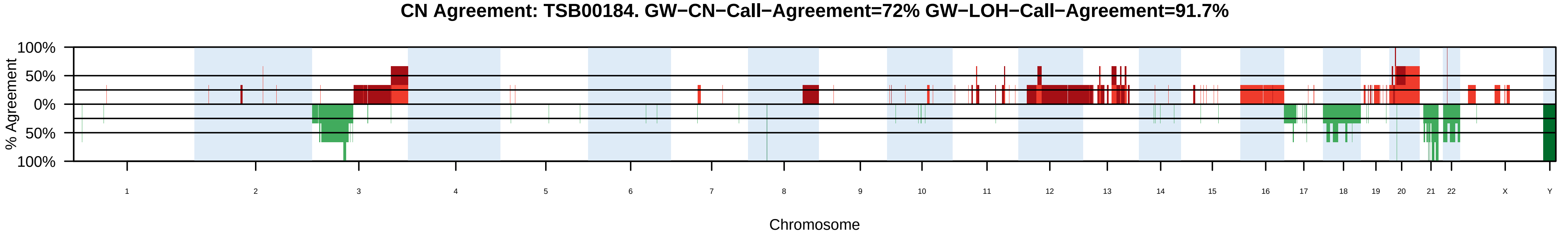
CN Agreement: TSB00184. GW−CN−Call−Agreement=72% GW−LOH−Call−Agreement=91.7%
100%
50%
0%
50%
100%
% Agreement
1 2 3 4 5 6 7 8 9 10 11 12 13 14 15 16 17 18 19 20 21 22 X Y
Chromosome

TSB00186−LabA Ploidy=NA %AC=NA MAPD=0.387 ndSNPQC=14.1

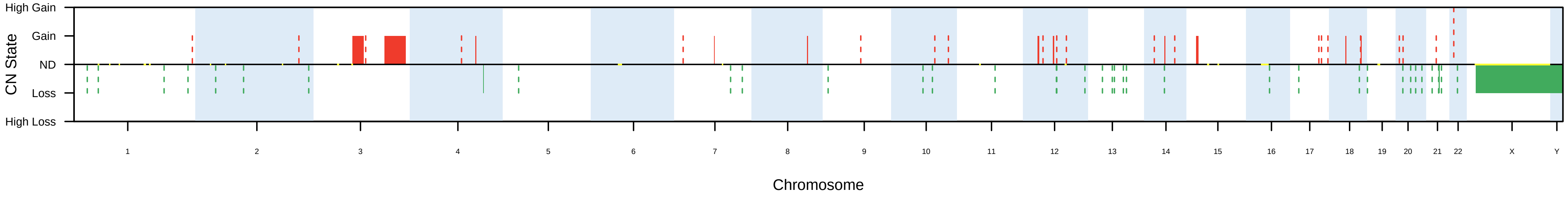

TSB00186−LabB Ploidy=NA %AC=NA MAPD=0.398 ndSNPQC=15

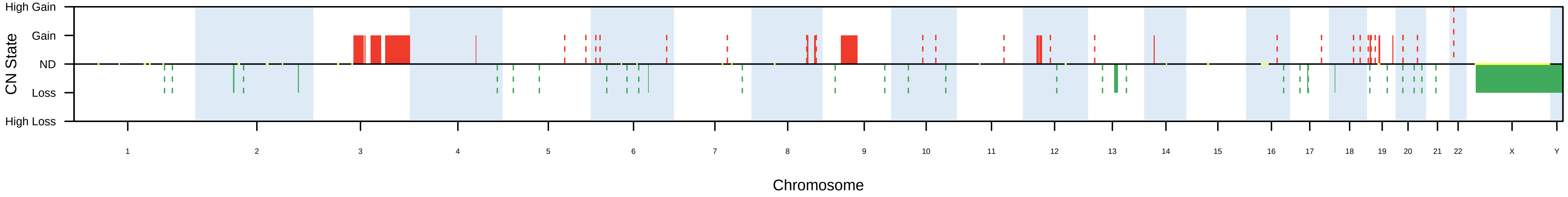

TSB00186−LabC Ploidy=NA %AC=NA MAPD=0.396 ndSNPQC=14.7

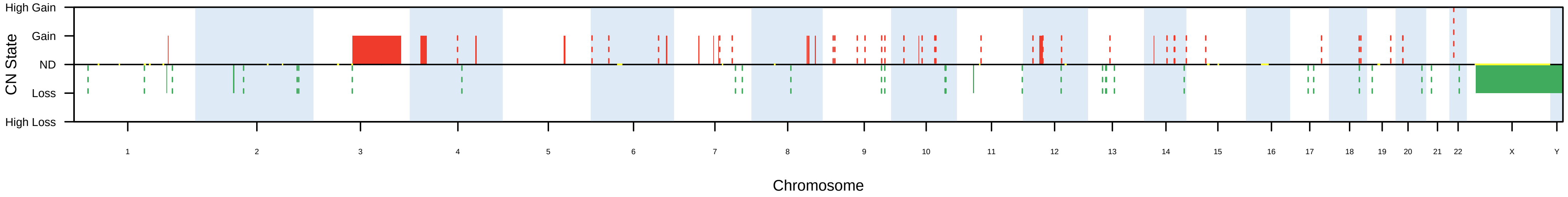

CN Agreement: TSB00186. GW−CN−Call−Agreement=92.6% GW−LOH−Call−Agreement=98.5%

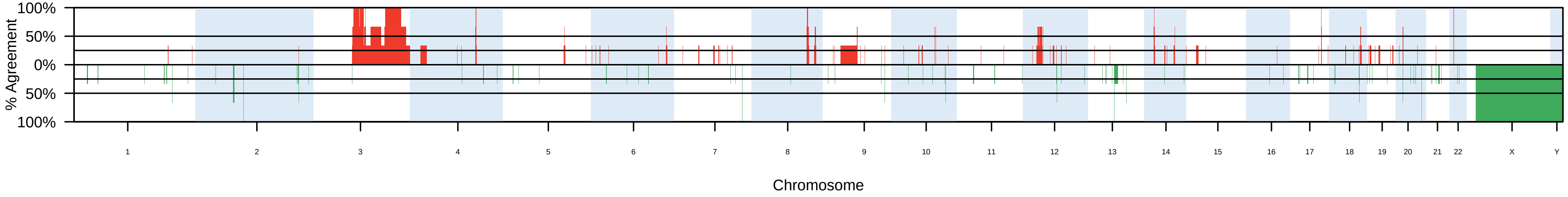

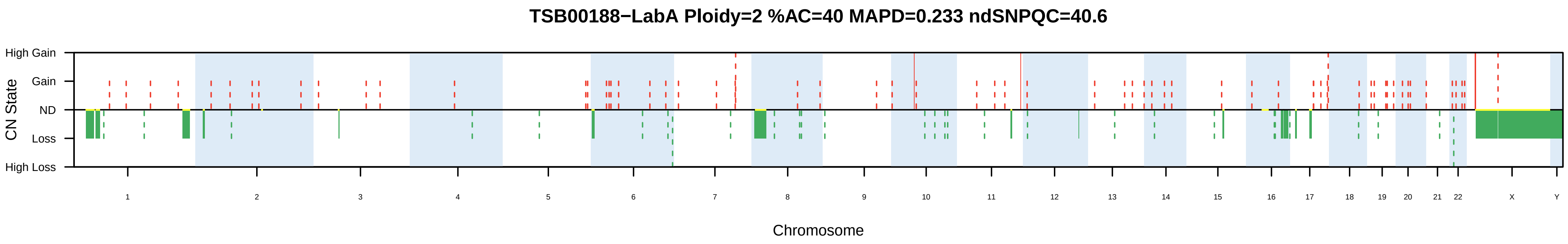
TSB00188−LabA Ploidy=2 %AC=40 MAPD=0.233 ndSNPQC=40.6
High Gain
Gain
ND
Loss
High Loss
CN State
1 2 3 4 5 6 7 8 9 10 11 12 13 14 15 16 17 18 19 20 21 22 X Y
Chromosome

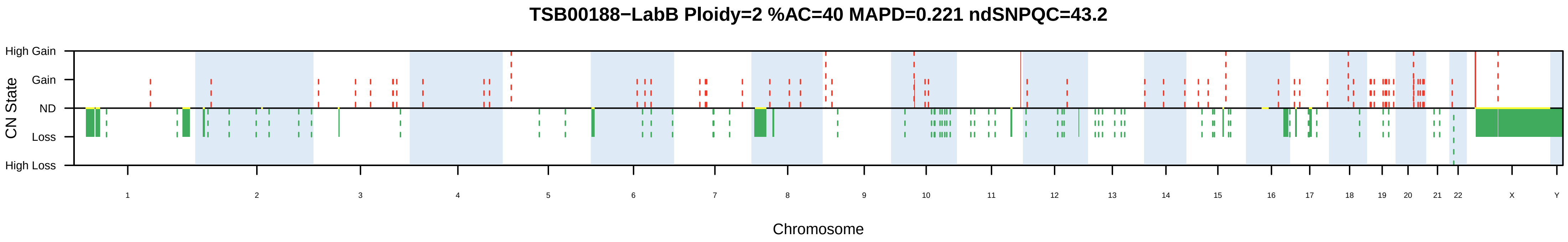
TSB00188−LabB Ploidy=2 %AC=40 MAPD=0.221 ndSNPQC=43.2
High Gain
Gain
ND
Loss
High Loss
CN State
1 2 3 4 5 6 7 8 9 10 11 12 13 14 15 16 17 18 19 20 21 22 X Y
Chromosome

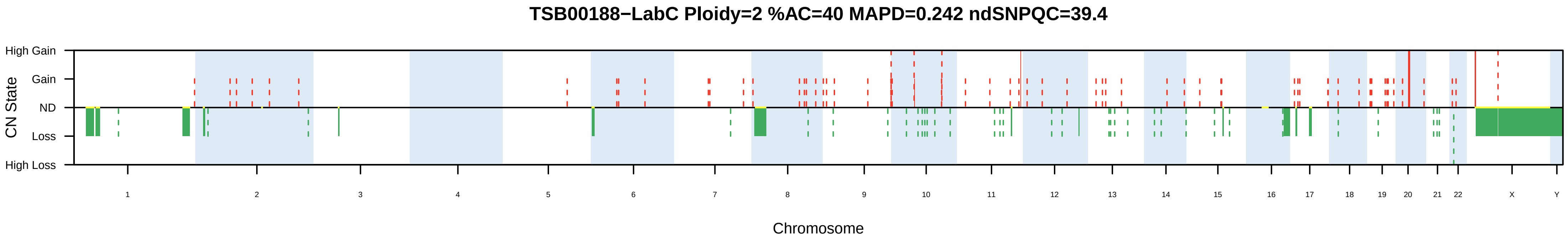
TSB00188−LabC Ploidy=2 %AC=40 MAPD=0.242 ndSNPQC=39.4
High Gain
Gain
ND
Loss
High Loss
CN State
1 2 3 4 5 6 7 8 9 10 11 12 13 14 15 16 17 18 19 20 21 22 X Y
Chromosome

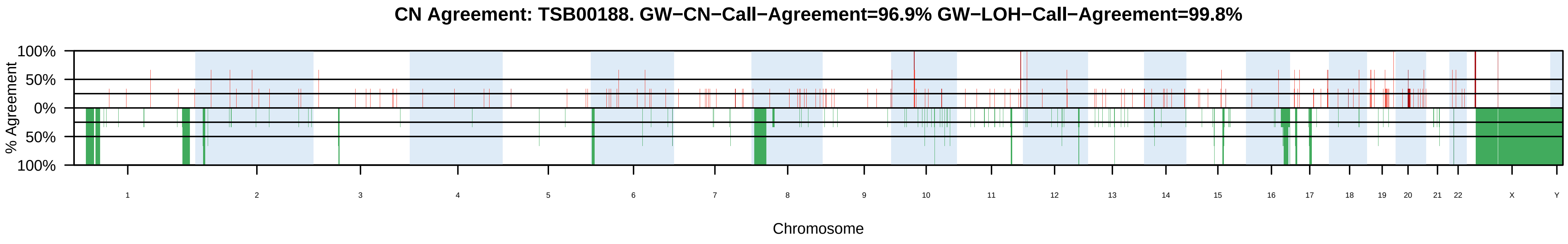
CN Agreement: TSB00188. GW−CN−Call−Agreement=96.9% GW−LOH−Call−Agreement=99.8%
100%
50%
0%
50%
100%
% Agreement
1 2 3 4 5 6 7 8 9 10 11 12 13 14 15 16 17 18 19 20 21 22 X Y
Chromosome

TSB00189−LabA Ploidy=2 %AC=homogeneous MAPD=0.216 ndSNPQC=41.6

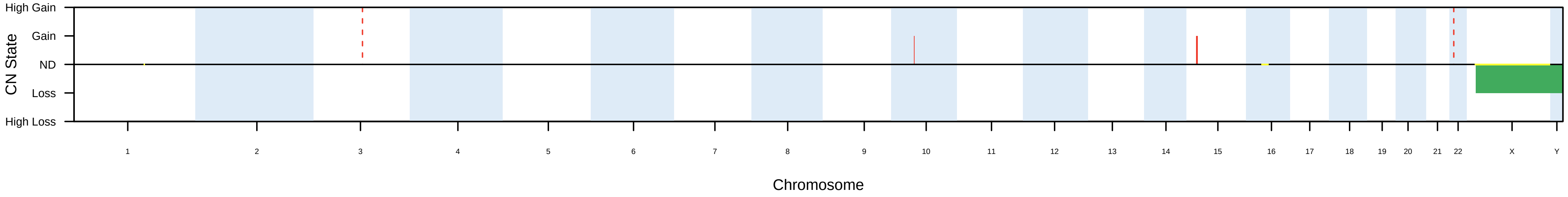

TSB00189−LabB Ploidy=2 %AC=homogeneous MAPD=0.211 ndSNPQC=42.3

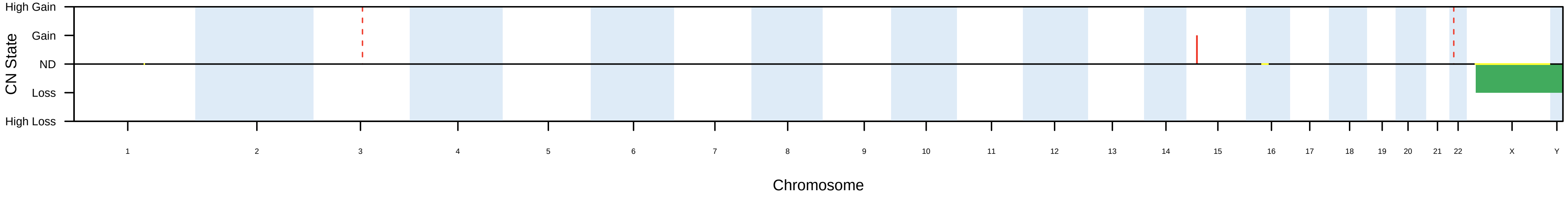

TSB00189−LabC Ploidy=NA %AC=NA MAPD=0.238 ndSNPQC=14.8

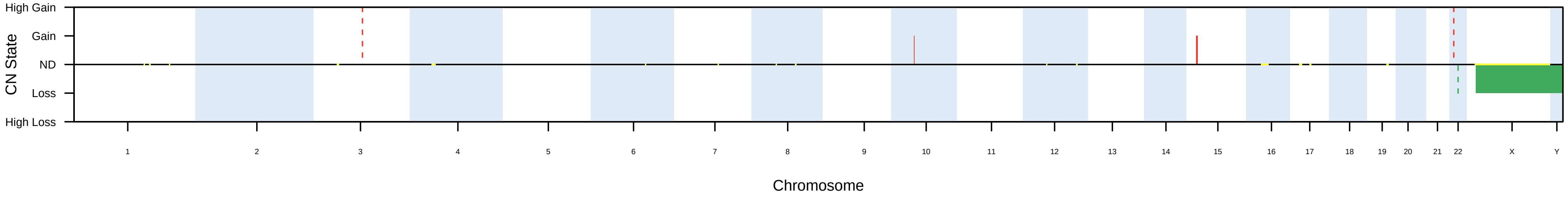

CN Agreement: TSB00189. GW−CN−Call−Agreement=100% GW−LOH−Call−Agreement=98.2%

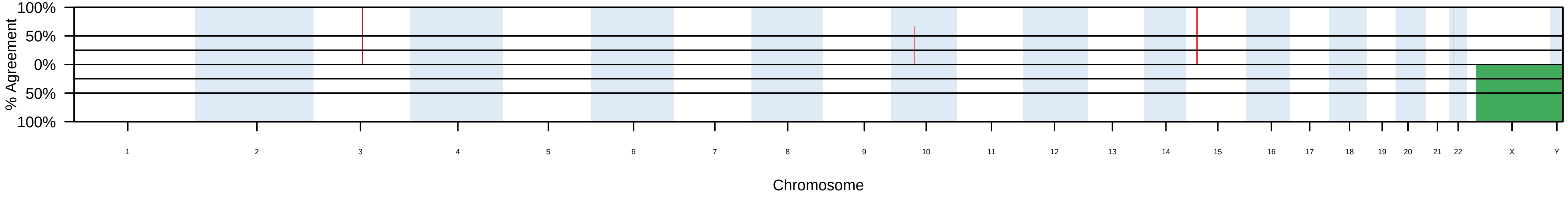

TSB00190−LabA Ploidy=NA %AC=NA MAPD=0.223 ndSNPQC=38.7

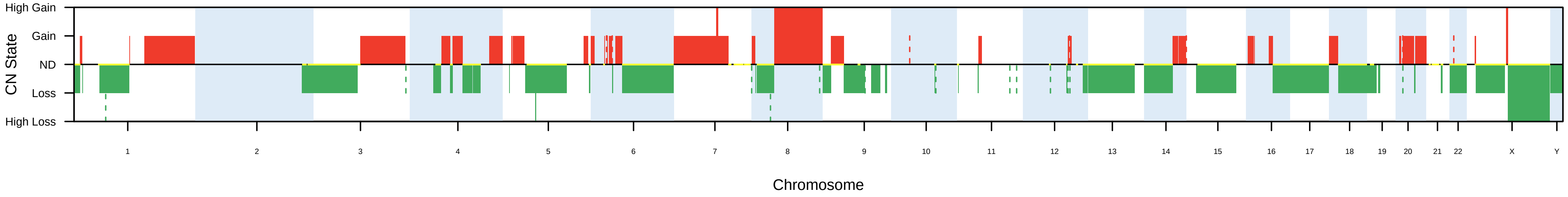

TSB00190−LabB Ploidy=NA %AC=NA MAPD=0.21 ndSNPQC=40.9

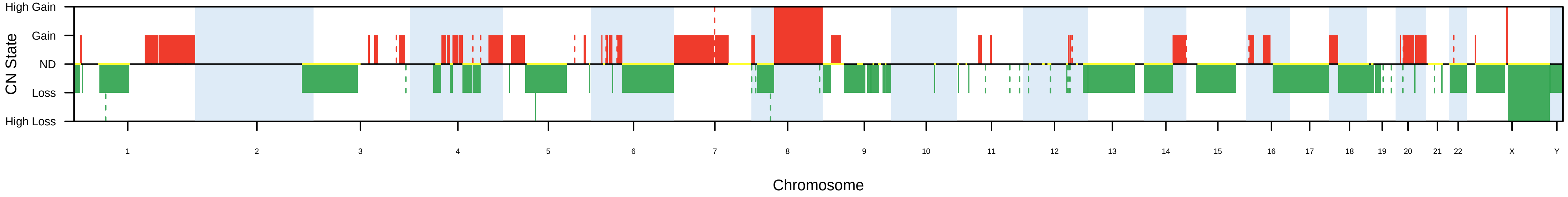

TSB00190−LabC Ploidy=NA %AC=NA MAPD=0.221 ndSNPQC=40.9

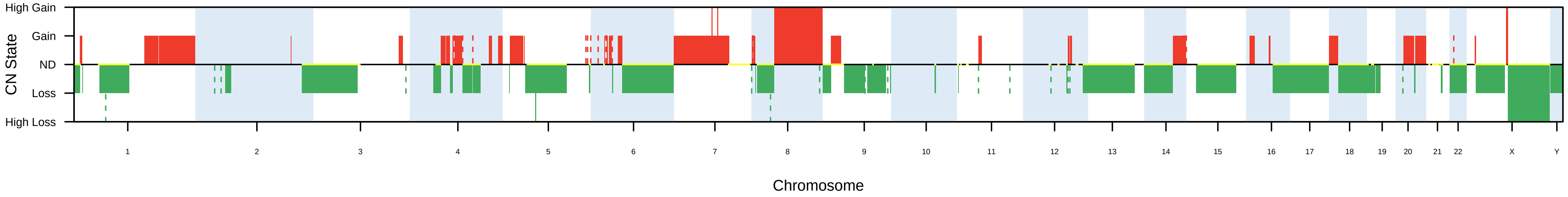

CN Agreement: TSB00190. GW−CN−Call−Agreement=90.7% GW−LOH−Call−Agreement=97.4%

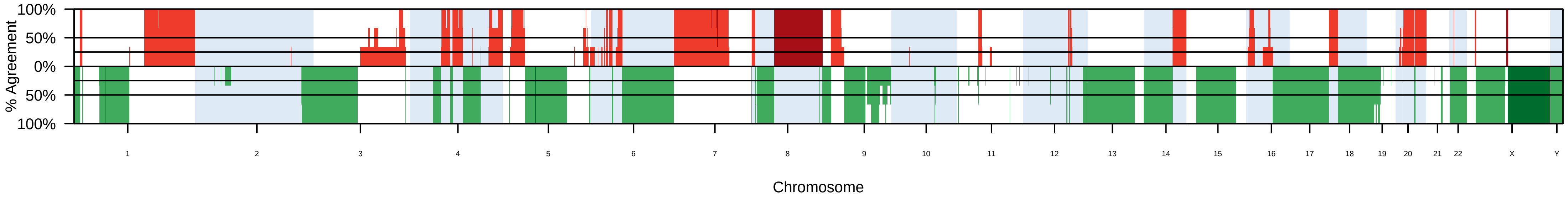

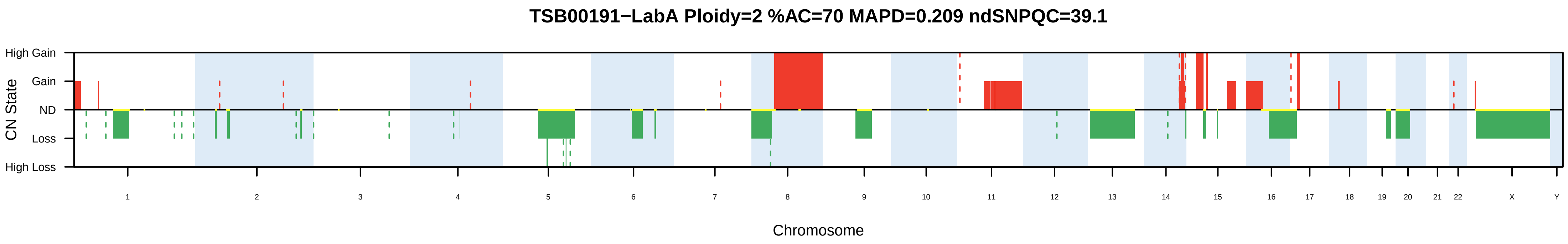
TSB00191−LabA Ploidy=2 %AC=70 MAPD=0.209 ndSNPQC=39.1
High Gain
Gain
ND
Loss
High Loss
CN State
1 2 3 4 5 6 7 8 9 10 11 12 13 14 15 16 17 18 19 20 21 22 X Y
Chromosome

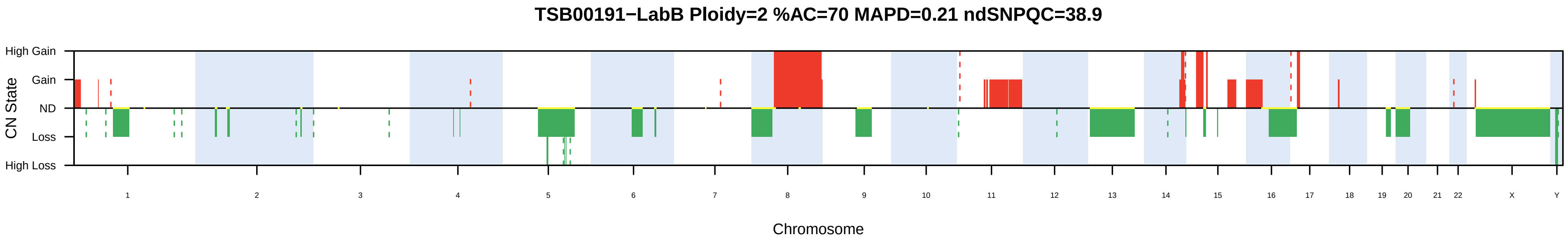
TSB00191−LabB Ploidy=2 %AC=70 MAPD=0.21 ndSNPQC=38.9
High Gain
Gain
ND
Loss
High Loss
CN State
1 2 3 4 5 6 7 8 9 10 11 12 13 14 15 16 17 18 19 20 21 22 X Y
Chromosome

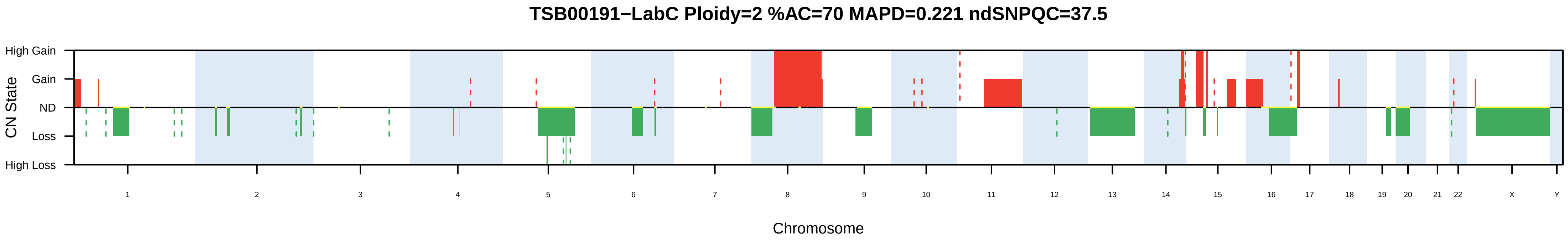
TSB00191−LabC Ploidy=2 %AC=70 MAPD=0.221 ndSNPQC=37.5
High Gain
Gain
ND
Loss
High Loss
CN State
1 2 3 4 5 6 7 8 9 10 11 12 13 14 15 16 17 18 19 20 21 22 X Y
Chromosome

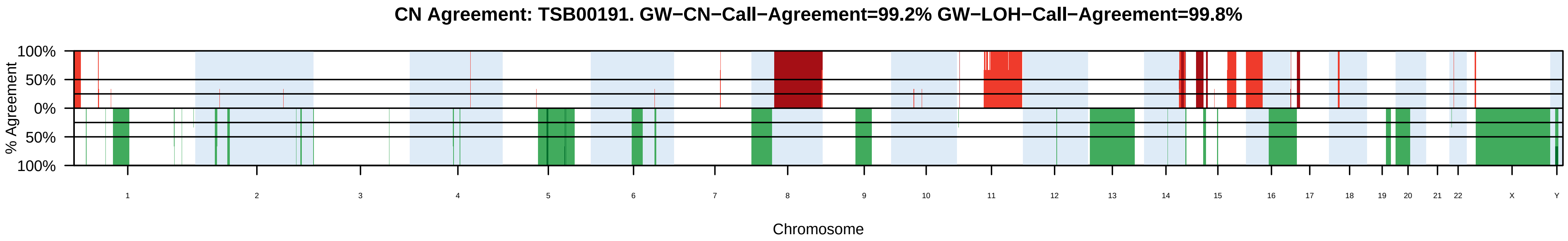
CN Agreement: TSB00191. GW−CN−Call−Agreement=99.2% GW−LOH−Call−Agreement=99.8%
100%
50%
0%
50%
100%
% Agreement
1 2 3 4 5 6 7 8 9 10 11 12 13 14 15 16 17 18 19 20 21 22 X Y
Chromosome

TSB00192−LabA Ploidy=2 %AC=homogeneous MAPD=0.259 ndSNPQC=29

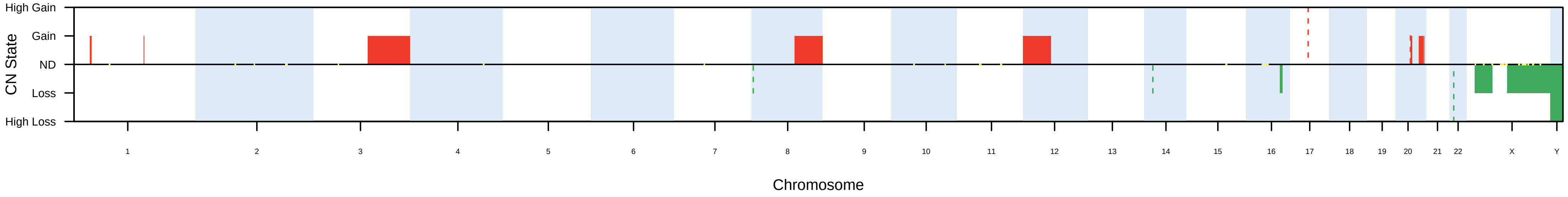

TSB00192−LabB Ploidy=2 %AC=homogeneous MAPD=0.246 ndSNPQC=29.2

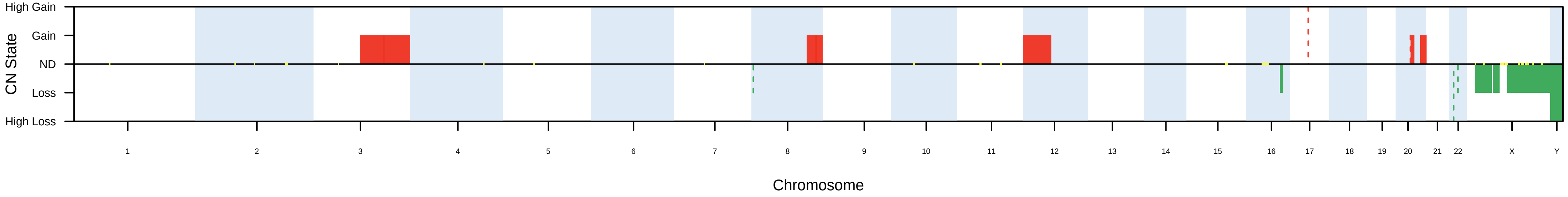

TSB00192−LabC Ploidy=2 %AC=homogeneous MAPD=0.258 ndSNPQC=27

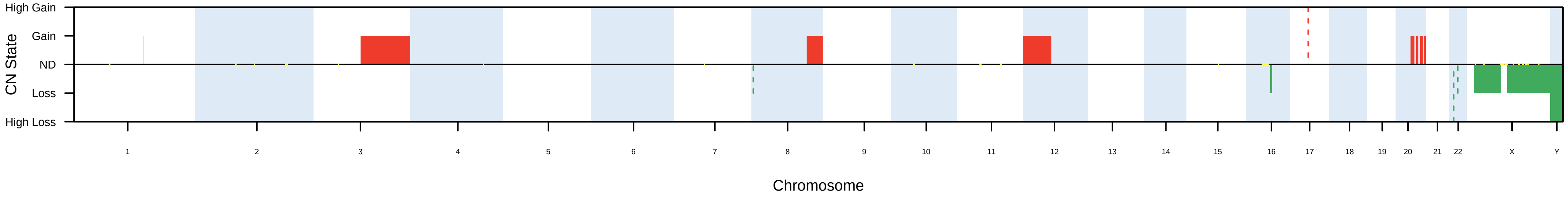

CN Agreement: TSB00192. GW−CN−Call−Agreement=96.6% GW−LOH−Call−Agreement=98.7%

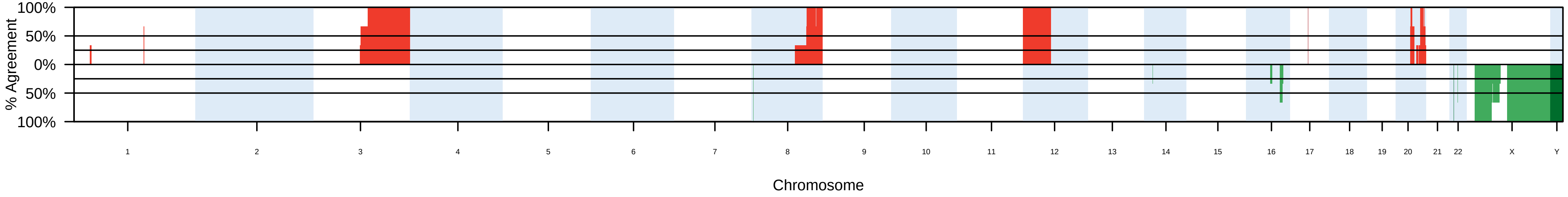

TSB00193−LabA Ploidy=2 %AC=homogeneous MAPD=0.318 ndSNPQC=26.1

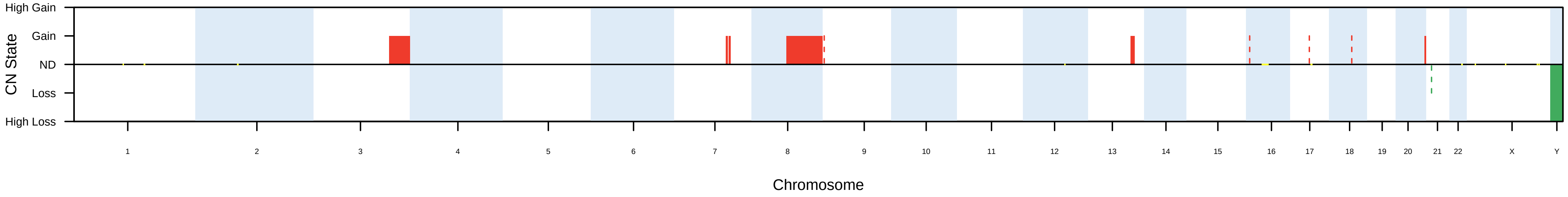

TSB00193−LabB Ploidy=2 %AC=homogeneous MAPD=0.322 ndSNPQC=23.2

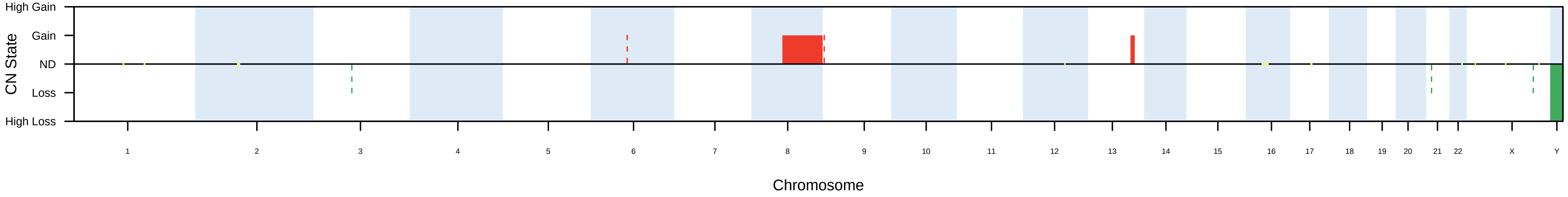

TSB00193−LabC Ploidy=2 %AC=homogeneous MAPD=0.337 ndSNPQC=23.4

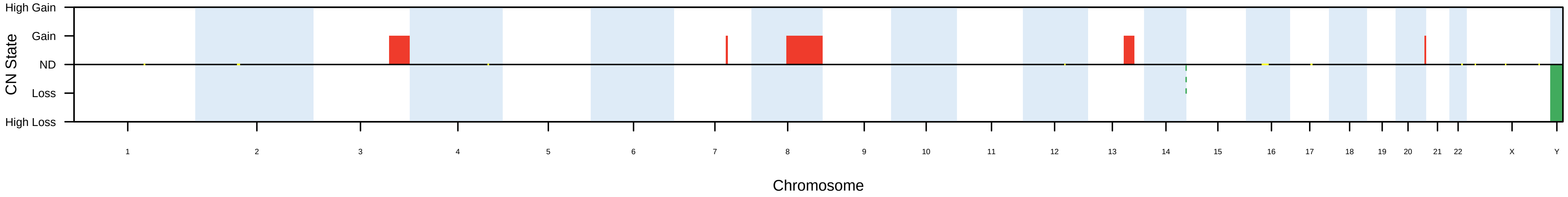

CN Agreement: TSB00193. GW−CN−Call−Agreement=97% GW−LOH−Call−Agreement=99.6%

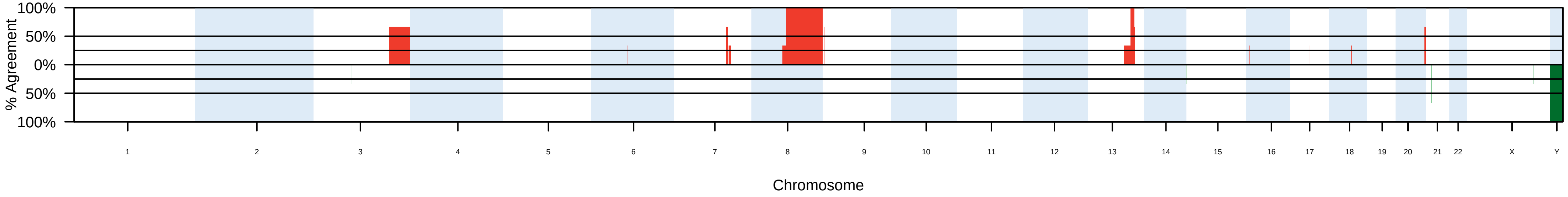

TSB00197−LabA Ploidy=2 %AC=homogeneous MAPD=0.189 ndSNPQC=48.5

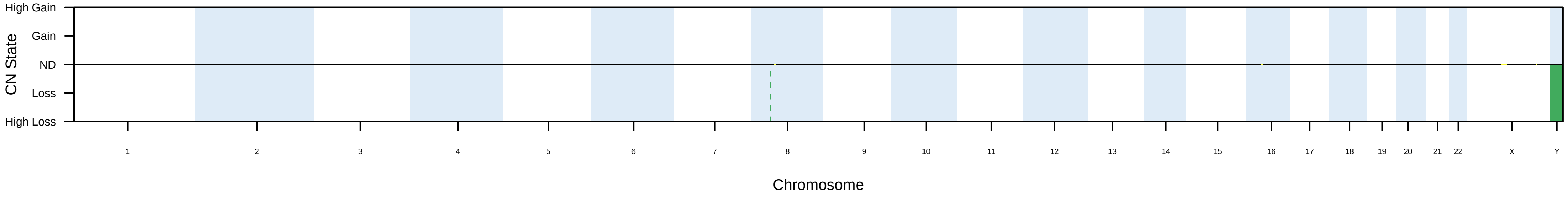

TSB00197−LabB Ploidy=2 %AC=homogeneous MAPD=0.211 ndSNPQC=56.9

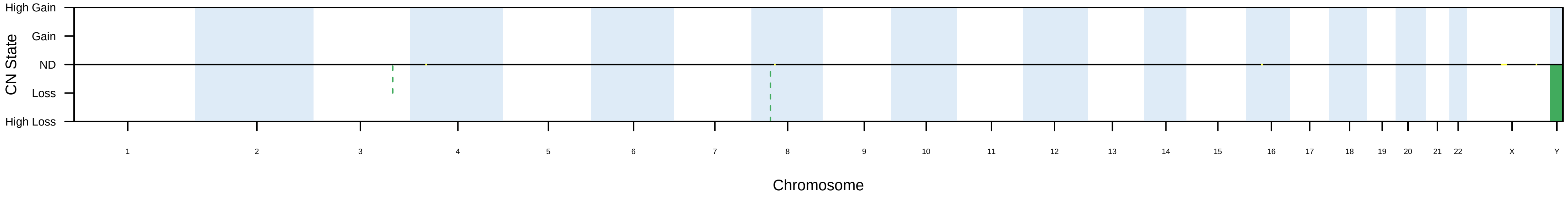

TSB00197−LabC Ploidy=2 %AC=homogeneous MAPD=0.188 ndSNPQC=48.6

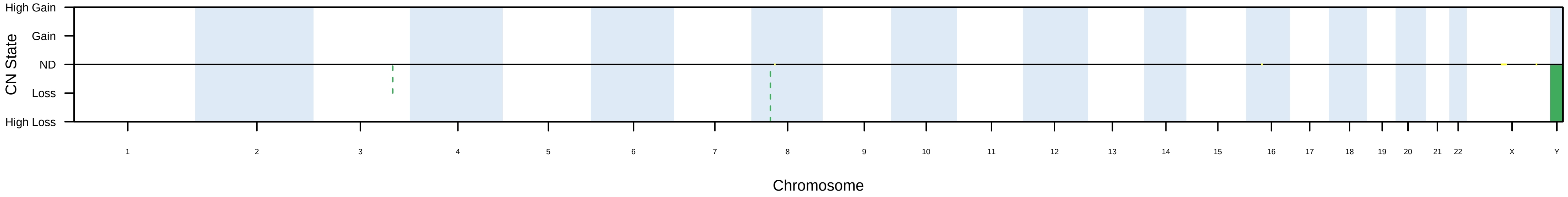

CN Agreement: TSB00197. GW−CN−Call−Agreement=100% GW−LOH−Call−Agreement=99.9%

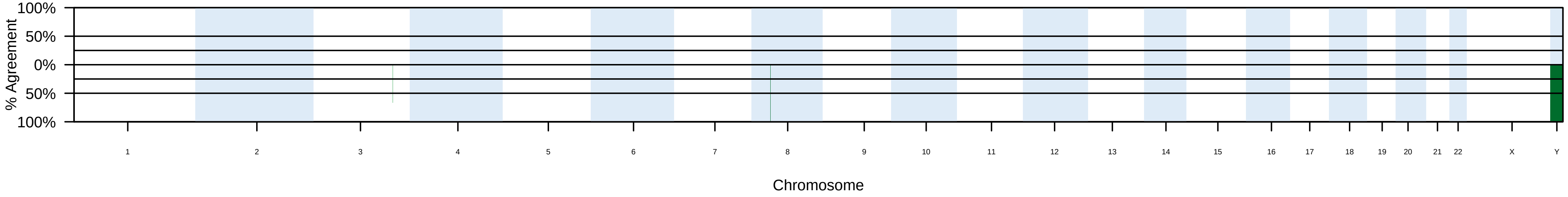

TSB00198−LabA Ploidy=NA %AC=NA MAPD=0.26 ndSNPQC=22.3

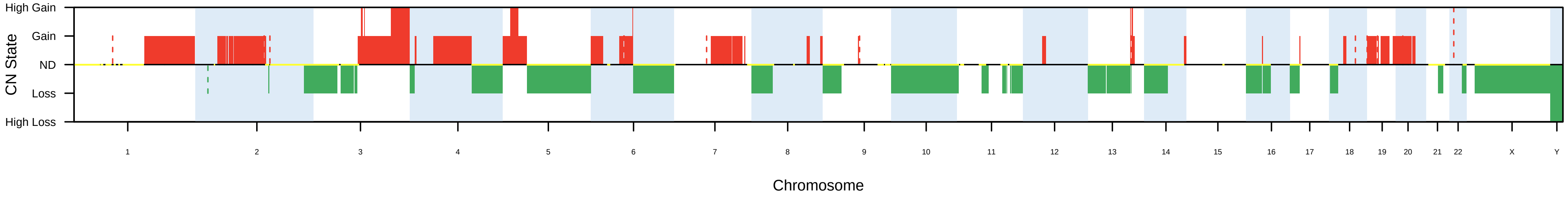

TSB00198−LabB Ploidy=2 %AC=45 MAPD=0.222 ndSNPQC=25

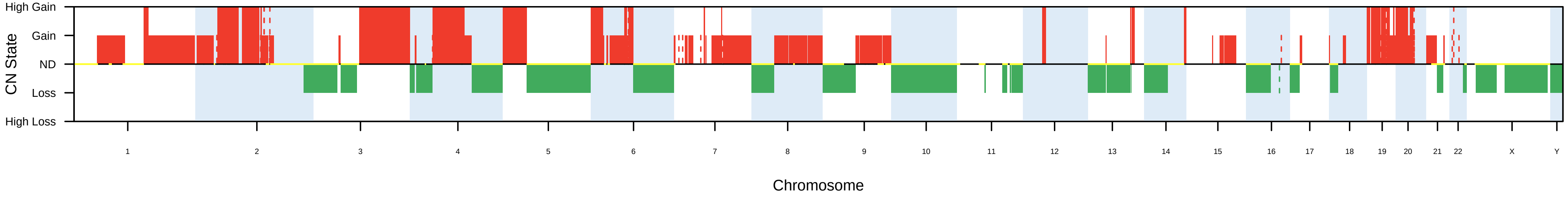

TSB00198−LabC Ploidy=NA %AC=NA MAPD=0.261 ndSNPQC=15.3

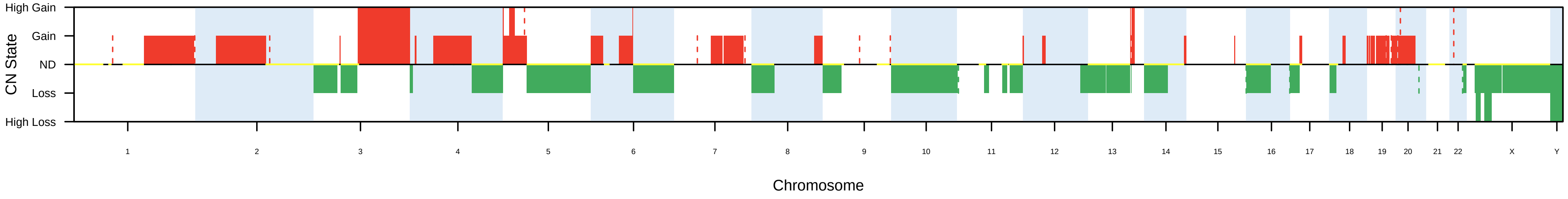

CN Agreement: TSB00198. GW−CN−Call−Agreement=65.7% GW−LOH−Call−Agreement=96.6%

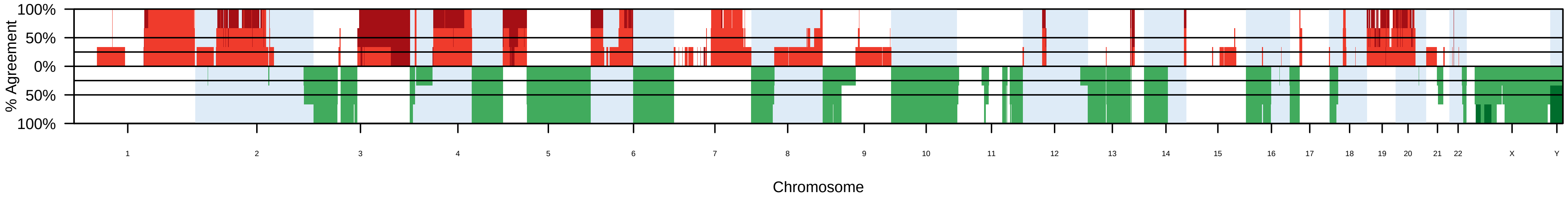

TSB00199−LabA Ploidy=2 %AC=70 MAPD=0.258 ndSNPQC=24.9

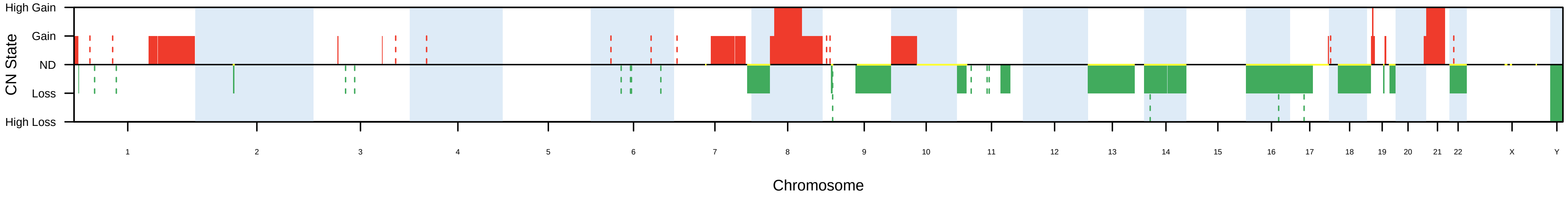

TSB00199−LabB Ploidy=2 %AC=70 MAPD=0.232 ndSNPQC=34.4

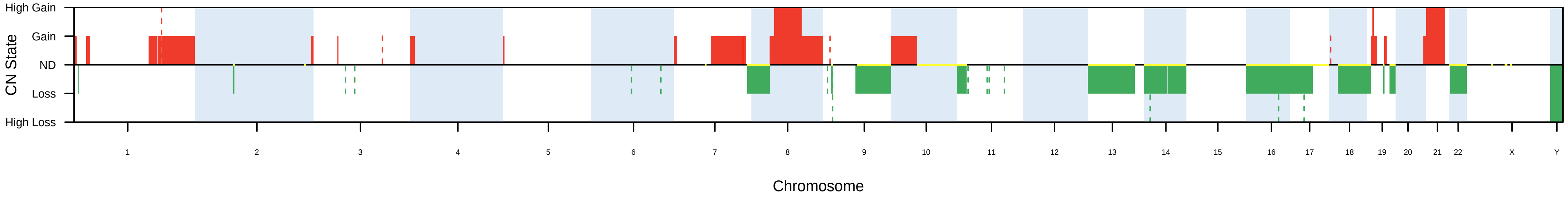

TSB00199−LabC Ploidy=2 %AC=70 MAPD=0.229 ndSNPQC=33.7

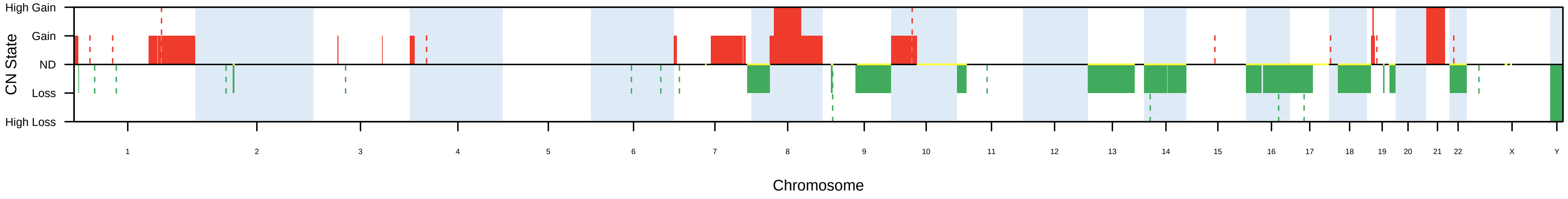

CN Agreement: TSB00199. GW−CN−Call−Agreement=96.7% GW−LOH−Call−Agreement=99.6%

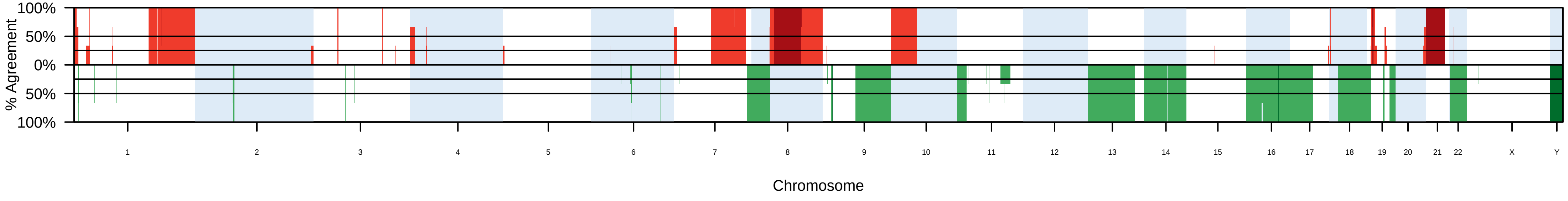

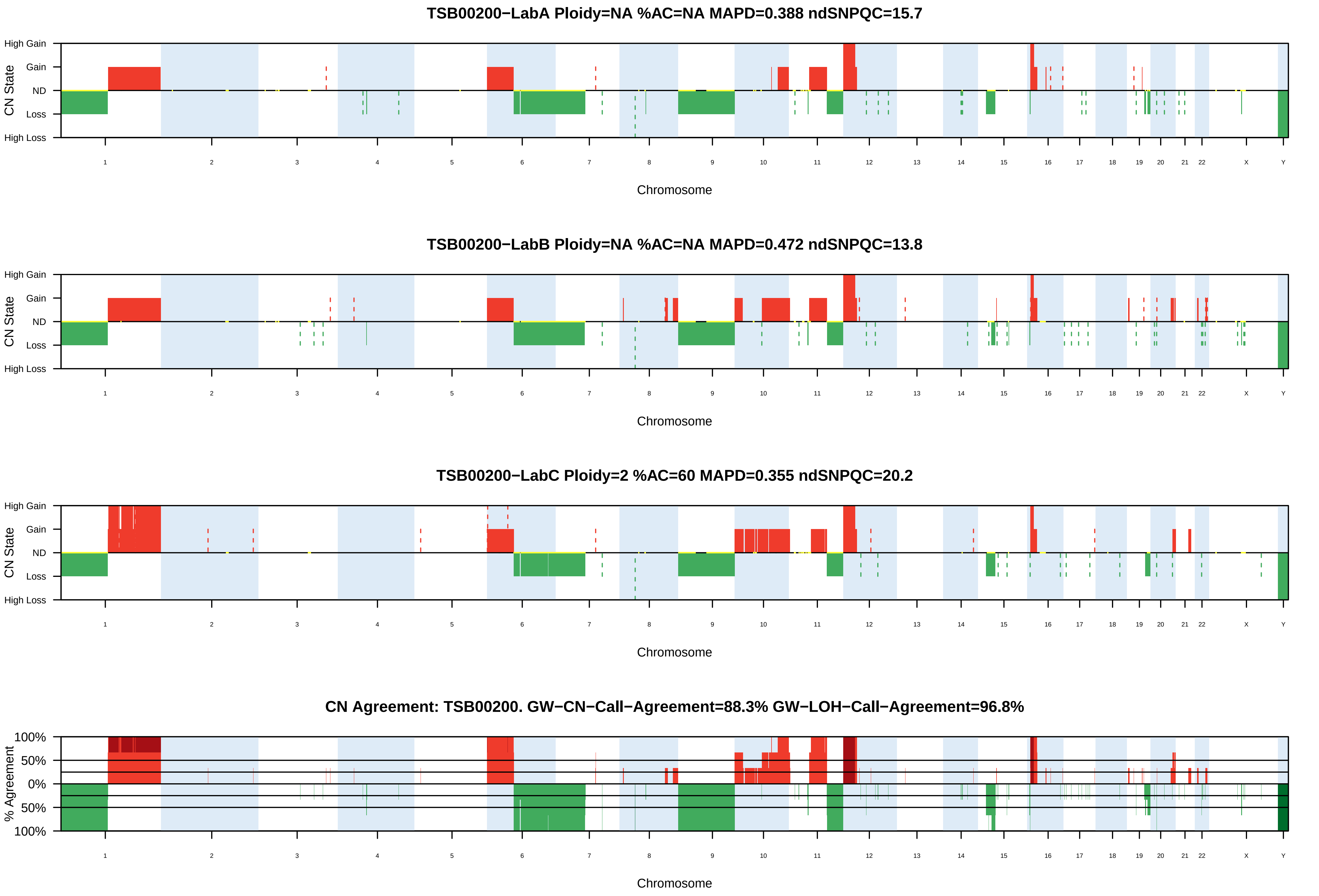
TSB00200−LabA Ploidy=NA %AC=NA MAPD=0.388 ndSNPQC=15.7
High Gain
Gain
ND
Loss
High Loss
CN State
1 2 3 4 5 6 7 8 9 10 11 12 13 14 15 16 17 18 19 20 21 22 X Y
Chromosome
TSB00200−LabB Ploidy=NA %AC=NA MAPD=0.472 ndSNPQC=13.8
High Gain
Gain
ND
Loss
High Loss
CN State
1 2 3 4 5 6 7 8 9 10 11 12 13 14 15 16 17 18 19 20 21 22 X Y
Chromosome
TSB00200−LabC Ploidy=2 %AC=60 MAPD=0.355 ndSNPQC=20.2
High Gain
Gain
ND
Loss
High Loss
CN State
1 2 3 4 5 6 7 8 9 10 11 12 13 14 15 16 17 18 19 20 21 22 X Y
Chromosome
CN Agreement: TSB00200. GW−CN−Call−Agreement=88.3% GW−LOH−Call−Agreement=96.8%
100%
50%
0%
50%
100%
% Agreement
1 2 3 4 5 6 7 8 9 10 11 12 13 14 15 16 17 18 19 20 21 22 X Y
Chromosome

TSB00201−LabA Ploidy=2 %AC=45 MAPD=0.262 ndSNPQC=23.6

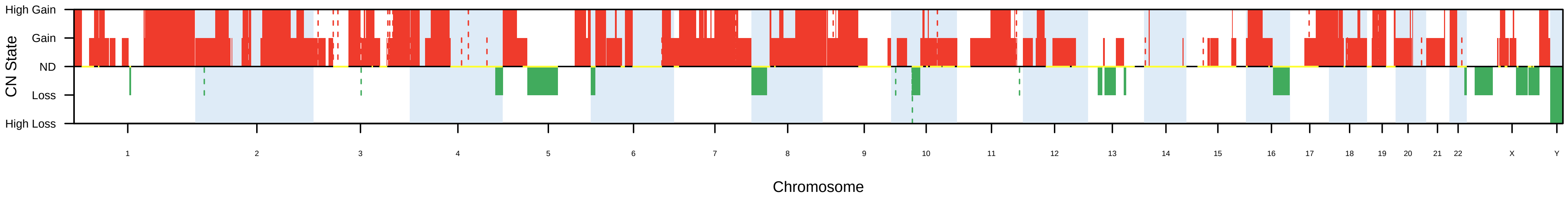

TSB00201−LabB Ploidy=2 %AC=50 MAPD=0.244 ndSNPQC=30

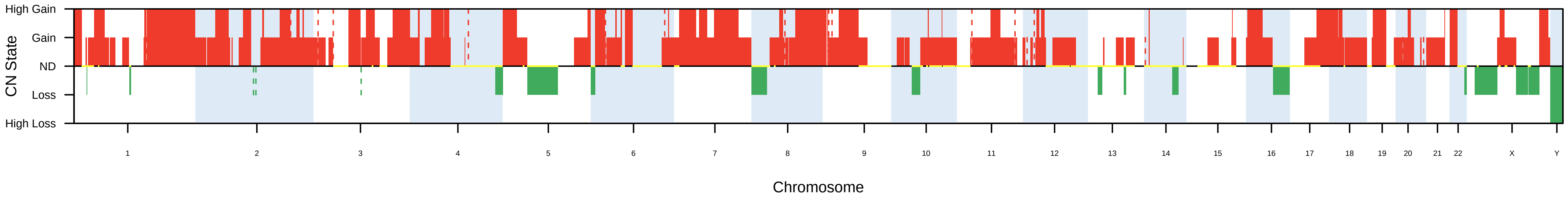

TSB00201−LabC Ploidy=2 %AC=50 MAPD=0.258 ndSNPQC=27.7

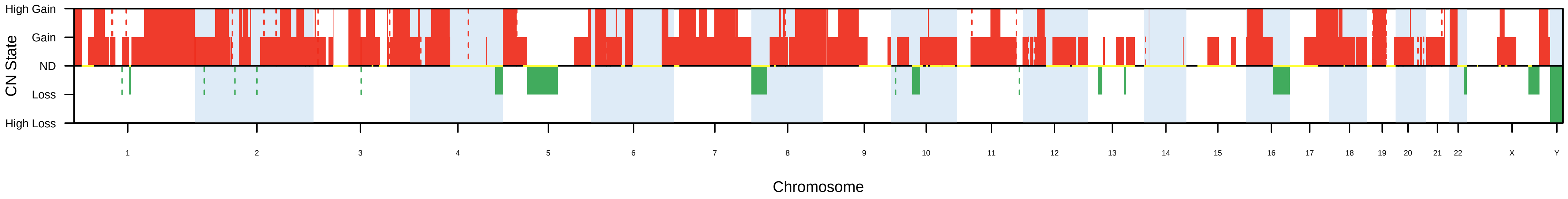

CN Agreement: TSB00201. GW−CN−Call−Agreement=84.5% GW−LOH−Call−Agreement=98.4%

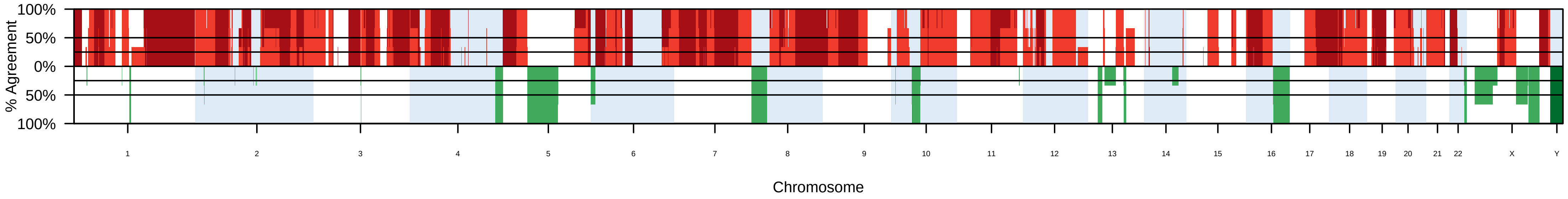

TSB00202−LabA Ploidy=NA %AC=NA MAPD=0.189 ndSNPQC=38.8

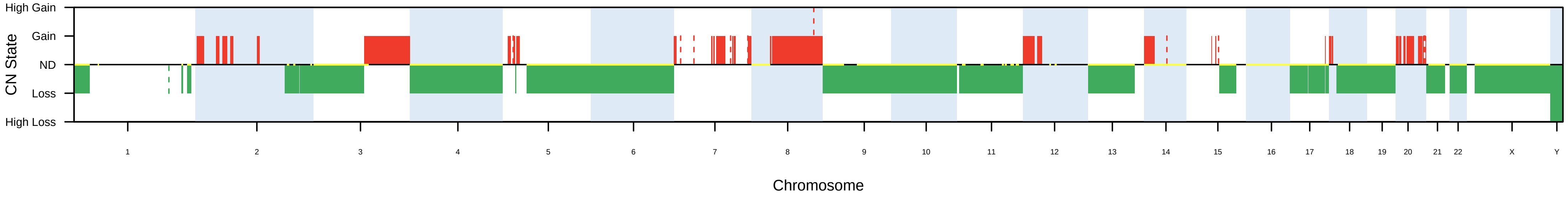

TSB00202−LabB Ploidy=NA %AC=NA MAPD=0.196 ndSNPQC=40.8

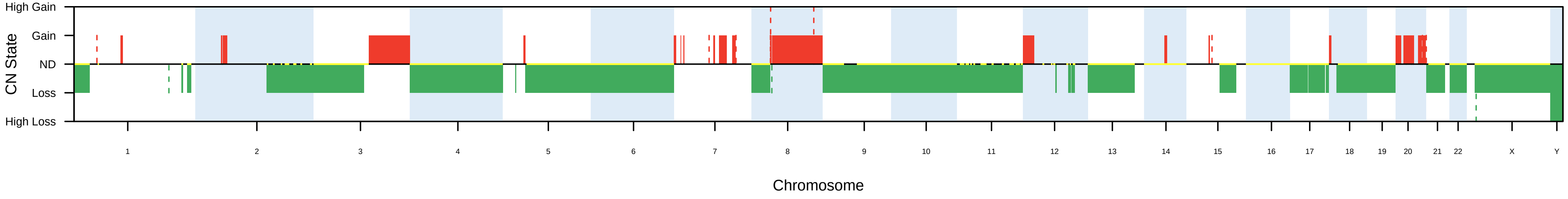

TSB00202−LabC Ploidy=NA %AC=NA MAPD=0.224 ndSNPQC=30.9

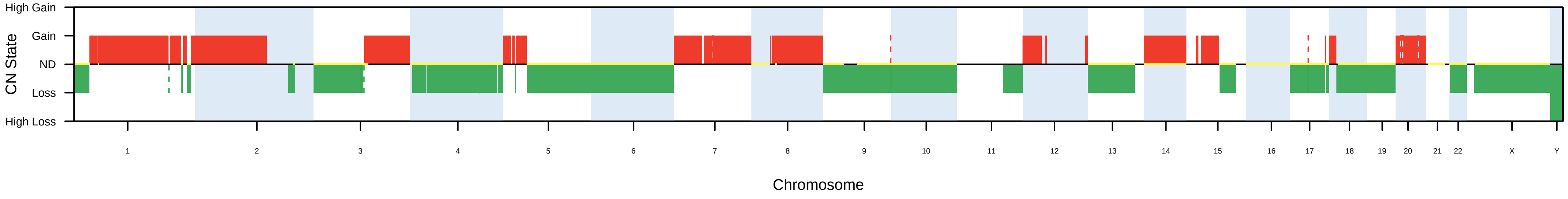

CN Agreement: TSB00202. GW−CN−Call−Agreement=66.6% GW−LOH−Call−Agreement=95.6%

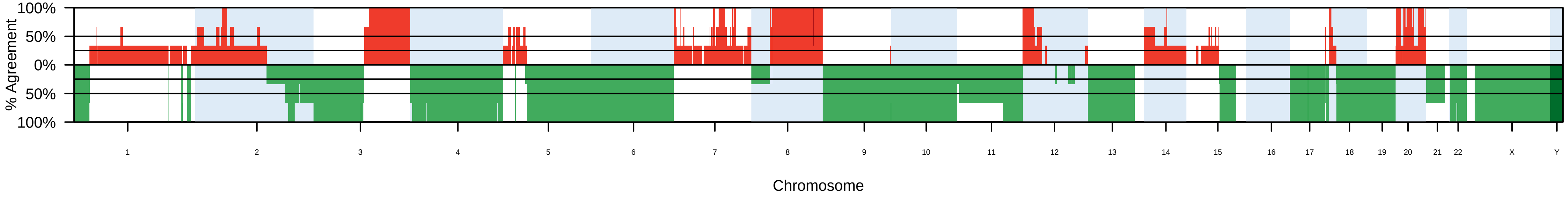

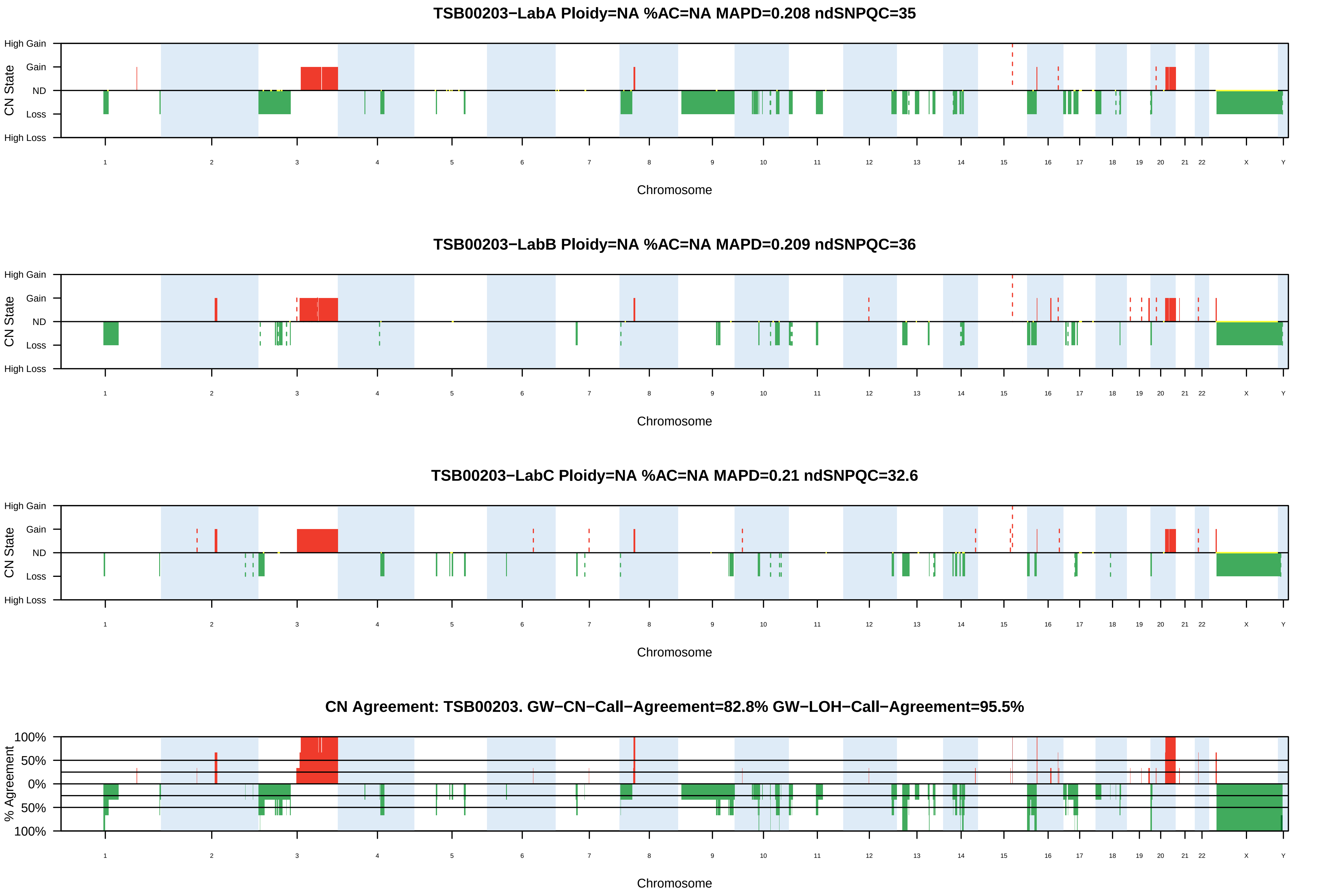

TSB00203−LabA Ploidy=NA %AC=NA MAPD=0.208 ndSNPQC=35
TSB00203−LabB Ploidy=NA %AC=NA MAPD=0.209 ndSNPQC=36
TSB00203−LabC Ploidy=NA %AC=NA MAPD=0.21 ndSNPQC=32.6
CN Agreement: TSB00203. GW−CN−Call−Agreement=82.8% GW−LOH−Call−Agreement=95.5%
CN State
High Gain
Gain
ND
Loss
High Loss
% Agreement
100%
50%
0%
50%
100%
Chromosome
1 2 3 4 5 6 7 8 9 10 11 12 13 14 15 16 17 18 19 20 21 22 X Y

TSB00204−LabA Ploidy=2 %AC=45 MAPD=0.221 ndSNPQC=36.7

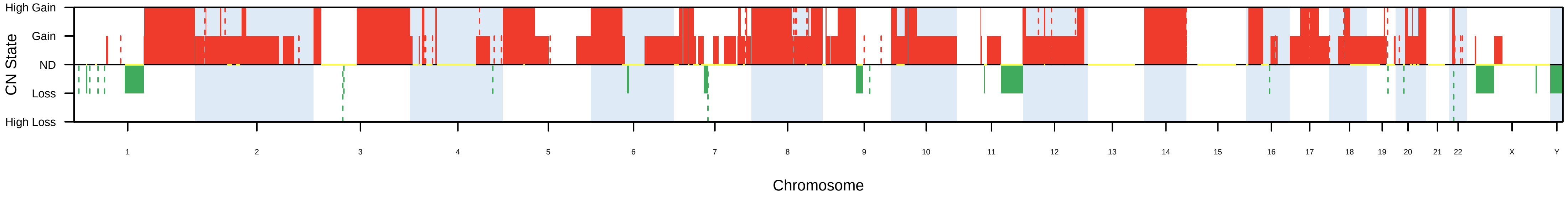

TSB00204−LabB Ploidy=2 %AC=45 MAPD=0.219 ndSNPQC=38

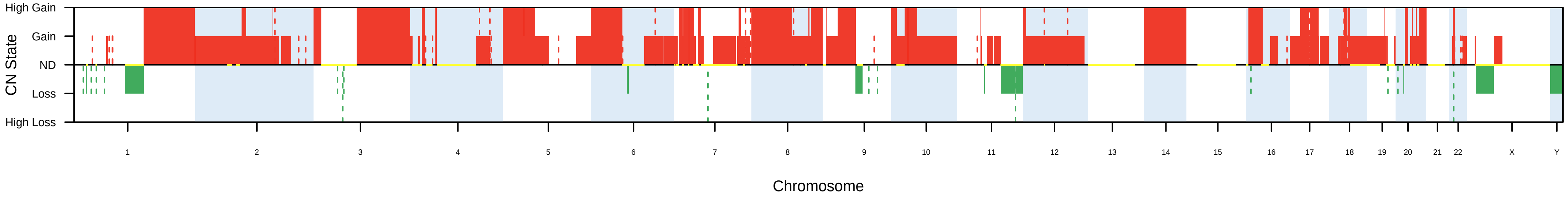

TSB00204−LabC Ploidy=2 %AC=45 MAPD=0.226 ndSNPQC=31.6

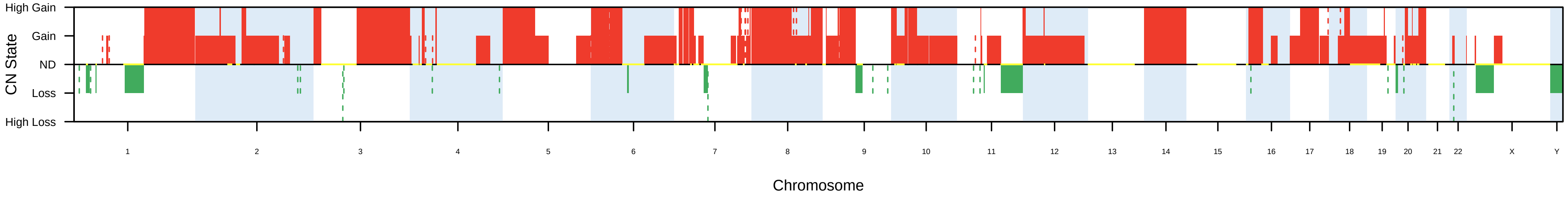

CN Agreement: TSB00204. GW−CN−Call−Agreement=92.6% GW−LOH−Call−Agreement=99%

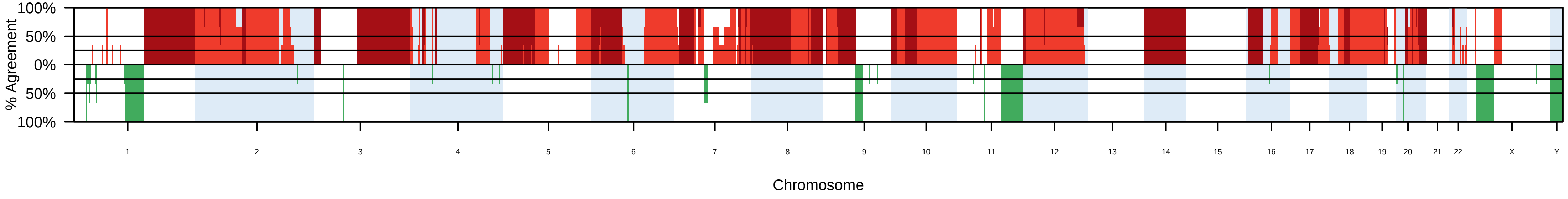

TSB00206−LabA Ploidy=2 %AC=homogeneous MAPD=0.199 ndSNPQC=34.6

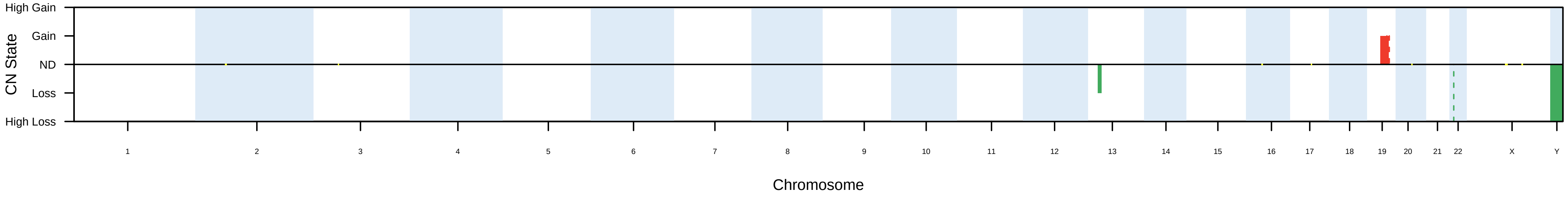

TSB00206−LabB Ploidy=NA %AC=NA MAPD=0.27 ndSNPQC=13.5

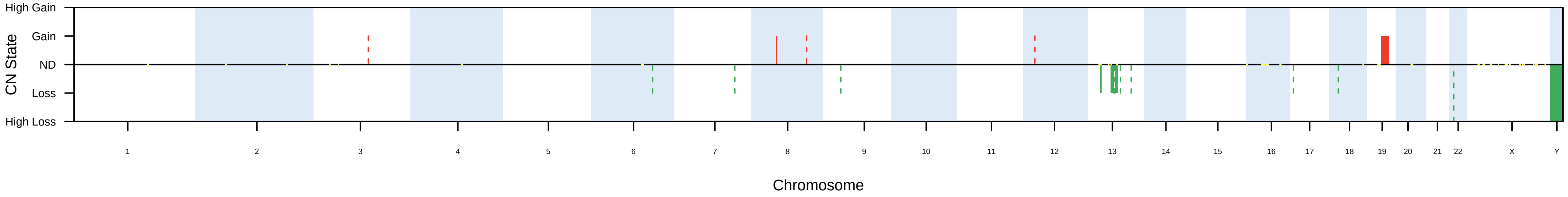

TSB00206−LabC Ploidy=2 %AC=homogeneous MAPD=0.208 ndSNPQC=27.6

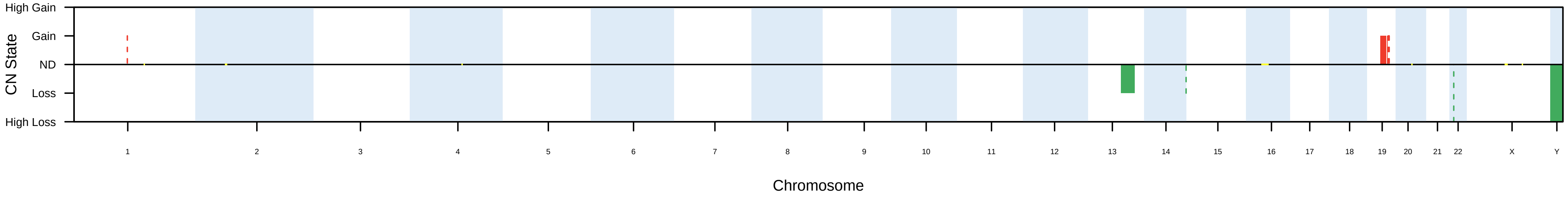

CN Agreement: TSB00206. GW−CN−Call−Agreement=97.5% GW−LOH−Call−Agreement=96.1%

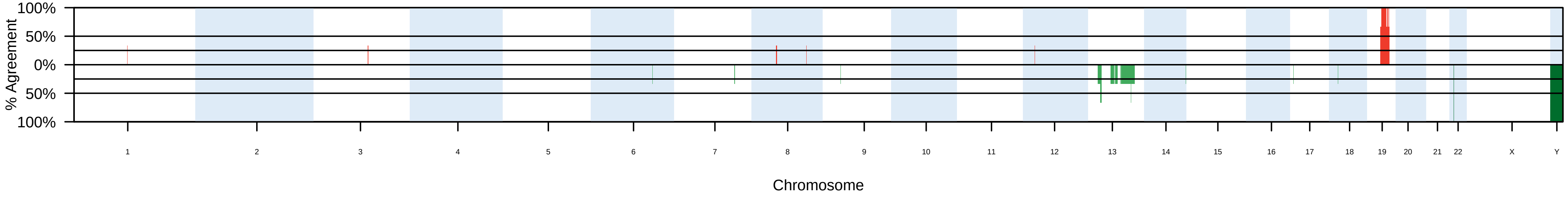

TSB00207−LabA Ploidy=NA %AC=NA MAPD=0.236 ndSNPQC=25.3

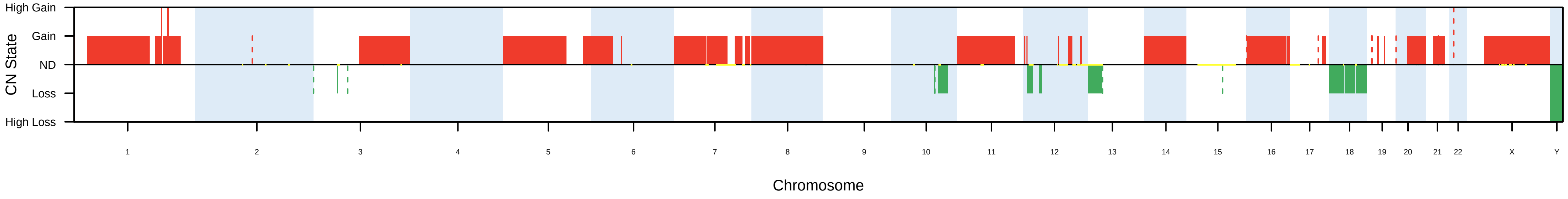

TSB00207−LabB Ploidy=NA %AC=NA MAPD=0.232 ndSNPQC=26.2

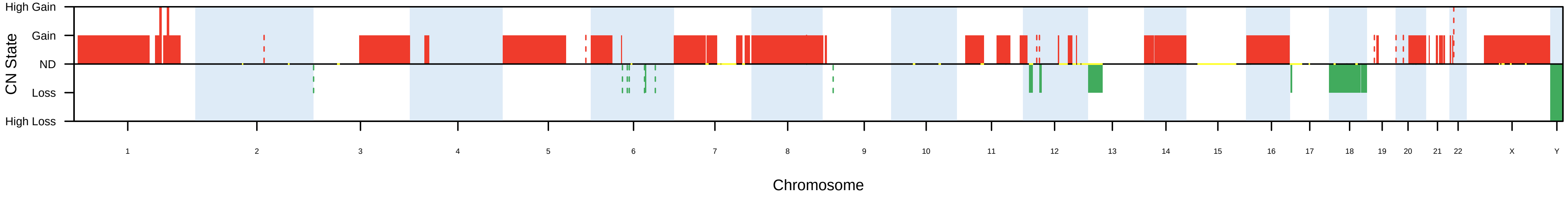

TSB00207−LabC Ploidy=NA %AC=NA MAPD=0.252 ndSNPQC=22.3

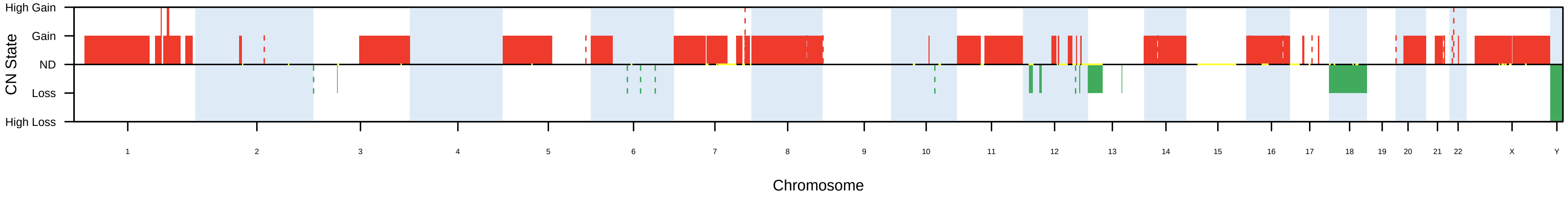

CN Agreement: TSB00207. GW−CN−Call−Agreement=86.8% GW−LOH−Call−Agreement=97.7%

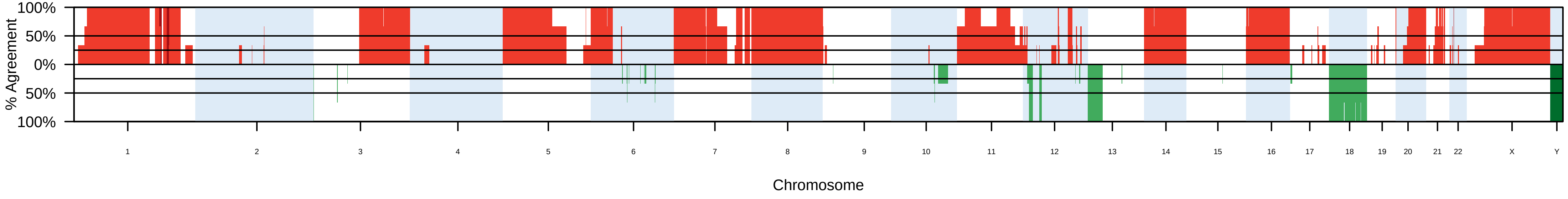

TSB00208−LabA Ploidy=NA %AC=NA MAPD=0.236 ndSNPQC=33.1

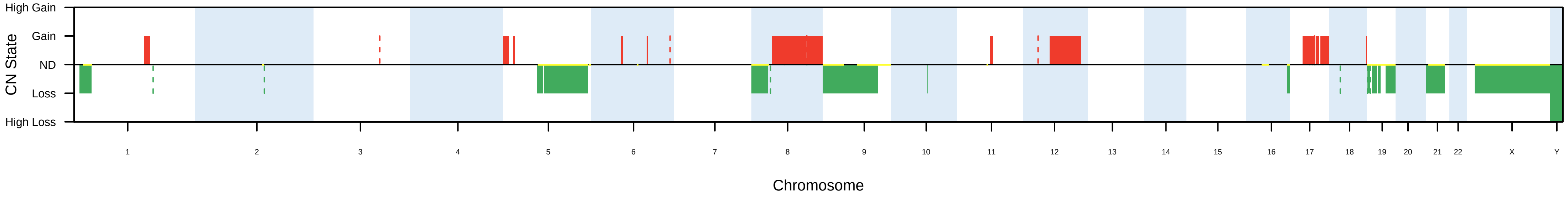

TSB00208−LabB Ploidy=NA %AC=NA MAPD=0.234 ndSNPQC=33.9

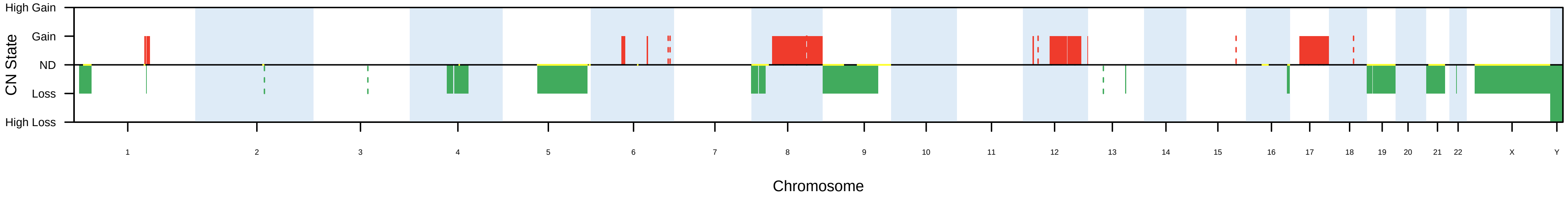

TSB00208−LabC Ploidy=2 %AC=30 MAPD=0.225 ndSNPQC=29.8

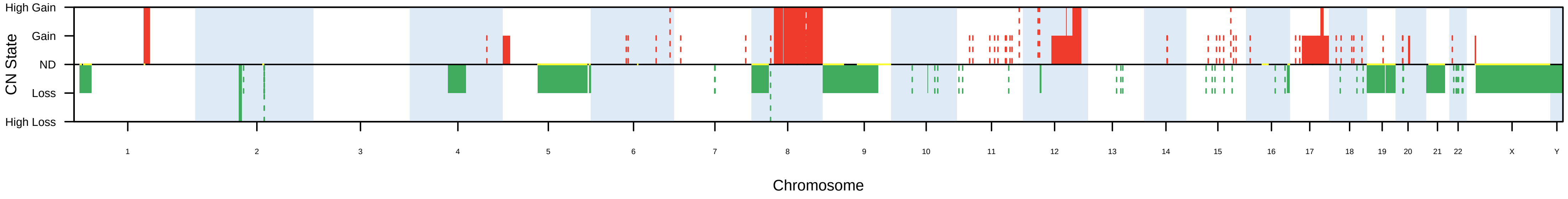

CN Agreement: TSB00208. GW−CN−Call−Agreement=88.7% GW−LOH−Call−Agreement=99.4%

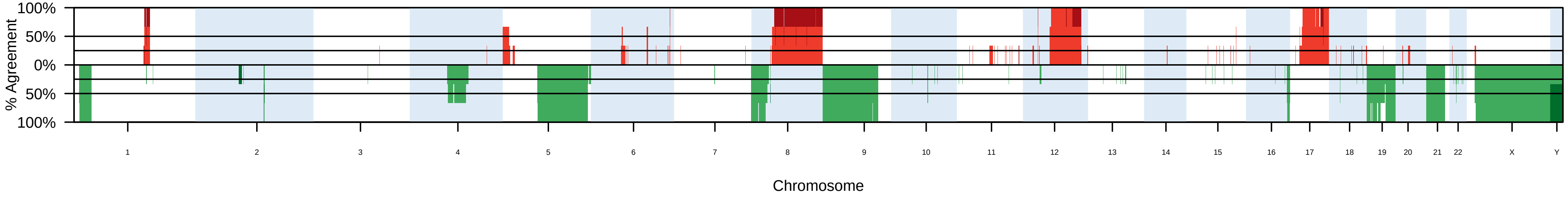

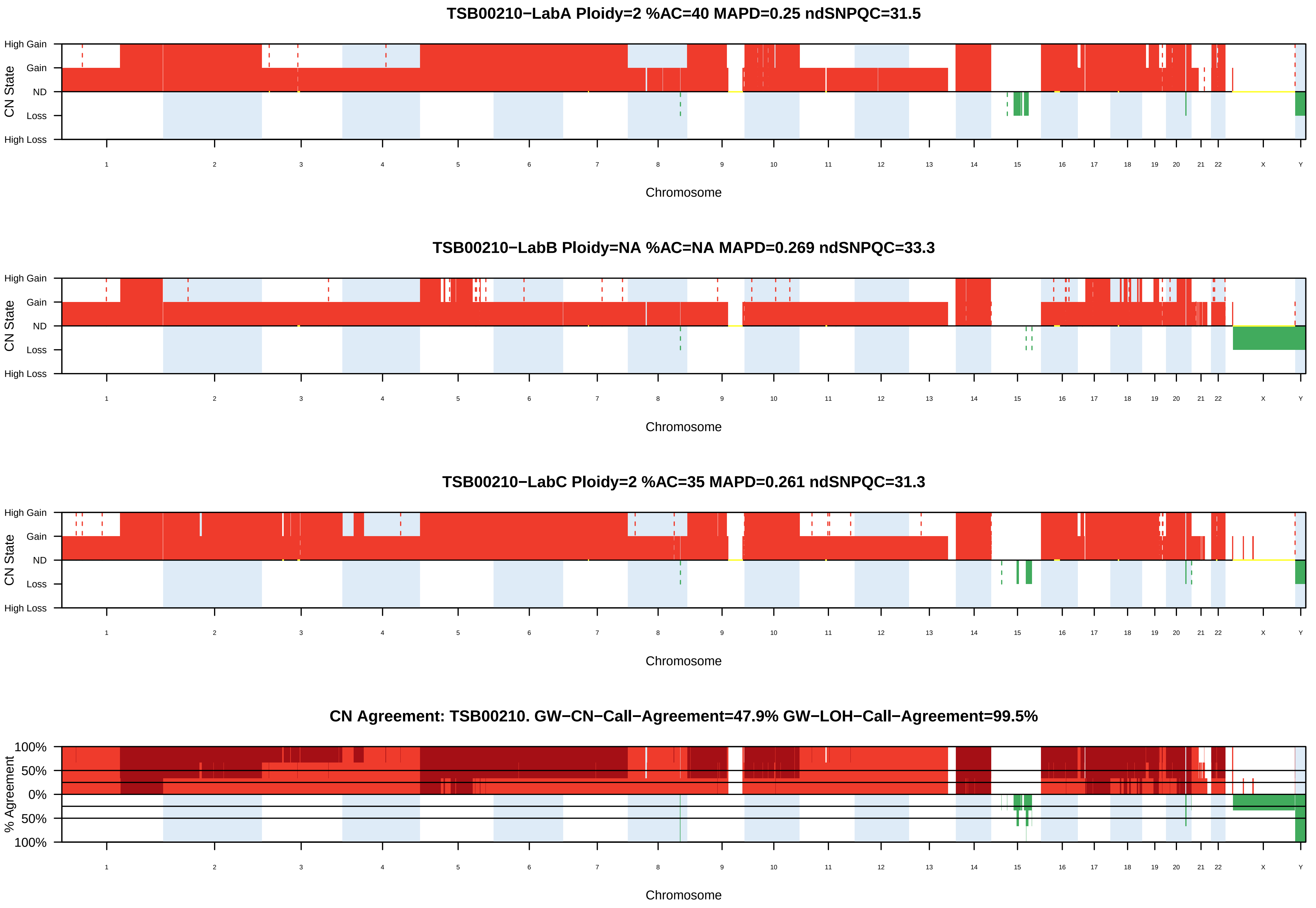
TSB00210−LabA Ploidy=2 %AC=40 MAPD=0.25 ndSNPQC=31.5
High Gain
Gain
ND
Loss
High Loss
CN State
1 2 3 4 5 6 7 8 9 10 11 12 13 14 15 16 17 18 19 20 21 22 X Y
Chromosome
TSB00210−LabB Ploidy=NA %AC=NA MAPD=0.269 ndSNPQC=33.3
High Gain
Gain
ND
Loss
High Loss
CN State
1 2 3 4 5 6 7 8 9 10 11 12 13 14 15 16 17 18 19 20 21 22 X Y
Chromosome
TSB00210−LabC Ploidy=2 %AC=35 MAPD=0.261 ndSNPQC=31.3
High Gain
Gain
ND
Loss
High Loss
CN State
1 2 3 4 5 6 7 8 9 10 11 12 13 14 15 16 17 18 19 20 21 22 X Y
Chromosome
CN Agreement: TSB00210. GW−CN−Call−Agreement=47.9% GW−LOH−Call−Agreement=99.5%
100%
50%
0%
50%
100%
% Agreement
1 2 3 4 5 6 7 8 9 10 11 12 13 14 15 16 17 18 19 20 21 22 X Y
Chromosome

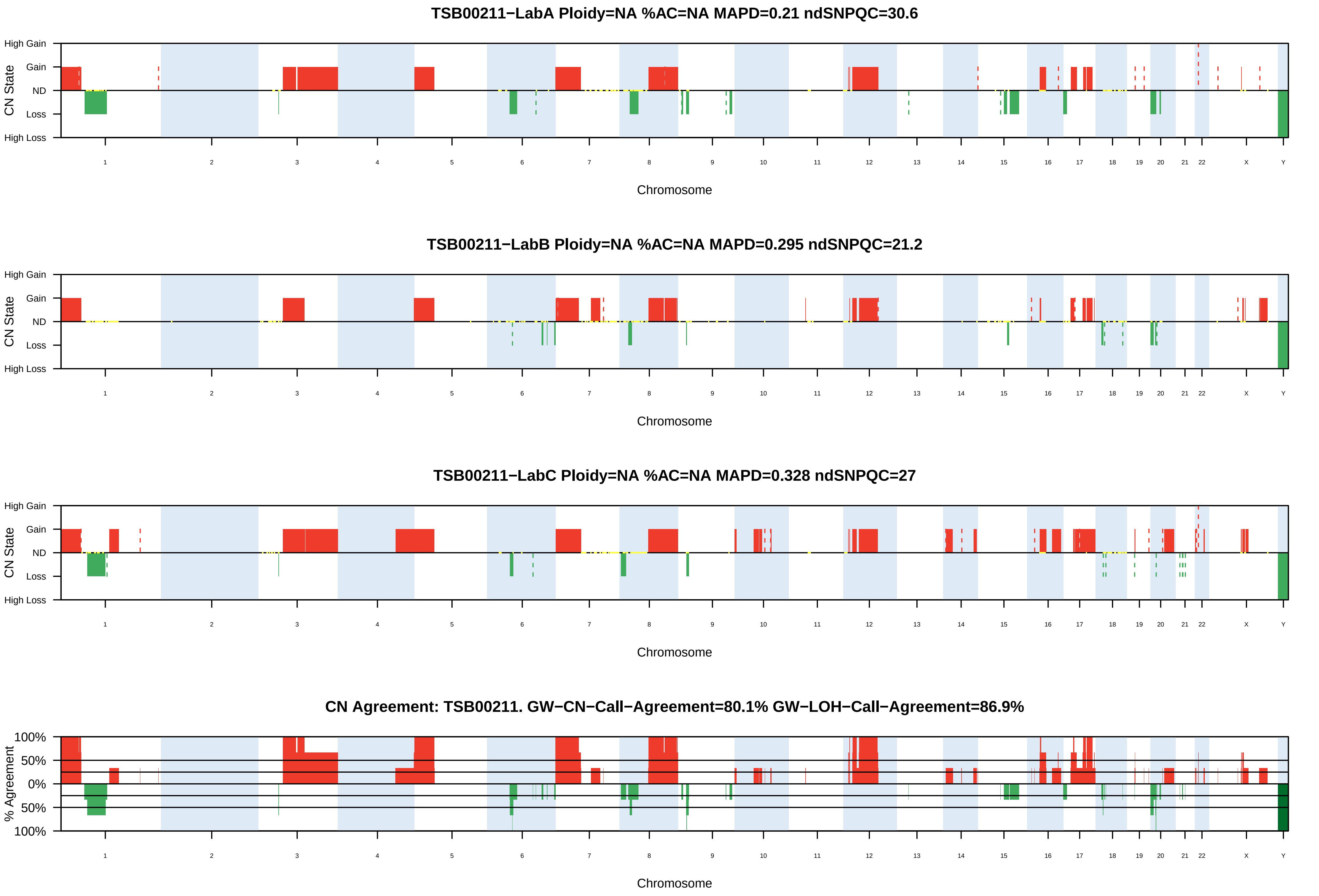
TSB00211−LabA Ploidy=NA %AC=NA MAPD=0.21 ndSNPQC=30.6
TSB00211−LabB Ploidy=NA %AC=NA MAPD=0.295 ndSNPQC=21.2
TSB00211−LabC Ploidy=NA %AC=NA MAPD=0.328 ndSNPQC=27
CN Agreement: TSB00211. GW−CN−Call−Agreement=80.1% GW−LOH−Call−Agreement=86.9%
CN State
High Gain
Gain
ND
Loss
High Loss
% Agreement
100%
50%
0%
50%
100%
1 2 3 4 5 6 7 8 9 10 11 12 13 14 15 16 17 18 19 20 21 22 X Y
Chromosome

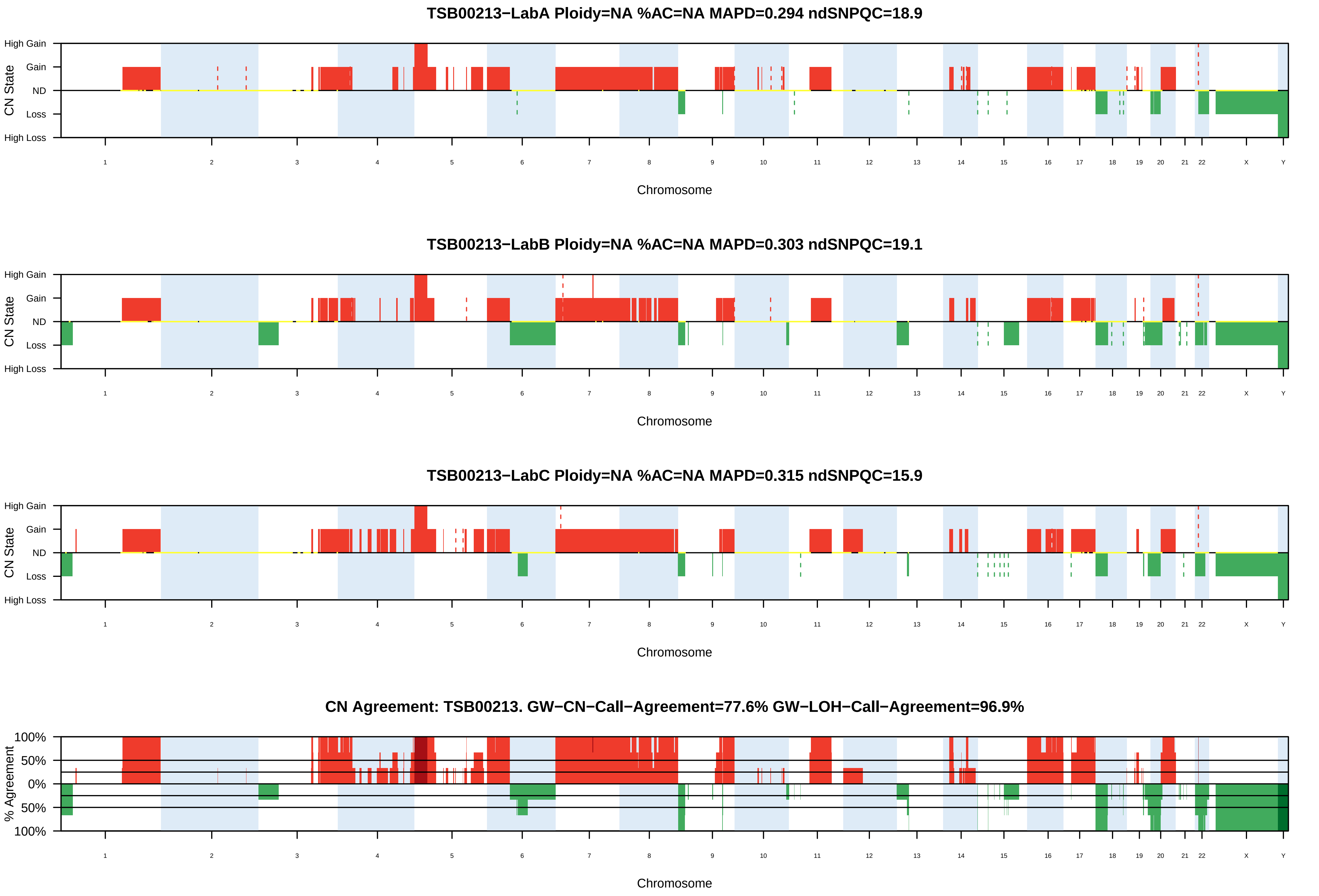
TSB00213−LabA Ploidy=NA %AC=NA MAPD=0.294 ndSNPQC=18.9
High Gain
Gain
ND
Loss
High Loss
CN State
1 2 3 4 5 6 7 8 9 10 11 12 13 14 15 16 17 18 19 20 21 22 X Y
Chromosome
TSB00213−LabB Ploidy=NA %AC=NA MAPD=0.303 ndSNPQC=19.1
High Gain
Gain
ND
Loss
High Loss
CN State
1 2 3 4 5 6 7 8 9 10 11 12 13 14 15 16 17 18 19 20 21 22 X Y
Chromosome
TSB00213−LabC Ploidy=NA %AC=NA MAPD=0.315 ndSNPQC=15.9
High Gain
Gain
ND
Loss
High Loss
CN State
1 2 3 4 5 6 7 8 9 10 11 12 13 14 15 16 17 18 19 20 21 22 X Y
Chromosome
CN Agreement: TSB00213. GW−CN−Call−Agreement=77.6% GW−LOH−Call−Agreement=96.9%
100%
50%
0%
50%
100%
% Agreement
1 2 3 4 5 6 7 8 9 10 11 12 13 14 15 16 17 18 19 20 21 22 X Y
Chromosome

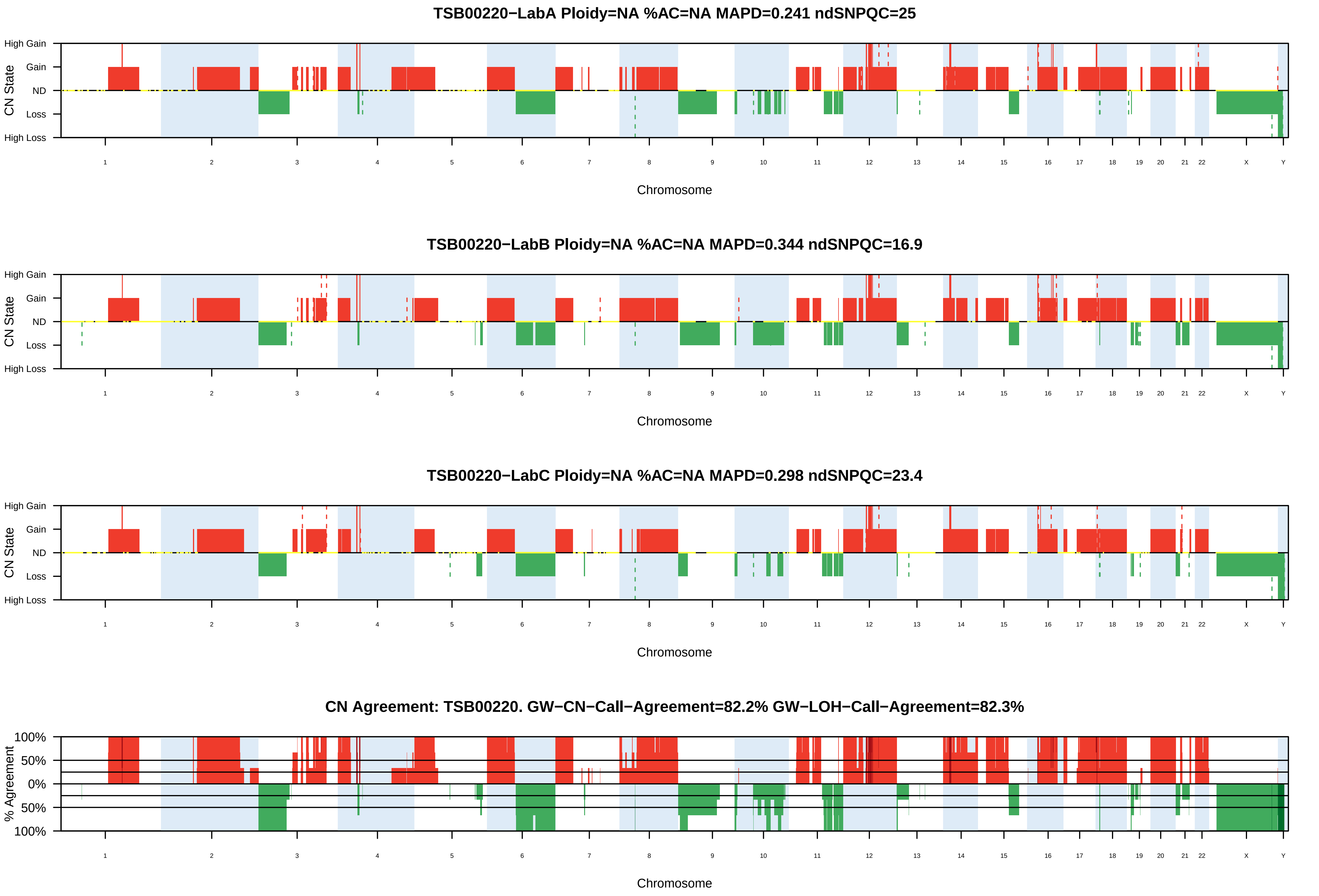

TSB00220−LabA Ploidy=NA %AC=NA MAPD=0.241 ndSNPQC=25
TSB00220−LabB Ploidy=NA %AC=NA MAPD=0.344 ndSNPQC=16.9
TSB00220−LabC Ploidy=NA %AC=NA MAPD=0.298 ndSNPQC=23.4
CN Agreement: TSB00220. GW−CN−Call−Agreement=82.2% GW−LOH−Call−Agreement=82.3%
CN State
High Gain
Gain
ND
Loss
High Loss
% Agreement
100%
50%
0%
Chromosome
1 2 3 4 5 6 7 8 9 10 11 12 13 14 15 16 17 18 19 20 21 22 X Y

TSB00222−LabA Ploidy=NA %AC=NA MAPD=0.206 ndSNPQC=33.3

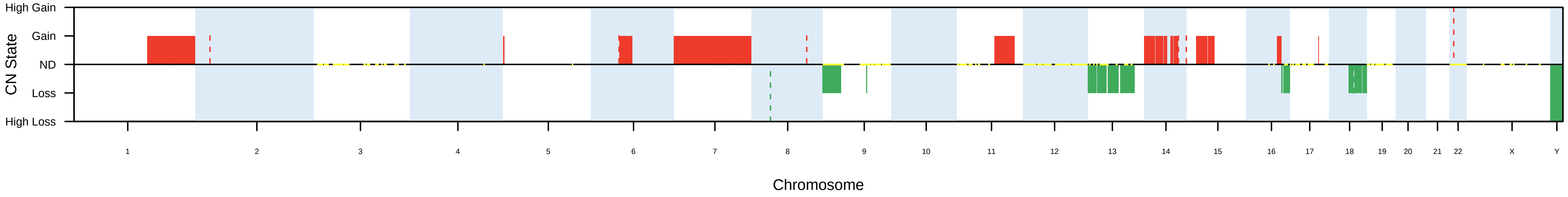

TSB00222−LabB Ploidy=NA %AC=NA MAPD=0.173 ndSNPQC=39.1

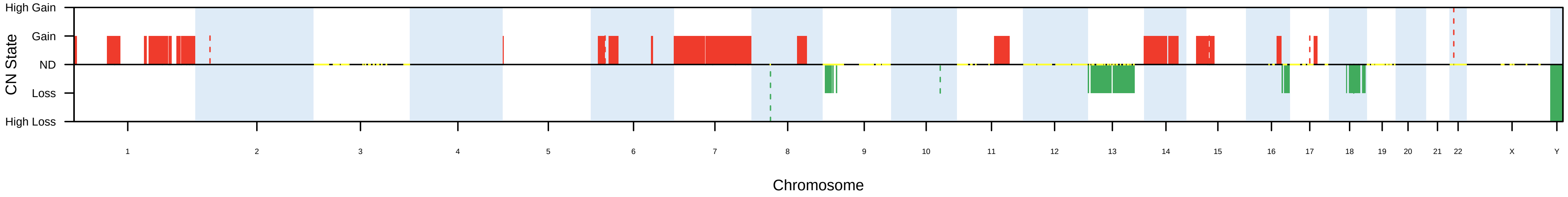

TSB00222−LabC Ploidy=NA %AC=NA MAPD=0.204 ndSNPQC=31.5

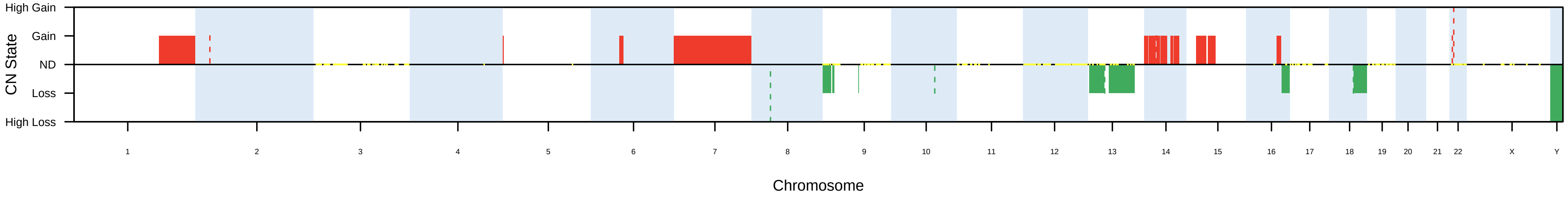

CN Agreement: TSB00222. GW−CN−Call−Agreement=89.1% GW−LOH−Call−Agreement=92.3%

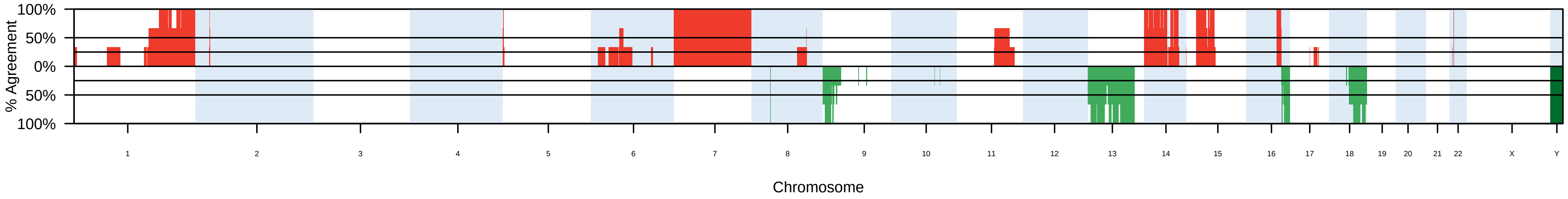

Supplement: Additional file 2: — For each sample run in triplicate (once at each of the three test laboratories) the CN/LOH profile is plotted alongside a CN agreement plot that displayed the % of the 3 samples that agree on a given CN event. [file 12920_2015_79_MOESM2_ESM.pdf]
